# Listening-Based Communication Ability in Adults With Hearing Loss: A Scoping Review of Existing Measures

**DOI:** 10.3389/fpsyg.2022.786347

**Published:** 2022-03-10

**Authors:** Katie Neal, Catherine M. McMahon, Sarah E. Hughes, Isabelle Boisvert

**Affiliations:** ^1^Department of Lingustics, Macquarie University, Sydney, NSW, Australia; ^2^Hearing, Macquarie University, Sydney, NSW, Australia; ^3^Centre for Patient Reported Outcome Research, Institute of Applied Health Research, University of Birmingham, Birmingham, United Kingdom; ^4^National Institute of Health Research (NIHR), Applied Research Collaboration (ARC), West Midlands, United Kingdom; ^5^Faculty of Medicine, Health and Life Science, Swansea University, Swansea, United Kingdom; ^6^Sydney School of Health Sciences, Faculty of Medicine and Health, The University of Sydney, Sydney, NSW, Australia

**Keywords:** listening, communication ability, hearing loss, adults, scoping review, outcome measure

## Abstract

**Introduction:**

Hearing loss in adults has a pervasive impact on health and well-being. Its effects on everyday listening and communication can directly influence participation across multiple spheres of life. These impacts, however, remain poorly assessed within clinical settings. Whilst various tests and questionnaires that measure listening and communication abilities are available, there is a lack of consensus about which measures assess the factors that are most relevant to optimising auditory rehabilitation. This study aimed to map current measures used in published studies to evaluate listening skills needed for oral communication in adults with hearing loss.

**Methods:**

A scoping review was conducted using systematic searches in Medline, EMBASE, Web of Science and Google Scholar to retrieve peer-reviewed articles that used one or more linguistic-based measure necessary to oral communication in adults with hearing loss. The range of measures identified and their frequency where charted in relation to auditory hierarchies, linguistic domains, health status domains, and associated neuropsychological and cognitive domains.

**Results:**

9121 articles were identified and 2579 articles that reported on 6714 discrete measures were included for further analysis. The predominant linguistic-based measure reported was word or sentence identification in quiet (65.9%). In contrast, discourse-based measures were used in 2.7% of the articles included. Of the included studies, 36.6% used a self-reported instrument purporting to measures of listening for communication. Consistent with previous studies, a large number of self-reported measures were identified (*n* = 139), but 60.4% of these measures were used in only one study and 80.7% were cited five times or fewer.

**Discussion:**

Current measures used in published studies to assess listening abilities relevant to oral communication target a narrow set of domains. Concepts of communicative interaction have limited representation in current measurement. The lack of measurement consensus and heterogeneity amongst the assessments limit comparisons across studies. Furthermore, extracted measures rarely consider the broader linguistic, cognitive and interactive elements of communication. Consequently, existing measures may have limited clinical application if assessing the listening-related skills required for communication in daily life, as experienced by adults with hearing loss.

## Introduction

Communication forms the foundation of social interaction. For adults, communication is recognised as a critical component to adapting and adjusting to aging, essential to maintaining independence and personal relationships, performing social roles and functions, making decisions and having control over life quality (Heinrich et al., 2016). While language use and structure change across the life span, conversational skills are generally preserved in typically aging adults (Shadden, 1988). Aging, however, is associated with an increased prevalence of conditions that affect communication, of which hearing loss is the most prevalent (Wallhagen and Pettengill, 2008). The effect of impaired communication is linked to several aspects of social relationships and psychological well-being. For example, Palmer et al. (2019) demonstrated that communication impairment is an independent predictor for reduced social integration and participation, increased levels of loneliness and depression, and reduced social self-efficacy. Findings from this work are not isolated, Keidser et al. (2015) and Sung et al. (2016) emphasise the importance of communication as the conduit for social connection and its associated health and well-being impacts.

Oral communication is dynamic, spanning multiple interconnected domains of hearing, listening, language and cognition and is overlayed by contextual nuances that make up real-world communication. Listening experiences underpin the development and use of this dynamic complex (Nittrouer, 2002; Kuhl and Rivera-Gaxiola, 2008); hence, disruptions in listening caused by hearing loss can have broad impacts across this communication complex. The significant gap between traditional measures of hearing loss, such as hearing thresholds, and the pervasive expression of its effects across oral communication and social participation for an individual (Ambert-Dahan et al., 2018; Lin, 2020) fails to provide individuals (or their hearing healthcare professionals) with an understanding of one’s full communication capacity (Manchaiah, 2017).

For adults with hearing loss, listening and communication ability are rarely measured in the context they are experienced (Beechey et al., 2019). From a diagnostic and device fitting perspective, standards are principally and necessarily focused toward measures of *hearing impairment* that enable a comparable numeric representation of hearing acuity. Assessments such as audiometric threshold measures provide a sensitive and valid representation of changes within the auditory pathway. However, these measures are associated with the integrity of the peripheral auditory pathway, thereby separating hearing from its role as part of a complex brain network, one that both precedes and provides the basis for listening (Stewart and Arnold, 2018). Clinically, the limitations of hearing measurement are commonly addressed with the inclusion of speech audiometry, which requires the listener to repeat single words or brief sentences. While also sensitive to changes in auditory function, speech-based measures involve the engagement of components of the complex brain network of listening, such as attention and linguistic knowledge. It is therefore logical to infer that this type of assessment adequately reflects the requirements of listening for communication.

Effective communication relates to the complex and interwoven systems that enable adults to participate, ask and answer questions, comment and understand indirect and often abstract language. To achieve this, adults need to be competent across the linguistic, social, and cognitive complexes that define and constitute communication. Additionally, real-world processing of acoustic information is strongly influenced by environmental, linguistic, contextual and production (speaker) factors (Gifford and Revit, 2010; Klatte et al., 2010). These factors affect the interpretation of speech signals and require cognitive mechanisms to engage, compensate and resolve frequent ambiguity (Rönnberg et al., 2013; Guediche et al., 2014; Baskent et al., 2016). Understanding this relationship has become an increasingly important consideration in the field of hearing, as listeners vary significantly in their ability to understand speech in complex environments and traditional audiological assessment can only partly explain this variation (Pichora-Fuller, 2003; Anderson and Kraus, 2010; Rönnerg et al., 2016).

Defining listening function in terms of a dynamic communicative complex has broad implications for both the individual and clinical practice. A reductionist conceptualisation of listening focussed on hearing impairment not only limits our understanding of how listening is experienced for an individual but may also fail to demonstrate the impacts of hearing impairment as a social, health and economic priority (Deloitte Access Economics, 2017; World Health Organization, 2017). In general, clinical audiology services are increasingly aware of the need to adapt hearing evaluations toward a more person-centred ideal (Boisvert et al., 2017). Measures that fully explore and provide an understanding of an individual’s needs and prognosis in relation to different audiological interventions, however, seem to be lacking, which can affect the adoption and development of technology and rehabilitation programs (Rudner, 2016; Hughes et al., 2017).

The concern about the limitations of existing measures to adequately assess communication function in adults with hearing loss is not new (Cox et al., 2000; Moberly et al., 2018a). It is unclear, however, how knowledge of these limitations has influenced recent studies that assess functional abilities in adults with hearing loss. While self-report instruments have been identified as measures that could bridge assessment gaps (Rivera et al., 2019; Shao et al., 2020), the constructs of listening and communication do not appear to be well conceptualised within existing self-reported measures for adults with hearing loss. In view of this, this scoping review aimed to identify measures used in recently published studies to evaluate skills that are necessary for oral communication in adults with hearing loss, and to map these measures in relation to constructs of listening and communication to assess potential gaps or biases in measurement.

## Methodology

This study used a systematic scoping review approach guided by the Preferred Reporting Items for Systematic Reviews and Meta-Analysis extension for Scoping Reviews [PRISMA-ScR; 22] (Tricco et al., 2018).

### Eligibility Criteria

Published studies were included in this review if participants were adults (18 years and over) who reported or had been identified as having any hearing difficulty. The assessments used within the study had to meet the following criteria: 1) linguistic-based measurement relevant to oral communication, AND 2) behavioural or self-report measures of listening abilities, with listening ability defined as the conscious processing and response to an auditory stimulus. Cognitive assessments that included an auditory function element in the assessment of abilities [for example: Montreal Cognitive Assessment (MoCA)] were also included. Studies measuring vestibular function, tinnitus or hyperacusis (classified as additional symptoms as opposed to hearing or listening ability), device output measures, measures of hearing sensitivity only (e.g., detection thresholds), detection-based localisation, physiological or anatomical measures, and music-based measures that did not include a behavioural or self-reported linguistic measure of listening ability relevant to oral communication were excluded. To focus the review on listening assessments that were more likely to be used with hard-of-hearing adult participants, studies that included both paediatric and adult data were excluded as were studies with a sample size of fewer than ten hard-of-hearing adults.

### Information Sources

A systematic search of databases [Medline (Ovid), EMBASE (Ovid), Web of Science Core Collection (Web of Science) and Google Scholar] was initially performed in September 2018 and repeated in December 2019. This combination of four databases was selected in accordance with Bramer et al. (2017) findings which demonstrated a retrieval performance of 98.3% for systematic searches using this combination. Search terms and strategy were devised and supported with the assistance of a research librarian at Macquarie University. Keyword and related MeSH terms relevant to ‘oral-communication’, ‘listening’ and ‘hearing’ were combined with terms associated with ‘hearing loss’ and ‘measurement’. The search strategy was limited by year of publication (2008-current) to focus on contemporary studies, and avoid duplication with a previous comprehensive systematic review of hearing outcome measures (Granberg et al., 2014). Publication language was limited to English; however, the assessment language was not restricted in the search criteria. The final search strategy applied with Medline (Ovid) is shown in Supplementary Material 1. The results of the searches were uploaded into the reference management software, Endnote X9.2 (Clarivate Analytics, Boston, MA, United States). Duplicates were removed and the remaining abstracts imported into Covidence (Covidence^1^) online systematic review management software. Deduplication was repeated in Covidence to ensure all duplicate records were removed prior to screening.

### Selection of Sources of Evidence

The main author (KN) and two research assistants (RF, RK) were involved in the screening of studies against the eligibility criteria. Each study was independently screened by a minimum of two reviewers. An initial screening of titles and abstracts was conducted to remove records of studies that were out of scope for this review. Full-text screening was conducted for the remaining records. Excluded records were labelled with a reason for their exclusion. Reviewers flagged any study that did not clearly meet the inclusion or exclusion criteria. Reasons for ambiguity, such as studies that indicated audiological or functional assessment but did not specify the measurements used, were labelled accordingly and retained or removed following a discussion between the reviewers. Persistent discrepancies at all stages were managed in consultation with a third reviewer (IB), with final decisions regarding study inclusion or exclusion reached through consensus-based discussion. Because this review aimed to identify measures used within published studies, critical appraisal of the methodological quality of the included studies was not considered relevant to the aims of the review and not undertaken.

### Data Charting Process

All eligible studies were charted independently by two members of the review team. Percentage agreement was used to determine inter-rater agreement and consistency. This was set as a minimum of 90% agreement, that is, 10% or less of charted items being categorised as a conflict (McHugh, 2012). Unclear or ambiguous information about measures used within a study was clarified by retrieving and reviewing the source measure (for example, the specific questionnaire used within a study).

#### Coding Framework and Data Items

Data charting focused on extracting details of the assessment measures used in each study and study-specific information. Charting of assessment measures began by using the study tags within Covidence, and the charting of items was further refined using Microsoft Excel (2020). A bespoke coding framework to support data-charting was developed and piloted with 300 studies before being refined. All piloted studies were rescreened by two reviewers (KN, RF) to ensure that the refined coding scheme captured the relevant components. The coding framework (Table 1) was designed to categorise measures as: (1) measures of linguistic constructs of functional listening relevant for communication; (2) self-report measures; and (3) cognitive measures.

**TABLE 1:** Coding framework to categorise each measure used within the included studies.

Study charting	Subcategory
Assessment Measures	
Detection (based response)	
Phoneme	Independent Extracted from longer form stimuli
Word/sentence	
Word/sentence context	Quiet Noise
Word/sentence auditory hierarchy	Detection Discrimination Recognition Comprehension None
Discourse	
Linguistic unit	Acceptable noise level judgement Paralinguistic cues Phonology Semantic/Syntactic Suprasegmental Suprasegmental - Tonal language
Self-report measure	
Self-report assessment name	
Self-report category	Auditory Non-Auditory Unclear Condition specific Generic Modifiable
Cognitive measure	
Cognitive measure assessment name	
Cognitive measure administration	Auditory Non-auditory
Cognitive measure neurocognitive domain and/or type	DSM-5 Complex attention DSM-5 Executive function DSM-5 Learning & memory DSM-5 Language DSM-5 Social cognition DSM-5 Perceptual-motor function Unspecified Screening Diagnostic

For linguistic measures, key categories were derived initially based on a hierarchy of language unit components (i.e., from phonemes to discourse) and the level of auditory processing required (Estabrooks et al., 2020). Levels within the auditory hierarchy were defined as speech detection (the awareness of speech sounds), speech discrimination (the detection of changes in the acoustic stimuli), speech identification (the *recognition* of speech sounds, no semantic processing required; repetition of the stimuli), and speech comprehension (attaching meaning to the acoustic stimuli) (Erber, 1982; Thibodeau, 2007). Additional characteristics such as stimulus complexity (i.e., presented in quiet or in noise) were also extracted.

Charting of self-report measures identified hearing-specific measures as well as generic self-report measures that stated or implied the inclusion of auditory items relating to oral communication and functional language use. Charting included characteristics of the self-report measures such as single item, study-specific versus existing measure, and administration mode. Study-specific refers to measures that have been specifically developed or adapted (from existing formal assessment measures) for the purpose of a specific study. Formal measure describes previously published self-report assessments that are used within clinical studies and audiology clinics. All formal self-report measures where included irrespective of the extent of any psychometric evaluation of their measurement properties. When available, the target construct of study-specific measures [e.g., quality of life (QoL) or disability measurement] was extracted. For studies using published questionnaires, this information was reported based on the original description of the assessment, and classified into health status outcome domains. Health status domains reflect the status of individuals, in terms of conditions, functioning, and well-being. Categorisation into health status outcomes was derived from the principal description by the developers of respective measures, or from the description in the included studies from which the data was extracted (Barker et al., 2015; Madans and Webster, 2015). All accessible self-report measures, excluding study-specific measures, were sourced from the studies’ attached appendices, original development papers or through correspondence with authors, for the items (individual questions) of each measure to be extracted for further analysis.

Cognitive measures that included a functional auditory element were identified and coded according to the six neurocognitive domains specified in The Diagnostic and Statistical Manual of Mental Disorders (5*^th^* ed.; DSM-5; American Psychiatric Association, 2013). The six principal domains as stated in the DSM-5 are: complex attention, executive function, learning and memory, language, social cognition and perceptual-motor function. The methods sections of the included articles were used to clarify the targeted cognitive domains for any tests that could be administered in more than one way. For example, the digit span test can be used to assess either forward or backward recall, which relate to different neurocognitive domains. Cognitive *screening* tests, which typically assess multiple domains, and studies in which three or more domain-specific diagnostic measures were used were categorised as *multi-domain measures (screening)* or *multi-domain measures (diagnostic)* respectively. The code “Unspecified” was used when studies did not provide sufficient information to determine the cognitive domain associated with the measures used. Publication details (year of publication), assessment language (English or Non-English), the dataset country of origin, study sample size, and hearing devices used by participants were also charted.

### Data Synthesis

Descriptive analyses were conducted to: (1) provide an overview of the types and frequency of measures used for the assessment of listening and communication in clinical studies; (2) determine if the representation of measurement types changed across time; and to (3) compare the content of assessments and their underlying constructs in comparison with broader constructs of functional listening and communication as described in the literature. Measures using speech-based stimuli were categorised according to: (1) a language unit hierarchy from the phonemic unit (minimal) to the discourse unit (maximal), and (2) an auditory hierarchy from speech detection (minimal) to comprehension (maximal). Division into these units was chosen to reflect the broad terms used to identify speech-based assessment material, the associated complexities related to appraising the details of the stimulus used (phoneme, word, sentence, discourse), and what was measured in relation to the task requested from the listener (imitation or comprehension). The distinction between imitation and comprehension, the targeted language unit and the auditory context (quiet/noise) represents different levels of listening complexity and engagement of cognitive mechanisms (Rodd et al., 2012; Moberly and Reed, 2019), factors key to determining the relationship of these measures to functional listening and communication. Data analyses and figures were prepared using a combination of Tableau Public (Tableau Public^2^) and Microsoft Excel (2020).

## Results

### Included Studies

**FIGURE 1:**
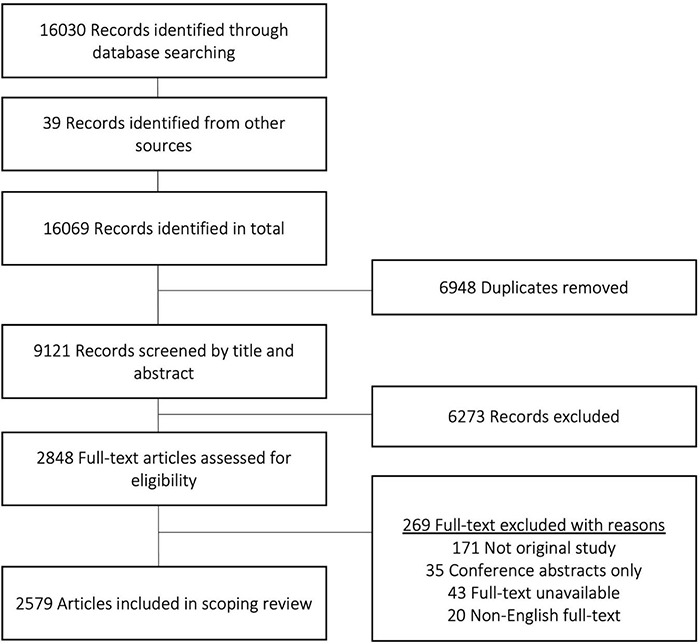
Preferred Reporting Items of Systematic Reviews and Meta-Analyses (PRISMA) flow diagram (Moher et al., 2009).

Details of search results and screening processes are shown in the Preferred Reporting Items for Systematic Reviews (PRISMA) diagram (Figure 1). From 16,069 records identified through the database and grey literature search, 6,948 duplicates were removed. The remaining 9,121 studies’ titles and abstracts were reviewed against the inclusion criteria. Of these, 6,273 studies were excluded. A full text screening of the 2,848 potentially eligible studies resulted in an additional 269 exclusions, leaving 2,579 studies which included adults with hearing difficulties and contained a linguistic measurement relevant to oral communication.

### Study Characteristics

**FIGURE 2:**
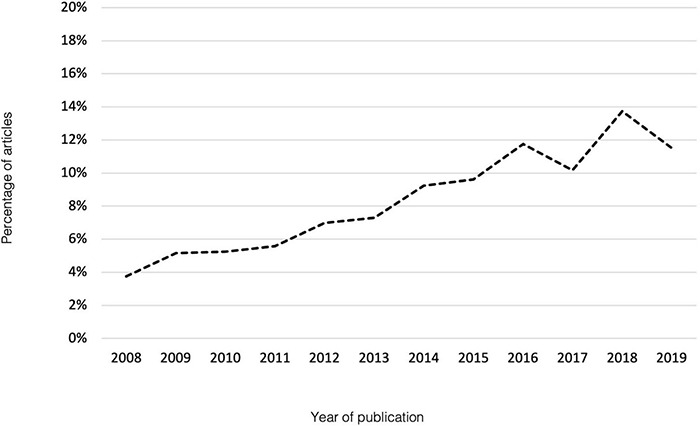
Percentage of included articles by year of publication 2008–2019.

Overall, the number of studies that met the inclusion criteria increased during the period assessed (see Figure 2). Data originated from 41 countries with the United States of America (*n* = 719 articles; 27.9%), the United Kingdom (*n* = 196 articles; 7.6%), and Netherlands (*n* = 172 articles; 6.7%) being the most represented. Two hundred and eighty-three studies (11.0%) presented data collected across multiple countries. Grouping by continent revealed that most publications originated from Europe (*n* = 1023, 39.7%) followed by the Americas (*n* = 928, 36.0%). Within the 2,579 included studies, 34.6% (*n* = 892) of the measures were presented in a language other than English. Participant numbers ranged from 10 (minimum specified in inclusion criteria) to 7,210,535. Most studies used a small number of participants with a group sample size of 10-25 participants accounting for 31.5% and 26–100 participants for 35.7% of studies, respectively. Larger population-based studies (*n* > 1000) were represented 10.1% of the included studies.

### Characteristics of Measures Used Within the Included Studies

**FIGURE 3:**
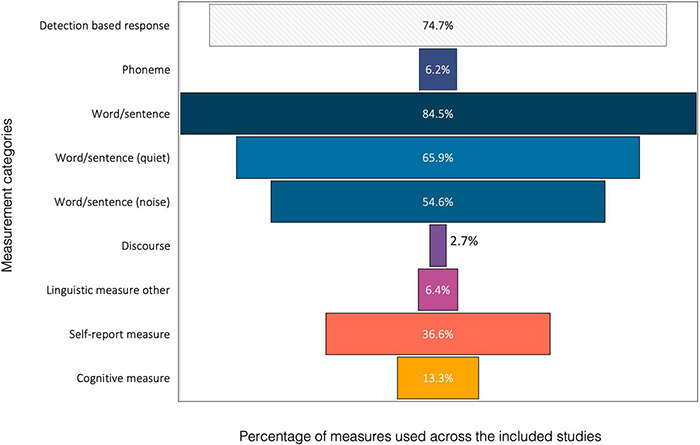
Percentage of assessment measures (total *n* = 6714) by category (vertical axis) identified in the included studies (*n* = 2579 studies). Charting categorisation details for specific measurement categories (Word/Sentence; Linguistic units; Self-report measures; and Cognitive measures) are presented in Table 2.

In total, 6,714 discrete assessment measures were extracted from the 2,579 included studies and charted in relation to the type of measure used (Figure 3) and their linguistic properties (Table 2). Detection-based responses [indicating the presence or absence of stimuli (tonal or other)] though not targeted for this review, were found in 74.7% of the included studies (*n* = 1927/2579).

**TABLE 2:** A. Word and sentence measures by auditory hierarchy; B. Linguistic measures by linguistic domain; C. Self-report measures by health status domain; and D. Cognitive measures by neuropsychological cognitive domain. Word/sentence measures are depicted as a total group (Word/sentence) and by presentation in either quiet [Word/sentence (quiet)] or noise [Word/sentence (noise)]. Percentages exceed 100% due to multiple measures used within studies.

A Word/sentence by auditory hierarchy (*n* = 2178 ST)		
	N	%
Speech detection	5	0.2%
Speech discrimination	132	6.1%
Speech recognition	1968	90.4%
Speech comprehension	72	3.3%

**B Linguistic units by domain (*n* = 165 ST)**		

ANLJ	30	18.2%
Paralinguistic	16	9.7%
Phonology	17	10.3%
Semantic/syntactic	43	26.1%
Suprasegmental (non-tonal language)	47	28.5%
Suprasegmental (tonal language)	12	7.3%

**C Self-report by health status domain (*n* = 945 ST)**		

Communication	18	1.3%
Device benefit	277	21.2%
Disability (condition specific)	408	31.2%
Disability (generic)	50	3.8%
Health	36	2.7%
Other	2	0.1%
Physiological	1	0.1%
Psychological	64	4.9%
Quality of Life	135	10.3%

**D Cognitive measures by domain (*n* = 343 ST)**		

Complex attention	103	30.7%
Executive function	142	41.4%
Learning & memory	50	14.6%
Language	70	20.4%
Social cognition	7	2.0%
Perceptual-motor function	14	4.1%
Unspecified domain	6	1.7%
Single domain	87	25.4%
Multidomain diagnostic assessment	135	39.4%
Screening (multidimensional)	115	33.5%

### Speech-Based Measures

The majority of studies (*n* = 2178/2579, 84.5%) included a word or sentence measure, which accounted for 32.4% (*n* = 6714) of the total measures identified. The most frequently used language unit was word or sentence identification presented in quiet (WSQ) (*n* = 1699/2579; 65.9%) followed by word or sentence identification in noise (WSN) (*n* = 1407/2579; 54.6%). Discourse-based measures, that extend beyond a single sentence and reflect the form and function of language in the social context, had the smallest representation with only 2.7% (*n* = 69/2579) of studies. One-hundred and fifty-nine studies (6.2%) used a phonemic (smallest language unit) measure. The phoneme-based measures were from studies that specifically stated the use of phonemes as an individual measure or directly reported on phonemic outcomes as a separate language unit derived from word or sentence stimuli. The upper part of Figure 3 illustrates the different categories of measures that used a speech-based stimuli.

When charting the word and sentence measures in relation to the auditory hierarchy (Table 2A), a high representation of speech *recognition* measures was found (*n* = 1968; 90.4%) in comparison to measures of speech *comprehension* (*n* = 72; 3.3%). Studies that used multiple levels of measurement, such as speech discrimination and speech comprehension, were categorised according to the highest auditory hierarchy level represented by the measures. Speech discrimination was used in 6.1% (*n* = 132) of the studies and only five studies (0.2%) used word or sentence stimuli as a speech detection task.

A few studies reported on linguistic measurement aspects complementary to, or as a related functional characterisation of, speech-based stimuli (*n* = 165/2579; 6.4%). Acceptable noise level judgement (ANLJ) tests that used speech material as the target stimuli were included in this grouping. Table 2B displays the other linguistic measures, found in 165 articles, categorised into their related linguistic domain. Suprasegmental features were assessed most often (35.8%; *n* = 59/165), including both non-tonal (28.5%; *n* = 47/165) and tonal languages (7.3%; *n* = 12/165). Paralinguistic cues (aspects of spoken communication that add emphasis and meaning but are not in words, such as gesture and body language, conversational proximity, mood) were assessed the least (9.7%, *n* = 16/165).

### Cognitive Measures

Measures of cognition were found in 13.3% (*n* = 343) of all included studies (Table 2D). Eighty-seven studies (25.4%) used a cognitive measure that targeted a single cognitive domain. Multi-domain diagnostic cognitive measures were reported most commonly (*n* = 135/343; 39.4%), with screening measures (single measures that assess multiple cognitive domains) used in 33.5% studies (*n* = 115). The most frequently used cognitive screening measure was the Mini-Mental State Examination (MMSE) (Folstein et al., 1975; Lacritz and Hom, 1996). All reported cognitive measures were categorised into the target neurocognitive domains per the DSM-5. According to DSM-5 categorisation, 41.4% of studies (*n* = 142) included a specific measure of executive function (which encompasses planning, decision making, working memory, responding to feedback/error correction, overriding habits/inhibition and mental flexibility). Measures of complex attention (including evaluation of sustained attention, divided attention, selective attention and processing speed) were present in 30.0% (*n* = 103/343) of studies utilising cognitive measures. Measures of language were used in 20.4% of studies (*n* = 71/343) and measures of learning and memory in 14.6% (*n* = 50/343) of studies. Measures of social cognition (such as assessment of emotion and theory of mind) were limited, with 2.0% (*n* = 7/343) of studies reporting measures related to this DSM-5 domain. Six studies, labelled as “unspecified,” did not state the specific cognitive measure used or provided inadequate methodological information, preventing DSM-5 domain allocation during data charting.

### Self-Report Measures

One or more self-report measures were used in 945 of all included studies (36.6%; *n* = 945/2579). Including all previously published self-report measures (study-specific questionnaires, as well as single-question self-report measures), a total of 1306 self-report measures were found across 945 studies. A total of 139 previously published self-report measures, classified as either condition-specific (76.9%; *n* = 107/139) or generic (23.0%; *n* = 32/139), were extracted and subsequently categorised in terms of health status outcomes, based on Barker et al. (2015), Madans and Webster (2015) (Table 2C). These domains included: (1) communication; (2) device benefit; (3) disability; (4) health; (5) physiological; (6) psychological; (7) quality of life, and (8) other. As ambiguity exists in relation to definitions for constructs such as disability and quality of life, a number of self-report measures were found to cover multiple constructs. Detailed discussion relating to this issue is beyond the scope of this review but interested readers can refer to Eyssen et al. (2011), Milton (2013) for more information. For this review, disability was used as an umbrella term to encompass impairments, activity limitations, and participation restrictions as linked constructs (World Health Organization, 2001).

Condition-specific (auditory) disability represented 31.2% (*n* = 408/1306) of self-report measures, followed by measures of device benefit (21.2%; *n* = 277/1306). Measures targetting communication as the primary construct accounted for 1.3% (*n* = 18/1306) of the self-report measures used. Over 70% (*n* = 664/945) of studies used a single self-report measure, 20.0% (*n* = 189/945) used two self-report measures, 7.1% (*n* = 68/945) three self-report measures, 2.0% (*n* = 19/945) four self-report measures, and 0.4% (*n* = 4/945) used four or more self-report measures. Of the formal self-report measures identified across studies, the majority *n* = 84/139 (60.4%) were used in a single study. In total, 80.5% (*n* = 112/139) of formal measures were cited five times or fewer, indicating a lack of consistency in the selection of self-report measures in clinical studies. Measures designed explicitly for a study (i.e., study-specific) were the self-reported measures used in most studies (*n* = 315/945; 33.3%). The most frequently used psychometrically validated measures were the Speech, Spatial and Qualities of hearing (SSQ) scale (Gatehouse and Noble, 2004), the Abbreviated Profile of Hearing Aid Benefit [APHAB; (Cox and Alexander, 1995)] and the Hearing Handicap Inventory for the Elderly [HHIE; (Ventry and Weinstein, 1982)].

### Representation of Assessment Measures Within Individual Studies

**FIGURE 4:**
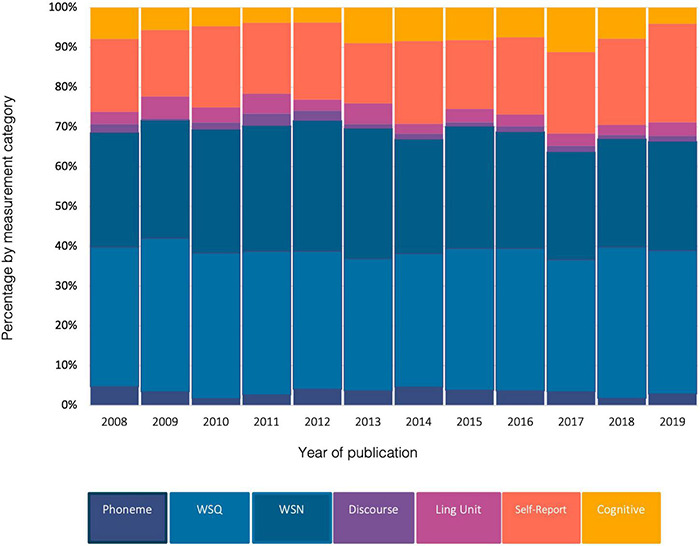
Assessment measure, linguistic categories by year of publication. Total percentages exceed 100% due to multiple measures used within studies.

To assess whether the makeup of communication-relevant measures used in published studies had changed over time, the number of measures, categorised by measurement type, used in studies per year was graphed (Figure 4). While the total number of publications increased over time (Figure 2), the distribution of measures by measure type remained relatively consistent. Word and sentence measures, specifically measures in quiet, were the most frequently used assessment measure each year. When measures were grouped by measurement type, comparison of measures across years demonstrated the relatively narrow range of variability within groupings. There was less than ten percent variation between the lowest and highest percentage of measurement group by type for all categories. The exception was word and sentence measures in noise (WSN) which varied from 47.5% to 66.5%. The limited variation found in the representation of cognitive measurement across years was unexpected. The recent developments in the field of cognitive hearing science, which highlights the intrinsic role of cognition in listening (Arlinger et al., 2009; Lunner et al., 2020), and the publication of studies that showed a relationship between hearing loss and neurocognitive disorders such as dementia (Lin et al., 2011; Livingston et al., 2017), would intuitively have promoted an increase in the use of cognitive measures. The data extracted in this review suggests, however, that there was a proportional increase in the use of all types of measures relevant to listening and communication. Articles published in 2019 had the highest percentage of self-report measures with 42.8% (*n* = 127/297) of included studies using some form of self-report. Discourse measures were the most infrequently used form of measurement (range = 0.8% – 5.6%) across publication years.

## Discussion

This scoping review identified and examined measures used within recently published studies to evaluate listening skills for oral communication in adults with hearing loss. In particular, using a linguistic perspective, the review provides a useful categorisation system to evaluate the capacity for existing measures to represent everyday communication as experienced by adults with hearing loss. Results from this review suggest that measures used to assess listening abilities target a narrow set of domains, limited predominantly to measures of speech detection and recognition at the word or sentence level, and that preferences for outcome measure selection have remained relatively constant for the last decade. Furthermore, despite these measurement preferences, there remains a lack of consensus within published studies regarding the selection of measures that target the complexities of listening and communication. The persistent focus on detection-based measures and the limited use of measures assessing complex/higher-level listening abilities suggests that current measures may not be evaluating those listening constructs of most relevance to adults with hearing loss when they are listening in the communication situations of everyday life.

### Measurement Bias – The Prevalence of Detection Measures

The prevalence of detection-based measures in the included articles points to a focus within outcome studies to undertake measurement at the level of impairment (i.e., hearing) and not at the level of disability or handicap (World Health Organization, 2001). These findings suggest that within published studies, assessment of hearing is conceptualised as an isolable function that is independent or disconnected from its role in listening and communication (Manchaiah et al., 2019). While detection-based measures are valuable for classifying hearing levels, they provide limited information about functional listening ability in communicative contexts. Evidence indicates that detection-based measures do not provide information beyond hearing sensitivity (Engdahl et al., 2013; Fredriksson et al., 2016; Musiek, 2017; Phatak et al., 2019). For example, two listeners with the same audiometric thresholds can have different speech-in-noise performance (Gifford et al., 2007), and many individuals report significant hearing difficulties that are not reflected in hearing threshold measurement (Füllgrabe et al., 2015; Bakay et al., 2018; Barbee et al., 2018; Vermiglio et al., 2018). In contrast, a large study exploring access barriers to hearing intervention in older adults, found that 40% of adults with audiometrically measurable hearing loss did not report a hearing difficulty (Sawyer et al., 2020). The review findings indicate that, despite these well-evidenced shortcomings, measurement at the level of detection continues to be the dominant assessment measure reported in published studies of adults with hearing loss.

### Measurement and Language Units

Beyond detection, measures using words or sentences as stimuli were the measures most frequently identified in this review. This finding is consistent with an earlier systematic review of outcome measures in hearing loss in which word-level speech recognition measures with and without noise comprised the largest measurement group (Granberg et al., 2014). The high representation of word and sentence measures found in this review was expected as words or sentences represent the primary language unit to which contextual, linguistic and cognitive modifications are applied. The high prevalence of word and sentence-based measures has also been reported in a scoping review of outcome measures used to assess adults with cochlear implants (Boisvert et al., 2020).

The high proportion of word or sentence measures identified in this review is problematic, however, because, similar to detection-based measures, limitations also exist when using word and sentence stimuli, particularly in quiet conditions. For example, word and sentence stimuli administered in a quiet environment are prone to ceiling effects, correlate poorly with reports of listening abilities, and have low ecological validity (Firszt et al., 2004; Best et al., 2016b; Musiek, 2017). For example, a study appraising speech perception protocols for cochlear implant users demonstrated that, when tested in quiet, 28% (*n* = 206) of participants achieved the maximum score of 100% (Gifford et al., 2008). While measures of speech perception are expected to correlate with each other, this study also found poor agreement between scores achieved in quiet and those achieved in noise for both monosyllables and sentences. Individual performance in quiet was not predictive of performance when measures used speech-based material in noise. Perhaps more significant from a functional perspective, difficulty listening in noise, not in quiet, is one of the most frequently reported auditory symptoms and a defining feature of adult hearing loss (Arlinger, 2009; Hughes et al., 2018; Pang et al., 2019).

### Language Units and Auditory Context

Attempts to address measurement limitations when using word and sentence in quiet stimuli frequently involves changes to the stimulus complexity (Klatte et al., 2010). Within this scoping review, noise was the most frequent modifier of word or sentence complexity. When viewed in relation to the challenges associated with listening in noise as reported by adults with hearing loss (Arlinger, 2009), the extensive use of words and sentences in noise measures have high face validity. The inclusion of noise in word and sentence-based measures has been found to: (1) contribute to a higher degree of diagnostic accuracy for the challenges of listening in noise (Vermiglio et al., 2018); (2) minimise ceiling effects associated with assessment of word and sentence recognition undertaken in quiet (Gifford and Revit, 2010); and (3) involve the engagement of additional cognitive mechanisms required to interpret degraded auditory input (Hwang et al., 2017). Therefore, changing stimulus complexity through the addition of noise may be a more realistic assessment of hearing and listening ability. There are, however, other considerations that may influence the representativeness of these measures. For instance, despite the preservation of some characteristics, the artificial noise generated as part of clinical testing protocols has little in common with the dynamic and reverberant acoustic environments encountered in everyday life (Weisser and Buchholz, 2019). Behaviours related to communicating in noise, such as speaker volume and physical proximity adaptations, are similarly not accounted for in existing measures. The adaptative behaviours of speakers assist with managing communication in varying noise levels and, therefore, may affect an individual’s varying capacity for communication in these environments (Beechey et al., 2019). The preference for the addition of noise to create representative measurement in the included studies suggests a reductive approach to measurement that does not account for the impact and importance of cognitive and higher-level linguistic factors on interpersonal communication.

### Measurement Units and Communication

Current outcome measures use language unit boundaries (phoneme, word and sentence) to create discrete independent measurement units. Attempting to represent communication *via* these unit boundaries implies that these independent units are present and measurable in continuous speech streams. However, natural speech and language is not easily divisible into distinct, and seamlessly recognisable components (Walsh, 2011). The imperfections, deletions and ill-defined boundaries that are present in spontaneous communication, provide rich information used to contextualise and clarify spoken communication between communication partners (Podlubny et al., 2018). Dysfluencies, prosodic shifts and fillers support natural conversation, acting as recognisable markers in speech to signify the need for repetition or request for clarification between speakers (Corley and Stewart, 2008). These features are supportive communicative tactics, but current unit-based (phoneme, word and sentence) measures, either do not represent these features, or classify them as inaccurate responses that are scored accordingly, contrary to their supportive communicative function. From this perspective, reductive unit measures, such as phonemes, words and sentences, lack the dynamic and multimodal elements that define interactive communication as experienced by a listener in the real world.

### Auditory Hierarchy and Comprehension

Charting word and sentence measures in relation to the auditory hierarchy demonstrated the disproportionate representation of measures considered to assess speech recognition. Classification within the auditory hierarchy is valuable when considering the capacity of speech perception measures to characterise listening and communication ability. As a measure of auditory ability, speech recognition measures, which are typically based on clients repeating the individual items they hear, require limited linguistic prosessing and do not represent comprehension of the presented stimuli. Auditory comprehension, extracting meaning from auditory input, is crucial for oral communication competence. Extracting meaning from words and sentences changes the speech paradigm to engage a variety of linguistic (e.g., lexical, syntactic and phonological) and cognitive (e.g., working memory, attention, processing speed) mechanisms (Macdonald, 2017). The changes in load and task associated with comprehension enable more direct measurement of higher-level speech processes that are central to functional communication. Comprehension measures in this review sought to clarify these mechanisms relative to hearing loss and included, for example: processing structurally complex sentences and degraded speech (Carroll et al., 2016); neural activation in speech understanding (Zhou et al., 2018); suitability of dynamic speech materials to capture features specific to conversation (Best et al., 2016a); and the influence of syntactic form on plausibility (Amichetti et al., 2016). Interestingly, studies comparing measures of recognition and comprehension suggest that existing comprehension paradigms in the assessment of listening may be inadequate (Best et al., 2018).

The complex and continuous process of auditory comprehension in the listening situations of daily life is reliant on mechanisms that enable accurate interpretation of dynamic inferential and contextual information (Doedens and Meteyard, 2019), as well as socio-cognitive contributions such as theory of mind and self-regulation (Worthington, 2018). Without representation of these dynamic and dependent elements, measures of auditory comprehension may have a reduced capacity to represent real world communication ability. Similarly, and as suggested by the findings of this scoping review, the continued preference for studies to utilise measures of speech recognition, maintains a focus on reductive instruments that are unable to measure the complex processes of auditory comprehension and its contribution to day-to-day communication.

### Cognitive Assessment Measures

The operationalisation of listening and communication is dependent on cognition (Wolvin and Wiley, 2010). Cognitive mechanisms are required to attend to, make sense of, and remember auditory information – the prerequisite functions of listening and communication (Rost, 2016). Measures of cognition are therefore relevant to understanding the processing and individual expression of listening and communication (Lunner et al., 2009). For example, a recent study using a hearing-impairment simulation demonstrated that hearing loss does indeed impact cognitive-test performance, and this is not only due to reduced audibility (Füllgrabe, 2020). The studies identified in this review used measures of cognition for a variety of purposes: (1) to understand relationships between cognitive domains and listening (Amichetti et al., 2013; Ferguson and Henshaw, 2015; Keidser et al., 2016); (2) to account for variance in listening ability that is not identified within standard audiological measures (Kronenberger et al., 2014; Kaandorp et al., 2017; Moberly et al., 2018b); (3) as an indicator of neurocognitive function (Gates et al., 2008; Wong et al., 2014; Dawes et al., 2015); and (4) to determine if targetting cognition assists with rehabilitation (Pichora-Fuller and Levitt, 2012; Castiglione et al., 2016; Nkyekyer et al., 2018). As with language and communication, measurement of cognition as a separate and discrete function is complex. The included studies have addressed this complexity with multi-domain diagnostic assessments aimed at clarifying how cognitive ability is impacted by hearing loss (Mosnier et al., 2015; Claes et al., 2018). While studies that included domain-specific and multi-domain cognitive assessments are driving our understanding of cognition in relation to listening, language, and hearing loss (Rönnberg et al., 2019), they are underrepresented in this review. A significant proportion of studies exploring cognition used screening measures, which have noted limitations as the primary form of assessment. Raymond et al. (2020) systematic review of cognitive screening with adults with post-lingual hearing loss confirmed the frequent use of screening assessments such as the MMSE and the Montreal Cognitive Assessment (MoCA), both of which are reliant on auditory components. The authors note that based on the available evidence, these auditory components may have a deleterious effect on scores for adults with hearing loss. Therefore, poor performance may be an indication of poor cognition, poor audibility for instruction, or increased effort for listening, which is known to impact working memory and recall (Wayne et al., 2016). Adaptations to screening measures to adjust for auditory components have also proven problematic, as the removal and modification of items can directly influence the pass/fail status (sensitivity) (Parada et al., 2020) and these modifications may not yet have been formally validated (Dawes et al., 2019; Raymond et al., 2020).

Classification of the extracted outcome measures into linguistic categories indicated that standard measures used to assess hearing and listening were not designed to assess basic information-processing operations of listening and communication. In regard to functional communication, product or output measures, such as speech perception, may misrepresent the experience of listening with hearing loss by ignoring the cognitive involvement required in the task (Moberly et al., 2018a). Consequently, currently available outcome measures do not capture the functional variability that is evidenced in adults with hearing loss. The measures most frequently used in the included studies did not appear to capture the cognitive involvement required to attend to and process speech information or the effort intrinsic in communication adaptation and compensation (Hughes et al., 2017; Peelle, 2018). These measures also do not reflect what listening and communication mean for the individual driven by the motivation and need for social connectedness (Hughes et al., 2018).

### Self-Report Measures

The self-report measures included in this review were described in relation to health status outcomes and the number of times each measure was cited in clinical studies. Consistent with previous reviews of self-report in hearing loss, a large number of self-report measures were identified (Granberg et al., 2014; Akeroyd et al., 2015). Outcomes from these reviews showed that the majority of measures were not used repeatedly in clinical studies (Granberg et al., 2014; Akeroyd et al., 2015; Barker et al., 2015). The complications of many different self-report measures used infrequently across studies are compounded by the large number of studies that used a bespoke, or study-specific, self-report measure. This lack of consistency has the potential to constrain cross-study comparison and prevent data aggregation, limiting the use of data beyond an individual study. The pervasive impact of hearing loss may account for the diversity of targeted health status domains in self-reports. This diverse representation (e.g., disability, device benefit, QoL) may provide some explanation as to why so many self-report measures have been developed (Vas et al., 2017). Similarly, it may also reflect the inability of current measures to address the targeted health domains effectively (Barker et al., 2017). The volume and prevalence of self-report measures, however, suggests that criteria for selecting an appropriate measure is not evident, and currently no single standard measure is widely adopted in clinical studies (Akeroyd et al., 2015).

### Study Limitations

There were a number of limitations associated with this review. Despite the use of a comprehensive search strategy, it is possible that some studies were not included due to abstracts not indicating the use of linguistic-based measurement. Studies published in languages other than English that did not have an accompanying translation were not considered. As such, a language bias is present in this review. Excluding studies with sample sizes smaller than ten subjects potentially limited the extraction of all relevant measures. In addition to language, potential country specific bias may also reflect the legislative and policy contexts that mandate the inclusion of particular measures for use in the included studies. The high number of studies and broad country representation helped to address these biases. From a semantic and cognitive perspective, the terminology used to define measure types was indistinctive. Without clarification into levels within the auditory hierarchy, categorisation based on the level of speech processing assessed by the measure was not possible. Using linguistic categorisation to chart the extracted outcome measures presented limitations related to exploring language and communication from a compartmentalised perspective (Walsh, 2011). Given that functional communication encompasses components across multiple domains of the communication complex, utilisation of a theoretical framework may lead to reductionist conceptualisations of communication with hearing loss. Recognising this limitation of current conceptualisations of functional communication may help us understand why current measurement limitations exist. Finally, the allocation of self-report measures according to health domains may not accurately reflect the intended content of a measure’s items. Manchaiah et al. (2019) study on content validity and readability in self-report measures of hearing disability demonstrated substantial variability in domain measurement. For example, measures were described as measures of disability; however, item analysis indicated the targetting of a number of additional constructs. The findings of this review, supported by Manchaiah et al. (2019) study lend support to the assertation that, without a rigorous evaluation of a measure’s content validity, it may not be possible to understand fully the conceptual coverage provided by a measure’s items (Terwee et al., 2018).

### Implications for Clinical Practice and Future Research Directions

This review outlines limitations in measures of listening and communication when these are viewed from a functional perspective. Findings from this review provide a reference to describe how outcome measures relate to the components of functional listening in daily life. This information could be used in clinical practice and research to provide a more nuanced evaluation of the listening abilities of adults with hearing loss. The reductive approach to measurement described in this review may account for the contrast between what is measured and the priorities and perspectives of adults living with hearing loss (Sawyer et al., 2020). The review findings may also assist in addressing the possible disconnect between people’s understanding of hearing loss and its relationship to communication. While this work provides insight into the potential domains that may be relevant for measurement of functional communication, additional investigation is required to match these theoretical foundations to the communication experiences of adults with hearing loss. For example, qualitative approaches, when applied to understanding functional communication from the perspective of deaf and hard-of-hearing adults, may identify missing links within the listening and communication complex or provide insight into the weighting of different domains and items within that complex. Consultation with stakeholder groups, including adults with hearing loss and clinicians, to corroborate and extend the review findings could provide valuable insights on their usefulness leading to recommendations for policy and practice. This information could then inform the development and selection of outcome measures that better align to the lived experience of adults with hearing loss. Future work is required to evaluate the psychometric properties (the validity and reliability) of new and existing outcome measures in line with the target construct to be measured and the proposed context of use. Further work must also consider the costs (e.g., time, equipment and training required) in comparison to the benefits of selecting and implementing specific outcome measures within clinical or research contexts.

## Conclusion

Real-life communication is quick, responsive, dynamic, continuous and unpredictable. To be an effective communicator we need not only language, but the ability to incorporate and understand language in the context of others and the complexity that they bring with them. Listening is the foundation of oral communication, but there is currently no consensus on how to best represent and measure the complexities of everyday listening for communication in audiological clinical practice. By categorising the included outcome measures in terms of the complexity of the stimuli used, the participant’s response required for the task, as well as the domains targeted within self-reports and cognitive measures that are relevant for listening, this scoping review highlighted both the reductive approach to measurement and the large and heterogenous pool of assessments available to measure functional listening in adults with hearing loss. Without consideration of the broader linguistic, cognitive and interactive elements of communication, measures cannot adequately capture the complex way adults with hearing loss experience listening for communication. To effectively represent functional listening, it will be necessary to expand how audiological measurement is conceptualised and undertaken to ensure functional listening for communication is measured in the context in which it is experienced and from the perspective of those who experience it.

## Author Contributions

KN conceived and led the study as part of her masters of research thesis supervised by IB and CM and charted the data and prepared the drafts of the manuscript, tables, and figures with critical guidance from IB, CM, and SH. KN and IB conducted the systematic searches. All authors provided substantial input into the final draft submitted.

## Conflict of Interest

SH receives funding from the National Institute for Health Research (NIHR) Applied Research Collaboration (ARC) West Midlands, UK Research and Innovation (UKRI) and declares personal fees from Aparito Limited and Cochlear Limited outside the submitted work. The funder was not involved in the study design, collection, analysis, interpretation of data, the writing of this article or the decision to submit it for publication. The remaining authors declare that the research was conducted in the absence of any commercial or financial relationships that could be construed as a potential conflict of interest.

## Publisher’s Note

All claims expressed in this article are solely those of the authors and do not necessarily represent those of their affiliated organizations, or those of the publisher, the editors and the reviewers. Any product that may be evaluated in this article, or claim that may be made by its manufacturer, is not guaranteed or endorsed by the publisher.

## Footnotes

1
http://www.covidence.org

2
https://public.tableau.com/

## References

[B1] Akeroyd M. A. Wright-Whyte K. Holman J. A. Whitmer W. M. (2015). A comprehensive survey of hearing questionnaires: how many are there, what do they measure, and how have they been validated? *Trials* 16:26. 10.1186/1745-6215-16-S1-P26

[B2] Ambert-Dahan E. Laouenan C. Lebredonchel M. Borel S. Carillo C. Bouccara D. (2018). Evaluation of the impact of hearing loss in adults: validation of a quality of life questionnaire. *Eur. Annals Otorhinolaryngology-Head Neck Dis.* 135 25–31. 10.1016/j.anorl.2017.09.003 29274768

[B3] American Psychiatric Association (2013). *Diagnostic and Statistical Manual of Mental Disorders: DSM-5.* Washington, D.C.: American Psychiatric Association.

[B4] Amichetti N. M. White A. G. Wingfield A. (2016). Multiple solutions to the same problem: utilization of plausibility and syntax in sentence comprehension by older adults with impaired hearing. *Front. Psychol.* 7:789. 10.3389/fpsyg.2016.00789 27303346 PMC4884746

[B5] Amichetti N. Stanley R. White A. Wingfield A. (2013). Monitoring the capacity of working memory: executive control and effects of listening effort. *Memory Cogn.* 41 839–849. 10.3758/s13421-013-0302-300 PMC3718871 23400826

[B6] Anderson S. Kraus N. (2010). Sensory-Cognitive interaction in the neural encoding of speech in noise: a review. *J. Am. Acad. Audiol.* 21 575–585. 10.3766/jaaa.21.9.3 21241645 PMC3075209

[B7] Arlinger S. (2009). Negative consequences of uncorrected hearing loss—a review. *Int. J. Audiol.* 42 17–20. 10.3109/14992020309074639 12918624

[B8] Arlinger S. Lunner T. Lyxell B. P.-F. Katherine. (2009). The emergence of cognitive hearing science. *Scand. J. Psychol.* 50 371–384. 10.1111/j.1467-9450.2009.00753.x 19778385

[B9] Bakay W. M. H. Anderson L. A. Garcia-Lazaro J. A. McAlpine D. Schaette R. (2018). Hidden hearing loss selectively impairs neural adaptation to loud sound environments. *Nat. Commun.* 9:4298. 10.1038/s41467-018-06777-y 30327471 PMC6191434

[B10] Barbee C. James J. Park J. Smith E. Johnson C. Clifton S. (2018). Effectiveness of auditory measures for detecting hidden hearing loss and/or cochlear synaptopathy: a systematic review. *Semin. Hear.* 39 172–209. 10.1055/s-0038-1641743 29915454 PMC6003814

[B11] Barker A. B. Leighton P. Ferguson M. A. (2017). Coping together with hearing loss: a qualitative meta-synthesis of the psychosocial experiences of people with hearing loss and their communication partners. *Int. J. Audiol.* 56 297–305. 10.1080/14992027.2017.1286695 28599604

[B12] Barker F. Mackenzie E. Elliott L. De Lusignan S. (2015). Outcome measurement in adult auditory rehabilitation: a scoping review of measures used in randomized controlled trials. *Ear Hear.* 36 567–573. 10.1097/AUD.0000000000000167 25919402

[B13] Baskent D. Clarke J. Pals C. Benard M. R. Bhargava P. Saija J. (2016). Cognitive compensation of speech perception with hearing impairment, cochlear implants, and aging: how and to what degree can it be achieved? *Trends Hear.* 20:2331216516670279.

[B14] Beechey T. Buchholz J. M. Keidser G. (2019). Eliciting naturalistic conversations: a method for assessing communication ability, subjective experience, and the impacts of noise and hearing impairment. *J. Speech Lang. Hear. Res.* 62 470–484. 10.1044/2018_jslhr-h-18-0107 30950689

[B15] Best V. Keidser G. Buchholz J. M. Freeston K. (2016a). Development and preliminary evaluation of a new test of ongoing speech comprehension. *Int. J. Audiol.* 55 45–52. 10.3109/14992027.2015.1055835 26158403 PMC4762876

[B16] Best V. Keidser G. Freeston K. Buchholz J. M. (2016b). A dynamic speech comprehension test for assessing real-world listening ability. *J. Am. Acad. Audiol.* 27 515–526. 10.3766/jaaa.15089 27406659

[B17] Best V. Keidser G. Freeston K. Buchholz J. M. (2018). Evaluation of the NAL dynamic conversations test in older listeners with hearing loss. *Int. J. Audiol.* 57 221–229. 10.1080/14992027.2017.1365275 28826285 PMC6114171

[B18] Boisvert I. Clemesha J. Lundmark E. Crome E. Barr C. McMahon C. M. (2017). Decision-Making in audiology: balancing evidence-based practice and patient-centered care. *Trends Hearing* 21:2331216517706397. 10.1177/2331216517706397 28752808 PMC5536381

[B19] Boisvert I. Reis M. Au A. Cowan R. Dowell R. C. (2020). Cochlear implantation outcomes in adults: a scoping review. *PLoS One* 15:e0232421. 10.1371/journal.pone.0232421 32369519 PMC7199932

[B20] Bramer W. M. Rethlefsen M. L. Kleijnen J. Franco O. H. (2017). Optimal database combinations for literature searches in systematic reviews: a prospective exploratory study. *Systematic Rev.* 6:78. 10.1186/s13643-017-0644-y 29208034 PMC5718002

[B21] Carroll R. Uslar V. Brand T. Ruigendijk E. (2016). Processing mechanisms in hearing-impaired listeners: evidence from reaction times and sentence interpretation. *Ear Hear.* 37 e391–e401. 10.1097/AUD.0000000000000339 27748664

[B22] Castiglione A. Benatti A. Velardita C. Favaro D. Padoan E. Severi D. (2016). Aging, cognitive decline and hearing loss: effects of auditory rehabilitation and training with hearing aids and cochlear implants on cognitive function and depression among older adults. *Audiol. Neurotol.* 21 21–28. 10.1159/000448350 27806352

[B23] Claes A. J. Van de Heyning P. Gilles A. Den Brandt A. H.-V. Van Rompaey V. Mertens G. (2018). Impaired cognitive functioning in cochlear implant recipients over the age of 55 years: a cross-sectional study using the repeatable battery for the assessment of neuropsychological status for hearing-impaired individuals (RBANS-H). *Front. Neurosci.* 12:580. 10.3389/fnins.2018.00580 30197584 PMC6117382

[B24] Corley M. Stewart O. W. (2008). Hesitation disfluencies in spontaneous speech: the meaning of um. *Lang. linguistics Compass* 2 589–602. 10.1111/j.1749-818X.2008.00068.x

[B25] Cox R. M. Alexander G. C. (1995). The abbreviated profile of hearing aid benefit. *Ear Hear.* 16 176–186. 10.1097/00003446-199504000-199504005 7789669

[B26] Cox R. Hyde M. Gatehouse S. Noble W. Dillon H. Bentler R. (2000). Optimal outcome measures, research priorities, and international cooperation. *Ear. Hear.* 21(4 Suppl.), 106S–115S. 10.1097/00003446-200008001-00014 10981601

[B27] Dawes P. Cruickshanks K. J. Fischer M. E. Klein B. E. Klein R. Nondahl D. M. (2015). Hearing-aid use and long-term health outcomes: hearing handicap, mental health, social engagement, cognitive function, physical health, and mortality. *Int. J. Audiol.* 54 838–844. 10.3109/14992027.2015.1059503 26140300 PMC4730911

[B28] Dawes P. Pye A. Reeves D. Yeung W. K. Sheikh S. Thodi C. (2019). Protocol for the development of versions of the Montreal Cognitive Assessment (MoCA) for people with hearing or vision impairment. *BMJ Open* 9:e026246. 10.1136/bmjopen-2018-026246 30928949 PMC6475249

[B29] Deloitte Access Economics (2017). *The Social and Economic Cost of Hearing Loss in Australia.* Sydney NSW: Hearing Care Industry Association.

[B30] Doedens W. J. Meteyard L. (2019). The importance of situated language use for aphasia rehabilitation. *PsyArXiv* [Preprint]. 10.31234/osf.io/svwpf

[B31] Engdahl B. Tambs K. Hoffman H. J. (2013). Otoacoustic emissions, pure-tone audiometry, and self-reported hearing. *Int. J. Audiol.* 52 74–82. 10.3109/14992027.2012.733423 23216196

[B32] Erber N. P. (1982). *Auditory Training / Norman P. Erber.* Washington, D.C: Alexander Graham Bell Association for the Deaf.

[B33] Estabrooks W. Morrison H. M. MacIver-Lux K. (2020). *Auditory-verbal Therapy: Science, Research, and Practice.* San Diego, CA: Plural Publishing, Inc.

[B34] Eyssen I. C. Steultjens M. P. Dekker J. Terwee C. B. (2011). A systematic review of instruments assessing participation: challenges in defining participation. *Arch. Phys. Med. Rehabil.* 92 983–997. 10.1016/j.apmr.2011.01.006 21621675

[B35] Ferguson M. A. Henshaw H. (2015). Auditory training can improve working memory, attention, and communication in adverse conditions for adults with hearing loss. *Front. Psychol.* 6:556. 10.3389/fpg.2015.00556 PMC4447061 26074826

[B36] Firszt B. J. Holden K. L. Skinner W. M. Tobey A. E. Peterson L. A. Gaggl A. W. (2004). Recognition of speech presented at soft to loud levels by adult cochlear implant recipients of three cochlear implant systems. *Ear Hear.* 25 375–387. 10.1097/01.aud.0000134552.22205.ee 15292777

[B37] Folstein M. F. Folstein S. E. McHugh P. R. (1975). “Mini-mental state”: a practical method for grading the cognitive state of patients for the clinician. *J. Psychiatr. Res.* 12 189–198. 10.1016/0022-3956(75)90026-90026 1202204

[B38] Fredriksson S. Hammar O. Magnusson L. Kahari K. Waye K. P. (2016). Validating self-reporting of hearing-related symptoms against pure-tone audiometry, otoacoustic emission, and speech audiometry. *Int. J. Audiol.* 55 454–462. 10.1080/14992027.2016.1177210 27195802

[B39] Füllgrabe C. (2020). On the possible overestimation of cognitive decline: the impact of age-related hearing loss on cognitive-test performance. *Front. Neurosci.* 14:454. 10.3389/fnins.2020.00454 32581666 PMC7296091

[B40] Füllgrabe C. Moore B. C. J. Stone M. A. (2015). Age-group differences in speech identification despite matched audiometrically normal hearing: contributions from auditory temporal processing and cognition. *Front. Aging Neurosci.* 6:347. 10.3389/fnagi.2014.00347 25628563 PMC4292733

[B41] Gatehouse S. Noble W. (2004). The speech, spatial and qualities of hearing scale (SSQ). *Int. J. Audiol.* 43 85–99. 10.1080/14992020400050014 15035561 PMC5593096

[B42] Gates G. A. Anderson M. L. Feeney M. P. McCurry S. M. Larson E. B. (2008). Central auditory dysfunction in older persons with memory impairment or Alzheimer dementia. *Arch. Otolaryngology-Head Neck Surgery* 134 771–777. 10.1001/archotol.134.7.771 18645130 PMC2871110

[B43] Gifford R. H. Bacon S. P. Williams E. J. (2007). An examination of speech recognition in a modulated background and of forward masking in younger and older listeners. *J. Speech Lang. Hear. Res.* 50:857. 10.1044/1092-4388(2007/060) PMC2441836 17675591

[B44] Gifford R. H. Shallop J. K. Peterson A. M. (2008). Speech recognition materials and ceiling effects: considerations for cochlear implant programs. *Audiol. Neurootol.* 13 193–205. 10.1159/000113510 18212519

[B45] Gifford R. Revit L. J. (2010). Speech perception for adult cochlear implant recipients in a realistic background noise: effectiveness of preprocessing strategies and external options for improving speech recognition in noise. *J. Am. Acad. Audiol.* 21 441–451 quiz 487–448. 10.3766/jaaa.21.7.3. 20807480 PMC4127078

[B46] Granberg S. Dahlström J. Möller C. Kähäri K. Danermark B. Linköpings U. (2014). The ICF core sets for hearing loss - researcher perspective. Part I: systematic review of outcome measures identified in audiological research. *Int. J. Audiol.* 53 65–76. 10.3109/14992027.2013.851799 24313738

[B47] Guediche S. Blumstein S. E. Fiez J. A. Holt L. L. (2014). Speech perception under adverse conditions: insights from behavioral, computational, and neuroscience research. *Front. Systems Neurosci.* 7:126. 10.3389/fnsys.2013.00126 24427119 PMC3879477

[B48] Heinrich A. Gagne J. P. Viljanen A. Levy D. Ben-David B. M. Schneider B. A. (2016). Effective communication as a fundamental aspect of active aging and well-being: paying attention to the challenges older adults face in noisy environments. *Soc. Inquiry Well-Being* 2 51–69. 10.13165/SIIW-16-2-1-05

[B49] Hughes E. S. Hutchings A. H. Rapport L. F. McMahon M. C. Boisvert M. I. (2018). Social connectedness and perceived listening effort in adult cochlear implant users: a grounded theory to establish content validity for a new patient-reported outcome measure. *Ear Hear.* 39 922–934. 10.1097/AUD.0000000000000553 29424766

[B50] Hughes S. E. Rapport F. L. Boisvert I. McMahon C. M. Hutchings H. A. (2017). Patient-reported outcome measures (PROMs) for assessing perceived listening effort in hearing loss: protocol for a systematic review. *BMJ Open* 7:e014995. 10.1136/bmjopen-2016-014995 28592576 PMC5734199

[B51] Hwang J. S. Kim K. H. Lee J. H. (2017). Factors affecting sentence-in-noise recognition for normal hearing listeners and listeners with hearing loss. *J. Audiol. Otol.* 21 81–87. 10.7874/jao.2017.21.2.81 28704894 PMC5516699

[B52] Kaandorp M. W. Smits C. Merkus P. Festen J. M. Goverts S. T. (2017). Lexical-Access ability and cognitive predictors of speech recognition in noise in adult cochlear implant users. *Trends Hear.* 21:2331216517743887. 10.1177/2331216517743887 29205095 PMC5721962

[B53] Keidser G. Rudner M. Seeto M. Hygge S. Ronnberg J. (2016). The effect of functional hearing and hearing aid usage on verbal reasoning in a large community-dwelling population. *Ear Hear.* 37 e26–e36. 10.1097/AUD.0000000000000196 26244401

[B54] Keidser G. Seeto M. Rudner M. Hygge S. Ronnberg J. (2015). On the relationship between functional hearing and depression. *Int. J. Audiol.* 54 653–664. 10.3109/14992027.2015.1046503 26070470

[B55] Klatte M. Lachmann T. Meis M. (2010). Effects of noise and reverberation on speech perception and listening comprehension of children and adults in a classroom-like setting. *Noise Health* 12 270–282. 10.4103/1463-1741.70506 20871182

[B56] Kronenberger W. G. Colson B. G. Henning S. C. Pisoni D. B. (2014). Executive functioning and speech-language skills following long-term use of cochlear implants. *J. Deaf Stud. Deaf Educ.* 19 456–470. 10.1093/deafed/enu011 24903605 PMC4146384

[B57] Kuhl P. Rivera-Gaxiola M. (2008). Neural substrates of language acquisition. *Annu. Rev. Neurosci.* 31 511–534. 10.1146/annurev.neuro.30.051606.094321 18558865

[B58] Lacritz L. H. Hom J. (1996). Mini-mental state examination (MMSE) severity ratings and neuropsychological functioning. *Arch. Clin. Neuropsychol.* 11:414. 10.1016/0887-6177(96)83934-83935

[B59] Lin F. R. (2020). Making sense of the senses in aging research. *J. Gerontol. A Biol. Sci. Med. Sci.* 75 529–530. 10.1093/gerona/glaa028 32060540

[B60] Lin F. R. Ferrucci L. Metter E. J. An Y. Zonderman A. B. Resnick S. M. (2011). Hearing loss and cognition in the baltimore longitudinal study of aging. *Neuropsychology* 25 763–770. 10.1037/a0024238 21728425 PMC3193888

[B61] Livingston G. Sommerlad A. Orgeta V. Costafreda S. G. Huntley J. Ames D. (2017). Dementia prevention, intervention, and care. *Lancet* 13 413–446. 10.1016/S0140-6736(17)31363-6 28735855

[B62] Lunner T. Alickovic E. Graversen C. Ng E. H. N. Wendt D. Keidser G. (2020). Three new outcome measures that tap into cognitive processes required for real-life communication. *Ear Hear.* 41 39S–47S. 10.1097/AUD.0000000000000941 33105258 PMC7676869

[B63] Lunner T. Rudner M. Rönnberg J. (2009). Cognition and hearing aids. *Scand. J. Psychol.* 50 395–403. 10.1111/j.1467-9450.2009.00742.x 19778387

[B64] Macdonald S. (2017). Introducing the model of cognitive-communication competence: a model to guide evidence-based communication interventions after brain injury. *Brain Injury* 31 1760–1780. 10.1080/02699052.2017.1379613 29064304

[B65] Madans J. H. Webster K. M. (2015). “Health surveys,” in *International Encyclopedia of the Social & Behavioral Sciences*, ed. Wright J. D. (Oxford: Elsevier), 725–730.

[B66] Manchaiah V. (2017). Role of self-reported hearing disability and measured hearing sensitivity in understanding participation restrictions and health-related quality of life: a study with hundred and three older adults with hearing loss. *Clin. Otolaryngol.* 42 924–926. 10.1111/coa.12758 27684383

[B67] Manchaiah V. Granberg S. Grover V. Saunders G. H. Hall D. A. (2019). Content validity and readability of patient-reported questionnaire instruments of hearing disability. *Int. J. Audiol.* 58 565–575. 10.1080/14992027.2019.1602738 31017493

[B68] McHugh M. L. (2012). Interrater reliability: the kappa statistic. *Biochem. Med.* 22 276–282. 10.11613/bm.2012.031 PMC3900052 23092060

[B69] Milton C. L. (2013). The ethics of defining quality of life. *Nursing Sci. Quarterly* 26 121–123. 10.1177/0894318413477153 23575485

[B70] Moberly A. Reed J. (2019). Making sense of sentences: top-down processing of speech by adult cochlear implant users. *J. Speech Lang. Hear. Res.* 62 2895–2905. 10.1044/2019_JSLHR-H-18-0472 31330118 PMC6802905

[B71] Moberly A. C. Castellanos I. Mattingly J. K. (2018b). Neurocognitive factors contributing to cochlear implant candidacy. *Otol. Neurotol.* 39 E1010–E1018. 10.1097/mao.0000000000002052 30444846 PMC7037805

[B72] Moberly A. Castellanos J. I. Vasil F. K. Adunka B. O. Pisoni B. D. (2018a). Product” Versus “Process” measures in assessing speech recognition outcomes in adults with cochlear implants. *Otol. Neurotol.* 39 e195–e202. 10.1097/MAO.0000000000001694 29342056 PMC5807136

[B73] Moher D. Liberati A. Tetzlaff J. Altman D. G. The PRISMA Group (2009). Preferred reporting items for systematic reviews and meta-analyses: the PRISMA statement. *PLoS Med.* 6:e1000097. 10.1371/journal.pmed.1000097 19621072 PMC2707599

[B74] Mosnier I. Bebear J. P. Marx M. Fraysse B. Truy E. Lina-Granade G. (2015). Improvement of cognitive function after cochlear implantation in elderly patients. *JAMA Otolaryngology– Head Neck Surg.* 141 442–450. 10.1001/jamaoto.2015.129 25763680

[B75] Musiek F. E. (2017). Perspectives on the pure-tone audiogram. *J. Am. Acad. Audiol.* 28 655–671. 10.3766/jaaa.16061 28722648

[B76] Nittrouer S. (2002). From ear to cortex: a perspective on what clinicians need to understand about speech perception and language processing. *Lang. Speech Hear. Serv. Sch.* 33 237–252. 10.1044/0161-1461(2002/020) 27764498

[B77] Nkyekyer J. Meyer D. Blamey P. J. Pipingas A. Bhar S. (2018). Investigating the impact of hearing aid use and auditory training on cognition, depressive symptoms, and social interaction in adults with hearing loss: protocol for a crossover trial. *JMIR Res. Protocols* 7:e85. 10.2196/resprot.8936 29572201 PMC5889491

[B78] Palmer A. D. Carder P. C. White D. L. Saunders G. Woo H. Graville D. J. (2019). The impact of communication impairments on the social relationships of older adults: pathways to psychological well-being. *J. Speech Lang. Hear. Res.: JSLHR* 62 1–21. 10.1044/2018_JSLHR-S-17-0495 30950760

[B79] Pang J. Beach E. F. Gilliver M. Yeend I. (2019). Adults who report difficulty hearing speech in noise: an exploration of experiences, impacts and coping strategies. *Int. J. Audiol.* 58 851–860. 10.1080/14992027.2019.1670363 31560221

[B80] Parada J. C. Hillyer J. Parbery-Clark A. (2020). Performance on the standard and hearing-impaired montreal cognitive assessment in cochlear implant users. *Int. J. Geriatric Psychiatry* 35 338–347. 10.1002/gps.5267 31989675

[B81] Peelle E. J. (2018). Listening effort: how the cognitive consequences of acoustic challenge are reflected in brain and behavior. *Ear Hear.* 39 204–214. 10.1097/AUD.0000000000000494 28938250 PMC5821557

[B82] Phatak A. S. Brungart S. D. Zion J. D. Grant W. K. (2019). Clinical assessment of functional hearing deficits: speech-in-noise performance. *Ear Hear.* 40 426–436. 10.1097/AUD.0000000000000635 30134353

[B83] Pichora-Fuller M. K. (2003). Cognitive aging and auditory information processing. *Int. J. Audiol.* 42 (Suppl. 2) S26–S22. 12918626

[B84] Pichora-Fuller M. Levitt H. (2012). Speech comprehension training and auditory and cognitive processing in older adults. *Am. J. Audiol.* 21 351–357. 10.1044/1059-0889(2012/12-0025) 23233521

[B85] Podlubny R. G. Nearey T. M. Kondrak G. Tucker B. V. (2018). Assessing the importance of several acoustic properties to the perception of spontaneous speech. *J. Acoust. Soc. Am.* 143 2255–2268. 10.1121/1.5031123 29716257

[B86] Raymond M. Barrett D. Lee D. J. Peterson S. Raol N. Vivas E. X. (2020). Cognitive screening of adults with postlingual hearing loss: a systematic review. *Otolaryngology–Head Neck Surg.* 164 49–56. 10.1177/0194599820933255 32689874

[B87] Rivera S. C. Kyte D. G. Aiyegbusi O. L. Slade A. L. McMullan C. Calvert M. J. (2019). The impact of patient-reported outcome (PRO) data from clinical trials: a systematic review and critical analysis. *Health Qual. Life Outcomes* 17:156. 10.1186/s12955-019-1220-z 31619266 PMC6796482

[B88] Rodd J. M. Johnsrude I. S. Davis M. H. (2012). Dissociating frontotemporal contributions to semantic ambiguity resolution in spoken sentences. *Cereb. Cortex* 22 1761–1773. 10.1093/cercor/bhr252 21968566

[B89] Rönnberg J. Holmer E. Rudner M. (2019). Cognitive hearing science and ease of language understanding. *Int. J. Audiol.* 58 247–261. 10.1080/14992027.2018.1551631 30714435

[B90] Rönnberg J. Lunner T. Zekveld A. Sörqvist P. Danielsson H. Lyxell B. (2013). The Ease of Language Understanding (ELU) model: theoretical, empirical, and clinical advances. *Front. Syst. Neurosci.* 7:31. 10.3389/fnsys.2013.00031 23874273 PMC3710434

[B91] Rönnerg J. Lunner T. Ng E. H. Lidestam B. Zekveld A. A. Sorqvist P. (2016). Hearing impairment, cognition and speech understanding: exploratory factor analyses of a comprehensive test battery for a group of hearing aid users, the n200 study. *Int. J. Audiol.* 55 623–642. 10.1080/14992027.2016.1219775 27589015 PMC5044772

[B92] Rost M. (2016). *Teaching and Researching Listening / Michael Rost.* New York, NY: Routledge.

[B93] Rudner M. (2016). Cognitive spare capacity as an index of listening effort. *Ear Hear.* 37 (Suppl. 1) 69S–76S. 10.1097/AUD.0000000000000302 27355773

[B94] Sawyer C. S. Armitage C. J. Munro K. J. Singh G. Dawes P. D. (2020). Biopsychosocial classification of hearing health seeking in adults aged over 50 years in England. *Ear Hear.* 41 1215–1225. 10.1097/AUD.0000000000000839 31985532 PMC7676482

[B95] Shadden B. B. (1988). Communication behavior and aging: a sourcebook for clinicians. *Ear Hear.* 9:228. 10.1097/00003446-198808000-198808044

[B96] Shao D. Moberly A. C. Ray C. (2020). Quality of life outcomes reported by patients and significant others following cochlear implantation. *Am. J. Audiol.* 29 404–409. 10.1044/2020_AJA-19-00101 32598160

[B97] Stewart M. C. Arnold C. L. (2018). Defining social listening: recognizing an emerging dimension of listening. *Int. J. Listening* 32 85–100. 10.1080/10904018.2017.1330656

[B98] Sung Y. K. Li L. Blake C. Betz J. Lin F. R. (2016). Association of hearing loss and loneliness in older adults. *J. Aging Health* 28 979–994. 10.1177/0898264315614570 26597841

[B99] Terwee C. B. Prinsen C. A. C. Chiarotto A. Westerman M. J. Patrick D. L. Alonso J. (2018). COSMIN methodology for evaluating the content validity of patient-reported outcome measures: a Delphi study. *Qual. Life Res.* 27 1159–1170. 10.1007/s11136-018-1829-1820 29550964 PMC5891557

[B100] Thibodeau L. (2007). “Speech audiometry,” in *Audiology Diagnosis*, 2nd Edn, ed. Hosford-Dunn R. (New York, NY: Thieme Publishing Group), 228–313.

[B101] Tricco A. C. Lillie E. Zarin W. O’Brien K. K. Colquhoun H. Levac D. (2018). PRISMA extension for scoping reviews (PRISMA-ScR) : checklist and explanation. *Ann. Intern. Med.* 169 467–473. 10.7326/M18-0850 30178033

[B102] Vas V. Akeroyd M. A. Hall D. A. (2017). A data-driven synthesis of research evidence for domains of hearing loss, as reported by adults with hearing loss and their communication partners. *Trends Hear.* 21:233121651773408. 10.1177/2331216517734088 28982021 PMC5638151

[B103] Ventry I. M. Weinstein B. E. (1982). The hearing handicap inventory for the elderly: a new tool. *Ear Hear.* 3 128–134. 10.1097/00003446-198205000-198205006 7095321

[B104] Vermiglio A. Soli S. D. Fang X. (2018). An argument for self-report as a reference standard in audiology. *J. Am. Acad. Audiol.* 29 206–222. 10.3766/jaaa.16128 29488871

[B105] Wallhagen M. I. Pettengill E. (2008). Hearing impairment. *J. Gerontol. Nursing* 34 36–42. 10.3928/00989134-20080201-20080212 18286791

[B106] Walsh R. (2011). Looking at the ICF and human communication through the lens of classification theory. *Int. J. Speech-Lang. Pathol.* 13 348–359. 10.3109/17549507.2011.550690 21480813

[B107] Wayne R. V. Hamilton C. Huyck J. J. Johnsrude I. S. (2016). Working memory training and speech in noise comprehension in older adults. *Front. Aging Neurosci.* 8:49. 10.3389/fnagi.2016.00049 27047370 PMC4801856

[B108] Weisser A. Buchholz J. M. (2019). Conversational speech levels and signal-to-noise ratios in realistic acoustic conditions. *J. Acoust. Soc. Am.* 145 349–349. 10.1121/1.5087567 30710956

[B109] Wolvin A. D. Wiley I. (2010). *Listening and Human Communication in the 21st century.* Malden, MA: Wiley-Blackwell.

[B110] Wong L. L. N. Yu J. K. Y. Chan S. S. Tong M. C. F. (2014). Screening of cognitive function and hearing impairment in older adults: a preliminary study. *BioMed Res. Int.* 2014:867852. 10.1155/2014/867852 25140321 PMC4130188

[B111] World Health Organization (2001). *International Classification of Functioning, Disability and Health (ICF).* Geneva: WHO Press.

[B112] World Health Organization (2017). *Global Costs of Unaddressed Hearing Loss and Cost-effectiveness of Interventions: a WHO report, 2017.* Geneva: World Health Organization.

[B113] Worthington D. L. (2018). “Modelling and measuring the cognitive components of listening,” in *The Sourcebook of Listening Research: Methodology and Measures*, eds Worthington D. L. Bodie G. (Hoboken, NJ: John Wiley & Sons, Inc).

[B114] Zhou X. Seghouane A. Shah A. Innes-Brown H. Cross W. Litovsky R. (2018). Cortical speech processing in postlingually deaf adult cochlear implant users, as revealed by functional near-infrared spectroscopy. *Trends Hear.* 22:2331216518786850. 10.1177/2331216518786850 30022732 PMC6053859

