# Supplementary material for: Listening-Based Communication Ability in Adults With Hearing Loss: A Scoping Review of Existing Measures
Source: Front Psychol. 2022 Mar 10;13:786347. doi: 10.3389/fpsyg.2022.786347 (PMC8960922; doi:10.3389/fpsyg.2022.786347)
Supplement: Supplementary file 1 [file Data_Sheet_1.PDF]

Supplementary material A. Search strategy developed in Medline

	Search Terms
1	Communication
2	(spoken or Oral or verbal or speech or language*).mp. [mp=title, abstract, original title, name of substance word, subject heading word, keyword heading word, protocol supplementary concept word, rare disease supplementary concept word, unique identifier, synonyms]
3	*1 and 2*<br>*(Communication and spoken or oral or verbal or speech or language)*
4	(hearing or auditory or aural* or audition or listen*).mp. [mp=title, abstract, original title, name of substance word, subject heading word, keyword heading word, protocol supplementary concept word, rare disease supplementary concept word, unique identifier, synonyms]
5	*3 or 4*<br>*(Communication and spoken or oral or verbal or speech or language or hearing or auditory or aural* or audition or listen*)*
6	(assess* or evalu* or measur* or diagnos* or index or scale or test*).mp.
7	*hearing tests/ or audiometry/ or audiometry, evoked response/ or audiometry, pure-tone/ or audiometry, speech/ or psychoacoustics/ or dichotic listening tests/
8	speech perception.mp.
9	*6 or 7 or 8*<br>*(assess* or evalu* or measur* or diagnos* or index or scale or test* or *hearing tests/ or audiometry/ or audiometry, evoked response/ or audiometry, pure-tone/ or audiometry, speech/ or psychoacoustics/ or dichotic listening tests/or speech perception.mp.)*
10	(deaf* or hearing impair* or hearing loss* or hearing disorder*).mp.
11	(cochlear implant* or hearing aid*).mp. [mp=title, abstract, original title, name of substance word, subject heading word, keyword heading word, protocol supplementary concept word, rare disease supplementary concept word, unique identifier, synonyms]
12	*10 or 11*<br>*(deaf* or hearing impair* or hearing loss* or hearing disorder*or cochlear implant* or hearing aid*)*
13	*5 and 9 and 12*<br>*(Communication and spoken or oral or verbal or speech or language or hearing or auditory or aural* or audition or listen*) AND (assess* or evalu* or measur* or diagnos* or index or scale or test* or *hearing tests/ or audiometry/ or audiometry, evoked response/ or audiometry, pure-tone/ or audiometry, speech/ or psychoacoustics/ or dichotic listening tests/or speech perception.mp.) AND (deaf* or hearing impair* or hearing loss* or hearing disorder*or cochlear implant* or hearing aid*)*
	Search Filters
14	(pediatric or paediatric or child* or infant).mp. [mp=title, abstract, original title, name of substance word, subject heading word, keyword heading word, protocol supplementary concept word, rare disease supplementary concept word, unique identifier, synonyms]
15	*13 not 14*
16	*limit 15 to (english language and yr="2008 -Current" and "all adult (19 plus years)")= 8026*
