# Supplementary material for: Listening-Based Communication Ability in Adults With Hearing Loss: A Scoping Review of Existing Measures
Source: Front Psychol. 2022 Mar 10;13:786347. doi: 10.3389/fpsyg.2022.786347 (PMC8960922; doi:10.3389/fpsyg.2022.786347)
Supplement: Supplementary file 2 [file Data_Sheet_2.PDF]

TITLE	AUTH	PUB
Electric acoustic stimulation of the auditory system: Results of a multi-centre investigation	Gstoettner, W. K.; Van De Heyning, P.; Fitzgerald O'Connor, A.; Morera, C.; Sainz, M.; Vermeire, K.; McDonald, S.; Cavalle, L.; Helbig, S.; Garcia Valdecasas, J.; Anderson, I.; Adunka, O. F.	2008
Benefits from a mobile telephone adapter in Combi40+ cochlear implant users	Castro, A.; Lassaletta, L.; Bastarrica, M.; Perez-Mora, R.; Josefa De Sarria, M.; Gavilan, J.	2008
Comparison of hearing thresholds obtained using pure-tone behavioral audiometry, the Cantonese Hearing in Noise Test (CHINT) and cortical evoked response audiometry	Wong, L. L. N.; Cheung, C.; Wong, E. C. M.	2008
Psychological profile and social behaviour of working adults with mild or moderate hearing loss	Monzani, D.; Galeazzi, G. M.; Genovese, E.; Marrara, A.; Martini, A.	2008
A Mini-Mental Status Examination for the hearing impaired	De Silva, M. L.; McLaughlin, M. T.; Rodrigues, E. J.; Broadbent, J. C.; Gray, A. R.; Hammond-Tooke, G. D.	2008
Can auditory and visual speech perception be trained within a group setting?	Preminger, J. E.; Ziegler, C. H.	2008
The Hearing-Dependent Daily Activities Scale to Evaluate Impact of Hearing Loss in Older People	Hidalgo, J. L. T.; Gras, C. B.; Lapeira, J. M. T.; Martinez, I. P.; Verdejo, M. A. L.; Rabadan, F. E.; Puime, A. O.	2008
Phonological awareness and short-term memory in hearing and deaf individuals of different communication backgrounds	Koo, D.; Crain, K.; LaSasso, C.; Eden, G. F.	2008

Audiometric characteristics of a Dutch family linked to DFNA15 with a novel mutation (p.L289F) in POU4F3	Pauw, R. J.; van Drunen, F. J.; Collin, R. W.; Huygen, P. L.; Kremer, H.; Cremers, C. W.	2008
Central auditory dysfunction in older persons with memory impairment or Alzheimer dementia	Gates, G. A.; Anderson, M. L.; Feeney, M. P.; McCurry, S. M.; Larson, E. B.	2008
Renewed prescription of hearing aids: A clinically based study	Helvik, A. S.; Iversen, V. C.; Wennberg, S.; Jacobsen, G. W.	2008
The effects of a sound awareness pre-fitting intervention: A randomized controlled trial	Oberg, M.; Andersson, G.; Wanstrom, G.; Lunner, T.	2008
Why do some individuals with objectively verified hearing loss reject hearing aids?	Helvik, A. S.; Wennberg, S.; Jacobsen, G.; Hallberg, L. R. M.	2008
Speech recognition materials and ceiling effects: Considerations for cochlear implant programs	Gifford, R. H.; Shallop, J. K.; Peterson, A. M.	2008
Factors associated with self-rated health in older people living in institutions	Damian, J.; Pastor-Barriuso, R.; Valderrama-Gama, E.	2008
Prevalence of high frequency hearing loss consistent with noise exposure among people working with sound systems and general population in Brazil: A cross-sectional study	El Dib, R. P.; Silva, E. M. K.; Morais, J. F.; Trevisani, V. F. M.	2008
McGurk effects in cochlear-implanted deaf subjects	Rouger, J.; Fraysse, B.; Deguine, O.; Barone, P.	2008
The relationship between changes in self-rated quality of life after cochlear implantation and changes in individual complaints	Zhao, F.; Bai, Z.; Stephens, D.	2008
Performance benefits for adults using a cochlear implant with adaptive dynamic range optimization (ADRO): a comparative study	Muller-Deile, J.; Kiefer, J.; Wyss, J.; Nicolai, J.; Battmer, R.	2008

Comparison of rehabilitation results in deaf patients with and without genetically related hearing loss	Wrobel, M.; Magierska-Krzyszton, M.; Mietkiewska, D.; Szyfter, W.; Rydzanicz, M.; Szyfter, K.; Karlik, M.	2008
Self-reported hearing difficulties, communication strategies and psychological general well-being (quality of life) in patients with acquired hearing impairment	Hallberg, L. R. M.; Hallberg, U.; Kramer, S. E.	2008
Hearing handicap ratings among different profiles of adult cochlear implant users	Noble, W.; Tyler, R.; Dunn, C.; Bhullar, N.	2008
Aging and speech-on-speech masking	Helfer, K. S.; Freyman, R. L.	2008
Multicenter U.S. bilateral MED-EL cochlear implantation study: speech perception over the first year of use	Buss, E.; Pillsbury, H. C.; Buchman, C. A.; Pillsbury, C. H.; Clark, M. S.; Haynes, D. S.; Labadie, R. F.; Amberg, S.; Roland, P. S.; Kruger, P.; Novak, M. A.; Wirth, J. A.; Black, J. M.; Peters, R.; Lake, J.; Wackym, P. A.; Firszt, J. B.; Wilson, B. S.; Lawson, D. T.; Schatzer, R.; D'Haese, P. S.; Barco, A. L.	2008
Comparison of speech recognition and localization performance in bilateral and unilateral cochlear implant users matched on duration of deafness and age at implantation	Dunn, C. C.; Tyler, R. S.; Oakley, S.; Gantz, B. J.; Noble, W.	2008
Psychophysical versus physiological spatial forward masking and the relation to speech perception in cochlear implants	Hughes, M. L.; Stille, L. J.	2008
A prospective multi-centre study of the benefits of bilateral hearing aids	Boymans, M.; Goverts, S. T.; Kramer, S. E.; Festen, J. M.; Dreschler, W. A.	2008

Cross-sectional age-changes of hearing in the elderly	Gates, G. A.; Feeney, M. P.; Mills, D.	2008
A comparative study of the audiological outcomes with Retro-X (semi-implantable hearing aid system) and conventional open fitting hearing aids	Lenarz, M.; Lenarz, T.; Stieve, M.; Winter, M.	2008
Comparison of speech intelligibility in quiet and in noise after hearing aid fitting according to a purely prescriptive and a comparative fitting procedure	Metselaar, M.; Maat, B.; Krijnen, P.; Verschuure, H.; Dreschler, W.; Feenstra, L.	2008
Electric to acoustic pitch matching: A possible way to improve individual cochlear implant fitting	Nardo, W. D.; Cantore, I.; Marchese, M. R.; Cianfrone, F.; Scorpecci, A.; Giannantonio, S.; Paludetti, G.	2008
Integrating the acoustics of running speech into the pure tone audiogram: a step from audibility to intelligibility and disability	Corthals, P.	2008
Persons with acquired profound hearing loss (APHL): how do they and their families adapt to the challenge?	Hallam, R.; Ashton, P.; Sherbourne, K.; Gailey, L.	2008
Processing F0 with cochlear implants: Modulation frequency discrimination and speech intonation recognition	Chatterjee, M.; Peng, S. C.	2008
Perceptual integration between target speech and target-speech reflection reduces masking for target-speech recognition in younger adults and older adults	Huang, Y.; Huang, Q.; Chen, X.; Qu, T. S.; Wu, X. H.; Li, L.	2008
Unilateral and bilateral cochlear implants and the implant-plus-hearing-aid profile: comparing self-assessed and measured abilities	Noble, W.; Tyler, R.; Dunn, C.; Bhullar, N.	2008
Self reported hearing difficulties following excision of vestibular schwannoma	McLeod, B.; Upfold, L.; Taylor, A.	2008
Digital noise reduction: outcomes from laboratory and field studies	Bentler, R.; Wu, Y. H.; Kettel, J.; Hurtig, R.	2008
Evaluation of binaural functions in bilateral cochlear implant users	Chan, J. C.; Freed, D. J.; Vermiglio, A. J.; Soli, S. D.	2008
The interference of different background noises on speech processing in elderly hearing impaired subjects	Larsby, B.; Hallgren, M.; Lyxell, B.	2008

New cochlear implant coding strategy for tonal language speakers	Wong, L. L.; Vandali, A. E.; Ciocca, V.; Luk, B.; Ip, V. W.; Murray, B.; Yu, H. C.; Chung, I.	2008
Phonological mismatch and explicit cognitive processing in a sample of 102 hearing-aid users	Rudner, M.; Foo, C.; Sundewall-Thoren, E.; Lunner, T.; Ronnberg, J.	2008
Discrimination of Schroeder-phase harmonic complexes by normal-hearing and cochlear-implant listeners	Drennan, W. R.; Longnion, J. K.; Ruffin, C.; Rubinstein, J. T.	2008
Hearing impairment: significant but underassessed in primary care settings	Wallhagen, M. I.; Pettengill, E.	2008
Perception of hearing status, communication, and hearing aids among socially active older individuals	Rawool, V. W.; Keihl, J. M.	2008
Results of intratympanic dexamethasone injection as salvage treatment in idiopathic sudden hearing loss	Lee, H. S.; Jun, M. K.; Kim, Y. J.; Duk, H. C.; Beom, S. S.; Seok, H. K.	2008
Impact of cochlear implantation on speech understanding, depression, and loneliness in the elderly	Poissant, S. F.; Beaudoin, F.; Huang, J.; Brodsky, J.; Lee, D. J.	2008
Over-the-fence cochlear implantation: is it worthwhile?	Tremblay, G.; Bergeron, F.; Ferron, P.	2008
Integration of acoustic and electrical hearing	Turner, C.; Gantz, B. J.; Reiss, L.	2008
Is It Really Clear? Adapting Research Tools for the Needs of the Deaf Population	Levinger, M.; Ronen, T.	2008
Acceptance of noise growth patterns in hearing aid users	Freyaldenhoven, M. C.; Plyler, P. N.; Thelin, J. W.; Muenchen, R. A.	2008
Multichannel compression: effects of reduced spectral contrast on vowel identification	Bor, S.; Souza, P.; Wright, R.	2008
Factors affecting the benefits of high-frequency amplification	Horwitz, A. R.; Ahlstrom, J. B.; Dubno, J. R.	2008
Subjective and objective effects of fast and slow compression on the perception of reverberant speech in listeners with hearing loss	Shi, L. F.; Doherty, K. A.	2008

Longitudinal changes in speech recognition in older persons	Dubno, J. R.; Lee, F. S.; Matthews, L. J.; Ahlstrom, J. B.; Horwitz, A. R.; Mills, J. H.	2008
The role of temporal cues in word identification by younger and older adults: effects of sentence context	Gordon-Salant, S.; Yeni-Komshian, G.; Fitzgibbons, P.	2008
Spectral weighting strategies for hearing-impaired listeners measured using a correlational method	Calandruccio, L.; Doherty, K. A.	2008
Using blind source separation techniques to improve speech recognition in bilateral cochlear implant patients	Kokkinakis, K.; Loizou, P. C.	2008
The effects of hearing loss and age on the benefit of spatial separation between multiple talkers in reverberant rooms	Marrone, N.; Mason, C. R.; Kidd, G., Jr.	2008
Intra- versus intermodal integration in young and older adults	Spehar, B. P.; Tye-Murray, N.; Sommers, M. S.	2008
Speech recognition in noise as a function of highpass-filter cutoff frequency for people with and without low-frequency cochlear dead regions	Vinay,; Baer, T.; Moore, B. C.	2008
Measuring cochlear implant satisfaction in postlingually deafened adults with the SADL inventory	Ou, H.; Dunn, C. C.; Bentler, R. A.; Zhang, X.	2008
Optimizing the benefit of sound processors coupled to personal FM systems	Wolfe, J.; Schafer, E. C.	2008
Multivariate predictors of music perception and appraisal by adult cochlear implant users	Gfeller, K.; Oleson, J.; Knutson, J. F.; Breheny, P.; Driscoll, V.; Olszewski, C.	2008
Investigation of potential cognitive tests for use with older adults in audiology clinics	Vaughan, N.; Storzbach, D.; Furukawa, I.	2008
User preference and reliability of bilateral hearing aid gain adjustments	Hornsby, B. W. Y.; Mueller, H. G.	2008
Speech perception in noise using directional microphones in open-canal hearing aids	Klemp, E. J.; Dhar, S.	2008
Speech understanding using surgical masks: a problem in health care?	Mendel, L. L.; Gardino, J. A.; Atcherson, S. R.	2008
A comparison of two word-recognition tasks in multitalker babble: Speech Recognition in Noise Test (SPRINT) and Words-in-Noise Test (WIN)	Wilson, R. H.; Cates, W. B.	2008
Effects of expansion algorithms on speech reception thresholds	Wise, C. L.; Zakis, J. A.	2008

Effect of preoperative residual hearing on speech perception after cochlear implantation	Adunka, O. F.; Buss, E.; Clark, M. S.; Pillsbury, H. C.; Buchman, C. A.	2008
Long-term hearing results in gamma knife radiosurgery for acoustic neuromas	Bush, M. L.; Shinn, J. B.; Young, A. B.; Jones, R. O.	2008
Audiological application criteria for implantable hearing aid devices: a clinical experience at the Nijmegen ORL clinic	Verhaegen, V. J.; Mylanus, E. A.; Cremers, C. W.; Snik, A. F.	2008
Outcomes of treatment of partial deafness with cochlear implantation: a DUET study	Lorens, A.; Polak, M.; Piotrowska, A.; Skarzynski, H.	2008
Audiovestibular manifestations in patients with limited systemic sclerosis and centromere protein-B (CENP-B) antibodies	Amor-Dorado, J. C.; Arias-Nunez, M. C.; Miranda-Filloy, J. A.; Gonzalez-Juanatey, C.; Llorca, J.; Gonzalez-Gay, M. A.	2008
Prediction of the Pure-Tone Average from the Speech Reception and Auditory Brainstem Response Thresholds in a Geriatric Population	Chien, C. H.; Tu, T. Y.; Shiao, A. S.; Chien, S. F.; Wang, Y. F.; Li, A. C. I.; Yang, M. J.	2008
Measurement of hearing aid outcome in the elderly: comparison between young and old elderly	Chang, W. H.; Tseng, H. C.; Chao, T. K.; Hsu, C. J.; Liu, T. C.	2008
Combination therapy (intratympanic dexamethasone plus high-dose prednisone taper) for the treatment of idiopathic sudden sensorineural hearing loss	Battaglia, A.; Burchette, R.; Cueva, R.	2008
Audiologic outcomes with the penetrating electrode auditory brainstem implant	Otto, S. R.; Shannon, R. V.; Wilkinson, E. P.; Hitselberger, W. E.; McCreery, D. B.; Moore, J. K.; Brackmann, D. E.	2008
Are stage IV vestibular schwannomas preoperatively different from other stages?	Tringali, S.; Dubreuil, C.; Zaouche, S.; Ferber-Viart, C.	2008

Clinical relevance of quality of life outcome in cochlear implantation in postlingually deafened adults	Klop, W. M.; Boermans, P. P.; Ferrier, M. B.; van den Hout, W. B.; Stiggelbout, A. M.; Frijns, J. H.	2008
Results from a psychoacoustic model-based strategy for the nucleus-24 and freedom cochlear implants	Buchner, A.; Nogueira, W.; Edler, B.; Battmer, R. D.; Lenarz, T.	2008
Role of electrode placement as a contributor to variability in cochlear implant outcomes	Finley, C. C.; Holden, T. A.; Holden, L. K.; Whiting, B. R.; Chole, R. A.; Neely, G. J.; Hullar, T. E.; Skinner, M. W.	2008
The impact of cochlear implants from the perspective of significant others of adult cochlear implant users	Kennedy, V.; Stephens, D.; Fitzmaurice, P.	2008
Cochlear implant speech processor frequency allocations may influence pitch perception	Reiss, L. A. J.; Gantz, B. J.; Turner, C. W.	2008
Quality of life in patients with idiopathic sudden hearing loss: Comparison of different therapies using the Medical Outcome Short Form (36) Health Survey questionnaire	Mosges, R.; Koberlein, J.; Erdtracht, B.; Klingel, R.	2008
A new fine structure speech coding strategy: Speech perception at a reduced number of channels	Riss, D.; Arnoldner, C.; Baumgartner, W. D.; Kaider, A.; Hamzavi, J. S.	2008
Performance of elderly in a speech in noise test	Calais, L. L.; Russo, I. C. P.; De Carvalho Borges, A. C. L.	2008
Formal auditory training in elderly hearing aid users	de Miranda, E. C.; Gil, D.; Iorio, M. C. M.	2008
Social cognition as a mediator of cognition and outcome among deaf and hearing people with schizophrenia	Horton, H. K.; Silverstein, S. M.	2008
The effects of cueing temporal and spatial attention on word recognition in a complex listening task in hearing-impaired listeners	Gatehouse, S.; Akeroyd, M. A.	2008
Evaluating the benefit of hearing aids in solving the cocktail party problem	Marrone, N.; Mason, C. R.; Kidd, G., Jr.	2008

The effect of hearing loss and hearing aids on the use of information and communication technologies by community-living older adults	Gonsalves, Chris; Pichora-Fuller, Margaret Kathleen %J Canadian Journal on Aging/La Revue canadienne du vieillissement	2008
The relationship between hearing impairment and cognitive function: a 5-year longitudinal study	Wallhagen, Margaret I; Strawbridge, William J; Shema, Sarah J %J Research in Gerontological Nursing	2008
Auditory-visual discourse comprehension by older and young adults in favorable and unfavorable conditions	Tye-Murray, Nancy; Sommers, Mitchell; Spehar, Brent; Myerson, Joel; Hale, Sandra; Rose, Nathan S %J International Journal of Audiology	2008
The benefits of combining acoustic and electric stimulation for the recognition of speech, voice and melodies	Dorman, Michael F; Gifford, Rene H; Spahr, Anthony J; McKarns, Sharon A %J Audiology; Neurotology	2008
Speech recognition and temporal amplitude modulation processing by Mandarin-speaking cochlear implant users	Luo, Xin; Fu, Qian-Jie; Wei, Chao-Gang; Cao, Ke-Li %J Ear; hearing	2008
TORP vs round window implant for hearing restoration of patients with extensive ossicular chain defect	Colletti, V.; Carner, M.; Colletti, L.	2009
Screening for Hearing Loss in the Elderly Using Distortion Product Otoacoustic Emissions, Pure Tones, and a Self-Assessment Tool	Jupiter, T.	2009
Hearing aid outcomes for listeners with high-frequency hearing loss	Roup, C. M.; Noe, C. M.	2009
Radiographic analysis of cochlear nerve vascular compression	Clift, J. M.; Wong, R. D.; Carney, G. M.; Stavinoha, R. C.; Boyev, K. P.	2009

Profound mixed hearing loss: bone-anchored hearing aid system or cochlear implant?	Verhaegen, V. J.; Mulder, J. J.; Mylanus, E. A.; Cremers, C. W.; Snik, A. F.	2009
Self-reported listening habits and enjoyment of music among adult cochlear implant recipients	Migirov, L.; Kronenberg, J.; Henkin, Y.	2009
Functional status of elderly people with hearing loss	Lopez-Torres Hidalgo, J.; Boix Gras, C.; Tellez Lapeira, J.; Lopez Verdejo, M. A.; del Campo del Campo, J. M.; Escobar Rabadan, F.	2009
Quality of life improvement in hearing-impaired elderly people after wearing a hearing aid	Lotfi, Y.; Mehrkian, S.; Moossavi, A.; Faghih-Zadeh, S.	2009
Binaural hearing after cochlear implantation in subjects with unilateral sensorineural deafness and tinnitus	Vermeire, K.; Van de Heyning, P.	2009
Effects of cooperating and conflicting cues on speech intonation recognition by cochlear implant users and normal hearing listeners	Peng, S. C.; Lu, N.; Chatterjee, M.	2009
Hybrid 10 clinical trial: preliminary results	Gantz, B. J.; Hansen, M. R.; Turner, C. W.; Oleson, J. J.; Reiss, L. A.; Parkinson, A. J.	2009
Impact of low-frequency hearing	Buchner, A.; Schussler, M.; Battmer, R. D.; Stover, T.; Lesinski-Schiedat, A.; Lenarz, T.	2009
Cortical neural activity underlying speech perception in postlingual adult cochlear implant recipients	Henkin, Y.; Tetin-Schneider, S.; Hildesheimer, M.; Kishon-Rabin, L.	2009
Hearing conservation surgery using the Hybrid-L electrode. Results from the first clinical trial at the Medical University of Hannover	Lenarz, T.; Stover, T.; Buechner, A.; Lesinski-Schiedat, A.; Patrick, J.; Pesch, J.	2009

Speech performance and sound localization in a complex noisy environment in bilaterally implanted adult patients	Mosnier, I.; Sterkers, O.; Bebear, J. P.; Godey, B.; Robier, A.; Deguine, O.; Fraysse, B.; Bordure, P.; Mondain, M.; Bouccara, D.; Bozorg-Grayeli, A.; Borel, S.; Ambert-Dahan, E.; Ferrary, E.	2009
Word recognition following implantation of conventional and 10-mm hybrid electrodes	Dorman, M. F.; Gifford, R.; Lewis, K.; McKarns, S.; Ratigan, J.; Spahr, A.; Shallop, J. K.; Driscoll, C. L. W.; Luetje, C.; Thedinger, B. S.; Beatty, C. W.; Syms, M.; Novak, M.; Barrs, D.; Cowdrey, L.; Black, J.; Loiselle, L.	2009
Assessing binaural/bimodal advantages using auditory event-related potentials in subjects with cochlear implants	Sasaki, T.; Yamamoto, K.; Iwaki, T.; Kubo, T.	2009
Techniques and results of stapedial surgery	Fakir, M. A. Y.; Bhuyan, M. A. H.; Al-Masum, S. H. I.; Rahman, H. M. M.; Uddin, M. M.; Joarder, M. A. H.; Alauddin, M.	2009
Hearing aid effectiveness after aural rehabilitation - individual versus group (HEARING) trial: RCT design and baseline characteristics	Collins, M. P.; Souza, P. E.; Liu, C. F.; Heagerty, P. J.; Amtmann, D.; Yueh, B.	2009
Sensitivity, specificity and predictive values of hearing loss to different audiometric mean values	Calviti, K. C.; Pereira, L. D.	2009
Speech recognition and working memory capacity in young-elderly listeners: effects of hearing sensitivity	Cervera, T. C.; Soler, M. J.; Dasi, C.; Ruiz, J. C.	2009

Psychometric validity of the Cochlear Implant Function Index (CIFI): a quality of life assessment tool for adult cochlear implant users	Coelho, D. H.; Hammerschlag, P. E.; Bat-Chava, Y.; Kohan, D.	2009
Update on long-term results with auditory brainstem implants in NF2 patients	Maini, S.; Cohen, M. A.; Hollow, R.; Briggs, R.	2009
Baha solutions for patients with severe mixed hearing loss	Flynn, M. C.; Sadeghi, A.; Halvarsson, G.	2009
HiRes with Fidelity 120 benefit in native speakers of Korean	Park, H. J.; Lee, S. C.; Chun, Y. M.; Lee, J. Y.	2009
BKB-SIN and ANL predict perceived communication ability in cochlear implant users	Donaldson, G. S.; Chisolm, T. H.; Blasco, G. P.; Shinnick, L. J.; Ketter, K. J.; Krause, J. C.	2009
Development and validation of the University of Washington Clinical Assessment of Music Perception test	Kang, R.; Nimmons, G. L.; Drennan, W.; Longnion, J.; Ruffin, C.; Nie, K.; Won, J. H.; Worman, T.; Yueh, B.; Rubinstein, J.	2009
The benefits of remote microphone technology for adults with cochlear implants	Fitzpatrick, E. M.; Seguin, C.; Schramm, D. R.; Armstrong, S.; Chenier, J.	2009
Spatial benefit of bilateral hearing AIDS	Ahlstrom, J. B.; Horwitz, A. R.; Dubno, J. R.	2009
Effects of audibility and multichannel wide dynamic range compression on consonant recognition for listeners with severe hearing loss	Davies-Venn, E.; Souza, P.; Brennan, M.; Stecker, G. C.	2009
The benefits of hearing aids and closed captioning for television viewing by older adults with hearing loss	Gordon-Salant, S.; Callahan, J. S.	2009
Development and efficacy of a frequent-word auditory training protocol for older adults with impaired hearing	Humes, L. E.; Burk, M. H.; Strauser, L. E.; Kinney, D. L.	2009
Spatial hearing and speech intelligibility in bilateral cochlear implant users	Litovsky, R. Y.; Parkinson, A.; Arcaroli, J.	2009

The influence of age, hearing, and working memory on the speech comprehension benefit derived from an automatic speech recognition system	Zekveld, A. A.; Kramer, S. E.; Kessens, J. M.; Vlaming, M. S.; Houtgast, T.	2009
The association between hearing status and psychosocial health before the age of 70 years: results from an internet-based national survey on hearing	Nachtegaal, J.; Smit, J. H.; Smits, C.; Bezemer, P. D.; van Beek, J. H.; Festen, J. M.; Kramer, S. E.	2009
The Factors Associated with a Self-Perceived Hearing Handicap in Elderly People with Hearing Impairment-Results from a Community-Based Study	Chang, H. P.; Ho, C. Y.; Chou, P.	2009
Self-reported disability and handicap after hearing-aid fitting and benefit of hearing aids: comparison of fitting procedures, degree of hearing loss, experience with hearing aids and uni- and bilateral fittings	Metselaar, M.; Maat, B.; Krijnen, P.; Verschuure, H.; Dreschler, W. A.; Feenstra, L.	2009
Rheopheresis for idiopathic sudden hearing loss: Results from a large prospective, multicenter, randomized, controlled clinical trial	Mosges, R.; Koberlein, J.; Heibges, A.; Erdtracht, B.; Klingel, R.; Lehmacher, W.	2009
Use of intratympanic dexamethasone for the therapy of low frequency hearing loss	Alatas, N.	2009
Comprehension of Speeded Discourse by Younger and Older Listeners	Gordon, M. S.; Daneman, M.; Schneider, B. A.	2009
Using hearing aid adaptive directional microphones to enhance cochlear implant performance	Chung, K.; Zeng, F. G.	2009
Noise induced hearing loss and other hearing complaints among musicians of symphony orchestras	Jansen, E. J.; Helleman, H. W.; Dreschler, W. A.; de Laat, J. A.	2009
The effect of extending high-frequency bandwidth on the Acceptable Noise Level (ANL) of hearing-impaired listeners	Johnson, E.; Ricketts, T.; Hornsby, B.	2009
Audiovisual asynchrony detection and speech perception in hearing-impaired listeners with cochlear implants: a preliminary analysis	Hay-McCutcheon, M. J.; Pisoni, D. B.; Hunt, K. K.	2009
Environmental noise reduction configuration: Effects on preferences, satisfaction, and speech understanding	Zakis, J. A.; Hau, J.; Blamey, P. J.	2009

Benefit from spatial separation of multiple talkers in bilateral hearing-aid users: Effects of hearing loss, age, and cognition	Neher, T.; Behrens, T.; Carlile, S.; Jin, C.; Kragelund, L.; Petersen, A. S.; Schaik, A. V.	2009
The perception of prosody and speaker gender in normal-hearing listeners and cochlear implant recipients	Meister, H.; Landwehr, M.; Pyschny, V.; Walger, M.; Wedel, H. V.	2009
Cortical auditory steady-state responses to low modulation rates	Alaerts, J.; Luts, H.; Hofmann, M.; Wouters, J.	2009
Fitting range of the BAHA Intenso	Bosman, A. J.; Snik, F. M.; Mylanus, E. A.; Cremers, W. R.	2009
Development of Mandarin monosyllabic speech test materials in China	Han, D.; Wang, S.; Zhang, H.; Chen, J.; Jiang, W.; Mannell, R.; Newall, P.; Zhang, L.	2009
Hearing status, need for recovery after work, and psychosocial work characteristics: results from an internet-based national survey on hearing	Nachtegaal, J.; Kuik, D. J.; Anema, J. R.; Goverts, S. T.; Festen, J. M.; Kramer, S. E.	2009
Effects of sensorineural hearing loss on visually guided attention in a multitalker environment	Best, V.; Marrone, N.; Mason, C. R.; Kidd Jr, G.; Shinn-Cunningham, B. G.	2009
The health impact of a hearing disability on older people in Australia	Hogan, A.; O'Loughlin, K.; Miller, P.; Kendig, H.	2009
The prognostic value of speech recognition scores at diagnosis of vestibular schwannoma	Remenyi, J.; Marshall, A.; Enticott, J. C.; Briggs, R. J.	2009
Results of Factorial Validity and Reliability of the International Outcome Inventory for Hearing Aids in Turkish	Serbetcioglu, B.; Mutlu, B.; Kirkilm, G.; Uzunoglu, S.	2009
Hypobaric pressure exposure effects on cochlear frequency selectivity in fluctuating, low-frequency hearing loss	Brannstrom, K. J.; Grenner, J.	2009
Hearing results following intratympanic gentamicin perfusion for Meniere's disease	Kyrodimos, E.; Aidonis, I.; Sismanis, A.	2009

Randomised, double-blinded, placebo-controlled, clinical trial of ozone therapy as treatment of sudden sensorineural hearing loss	Ragab, A.; Shreef, E.; Behiry, E.; Zalat, S.; Noaman, M.	2009
Hearing results of 1145 stapedotomies evaluated with Amsterdam hearing evaluation plots	Kisilevsky, V. E.; Dutt, S. N.; Bailie, N. A.; Halik, J. J.	2009
Peripheral neuropathy and VIII cranial nerve involvement in fabry disease	Reisin, R.; Marchesoni, C.; Carmona, S.; Pardal, A.; Kisinovsky, I.; Caceres, G.; Birmingham, M.; Moreno, M.; Maira, S.; Acosta, P.	2009
Patterns of hearing loss after microvascular decompression for hemifacial spasm	Park, K.; Hong, S. H.; Hong, S. D.; Cho, Y. S.; Chung, W. H.; Gyu Ryu, N.	2009
Speech recognition in younger and older adults: a dependency on low-level auditory cortex	Harris, K. C.; Dubno, J. R.; Keren, N. I.; Ahlstrom, J. B.; Eckert, M. A.	2009
Predictors of hearing preservation after stereotactic radiosurgery for acoustic neuroma	Kano, H.; Kondziolka, D.; Khan, A.; Flickinger, J. C.; Lunsford, L. D.	2009
Hearing preservation in surgery for large vestibular schwannomas	Wanibuchi, M.; Fukushima, T.; McElveen, J. T., Jr.; Friedman, A. H.	2009
Indications for Bone-Anchored Hearing Aids: A Functional Outcomes Study	Dumper, J.; Hodgetts, B.; Liu, R.; Brandner, N.	2009
The Markham-Stouffville hospital experience with malleovestibulopexy	Kisilevsky, V.; Bailie, N. A.; Dutt, S. N.; Safar, A.; Halik, J. J.	2009

Sensorineural hearing loss in patients with mixed connective tissue disease: immunological markers and cytokine levels	Hajas, A.; Szodoray, P.; Barath, S.; Sipka, S.; Rezes, S.; Zeher, M.; Sziklai, I.; Szegedi, G.; Bodolay, E.	2009
Candidacy for bilateral hearing aids: a retrospective multicenter study	Boymans, M.; Goverts, S. T.; Kramer, S. E.; Festen, J. M.; Dreschler, W. A.	2009
Voiced initial consonant perception deficits in older listeners with hearing loss and good and poor word recognition	Phillips, S. L.; Richter, S. J.; McPherson, D.	2009
Spectral tilt change in stop consonant perception by listeners with hearing impairment	Alexander, J. M.; Kluender, K. R.	2009
The effect of temporal gap identification on speech perception by users of cochlear implants	Sagi, E.; Kaiser, A. R.; Meyer, T. A.; Svirsky, M. A.	2009
Message and talker identification in older adults: Effects of task, distinctiveness of the talkers' voices, and meaningfulness of the competing message	Rossi-Katz, J.; Arehart, K. H.	2009
Factor structure and reliability of the dutch version of seven scales of the communication profile for the hearing impaired (CPHI)	Mokkink, L. B.; Knol, D. L.; Zekveld, A. A.; Goverts, S. T.; Kramer, S. E.	2009
Processing of fast speech by elderly listeners	Janse, E.	2009
Intelligibility of interrupted sentences at subsegmental levels in young normal-hearing and elderly hearing-impaired listeners	Lee, J. H.; Kewley-Port, D.	2009
Informational masking in young and elderly listeners for speech masked by simultaneous speech and noise	Agus, T. R.; Akeroyd, M. A.; Gatehouse, S.; Warden, D.	2009
Effects of spectral smearing and temporal fine structure degradation on speech masking release	Gnansia, D.; Pean, V.; Meyer, B.; Lorenzi, C.	2009
Effects of electrode separation between speech and noise signals on consonant identification in cochlear implants	Kwon, B. J.	2009
Speech recognition by bilateral cochlear implant users in a cocktail-party setting	Loizou, P. C.; Hu, Y.; Litovsky, R.; Yu, G.; Peters, R.; Lake, J.; Roland, P.	2009

Abnormal processing of temporal fine structure in speech for frequencies where absolute thresholds are normal	Lorenzi, C.; Debruille, L.; Garnier, S.; Fleuriot, P.; Moore, B. C.	2009
Spectral modulation detection and vowel and consonant identifications in cochlear implant listeners	Saoji, A. A.; Litvak, L.; Spahr, A. J.; Eddins, D. A.	2009
Relations between frequency selectivity, temporal fine-structure processing, and speech reception in impaired hearing	Strelcyk, O.; Dau, T.	2009
Speech intelligibility in background noise with ideal binary time-frequency masking	Wang, D.; Kjems, U.; Pedersen, M. S.; Boldt, J. B.; Lunner, T.	2009
A comparison of presentation levels to maximize word recognition scores	Guthrie, L. A.; Mackersie, C. L.	2009
Self-perception of hearing ability as a strong predictor of hearing aid purchase	Palmer, C. V.; Solodar, H. S.; Hurley, W. R.; Byrne, D. C.; Williams, K. O.	2009
Recognition and localization of speech by adult cochlear implant recipients wearing a digital hearing aid in the nonimplanted ear (bimodal hearing)	Potts, L. G.; Skinner, M. W.; Litovsky, R. A.; Strube, M. J.; Kuk, F.	2009
Hearing Aid Outcomes: Effects of Gender and Experience on Patients' Use and Satisfaction	Williams, V. A.; Johnson, C. E.; Danhauer, J. L.	2009
Evaluation of cellular phone technology with digital hearing aid features: effects of encoding and individualized amplification	Mackersie, C. L.; Qi, Y.; Boothroyd, A.; Conrad, N.	2009
Hearing loss and cognitive effort in older adults' report accuracy for verbal materials	Stewart, R.; Wingfield, A.	2009
Subjective and objective evaluation of noise management algorithms	Peeters, H.; Kuk, F.; Lau, C. C.; Keenan, D.	2009
A naturalistic approach to assessing hearing aid candidacy and motivating hearing aid use	Walden, T. C.; Walden, B. E.; Summers, V.; Grant, K. W.	2009
Effects of expansion on consonant recognition and consonant audibility	Brennan, M.; Souza, P.	2009

Hearing in noise test in subjects with conductive hearing loss	Hsieh, D. L.; Lin, K. N.; Ho, J. H.; Liu, T. C.	2009
Cochlear implant surgery in patients more than seventy-nine years old	Eshraghi, A. A.; Rodriguez, M.; Balkany, T. J.; Telischi, F. F.; Angeli, S.; Hodges, A. V.; Adil, E.	2009
Efficacy of the bone-anchored hearing aid for single-sided deafness	Linstrom, C. J.; Silverman, C. A.; Yu, G. P.	2009
Case-Control Analysis of Cochlear Implant Performance in the Elderly	Runge-Samuelson, C.; Baig, H.; Jensen, J.; Wackym, P. A.; Friedland, D.	2009
Case-control analysis of cochlear implant performance in the elderly case-control analysis of cochlear implant performance in the elderly	Runge-Samuelson, C.; Baig, H.; Jensen, J.; Wackym, P. A.; Friedland, D.	2009
Randomized, double blind, placebo controlled trial on the safety and efficacy of continuous intratympanic dexamethasone delivered via a round window catheter for severe to profound sudden idiopathic sensorineural hearing loss after failure of systemic therapy	Plontke, S. K.; Lowenheim, H.; Mertens, J.; Engel, C.; Meisner, C.; Weidner, A.; Zimmermann, R.; Preyer, S.; Koitschev, A.; Zenner, H. P.	2009
Gamma knife radiosurgery for vestibular schwannomas: results of hearing preservation in relation to the cochlear radiation dose	Timmer, F. C.; Hanssens, P. E.; van Haren, A. E.; Mulder, J. J.; Cremers, C. W.; Beynon, A. J.; van Overbeeke, J. J.; Graamans, K.	2009
On the problem of listening while talking	Borg, E.; Gustafsson, D.; Bergkvist, C.; Wikstrom, C.	2009
Visual stimuli can impair auditory processing in cochlear implant users	Champoux, F.; Lepore, F.; Gagne, J. P.; Theoret, H.	2009

Multiple sensory impairment and quality of life	Fischer, M. E.; Cruickshanks, K. J.; Klein, B. E.; Klein, R.; Schubert, C. R.; Wiley, T. L.	2009
Development of a speaker discrimination test for cochlear implant users based on the Oldenburg Logatome corpus	Muhler, R.; Ziese, M.; Rostalski, D.	2009
Management of single-sided deafness with the bone-anchored hearing aid	Yuen, H. W.; Bodmer, D.; Smilsky, K.; Nedzelski, J. M.; Chen, J. M.	2009
Effect of preoperative hearing level on success of stapes surgery	Caylakli, F.; Yavuz, H.; Yilmazer, C.; Yilmaz, I.; Ozluoglu, L. N.	2009
Younger- and older-age adults with unilateral and bilateral cochlear implants: speech and spatial hearing self-ratings and performance	Noble, W.; Tyler, R. S.; Dunn, C. C.; Bhullar, N.	2009
Bone-Anchored Hearing Aid: Patient Satisfaction With the Cordelle Device	Ho, E. C.; Monksfield, P.; Egan, E.; Reid, A.; Proops, D.	2009
Auditory performance after cochlear implantation in late septuagenarians and octogenarians	Williamson, R. A.; Pytynia, K.; Oghalai, J. S.; Vrabec, J. T.	2009
Coupling the Vibrant Soundbridge to cochlea round window: auditory results in patients with mixed hearing loss	Beltrame, A. M.; Martini, A.; Prosser, S.; Giarbini, N.; Streitberger, C.	2009
A predictive model of cochlear implant performance in postlingually deafened adults	Roditi, R. E.; Poissant, S. F.; Bero, E. M.; Lee, D. J.	2009
Asymmetric hearing loss: rule 3,000 for screening vestibular schwannoma	Saliba, I.; Martineau, G.; Chagnon, M.	2009
Age-related use and benefit of the bone-anchored hearing aid compact	de Wolf, M. J.; Leijendeckers, J. M.; Mylanus, E. A.; Hol, M. K.; Snik, A. F.; Cremers, C. W.	2009

Exclusive transcanal surgical approach for Vibrant Soundbridge implantation: surgical and functional results	Bruschini, L.; Forli, F.; Giannarelli, M.; Bruschini, P.; Berrettini, S.	2009
Indication for surgery in otosclerotic patients with unilateral hearing loss	De Seta, E.; Rispoli, G.; Balsamo, G.; Covelli, E.; De Seta, D.; Filipo, R.	2009
European results with totally implantable carina placed on the round window: 2-year follow-up	Martin, C.; Deveze, A.; Richard, C.; Lefebvre, P. P.; Decat, M.; Ibanez, L. G.; Truy, E.; Mom, T.; Lavieille, J. P.; Magnan, J.; Dubreuil, C.; Tringali, S.	2009
Bone-anchored Hearing Aids: correlation between pure-tone thresholds and outcome in three user groups	Pfiffner, F.; Kompis, M.; Stieger, C.	2009
Effect of cochlear implant technology in sequentially bilaterally implanted adults	Budenz, C. L.; Roland Jr, J. T.; Babb, J.; Baxter, P.; Waltzman, S. B.	2009
The BAHA hearing system for hearing-impaired postirradiated nasopharyngeal cancer patients: A new indication	Soo, G.; Tong, M. C. F.; Tsang, W. S. S.; Wong, T. K. C.; Ka-Fai, T.; Sing-Fai, L.; Van Hasselt, C. A.	2009
Frequency compression and its effects in speech recognition.	Costa Spyer Prates, L. P.; da Silva, F. J. F.; Martinelli Iorio, M. C. M.	2009
Aging, hearing acuity, and the attentional costs of effortful listening	Tun, P. A.; McCoy, S.; Wingfield, A.	2009
Audiological findings in patients treated with radio- and concomitant chemotherapy for head and neck tumors	Dell'Aringa, A. H.; Isaac, M. L.; Arruda, G. V.; Esteves, M. C.; Dell'aringa, A. R.; Junior, J. L.; Rodrigues, A. F.	2009

Risk factors for presbycusis in a socio-economic middle-class sample	Sousa, C. S.; Castro Junior, Nd; Larsson, E. J.; Ching, T. H.	2009
Vibrant Soundbridge middle ear implant in mixed hearing loss. Indications, techniques, results	Dumon, T.; Gratacap, B.; Firmin, F.; Vincent, R.; Pialoux, R.; Casse, B.; Firmin, B.	2009
Vibrant Soundbridge for hearing restoration after chronic ear surgery	Streitberger, C.; Perotti, M.; Beltrame, M. A.; Giarbini, N.	2009
Vision and hearing impairments and their associations with falling and loss of instrumental activities in daily living in acute hospitalized older persons in five Nordic hospitals	Grue, E. V.; Ranhoff, A. H.; Noro, A.; Finne-Soveri, H.; Jensdottir, A. B.; Ljunggren, G.; Bucht, G.; Bjornson, L. J.; Jonsen, E.; Schroll, M.; Jonsson, P. V.	2009
Aided speech-identification performance in single-talker competition by older adults with impaired hearing	Humes, L. E.; Coughlin, M.	2009
Cognition and aided speech recognition in noise: specific role for cognitive factors following nine-week experience with adjusted compression settings in hearing aids	Rudner, M.; Foo, C.; Ronnberg, J.; Lunner, T.	2009
Spoken word recognition by eye	Auer, E. T., Jr.	2009
Studies of hearing-aid outcome measures in older adults: A comparison of technologies and an examination of individual differences	Humes, Larry E; Ahlstrom, Jayne B; Bratt, Gene W; Peek, Barbara F	2009
Short-term hearing aid benefit in a large group	Ivory, P. J.; Hendricks, B. L.; Van Vliet, D.; Beyer, C. M.; Abrams, H. B.	2009
User evaluation of a communication system that automatically generates captions to improve telephone communication	Zekveld, A. A.; Kramer, S. E.; Kessens, J. M.; Vlaming, M. S.; Houtgast, T.	2009

Speech recognition and temporal processing in middle-aged women	Helfer, Karen S; Vargo, Megan %J Journal of the American Academy of Audiology	2009
Neural processing during older adults‚Äô comprehension of spoken sentences: age differences in resource allocation and connectivity	Peelle, Jonathan E; Troiani, Vanessa; Wingfield, Arthur; Grossman, Murray %J Cerebral Cortex	2009
The advanced bionics high resolution mode: Stimulation rates up to 5000 pps	Buechner, A.; Frohne-Bchner, C.; Gaertner, L.; Stoever, T.; Battmer, R. D.; Lenarz, T.	2010
Water-soluble coenzyme Q10 formulation (Q-TER()) in the treatment of presbycusis	Salami, A.; Mora, R.; Dellepiane, M.; Manini, G.; Santomauro, V.; Barettini, L.; Guastini, L.	2010
Bilateral vibrant soundbridge implantation: audiologic and subjective benefits in quiet and noisy environments	Garin, P.; Schmerber, S.; Magnan, J.; Truy, E.; Uziel, A.; Triglia, J. M.; Bebear, J. P.; Labassi, S.; Lavieille, J. P.	2010
Temporal fine structure in cochlear implants: preliminary speech perception results in Cantonese-speaking implant users	Schatzer, R.; Krenmayr, A.; Au, D. K.; Kals, M.; Zierhofer, C.	2010
Main peak interleaved sampling (MPIS) strategy: Effect of stimulation rate variations on speech perception in adult cochlear implant recipients using the Digisonic SP cochlear implant	Di Lella, F.; Bacciu, A.; Pasanisi, E.; Vincenti, V.; Guida, M.; Bacciu, S.	2010
Development and applications of alternative methods of segmentation for Mandarin Hearing in Noise Test in normal-hearing listeners and cochlear implant users	Zhang, N.; Liu, S.; Xu, J.; Liu, B.; Qi, B.; Yang, Y.; Kong, Y.; Han, D.	2010
Speech Intelligibility Measurements in Tinnitus Patients with and without Hearing Loss	Wicher, A.; Ozimek, E.; Szymiec, E.	2010

Clinical experience with the active middle ear implant Vibrant Soundbridge in sensorineural hearing loss	Pok, S. M.; Schlogel, M.; Boheim, K.	2010
Do group audiologic rehabilitation activities influence psychosocial outcomes?	Preminger, J. E.; Yoo, J. K.	2010
Benefit from bimodal hearing in a group of prelingually deafened adult cochlear implant users	Berrettini, S.; Passetti, S.; Giannarelli, M.; Forli, F.	2010
Cochlear implantation in late-implanted adults with prelingual deafness	Most, T.; Shrem, H.; Duvdevani, I.	2010
Neuro-otologic manifestations of multiple sclerosis	Peyvandi, A.; Naghibzadeh, B.; Ahmady Roozbahany, N.	2010
Case-control analysis of cochlear implant performance in elderly patients	Friedland, D. R.; Runge-Samuelson, C.; Baig, H.; Jensen, J.	2010
Speech perception after cochlear implantation in 53 patients with otosclerosis: multicentre results	Rotteveel, L. J.; Snik, A. F.; Cooper, H.; Mawman, D. J.; van Olphen, A. F.; Mylanus, E. A.	2010
Vestibular schwannoma and fitness to fly	Pons, Y.; Raynal, M.; Hunkemoller, I.; Lepage, P.; Kossowski, M.	2010
Comparison between new and old generation RetroX auditory implants	Van Damme, J. P.; Jamart, J.; Garin, P.	2010
Relationship between severity of traumatic brain injury (TBI) and extent of auditory dysfunction	Munjal, S. K.; Panda, N. K.; Pathak, A.	2010
Psychometric properties of the international outcome inventory for hearing AIDS	Gasparin, M.; Menegotto, I. H.; Cunha, C. S.	2010
Speech recognition according to the length of hearing aid use	Petry, T.; dos Santos, S. N.; Costa, M. J.	2010
Intratympanic methylprednisolone as rescue therapy in sudden sensorineural hearing loss	Raymundo, I. T.; Bahmad Jr, F.; Barros Filho, J.; Pinheiro, T. G.; Maia, N. A.; Oliveira, C. A.	2010

Does drill-induced noise have an impact on sensorineural hearing during craniotomy procedure?	Farzanegan, G.; Ghasemi, M.; Panahi, F.; Raza, M.; Alghasi, M.	2010
Electric acoustic stimulation of the auditory system: experience and results of ten patients using MED-EL's M and FlexEAS electrodes	Lee, A.; Jiang, D.; McLaren, S.; Nunn, T.; Demler, J. M.; Tysome, J. R.; Connor, S.; Fitzgerald O'Connor, A.	2010
The bone-anchored hearing aid in the rehabilitation of single-sided deafness: experience with 58 patients	Martin, T. P.; Lowther, R.; Cooper, H.; Holder, R. L.; Irving, R. M.; Reid, A. P.; Proops, D. W.	2010
The relationship between patients' perception of the effects of neurofibromatosis type 2 and the domains of the Short Form-36	Neary, W. J.; Hillier, V. F.; Flute, T.; Stephens, S. D. G.; Ramsden, R. T.; Evans, D. G. R.	2010
Coenzyme Q10 in combination with steroid therapy for treatment of sudden sensorineural hearing loss: a controlled prospective study	Ahn, J. H.; Yoo, M. H.; Lee, H. J.; Chung, J. W.; Yoon, T. H.	2010
Simultaneous bilateral cochlear implantation: prospective study in adults	Koch, D. B.; Soli, S. D.; Downing, M.; Osberger, M. J.	2010
Linguistic assessment tools for the Digisonic Dual electric-acoustic speech processor	Heeren, W.; Vaerenberg, B.; Coene, M.; Daemers, K.; Govaerts, P. J.; Avram, A. A.; Cardinaletti, A.; De Ceulaer, G.; Del Bo, L.; Gillis, S.; Pascu, A.; Rooryck, J.; Schauwers, K.; van Heuven, V.; Volpato, F.	2010
Preliminary results of DUET to DUET 2 upgrade	Lorens, A.; Zgoda, M.; Waskiewicz, B.; Lutek, A.; Skarzynski, H.	2010

Fitting of the hearing system affects partial deafness cochlear implant performance	Polak, M.; Lorens, A.; Helbig, S.; McDonald, S.; McDonald, S.; Vermeire, K.	2010
Executive Dysfunction and Presbycusis in Older Persons With and Without Memory Loss and Dementia	Gates, G. A.; Gibbons, L. E.; McCusrry, S. M.; Crane, P. K.; Feeney, M. P.; Larson, E. B.	2010
Evaluation of the CAMEQ2-HF method for fitting hearing aids with multichannel amplitude compression	Moore, B. C.; Fullgrabe, C.	2010
Development of APHAB norms for WDRC hearing aids and comparisons with original norms	Johnson, J. A.; Cox, R. M.; Alexander, G. C.	2010
Impact of visual cues on directional benefit and preference: Part I--laboratory tests	Wu, Y. H.; Bentler, R. A.	2010
Evidence for the expansion of adult cochlear implant candidacy	Gifford, R. H.; Dorman, M. F.; Shallop, J. K.; Sydlowski, S. A.	2010
Cochlear implant-mediated perception of nonlinguistic sounds	Inverso, Y.; Limb, C. J.	2010
Amplification and consonant modulation spectra	Souza, P.; Gallun, F.	2010
Psychoacoustic abilities associated with music perception in cochlear implant users	Won, J. H.; Drennan, W. R.; Kang, R. S.; Rubinstein, J. T.	2010
Neural Response Telemetry Reconsidered: I. The Relevance of ECAP Threshold Profiles and Scaled Profiles to Cochlear Implant Fitting	Botros, A.; Psarros, C.	2010
Advantages of a non-linear frequency compression algorithm in noise	Bohnert, A.; Nyffeler, M.; Keilmann, A.	2010
Pilot study on the effectiveness of the conventional CROS, the transcranial CROS and the BAHA transcranial CROS in adults with unilateral inner ear deafness	Hol, M. K. S.; Kunst, S. J. W.; Snik, A. F. M.; Cremers, Cwrj	2010
Non-verbal visual reinforcement affects speech audiometry in the elderly	Di Berardino, F.; Forti, S.; Mattei, V.; Alpini, D.; Cesarani, A.	2010
Modified intratympanic treatment for idiopathic sudden sensorineural hearing loss	Kara, E.; Cetik, F.; Tarkan, O.; Surmelioglu, O.	2010

Impact of hearing impairment on spousal mental health: the Nord-Trondelag Health Study	Ask, H.; Krog, N. H.; Tambs, K.	2010
Cochlear implantation in elderly patients: surgical and audiological outcome	Migirov, L.; Taitelbaum-Swead, R.; Drendel, M.; Hildesheimer, M.; Kronenberg, J.	2010
Phonemic restoration by hearing-impaired listeners with mild to moderate sensorineural hearing loss	Baskent, D.; Eiler, C. L.; Edwards, B.	2010
Sensitivity of psychophysical measures to signal processor modifications in cochlear implant users	Drennan, W. R.; Won, J. H.; Nie, K.; Jameyson, E.; Rubinstein, J. T.	2010
Intelligibility of interrupted and interleaved speech for normal-hearing listeners and cochlear implantees	Gnansia, D.; Pressnitzer, D.; Pean, V.; Meyer, B.; Lorenzi, C.	2010
Bimodal stimulus presentation and expanded auditory bandwidth improve older adults' speech perception	Brault, L. M.; Gilbert, J. L.; Lansing, C. R.; McCarley, J. S.; Kramer, A. F.	2010
Across-frequency delays based on the cochlear traveling wave: Enhanced speech presentation for cochlear implants	Taft, D. A.; Grayden, D. B.; Burkitt, A. N.	2010
Factors associated with hearing aid fitting outcomes on the IOI-HA	Hickson, L.; Clutterbuck, S.; Khan, A.	2010
Factors influencing rehabilitation decisions of adults with acquired hearing impairment	Laplante-Levesque, A.; Hickson, L.; Worrall, L.	2010
Users' perspectives on the benefits of FM systems with cochlear implants	Fitzpatrick, E. M.; Fournier, P.; Seguin, C.; Armstrong, S.; Chenier, J.; Schramm, D.	2010
Older adults' performance on the speech, spatial, and qualities of hearing scale (SSQ): Test-retest reliability and a comparison of interview and self-administration methods	Singh, G.; Kathleen Pichora-Fuller, M.	2010
Successful and unsuccessful users of bilateral amplification: differences and similarities in binaural performance	Kobler, S.; Lindblad, A. C.; Olofsson, A.; Hagerman, B.	2010

Illness perceptions and hearing difficulties in King-Kopetzky syndrome: What determines help seeking?	Pryce, H.; Metcalfe, C.; Hall, A.; Claire, L. S.	2010
Effectiveness of computer-based auditory training for adult users of cochlear implants	Stacey, P. C.; Raine, C. H.; O'Donoghue, G. M.; Tapper, L.; Twomey, T.; Summerfield, A. Q.	2010
The influence of mood on the perception of hearing-loss related quality of life in people with hearing loss and their significant others	Preminger, J. E.; Meeks, S.	2010
Speech perception performance as a function of stimulus pulse rate and processing strategy preference for the Cochlear Nucleus CI24RE device: relation to perceptual threshold and loudness comfort profiles	Battmer, R. D.; Dillier, N.; Lai, W. K.; Begall, K.; Leypon, E. E.; Gonzalez, J. C.; Manrique, M.; Morera, C.; Muller-Deile, J.; Wesarg, T.; Zarowski, A.; Killian, M. J.; von Wallenberg, E.; Smoorenburg, G. F.	2010
Preliminary evaluation of a method for fitting hearing aids with extended bandwidth	Fllgrabe, C.; Baer, T.; Stone, M. A.; Moore, B. C. J.	2010
The French digit triplet test: a hearing screening tool for speech intelligibility in noise	Jansen, S.; Luts, H.; Wagener, K. C.; Frachet, B.; Wouters, J.	2010
Comparing health care use and related costs between groups with and without hearing impairment	Nachtegaal, J.; Heymans, M. W.; van Tulder, M. W.; Goverts, S. T.; Festen, J. M.; Kramer, S. E.	2010
Neural plasticity in the elderly following hearing aids usage	Lavie, L.; Attias, J.; Karni, A.	2010
Prevalence and characteristics of hearing problems in a working and non-working Swedish population	Hasson, D.; Theorell, T.; Westerlund, H.; Canlon, B.	2010
Enhancement of Speech Intelligibility in Digital Hearing Aids Using Directional Microphone/Noise Reduction Algorithm	Tawfik, S.; El Danasoury, I. M. S.; AbuMoussa, H.; Naguib, Mfnf	2010

Quality of life in patients with untreated age-related hearing loss	Chew, H. S.; Yeak, S.	2010
Use of a closed set questionnaire to measure primary and secondary effects of neurofibromatosis type 2	Neary, W. J.; Hillier, V. F.; Flute, T.; Stephens, D.; Ramsden, R. T.; Evans, D. G. R.	2010
Dynamics of hearing status in closed head injury	Munjal, S. K.; Panda, N. K.; Pathak, A.	2010
Prevalence and degree of hearing loss among males in Beaver Dam cohort: comparison of veterans and nonveterans	Wilson, R. H.; Noe, C. M.; Cruickshanks, K. J.; Wiley, T. L.; Nondahl, D. M.	2010
Improving the quality and applicability of the Dutch scales of the Communication Profile for the Hearing Impaired using item response theory	Mokkink, L. B.; Knol, D. L.; van Nispen, R. M.; Kramer, S. E.	2010
Measuring the Effects of Reverberation and Noise on Sentence Intelligibility for Hearing-Impaired Listeners	George, E. L. J.; Goverts, S. T.; Festen, J. M.; Houtgast, T.	2010
Recognition of accented English in quiet by younger normal-hearing listeners and older listeners with normal-hearing and hearing loss	Gordon-Salant, S.; Yeni-Komshian, G. H.; Fitzgibbons, P. J.	2010
The role of segmentation difficulties in speech-in-speech understanding in older and hearing-impaired adults	Woodfield, A.; Akeroyd, M. A.	2010
Recognition of accented English in quiet and noise by younger and older listeners	Gordon-Salant, S.; Yeni-Komshian, G. H.; Fitzgibbons, P. J.	2010
Phonemic restoration in sensorineural hearing loss does not depend on baseline speech perception scores	Baskent, D.	2010
Revision, extension, and evaluation of a binaural speech intelligibility model	Beutelmann, R.; Brand, T.; Kollmeier, B.	2010
Speech reception by listeners with real and simulated hearing impairment: Effects of continuous and interrupted noise	Desloge, J. G.; Reed, C. M.; Braida, L. D.; Perez, Z. D.; Delhorne, L. A.	2010
Auditory temporal-order processing of vowel sequences by young and elderly listeners	Fogerty, D.; Humes, L. E.; Kewley-Port, D.	2010

Multi-microphone adaptive noise reduction strategies for coordinated stimulation in bilateral cochlear implant devices	Kokkinakis, K.; Loizou, P. C.	2010
Short-term adaptation to accented English by younger and older adults	Gordon-Salant, S.; Yeni-Komshian, G. H.; Fitzgibbons, P. J.	2010
The binaural intelligibility level difference in hearing-impaired listeners: the role of supra-threshold deficits	Goverts, S. T.; Houtgast, T.	2010
Short and long compression release times: speech understanding, real-world preferences, and association with cognitive ability	Cox, R. M.; Xu, J.	2010
Evaluation of an audiological rehabilitation program for spouses of people with hearing loss	Preminger, J. E.; Meeks, S.	2010
Use of Hearing Aids and Assistive Listening Devices in an Older Australian Population	Hartley, D.; Rochtchina, E.; Newall, P.; Golding, M.; Mitchell, P.	2010
Assessing binaural hearing: results using the speech, spatial and qualities of hearing scale	Noble, W.	2010
Effectiveness of the directional microphone in the Baha DivinoTM	Oeding, K.; Valente, M.; Kerckhoff, J.	2010
A clinical protocol for predicting outcomes with an implantable prosthetic device (Baha) in patients with single-sided deafness	Snapp, H. A.; Fabry, D. A.; Telischi, F. F.; Arheart, K. L.; Angeli, S. I.	2010
The Effects of Receiver Placement on Probe Microphone, Performance, and Subjective Measures with Open Canal Hearing Instruments	Alworth, L. N.; Plyler, P. N.; Rebert, M. B.; Johnstone, P. M.	2010
Effect of age on directional microphone hearing aid benefit and preference	Wu, Y. H.	2010
Impact of spectrally asynchronous delays on consonant voicing perception	Ortmann, A. J.; Palmer, C. V.; Pratt, S. R.	2010
Performance over time on adults with simultaneous bilateral cochlear implants	Chang, S. A.; Tyler, R. S.; Dunn, C. C.; Ji, H.; Witt, S. A.; Gantz, B.; Hansen, M.	2010
Benefits of localization and speech perception with multiple noise sources in listeners with a short-electrode cochlear implant	Dunn, C. C.; Perreau, A.; Gantz, B.; Tyler, R. S.	2010

Speech perception for adult cochlear implant recipients in a realistic background noise: effectiveness of preprocessing strategies and external options for improving speech recognition in noise	Gifford, R. H.; Revit, L. J.	2010
Partial deafness cochlear implantation at the University of Kansas: techniques and outcomes	Prentiss, S.; Sykes, K.; Staecker, H.	2010
Word-recognition performance in interrupted noise by young listeners with normal hearing and older listeners with hearing loss	Wilson, R. H.; McArdle, R.; Betancourt, M. B.; Herring, K.; Lipton, T.; Chisolm, T. H.	2010
Intelligibility of foreign-accented speech for older adults with and without hearing loss	Ferguson, S. H.; Jongman, A.; Sereno, J. A.; Keum, K. A.	2010
Hearing impairment affects older people's ability to drive in the presence of distracters	Hickson, L.; Wood, J.; Chaparro, A.; Lacherez, P.; Marszalek, R.	2010
Long-term effectiveness of screening for hearing loss: The screening for auditory impairment - Which hearing assessment test (SAI-WHAT) randomized trial	Yueh, B.; Collins, M. P.; Souza, P. E.; Boyko, E. J.; Loovis, C. F.; Heagerty, P. J.; Liu, C. F.; Hedrick, S. C.	2010
Bone-anchored hearing aid subjective benefit for unilateral deafness	House, J. W.; Kutz Jr, J. W.; Chung, J.; Fisher, L. M.	2010
Audiological outcome of the pull-back technique in cochlear implantees	Basta, D.; Todt, I.; Ernst, A.	2010
Analysis of hearing preservation after endolymphatic mastoid sac surgery for Meniere's disease	Sun, G. H.; Leung, R.; Samy, R. N.; McAfee, J. S.; Hearst, M. J.; Savage, C. R.; Choo, D. I.; Pensak, M. L.	2010
Cochlear implantation in patients with autoimmune inner ear disease including cogan syndrome: a comparison with age- and sex-matched controls	Wang, J. R.; Yuen, H. W.; Shipp, D. B.; Stewart, S.; Lin, V. Y.; Chen, J. M.; Nedzelski, J. M.	2010
Improved speech discrimination after cochlear implantation in the Southern Cochlear Implant Adult Programme	Bradley, J.; Bird, P.; Monteath, P.; Wells, J. E.	2010

Speech perception with cochlear implants as measured using a roving-level adaptive test method	Haumann, S.; Lenarz, T.; Buchner, A.	2010
Intratympanic gentamicin injections for the treatment of Meniere's disease	Katzenell, U.; Gordon, M.; Page, M.	2010
The Baha system in patients with single-sided deafness and contralateral hearing loss	Wazen, J. J.; Van Ess, M. J.; Alameda, J.; Ortega, C.; Modisett, M.; Pinsky, K.	2010
Intratympanic gentamicin treatment of patients with Meniere's disease with normal hearing	Silverstein, H.; Wazen, J.; Van Ess, M. J.; Daugherty, J.; Alameda, Y. A.	2010
Is electric acoustic stimulation better than conventional cochlear implantation for speech perception in quiet?	Adunka, O. F.; Pillsbury, H. C.; Adunka, M. C.; Buchman, C. A.	2010
Cochlear implantation outcomes in patients with autoimmune and immune-mediated inner ear disease	Aftab, S.; Semaan, M. T.; Murray, G. S.; Megerian, C. A.	2010
Cochlear implantation in the octogenarian and nonagenarian	Carlson, M. L.; Breen, J. T.; Gifford, R. H.; Driscoll, C. L.; Neff, B. A.; Beatty, C. W.; Peterson, A. M.; Olund, A. P.	2010
The relationship between electrically evoked compound action potential and speech perception: a study in cochlear implant users with short electrode array	Kim, J. R.; Abbas, P. J.; Brown, C. J.; Etler, C. P.; O'Brien, S.; Kim, L. S.	2010
Long-term hearing preservation in vestibular schwannoma	Stangerup, S. E.; Thomsen, J.; Tos, M.; Caye-Thomasen, P.	2010
Impact of hair cell preservation in cochlear implantation: combined electric and acoustic hearing	Turner, C. W.; Gantz, B. J.; Karsten, S.; Fowler, J.; Reiss, L. A.	2010

Long-term hearing preservation after microsurgical excision of vestibular schwannoma	Woodson, E. A.; Dempewolf, R. D.; Gubbels, S. P.; Porter, A. T.; Oleson, J. J.; Hansen, M. R.; Gantz, B. J.	2010
Results of a pilot study with a signal enhancement algorithm for HiRes 120 cochlear implant users	Buechner, A.; Brendel, M.; Saalfeld, H.; Litvak, L.; Frohne-Buechner, C.; Lenarz, T.	2010
Active middle ear implant compared with open-fit hearing aid in sloping high-frequency sensorineural hearing loss	Boeheim, K.; Pok, S. M.; Schloegel, M.; Filzmoser, P.	2010
How internet telephony could improve communication for hearing-impaired individuals	Mantokoudis, G.; Kompis, M.; Dubach, P.; Caversaccio, M.; Senn, P.	2010
Auditory training: assessment of the benefit of hearing aids in elderly individuals	Megale, R. L.; Iorio, M. C.; Schochat, E.	2010
Acclimatization effect in speech recognition: evaluation without hearing aids	Santos, S. N.; Petry, T.; Costa, M. J.	2010
Evaluation of cochlear nerve size by magnetic resonance imaging in elderly patients with sensorineural hearing loss	Sildiroglu, O.; Cincik, H.; Sonmez, G.; Ozturk, E.; Mutlu, H.; Gocgeldi, E.; Tunca Keskin, A.; Basekim, C.; Kizilkaya, E.	2010
Assessment of the auditory handicap in adults with unilateral hearing loss	Araujo, P. G.; Mondelli, M. F.; Lauris, J. R.; Richieri-Costa, A.; Feniman, M. R.	2010
Speech perception of hearing impaired people using a hearing aid with noise supression algorithms	Oliveira, J. R.; Lopes, E. S.; Alves, A. F.	2010
Auditory results from cochlear implants in elderly people	Oyanguren, V.; Gomes, M. V.; Tsuji, R. K.; Bento, R. F.; Brito Neto, R.	2010

Otosclerosis among patients with dizziness	Eza-Nunez, P.; Manrique-Rodriguez, M.; Perez-Fernandez, N.	2010
"Can you hear me now?" The validation of a self-assessment scale for telephone abilities through structured conversation ratings	Kaplan, H. S.; Holmes, A. E.	2010
The effectiveness of communication strategy training with adult cochlear implantees	Sparrow, K. M.; Hird, K.	2010
The prevalence and correlates of self-reported hearing impairment in the Ibadan Study of Ageing	Lasisi, A. O.; Abiona, T.; Gureje, O.	2010
Exploring the factors influencing discontinued hearing aid use in patients with unilateral cochlear implants	Fitzpatrick, E. M.; Leblanc, S.	2010
Improvements in speech understanding with wireless binaural broadband digital hearing instruments in adults with sensorineural hearing loss	Kreisman, B. M.; Mazevski, A. G.; Schum, D. J.; Sockalingam, R.	2010
Hearing loss in elderly	Yilmaz, O.; Kuran, G.; Vuralkan, E.; Sagit, M.; Elverici, O.; Alicura, S.	2010
A Pilot Investigation Regarding Speech-Recognition Performance in Noise for Adults with Hearing Loss in the FM plus HA Listening Condition	Lewis, M. S.; Gallun, F. J.; Gordon, J.; Lilly, D. J.; Crandell, C.	2010
Response latencies in auditory sentence comprehension: effects of linguistic versus perceptual challenge	Tun, Patricia A; Benichov, Jonathan; Wingfield, Arthur %J Psychology; aging	2010
Bilateral and unilateral cochlear implant users compared on speech perception in noise	Dunn, Camille C; Noble, William; Tyler, Richard S; Kordus, Monika; Gantz, Bruce J; Ji, Haihong %J Ear; hearing	2010
Interrupted speech perception: The effects of hearing sensitivity and frequency resolution	Jin, Su-Hyun; Nelson, Peggy B %J The Journal of the Acoustical Society of America	2010

Comprehension of a novel accent by young and older listeners	Adank, Patti; Janse, Esther %J Psychology; aging	2010
Temporal resolution in regions of normal hearing and speech perception in noise for adults with sloping high-frequency hearing loss	Feng, Yanmei; Yin, Shankai; Kiefte, Michael; Wang, Jian %J Ear; hearing	2010
The patterns and risk factors of hearing loss following microvascular decompression for hemifacial spasm	Jo, K. W.; Kim, J. W.; Kong, D. S.; Hong, S. H.; Park, K.	2011
Speech recognition with BAHA simulator in subjects with acquired unilateral sensorineural hearing loss	Bovo, R.; Prosser, S.; Ortore, R. P.; Martini, A.	2011
Water-soluble coenzyme Q10 formulation in presbycusis: long-term effects	Guastini, L.; Mora, R.; Dellepiane, M.; Santomauro, V.; Giorgio, M.; Salami, A.	2011
Hearing preservation surgery in acoustic neuroma. Slow progress and new strategies	Mazzoni, A.; Biroli, F.; Foresti, C.; Signorelli, A.; Sortino, C.; Zanoletti, E.	2011
Audiological results with Baha in conductive and mixed hearing loss	Pfiffner, F.; Caversaccio, M. D.; Kompis, M.	2011
Development of the screening test for hearing problems	Demorest, M. E.; Wark, D. J.; Erdman, S. A.	2011
Effects of transient noise reduction algorithms on speech intelligibility and ratings of hearing aid users	DiGiovanni, J. J.; Davlin, E. A.; Nagaraj, N. K.	2011
Ear asymmetries and asymmetric directional microphone hearing aid fittings	Cord, M. T.; Surr, R. K.; Walden, B. E.; Dittberner, A. B.	2011
Long-term effects of hearing aids on word recognition scores	Song, J. E.; Tanaka, S. M.; Pinto, J. M.; Rasmussen, B.; Ferro, L. M.; Saadia-Redleaf, M. I.	2011

The prevalence of hearing impairment and associated risk factors: the Beaver Dam Offspring Study	Nash, S. D.; Cruickshanks, K. J.; Klein, R.; Klein, B. E.; Nieto, F. J.; Huang, G. H.; Pankow, J. S.; Tweed, T. S.	2011
Bone anchored hearing aid in single sided deafness: Outcome in right-handed patients	Saliba, I.; Nader, M. E.; Fata, F. E.; Leroux, T.	2011
Low-tone air-bone gaps after endolymphatic sac surgery	Kitahara, T.; Horii, A.; Mishiro, Y.; Kawashima, T.; Imai, T.; Nishiike, S.; Inohara, H.	2011
Quantitative analysis of the effect of the demographic factors on presbyacusis	Dayasiri, M. B. K. C.; Dayasena, R. P.; Jayasuriya, C.; Perera, D. S. C.; Kuruppu, K. A. M. P.; Peris, M. N. P.	2011
Validity and reliability of the hearing handicap inventory for adults	Aiello, C. P.; de Lima,, II; Ferrari, D. V.	2011
Older Adults' Views of their Communication Difficulties and Needs while Driving in a Motor Vehicle	Meston, C. N.; Jennings, M. B.; Cheesman, M. F.	2011
Speech audiometry with non-native English speakers: The use of digits and Cantonese words as stimuli	Marinova-Todd, S. H.; Siu, C. K.; Jenstad, L. M.	2011
Vowel confusion patterns in adults during initial 4 years of implant use	Valimaa, T. T.; Sorri, M. J.; Laitakari, J.; Sivonen, V.; Muhli, A.	2011
Auditory cortical N100 in pre- and post-synaptic auditory neuropathy to frequency or intensity changes of continuous tones	Dimitrijevic, A.; Starr, A.; Bhatt, S.; Michalewski, H. J.; Zeng, F. G.; Pratt, H.	2011
Hearing disability in patients with Fuchs' endothelial corneal dystrophy: Unrecognized co-pathology?	Stehouwer, M.; Bijlsma, W. R.; van der Lelij, A.	2011

Functional benefit of the bone-anchored hearing aid with different auditory profiles: objective and subjective measures	van Wieringen, A.; De Voecht, K.; Bosman, A. J.; Wouters, J.	2011
Spectral cues for understanding speech in quiet and in noise	Spahr, A.; Saoji, A.; Litvak, L.; Dorman, M.	2011
Cochlear implants in adults with prelinguistic deafness: do auditory gains match the subjective benefits?	Millette, I.; Gobeil, S.; Bherer, M.; Duchesne, L.	2011
Preliminary results of a novel enhancement method for high-frequency hearing loss	Arioz, U.; Arda, K.; Tuncel, U.	2011
Determination of preferred parameters for multichannel compression using individually fitted simulated hearing AIDS and paired comparisons	Moore, B. C.; Fullgrabe, C.; Stone, M. A.	2011
Clinical evaluation of signal-to-noise ratio-based noise reduction in Nucleus cochlear implant recipients	Dawson, P. W.; Mauger, S. J.; Hersbach, A. A.	2011
The placebo effect and the influence of participant expectation on hearing aid trials	Dawes, P.; Powell, S.; Munro, K. J.	2011
Determining perceived sound quality in a simulated hearing aid using the international speech test signal	Arehart, K. H.; Kates, J. M.; Anderson, M. C.; Moats, P.	2011
The AMA method of estimation of hearing disability: a validation study	Dobie, R. A.	2011
A neural basis of speech-in-noise perception in older adults	Anderson, S.; Parbery-Clark, A.; Yi, H. G.; Kraus, N.	2011
Perception of environmental sounds by experienced cochlear implant patients	Shafiro, V.; Gygi, B.; Cheng, M. Y.; Vachhani, J.; Mulvey, M.	2011
Listening comprehension across the adult lifespan	Sommers, M. S.; Hale, S.; Myerson, J.; Rose, N.; Tye-Murray, N.; Spehar, B.	2011
Technology-limited and patient-derived versus audibility-derived fittings in bone-anchored hearing aid users: a validation study	Hodgetts, W. E.; Hagler, P.; Hakansson, B. E.; Soli, S. D.	2011
Within-subjects comparison of the HiRes and Fidelity120 speech processing strategies: speech perception and its relation to place-pitch sensitivity	Donaldson, G. S.; Dawson, P. K.; Borden, L. Z.	2011

The perception of sentence stress in cochlear implant recipients	Meister, H.; Landwehr, M.; Pyschny, V.; Wagner, P.; Walger, M.	2011
Relative importance of monaural sound deprivation and bilateral significant hearing loss in predicting cochlear implantation outcomes	Boisvert, I.; McMahon, C. M.; Tremblay, G.; Lyxell, B.	2011
Cross-modal plasticity and speech perception in pre- and postlingually deaf cochlear implant users	Buckley, K. A.; Tobey, E. A.	2011
Cochlear dead regions in typical hearing aid candidates: prevalence and implications for use of high-frequency speech cues	Cox, R. M.; Alexander, G. C.; Johnson, J.; Rivera, I.	2011
Effects of degree and configuration of hearing loss on the contribution of high- and low-frequency speech information to bilateral speech understanding	Hornsby, B. W.; Johnson, E. E.; Picou, E.	2011
Digit training in noise can improve cochlear implant users' speech understanding in noise	Oba, S. I.; Fu, Q. J.; Galvin, J. J., 3rd	2011
Comparison of wireless and acoustic hearing aid-based telephone listening strategies	Picou, E. M.; Ricketts, T. A.	2011
Speech intelligibility as a predictor of cochlear implant outcome in prelingually deafened adults	van Dijkhuizen, J. N.; Beers, M.; Boermans, P. P.; Briaire, J. J.; Frijns, J. H.	2011
Benefits of low-frequency attenuation of baha in single-sided sensorineural deafness	Pfiffner, F.; Kompis, M.; Flynn, M.; Asnes, K.; Arnold, A.; Stieger, C.	2011
Contribution of spectral cues to mandarin lexical tone recognition in normal-hearing and hearing-impaired Mandarin Chinese speakers	Wang, S.; Mannell, R.; Newall, P.; Han, D.	2011
Preference for One or Two Hearing Aids Among Adult Patients	Cox, R. M.; Schwartz, K. S.; Noe, C. M.; Alexander, G. C.	2011
Audiovisual asynchrony detection and speech intelligibility in noise with moderate to severe sensorineural hearing impairment	Baskent, D.; Bazo, D.	2011
Functional outcomes in retrosigmoid approach microsurgery and gamma knife stereotactic radiosurgery in vestibular schwannoma	Park, C. E.; Park, B. J.; Lim, Y. J.; Yeo, S. G.	2011

Central auditory processing in elderly with mild cognitive impairment	Rahman, T. T. A.; Mohamed, S. T.; Albanouby, M. H.; Bekhet, H. F.	2011
General and disease-specific quality of life in patients with chronic suppurative otitis media - a prospective study	Baumann, I.; Gerendas, B.; Plinkert, P. K.; Praetorius, M.	2011
Mismatch negativity and adaptation measures of the late auditory evoked potential in cochlear implant users	Zhang, F.; Hammer, T.; Banks, H. L.; Benson, C.; Xiang, J.; Fu, Q. J.	2011
Voice discrimination in cochlear-implanted deaf subjects	Massida, Z.; Belin, P.; James, C.; Rouger, J.; Fraysse, B.; Barone, P.; Deguine, O.	2011
Variable degrees of hearing impairment in a Dutch DFNX4 (DFN6) family	Weegerink, N. J. D.; Huygen, P. L. M.; Schraders, M.; Kremer, H.; Pennings, R. J. E.; Kunst, H. P. M.	2011
Indication for and verification of hearing aid benefit using modern methods of speech audiometry in German. [German, English]	Kollmeier, B.; Lenarz, T.; Winkler, A.; Zokoll, M. A.; Sukowski, H.; Brand, T.; Wagener, K. C.	2011
Conductive hearing loss and bone conduction devices: Restored binaural hearing?	Agterberg, M. J. H.; Hol, M. K. S.; Cremers, C. W. R. J.; Mylanus, E. A. M.; Van Opstal, J.; Snik, A. F. M.	2011
Development of the Listening Self-Efficacy Questionnaire (LSEQ)	Smith, S. L.; Pichora-Fuller, K. M.; Watts, K. L.; La More, C.	2011
Voice gender discrimination provides a measure of more than pitch-related perception in cochlear implant users	Li, T.; Fu, Q. J.	2011
Effects of stimulation rate on modulation detection and speech recognition by cochlear implant users	Arora, K.; Vandali, A.; Dowell, R.; Dawson, P.	2011

Tailoring auditory training to patient needs with single and multiple talkers: transfer-appropriate gains on a four-choice discrimination test	Barcroft, J.; Sommers, M. S.; Tye-Murray, N.; Mauze, E.; Schroy, C.; Spehar, B.	2011
Optimizing the perception of soft speech and speech in noise with the Advanced Bionics cochlear implant system	Holden, L. K.; Reeder, R. M.; Firszt, J. B.; Finley, C. C.	2011
Auditory steady state responses recorded in multitalker babble	Leigh-Paffenroth, E. D.; Murnane, O. D.	2011
A study of recorded versus live voice word recognition	Mendel, L. L.; Owen, S. R.	2011
Clinical evaluation of a new hearing aid anti-cardioid directivity pattern	Mueller, H. G.; Weber, J.; Bellanova, M.	2011
Measuring spectral and temporal resolution simultaneously: A comparison between two tests	Van Esch, T. E. M.; Dreschler, W. A.	2011
The speech intelligibility benefit of a unilateral wireless system for hearing-impaired adults	Whitmer, W. M.; Brennan-Jones, C. G.; Akeroyd, M. A.	2011
Prospective effects of hearing status on loneliness and depression in older persons: Identification of subgroups	Pronk, M.; Deeg, D. J. H.; Smits, C.; Van Tilburg, T. G.; Kuik, D. J.; Festen, J. M.; Kramer, S. E.	2011
Comparison of two bone anchored hearing instruments: BP100 and Ponto Pro	Olsen, S. O.; Glad, H.; Nielsen, L. H.	2011
International outcome inventory for hearing aids (IOI-HA): results from the Chinese version	Liu, H.; Zhang, H.; Liu, S.; Chen, X.; Han, D.; Zhang, L.	2011
Perception of temporally modified speech in auditory neuropathy	Hassan, D. M.	2011
How does linguistic complexity influence intelligibility in a German audiometric sentence intelligibility test?	Uslar, V.; Ruigendijk, E.; Hamann, C.; Brand, T.; Kollmeier, B.	2011
Comparison of fluctuating maskers for speech recognition tests	Francart, T.; van Wieringen, A.; Wouters, J.	2011
Speech-in-noise screening tests by internet, part 1: test evaluation for noise-induced hearing loss identification	Leensen, M. C.; de Laat, J. A.; Dreschler, W. A.	2011

Speech-in-noise screening tests by internet, part 2: improving test sensitivity for noise-induced hearing loss	Leensen, M. C.; de Laat, J. A.; Snik, A. F.; Dreschler, W. A.	2011
Comparison of the fine structure processing (FSP) strategy and the CIS strategy used in the MED-EL cochlear implant system: speech intelligibility and music sound quality	Magnusson, L.	2011
Speech understanding in noise with an eyeglass hearing aid: asymmetric fitting and the head shadow benefit of anterior microphones	Mens, L. H.	2011
The Danish hearing in noise test	Nielsen, J. B.; Dau, T.	2011
Speech perception abilities for spectrally modified signals in individuals with auditory dys-synchrony	Prabhu, P.; Avilala, V.; Barman, A.	2011
Otological diagnoses and probable age-related auditory neuropathy in "younger" and "older" elderly persons	Rosenhall, U.; Hederstierna, C.; Idrizbegovic, E.	2011
The use of fundamental frequency information for speech recognition via bimodal stimulation: Cochlear implant on one ear and hearing aid on the other	Shpak, T.; Most, T.; Luntz, M.	2011
Quality of life for individuals with hearing impairment who have not consulted for services and their significant others: Same- and different-sex couples	Kelly, R. J.; Atcherson, S. R.	2011
Quality of life improvement for bone-anchored hearing aid users and their partners	McNeil, M. L.; Gulliver, M.; Morris, D. P.; Bance, M.	2011
Clinical characteristics of acoustic trauma caused by gunshot noise in mass rifle drills without ear protection	Moon, I. S.; Park, S. Y.; Park, H. J.; Yang, H. S.; Hong, S. J.; Lee, W. S.	2011
The roles of bottom-up and top-down information in the recognition of reduced speech: Evidence from listeners with normal and impaired hearing	Janse, E.; Ernestus, M.	2011
Auriculo-vestibular symptoms related to structural and functional disorders of stomatognatic system	Pihut, M.; Majewski, P.; Wisniewska, G.; Reron, E.	2011
Predictors of rehabilitation intervention decisions in adults with acquired hearing impairment	Laplante-Levesque, A.; Hickson, L.; Worrall, L.	2011
Evaluation of a telephone speech-enhancement algorithm among older adults with hearing loss	Roup, C. M.; Poling, G. L.; Harhager, K.; Krishnamurthy, A.; Feth, L. L.	2011

Hearing loss is negatively related to episodic and semantic long-term memory but not to short-term memory	Ronnberg, J.; Danielsson, H.; Rudner, M.; Arlinger, S.; Sternang, O.; Wahlin, A.; Nilsson, L. G.	2011
Perception of suprasegmental speech features via bimodal stimulation: cochlear implant on one ear and hearing aid on the other	Most, T.; Harel, T.; Shpak, T.; Luntz, M.	2011
Bimodal hearing and speech perception with a competing talker	Pyschny, V.; Landwehr, M.; Hahn, M.; Walger, M.; von Wedel, H.; Meister, H.	2011
The effect of hearing impairment on localization dominance for single-word stimuli	Akeroyd, M. A.; Guy, F. H.	2011
Comparing spatial tuning curves, spectral ripple resolution, and speech perception in cochlear implant users	Anderson, E. S.; Nelson, D. A.; Kreft, H.; Nelson, P. B.; Oxenham, A. J.	2011
Auditory filter shapes and high-frequency hearing in adults who have impaired speech in noise performance despite clinically normal audiograms	Badri, R.; Siegel, J. H.; Wright, B. A.	2011
Spatial release from masking in normally hearing and hearing-impaired listeners as a function of the temporal overlap of competing talkers	Best, V.; Mason, C. R.; Kidd, G., Jr.	2011
Fundamental frequency is critical to speech perception in noise in combined acoustic and electric hearing	Carroll, J.; Tiaden, S.; Zeng, F. G.	2011
The effects of age and cochlear hearing loss on temporal fine structure sensitivity, frequency selectivity, and speech reception in noise	Hopkins, K.; Moore, B. C.	2011
Spatial tuning curves from apical, middle, and basal electrodes in cochlear implant users	Nelson, D. A.; Kreft, H. A.; Anderson, E. S.; Donaldson, G. S.	2011
Statistical bias in the assessment of binaural benefit relative to the better ear	van Hoesel, R. J. M.; Litovsky, R. Y.	2011
Acoustic temporal modulation detection and speech perception in cochlear implant listeners	Won, J. H.; Drennan, W. R.; Nie, K.; Jameyson, E. M.; Rubinstein, J. T.	2011

Can basic auditory and cognitive measures predict hearing-impaired listeners' localization and spatial speech recognition abilities?	Neher, T.; Laugesen, S.; Jensen, N. S.; Kragelund, L.	2011
Effects of speaking style on speech intelligibility for Mandarin-speaking cochlear implant users	Li, Y.; Zhang, G.; Kang, H. Y.; Liu, S.; Han, D.; Fu, Q. J.	2011
Use of intonation contours for speech recognition in noise by cochlear implant recipients	Meister, H.; Landwehr, M.; Pyschny, V.; Grugel, L.; Walger, M.	2011
Transitioning hearing aid users with severe and profound loss to a new gain/frequency response: benefit, perception, and acceptance	Convery, E.; Keidser, G.	2011
Postlingually deaf adults of all ages derive equal benefits from unilateral multichannel cochlear implant	Park, E.; Shipp, D. B.; Chen, J. M.; Nedzelski, J. M.; Lin, V. Y.	2011
A randomized, controlled trial of the short-term effects of complementing an educational program for hearing aid users with telephone consultations	Lundberg, M.; Andersson, G.; Lunner, T.	2011
Evaluation of different signal processing options in unilateral and bilateral cochlear freedom implant recipients using R-Space background noise	Brockmeyer, A. M.; Potts, L. G.	2011
Effect of maximum power output and noise reduction on speech recognition in noise	Kuk, F.; Peeters, H.; Lau, C.; Korhonen, P.	2011
Behavioral and electrophysiologic binaural processing in persons with symmetric hearing loss	Leigh-Paffenroth, E. D.; Roup, C. M.; Noe, C. M.	2011
Clinical experience with the words-in-noise test on 3430 veterans: comparisons with pure-tone thresholds and word recognition in quiet	Wilson, R. H.	2011
Some observations on the nature of the audiometric 4000 hz notch: data from 3430 veterans	Wilson, R. H.	2011
Self-reported hearing problems among older adults: prevalence and comparison to measured hearing impairment	Hannula, S.; Bloigu, R.; Majamaa, K.; Sorri, M.; Maki-Torkko, E.	2011
Rehabilitative online education versus internet discussion group for hearing aid users: A randomized controlled trial	Thoren, E.; Svensson, M.; Tornqvist, A.; Andersson, G.; Carlbring, P.; Lunner, T.	2011

Microphone directionality, pre-emphasis filter, and wind noise in cochlear implants	Chung, K.; McKibben, N.	2011
Working memory supports listening in noise for persons with hearing impairment	Rudner, M.; Ronnberg, J.; Lunner, T.	2011
Evaluation of auditory functions for Royal Canadian Mounted Police officers	Vaillancourt, V.; Laroche, C.; Giguere, C.; Beaulieu, M. A.; Legault, J. P.	2011
The effects of cochlear implantation on speech perception in older adults	Budenz, C. L.; Cosetti, M. K.; Coelho, D. H.; Birenbaum, B.; Babb, J.; Waltzman, S. B.; Roehm, P. C.	2011
Therapy of idiopathic sudden sensorineural hearing loss with intratympanic steroid injection	Khaimook, W.; Jantarapattana, K.	2011
Cochlear implantation has a positive influence on quality of life, tinnitus, and psychological comorbidity	Olze, H.; Szczepek, A. J.; Haupt, H.; Forster, U.; Zirke, N.; Grabel, S.; Mazurek, B.	2011
Effects of stimulation rate on speech perception in elderly cochlear implant users	Zhang, H.; Runge-Samuelson, C.; Friedland, D. R.	2011
Better performance with bone-anchored hearing aid than acoustic devices in patients with severe air-bone gap	De Wolf, M. J. F.; Hendrix, S.; Cremers, C. W. R. J.; Snik, A. F. M.	2011
Effect of technological advances on cochlear implant performance in adults	Lenarz, M.; Joseph, G.; Sonmez, H.; Buchner, A.; Lenarz, T.	2011
Natural history of hearing deterioration in intracanalicular vestibular schwannoma	Pennings, R. J.; Morris, D. P.; Clarke, L.; Allen, S.; Walling, S.; Bance, M. L.	2011
Quality-of-life outcomes for adult cochlear implant recipients in New Zealand	Looi, V.; Mackenzie, M.; Bird, P.	2011
Hearing speech in music	Ekstrom, S. R.; Borg, E.	2011

Assessment of the subjective benefit of electric acoustic stimulation with the abbreviated profile of hearing aid benefit	Gstoettner, W. K.; Van De Heyning, P.; Fitzgerald O'Connor, A.; Kiefer, J.; Morera, C.; Sainz, M.; Vermeire, K.; McDonald, S.; Cavalle, L.; Garcia Valdecasas, J.; Adunka, O. F.; Baumann, U.; Kleine-Punte, A.; Brockmeier, H.; Anderson, I.; Helbig, S.	2011
Early transtympanic steroid injection in patients with 'poor prognosis' idiopathic sensorineural sudden hearing loss	Zhou, Y.; Zheng, H.; Zhang, Q.; Campione, P. A.	2011
Envoy Esteem Totally Implantable Hearing System: phase 2 trial, 1-year hearing results	Kraus, E. M.; Shohet, J. A.; Catalano, P. J.	2011
Impact of floating mass transducer coupling and positioning in round window vibroplasty	Rajan, G. P.; Lampacher, P.; Ambett, R.; Dittrich, G.; Kuthubutheen, J.; Wood, B.; McArthur, A.; Marino, R.	2011
Long-term safety and benefit of a new intraoral device for single-sided deafness	Murray, M.; Miller, R.; Hujoel, P.; Popelka, G. R.	2011
Cochlear implantation in patients with bilateral severe sensorineural hearing loss after major blunt head trauma	Greenberg, S. L.; Shipp, D.; Lin, V. Y.; Chen, J. M.; Nedzelski, J. M.	2011
Audiometric Outcomes for Acoustic Neuroma Patients After Single Versus Multiple Fraction Stereotactic Irradiation	McWilliams, W.; Trombetta, M.; Werts, E. D.; Fuhrer, R.; Hillman, T.	2011
Superficial siderosis of the central nervous system: phenotype and implications for audiology and otology	Sydlowski, S. A.; Cevette, M. J.; Shallop, J.	2011

Assessment of Electrode Placement and Audiological Outcomes in Bilateral Cochlear Implantation	Wanna, G. B.; Noble, J. H.; McRackan, T. R.; Dawant, B. M.; Dietrich, M. S.; Watkins, L. D.; Rivas, A.; Schuman, T. A.; Labadie, R. F.	2011
Active middle ear implantation in elderly people: a retrospective study	Wolf-Magele, A.; Schnabl, J.; Woellner, T.; Koci, V.; Riechelmann, H.; Sprinzl, G. M.	2011
Comparisons of sound processors based on osseointegrated implants in patients with conductive or mixed hearing loss	Pfiffner, F.; Caversaccio, M. D.; Kompis, M.	2011
Hearing preservation after complete cochlear coverage in cochlear implantation with the free-fitting FLEXSOFT electrode carrier	Helbig, S.; Baumann, U.; Hey, C.; Helbig, M.	2011
Envelope versus fine structure speech coding strategy: a crossover study	Riss, D.; Hamzavi, J. S.; Selberherr, A.; Kaider, A.; Blineder, M.; Starlinger, V.; Gstoettner, W.; Arnoldner, C.	2011
Efficacy and safety of an in-the-mouth bone conduction device for single-sided deafness	Murray, M.; Popelka, G. R.; Miller, R.	2011
Elucidating the effects of ageing on remembering perceptually distorted word pairs	Heinrich, A.; Schneider, B. A.	2011
Quality of life and participation restrictions, a study in elderly	Magalhaes, R.; Iorio, M. C.	2011
Cultural adaptation of the SADL (satisfaction with amplification in daily life) questionaire for Brazilian Portuguese	Mondelli, M. F.; Magalhaes, F. F.; Lauris, J. R.	2011
Hearing organ disorders in patients with systemic sclerosis	Maciaszczyk, K.; Waszczykowska, E.; Pajor, A.; Bartkowiak-Dziankowska, B.; Durko, T.	2011

Hearing loss in diffuse cutaneous systemic scleroderma	Monteiro, T. A.; Christmann, R. B.; Bonfa, E.; Bento, R. F.; Novalo-Goto, E. S.; Vasconcelos, L. G.	2011
Consonant perception by adults with bimodal fitting	Incerti, P. V.; Ching, T. Y. C.; Hill, A.	2011
The patient journey of adults with hearing impairment: the patients‚Äô views	Manchaiah, Vinaya KC; Stephens, Dafydd; Meredith, Rhys %J Clinical Otolaryngology	2011
Is cognitive function in adults with hearing impairment improved by the use of hearing aids?	Choi, A Young; Shim, Hyun Joon; Lee, Sung Hee; Yoon, Sang Won; Joo, Eun-Jeong %J Clinical; experimental otorhinolaryngology	2011
Understanding of spoken language under challenging listening conditions in younger and older listeners: a combined behavioral and electrophysiological study	Getzmann, Stephan; Falkenstein, Michael %J Brain research	2011
Round window vibroplasty: long-term results	Boheim, K.; Mlynski, R.; Lenarz, T.; Schlogel, M.; Hagen, R.	2012
Contralateral hearing aid use in cochlear implanted patients: multicenter study of bimodal benefit	Morera, C.; Cavalle, L.; Manrique, M.; Huarte, A.; Angel, R.; Osorio, A.; Garcia-Ibanez, L.; Estrada, E.; Morera-Ballester, C.	2012

Benefits of the HiRes 120 coding strategy combined with the Harmony processor in an adult European multicentre study	Buchner, A.; Lenarz, T.; Boermans, P. P.; Frijns, J. H.; Mancini, P.; Filipo, R.; Fielden, C.; Cooper, H.; Eklof, M.; Freijd, A.; Lombaard, S.; Meerton, L.; Pickerill, M.; Vanat, Z.; Wesarg, T.; Aschendorff, A.; Kienast, B.; Boyle, P.; Arnold, L.; Meyer, B.; Sterkers, O.; Muller-Deile, J.; Ambrosch, P.; Helbig, S.; Frachet, B.; Gallego, S.; Truy, E.; Jeffs, E.; Morant, A.; Marco, J.	2012
Evolution of otosclerosis to cochlear implantation	Munoz-Fernandez, N.; Morant-Ventura, A.; Achiques, M. T.; Dualde-Beltran, D.; Garcia-Callejo, F. J.; Monrroy-Parada, M. V.; Pitarch, I.; Latorre, E.; Marco-Algarra, J.	2012
Cochleovestibular dysfunction in patients with diabetes mellitus, hypertension and dyslipidemia	Chavez-Delgado, M. E.; Vazquez-Granados, I.; Rosales-Cortes, M.; Velasco-Rodriguez, V.	2012
A comparison between the first-fit settings of two multichannel digital signal-processing strategies: music quality ratings and speech-in-noise scores	Higgins, P.; Searchfield, G.; Coad, G.	2012

Effect of rate-alteration on speech perception in noise in older adults with normal hearing and hearing impairment	Adams, E. M.; Gordon-Hickey, S.; Morlas, H.; Moore, R.	2012
Sequential bilateral cochlear implantation: speech perception and localization pre- and post-second cochlear implantation	Dunn, C. C.; Tyler, R. S.; Witt, S.; Ji, H.; Gantz, B. J.	2012
Analgesic Use and the Risk of Hearing Loss in Women	Curhan, S. G.; Shargorodsky, J.; Eavey, R.; Curhan, G. C.	2012
Cochlear implantation outcomes in patients with far advanced otosclerosis	Semaan, M. T.; Gehani, N. C.; Tummala, N.; Coughlan, C.; Fares, S. A.; Hsu, D. P.; Murray, G. S.; Lippy, W. H.; Megerian, C. A.	2012
Short-term audiologic effect of intratympanic gadolinium contrast agent application in patients with Meniere's disease	Louza, J. P. R.; Flatz, W.; Krause, E.; Gurkov, R.	2012
VTMR, a new speech audiometry test with verbal tasks and motor responses	Di Berardino, F.; Forti, S.; Cesarani, A.	2012
Self-reported hearing performance among subjects with unilateral sensorineural hearing loss	Olsen, S. O.; Hernvig, L. H.; Nielsen, L. H.	2012
Esteem 2 middle ear implant: our experience	Gerard, J. M.; Thill, M. P.; Chantrain, G.; Gersdorff, M.; Deggouj, N.	2012
Intratympanic steroid therapy using the Silverstein MicrowickTM for refractory sudden sensorineural hearing loss increases speech intelligibility	Barriat, S.; van Wijck, F.; Staecker, H.; Lefebvre, P. P.	2012
Improvement in word recognition score with level is associated with hearing aid ownership among patients with hearing loss	Halpin, C.; Rauch, S. D.	2012
Partial deafness treatment with the nucleus straight research array cochlear implant	Skarzynski, H.; Lorens, A.; Matusiak, M.; Porowski, M.; Skarzynski, P. H.; James, C. J.	2012

Speech Performance and Sound Localization Abilities in Neurelec Digisonic (R) SP Binaural Cochlear Implant Users	Verhaert, N.; Lazard, D. S.; Gnansia, D.; Bebear, J. P.; Romanet, P.; Meyer, B.; Pean, V.; Mollard, D.; Truy, E.	2012
Indication criteria for cochlear implants and hearing aids: impact of audiological and non-audiological findings	Haumann, S.; Hohmann, V.; Meis, M.; Herzke, T.; Lenarz, T.; Buchner, A.	2012
Hearing aids reduce overestimation in pre-fitting self-assessment	Nishimura, T.; Uratani, Y.; Fukuda, F.; Saito, O.; Hosoi, H.	2012
Quantifying the bystander-effect of 2.5G mobile telephones on the speech perception of digital hearing aid users	Vlastarakos, P. V.; Nikolopoulos, T. P.; Manolopoulos, L.; Stamou, A.; Halkiotis, K. K.; Ferekidis, E.; Georgiou, E.	2012
Visual activation of auditory cortex reflects maladaptive plasticity in cochlear implant users	Sandmann, P.; Dillier, N.; Eichele, T.; Meyer, M.; Kegel, A.; Pascual-Marqui, R. D.; Marcar, V. L.; Jancke, L.; Debener, S.	2012
Satisfaction of patients fit with a hearing aid in a high complexity clinic	Aurelio, F. S.; da Silva, S. P.; Rodrigues, L. B.; Kuniyoshi, I. C.; Botelho, M. S. N.	2012
Quality of life in elderly adults before and after hearing aid fitting	Mondelli, Mfcg; de Souza, P. J. S.	2012
Result on speech perception after conversion from Spectra to Freedom	de Matos Magalhaes, A. T.; Goffi-Gomez, M. V. S.; Hoshino, A. C.; Tsuji, R. K.; Bento, R. F.; Brito, R.	2012

Hearing aid fitting and unilateral auditory deprivation: Behavioral and electrophysiologic assessment	Wieselberg, M. B.; Iorio, M. C. M.	2012
Hearing preservation in patients with unilateral vestibular schwannoma who undergo stereotactic radiosurgery: Reinterpretation of the auditory brainstem response	Han, J. H.; Kim, D. G.; Chung, H. T.; Paek, S. H.; Park, C. K.; Kim, C. Y.; Kim, Y. H.; Kim, J. W.; Kim, Y. H.; Song, S. W.; Kim, I. K.; Jung, H. W.	2012
Music Perception Ability of Korean Adult Cochlear Implant Listeners	Kim, E.; Lee, H. J.; Kim, H. J.	2012
Evaluation of the ClearVoice Strategy in Adults Using HiResolution Fidelity 120 Sound Processing	Kam, A. C. S.; Ng, I. H. Y.; Cheng, M. M. Y.; Wong, T. K. C.; Tong, M. C. F.	2012
Functional and structural evaluation of hearing in acromegaly	Aydin, K.; Ozturk, B.; Turkyilmaz, M. D.; Dagdelen, S.; Ozgen, B.; Unal, F.; Erbas, T.	2012
Hearing outcomes following microvascular decompression for hemifacial spasm	Shah, A.; Nikonow, T.; Thirumala, P.; Hirsch, B.; Chang, Y.; Gardner, P.; Balzer, J.; Habeych, M.; Crammond, D.; Burkhart, L.; Horowitz, M.	2012
Hearing and cochlear function of patients with ankylosing spondylitis	Kahveci, O. K.; Demirdal, U. S.; Duran, A.; Altuntas, A.; Kavuncu, V.; Okur, E.	2012
Adaptation of the Amsterdam Inventory for Auditory Disability and Handicap into Spanish	Fuente, A.; McPherson, B.; Kramer, S. E.; Hormazabal, X.; Hickson, L.	2012
Quality of life in older Chinese-speaking adults with hearing impairment	Wong, L. L. N.; Cheng, L. K.	2012
Combining directional microphone and single-channel noise reduction algorithms: a clinical evaluation in difficult listening conditions with cochlear implant users	Hersbach, A. A.; Arora, K.; Mauger, S. J.; Dawson, P. W.	2012

Factors associated with third-party disability in spouses of older people with hearing impairment	Scarinci, N.; Worrall, L.; Hickson, L.	2012
What makes adults with hearing impairment take up hearing AIDS or communication programs and achieve successful outcomes?	Laplante-Levesque, A.; Hickson, L.; Worrall, L.	2012
Implications of high-frequency cochlear dead regions for fitting hearing aids to adults with mild to moderately severe hearing loss	Cox, R. M.; Johnson, J. A.; Alexander, G. C.	2012
Cochlear implantation in adults with asymmetric hearing loss	Firszt, J. B.; Holden, L. K.; Reeder, R. M.; Cowdrey, L.; King, S.	2012
Feasibility of ecological momentary assessment of hearing difficulties encountered by hearing aid users	Galvez, G.; Turbin, M. B.; Thielman, E. J.; Istvan, J. A.; Andrews, J. A.; Henry, J. A.	2012
Sound localization in noise by normal-hearing listeners and cochlear implant users	Kerber, S.; Seeber, B. U.	2012
Development and validation of the AzBio sentence lists	Spahr, A. J.; Dorman, M. F.; Litvak, L. M.; Van Wie, S.; Gifford, R. H.; Loizou, P. C.; Loiselle, L. M.; Oakes, T.; Cook, S.	2012
The influence of audiovisual ceiling performance on the relationship between reverberation and directional benefit: perception and prediction	Wu, Y. H.; Bentler, R. A.	2012
Hearing disability measured by the speech, spatial, and qualities of hearing scale in clinically normal-hearing and hearing-impaired middle-aged persons, and disability screening by means of a reduced SSQ (the SSQ5)	Demeester, K.; Topsakal, V.; Hendrickx, J. J.; Fransen, E.; van Laer, L.; Van Camp, G.; Van de Heyning, P.; van Wieringen, A.	2012
Effects of pulse width, pulse rate and paired electrode stimulation on psychophysical measures of dynamic range and speech recognition in cochlear implants	Bonnet, R. M.; Boermans, P. P.; Avenarius, O. F.; Briaire, J. J.; Frijns, J. H.	2012

Effect of speech material on the benefit of temporal fine structure information in speech for young normal-hearing and older hearing-impaired participants	Lunner, T.; Hietkamp, R. K.; Andersen, M. R.; Hopkins, K.; Moore, B. C.	2012
Hearing ability in working life and its relationship with sick leave and self-reported work productivity	Nachtegaal, J.; Festen, J. M.; Kramer, S. E.	2012
Stage II vestibular schwannoma: predictive factors for postoperative hearing loss and facial palsy	Milhe de Saint Victor, S.; Bonnard, D.; Darrouzet, V.; Bellec, O.; Franco-Vidal, V.	2012
Characteristics and determinants of music appreciation in adult CI users	Philips, B.; Vinck, B.; De Vel, E.; Maes, L.; D'Haenens, W.; Keppler, H.; Dhooge, I.	2012
Clinical need for a Baha trial in patients with single-sided sensorineural deafness. Analysis of a Baha database of 196 patients	Desmet, J.; Bouzegta, R.; Hofkens, A.; De Backer, A.; Lambrechts, P.; Wouters, K.; Claes, J.; De Bodt, M.; Van de Heyning, P.	2012
Auditory maturity and hearing performance in inner ear malformations: a histological and electrical stimulation approach	Sainz, M.; Garcia-Valdecasas, J.; Fernandez, E.; Pascual, M. T.; Roda, O.	2012
Noise reduction technologies implemented in head-worn preprocessors for improving cochlear implant performance in reverberant noise fields	Chung, K.; Nelson, L.; Teske, M.	2012
Undirected head movements of listeners with asymmetrical hearing impairment during a speech-in-noise task	Brimijoin, W. O.; McShefferty, D.; Akeroyd, M. A.	2012
Abnormal speech processing in frequency regions where absolute thresholds are normal for listeners with high-frequency hearing loss	Leger, A. C.; Moore, B. C.; Lorenzi, C.	2012

GRM7 variants associated with age-related hearing loss based on auditory perception	Newman, D. L.; Fisher, L. M.; Ohmen, J.; Parody, R.; Fong, C. T.; Frisina, S. T.; Mapes, F.; Eddins, D. A.; Robert Frisina, D.; Frisina, R. D.; Friedman, R. A.	2012
Hearing-aid counseling: comparison of single-session informational counseling with single-session performance-perceptual counseling	Saunders, G. H.; Forsline, A.	2012
Comparing response options for the International Outcome Inventory for Hearing Aids (IOI-HA) and for Alternative Interventions (IOI-AI) daily-use items	Laplante-Levesque, A.; Hickson, L.; Worrall, L.	2012
Validity of the National Acoustic Laboratories procedure for determining percentage loss of hearing	Macrae, J. H.	2012
Self-assessed hearing abilities in middle- and older-age adults: A stratified sampling approach	Noble, W.; Naylor, G.; Bhullar, N.; Akeroyd, M. A.	2012
Speech based optimization of cochlear implants	Holmes, A. E.; Shrivastav, R.; Krause, L.; Siburt, H. W.; Schwartz, E.	2012
The combined effects of reverberation and noise on speech intelligibility by cochlear implant listeners	Hazrati, O.; Loizou, P. C.	2012
Hearing impairment and self-masking: listening during vocalization	Borg, E.; Wikstrom, C.; Gustafsson, D.	2012
Binaural dichotic presentation to reduce the effects of spectral masking in moderate bilateral sensorineural hearing loss	Kulkarni, P. N.; Pandey, P. C.; Jangamashetti, D. S.	2012
The hidden effect of hearing acuity on speech recall, and compensatory effects of self-paced listening	Piquado, T.; Benichov, J. I.; Brownell, H.; Wingfield, A.	2012
Speech-in-noise measures: variable versus fixed speech and noise levels	Wilson, R. H.; McArdle, R.	2012
Acceptable noise level (ANL) with Danish and non-semantic speech materials in adult hearing-aid users	Olsen, S. O.; Lantz, J.; Nielsen, L. H.; Brannstrom, K. J.	2012
Evaluation of model-based versus non-parametric monaural noise-reduction approaches for hearing aids	Harlander, N.; Rosenkranz, T.; Hohmann, V.	2012

Hearing-aid outcomes in Chinese adults: Clinical application and psychometric properties of the Chinese version of the Satisfaction with Amplification in Daily Life questionnaire	Shan Kam, A. C.	2012
Measurement and prediction of the acceptable noise level for single-microphone noise reduction algorithms	Fredelake, S.; Holube, I.; Schlueter, A.; Hansen, M.	2012
Comparison of three types of French speech-in-noise tests: a multi-center study	Jansen, S.; Luts, H.; Wagener, K. C.; Kollmeier, B.; Del Rio, M.; Dauman, R.; James, C.; Fraysse, B.; Vormes, E.; Frachet, B.; Wouters, J.; van Wieringen, A.	2012
Fractionated stereotactic radiotherapy of vestibular schwannomas accelerates hearing loss	Rasmussen, R.; Claesson, M.; Stangerup, S. E.; Roed, H.; Christensen, I. J.; Caye-Thomasen, P.; Juhler, M.	2012
Temporal-envelope reconstruction for hearing-impaired listeners	Lorenzi, C.; Wallaert, N.; Gnansia, D.; Leger, A. C.; Ives, D. T.; Chays, A.; Garnier, S.; Cazals, Y.	2012
The influence of cognitive aspects and auditory processes on the hearing aid acclimatization in the elderly	Pinheiro, M. M.; Iorio, M. C.; Miranda, E. C.; Dias, K. Z.; Pereira, L. D.	2012
Evaluating a Dichotomized Measure of Self-Reported Hearing Loss Against Gold Standard Audiometry: Prevalence Estimates and Age Bias in a Pooled National Data set	Kiely, K. M.; Gopinath, B.; Mitchell, P.; Browning, C. J.; Anstey, K. J.	2012
Comparison of nursing home hearing handicap index with audiological findings: A presbycusis study	Nilforoush, M. H.; Nasr Esfahani, A. A.; Ishaghi, R.; Sepehrnejad, M.	2012

Long-term effect of acupuncture for treatment of tinnitus: a randomized, patient- and assessor-blind, sham-acupuncture-controlled, pilot trial	Jeon, S. W.; Kim, K. S.; Nam, H. J.	2012
Hearing, mobility, and pain predict mortality: a longitudinal population-based study	Feeny, D.; Huguet, N.; McFarland, B. H.; Kaplan, M. S.; Orpana, H.; Eckstrom, E.	2012
Effects of Multi-Channel Compression on Speech Intelligibility at the Patients with Loudness-Recruitment	Polat, Z.; Atas, A.; Sennaroglu, G.	2012
Computer skills and internet use in adults aged 50-74 years: influence of hearing difficulties	Henshaw, H.; Clark, D. P.; Kang, S.; Ferguson, M. A.	2012
Speech perception benefits of internet versus conventional telephony for hearing-impaired individuals	Mantokoudis, G.; Dubach, P.; Pfiffner, F.; Kompis, M.; Caversaccio, M.; Senn, P.	2012
The effects of musical and linguistic components in recognition of real-world musical excerpts by cochlear implant recipients and normal-hearing adults	Gfeller, K.; Jiang, D.; Oleson, J. J.; Driscoll, V.; Olszewski, C.; Knutson, J. F.; Turner, C.; Gantz, B.	2012
Cochlear implant optimized noise reduction	Mauger, S. J.; Arora, K.; Dawson, P. W.	2012
Speech recognition index of workers with tinnitus exposed to environmental or occupational noise: A comparative study	Soalheiro, M.; Rocha, L.; Do Vale, D. F.; Fontes, V.; Valente, D.; Teixeira, L. R.	2012
Performance on tests of central auditory processing by individuals exposed to high-intensity blasts	Gallun, F. J.; Diedesch, A. C.; Kubli, L. R.; Walden, T. C.; Folmer, R. L.; Lewis, M. S.; McDermott, D. J.; Fausti, S. A.; Leek, M. R.	2012

Effect of speaker age on speech recognition and perceived listening effort in older adults with hearing loss	McAuliffe, M. J.; Wilding, P. J.; Rickard, N. A.; O'Beirne, G. A.	2012
Talker differences in clear and conversational speech: vowel intelligibility for older adults with hearing loss	Ferguson, S. H.	2012
Consequences of broad auditory filters for identification of multichannel-compressed vowels	Souza, P.; Wright, R.; Bor, S.	2012
Informational masking and spatial hearing in listeners with and without unilateral hearing loss	Rothpletz, A. M.; Wightman, F. L.; Kistler, D. J.	2012
Relationship between consonant recognition in noise and hearing threshold	Yoon, Y. S.; Allen, J. B.; Gooler, D. M.	2012
Speech recognition and acoustic features in combined electric and acoustic stimulation	Yoon, Y. S.; Li, Y.; Fu, Q. J.	2012
Speech perception with music maskers by cochlear implant users and normal-hearing listeners	Eskridge, E. N.; Galvin 3rd, J. J.; Aronoff, J. M.; Li, T.; Fu, Q. J.	2012
Effect of fundamental-frequency and sentence-onset differences on speech-identification performance of young and older adults in a competing-talker background	Lee, J. H.; Humes, L. E.	2012
Assessing the role of spectral and intensity cues in spectral ripple detection and discrimination in cochlear-implant users	Anderson, E. S.; Oxenham, A. J.; Nelson, P. B.; Nelson, D. A.	2012
Auditory externalization in hearing-impaired listeners: the effect of pinna cues and number of talkers	Boyd, A. W.; Whitmer, W. M.; Soraghan, J. J.; Akeroyd, M. A.	2012
The relative importance of consonant and vowel segments to the recognition of words and sentences: effects of age and hearing loss	Fogerty, D.; Kewley-Port, D.; Humes, L. E.	2012
Effects of reverberation and masker fluctuations on binaural unmasking of speech	George, E. L. J.; Festen, J. M.; Theo Goverts, S.	2012
Sentence recognition in noise promoting or suppressing masking release by normal-hearing and cochlear-implant listeners	Kwon, B. J.; Perry, T. T.; Wilhelm, C. L.; Healy, E. W.	2012
Perceptually optimized gain function for cochlear implant signal-to-noise ratio based noise reduction	Mauger, S. J.; Dawson, P. W.; Hersbach, A. A.	2012
The use of acoustic cues for phonetic identification: effects of spectral degradation and electric hearing	Winn, M. B.; Chatterjee, M.; Idsardi, W. J.	2012

Predicting the speech reception threshold of cochlear implant listeners using an envelope-correlation based measure	Yousefian, N.; Loizou, P. C.	2012
Musical background not associated with self-perceived hearing performance or speech perception in postlingual cochlear-implant users	Fuller, C.; Free, R.; Maat, B.; Baskent, D.	2012
Binaural temporal fine structure sensitivity, cognitive function, and spatial speech recognition of hearing-impaired listeners (L)	Neher, T.; Lunner, T.; Hopkins, K.; Moore, B. C.	2012
Relationship between masking release in fluctuating maskers and speech reception thresholds in stationary noise	Christiansen, C.; Dau, T.	2012
Improving word recognition in noise among hearing-impaired subjects with a single-channel cochlear noise-reduction algorithm	Fink, N.; Furst, M.; Muchnik, C.	2012
Temporal and spectral masking release in low- and mid-frequency regions for normal-hearing and hearing-impaired listeners	Leger, A. C.; Moore, B. C. J.; Lorenzi, C.	2012
Age affects responses on the Speech, Spatial, and Qualities of Hearing Scale (SSQ) by adults with minimal audiometric loss	Banh, J.; Singh, G.; Pichora-Fuller, M. K.	2012
Initial-fit approach versus verified prescription: Comparing self-perceived hearing aid benefit	Abrams, H. B.; Chisolm, T. H.; McManus, M.; McArdle, R.	2012
The Hearing Impairment Impact-Significant Other Profile (HII-SOP): a tool to measure hearing loss-related quality of life in spouses of people with hearing loss	Preminger, J. E.; Meeks, S.	2012
Using patient perceptions of relative benefit and enjoyment to assess auditory training	Tye-Murray, N.; Sommers, M. S.; Mauze, E.; Schroy, C.; Barcroft, J.; Spehar, B.	2012
Subjective and objective outcomes from new BiCROS technology in a veteran sample	Williams, V. A.; McArdle, R. A.; Chisolm, T. H.	2012
Evaluation of TIMIT sentence list equivalency with adult cochlear implant recipients	King, S. E.; Firszt, J. B.; Reeder, R. M.; Holden, L. K.; Strube, M.	2012
Efficacy of a reverse cardioid directional microphone	Kuk, F.; Keenan, D.	2012
Are two ears not better than one?	McArdle, R. A.; Killion, M.; Mennite, M. A.; Chisolm, T. H.	2012
The relationship between high-frequency pure-tone hearing loss, hearing in noise test (HINT) thresholds, and the articulation index	Vermiglio, A. J.; Soli, S. D.; Freed, D. J.; Fisher, L. M.	2012

Telephone screening tests for functionally impaired hearing: current use in seven countries and development of a US version	Watson, C. S.; Kidd, G. R.; Miller, J. D.; Smits, C.; Humes, L. E.	2012
The revised speech perception in noise test (R-SPIN) in a multiple signal-to-noise ratio paradigm	Wilson, R. H.; McArdl, R.; Watt, K. L.; Smith, S. L.	2012
The effects of energetic and informational masking on The Words-in-Noise Test (WIN)	Wilson, R. H.; Trivette, C. P.; Williams, D. A.; Watts, K. L.	2012
The Words-in-Noise Test (WIN), list 3: a practice list	Wilson, R. H.; Watts, K. L.	2012
Working memory capacity may influence perceived effort during aided speech recognition in noise	Rudner, M.; Lunner, T.; Behrens, T.; Thoren, E. S.; Ronnberg, J.	2012
Self-Reported Hearing Loss in Older Adults Is Associated with Future Decline in Instrumental Activities of Daily Living but Not in Social Participation	Yamada, M.; Nishiwaki, Y.; Michikawa, T.; Takebayashi, T.	2012
Sentence comprehension in proficient adult cochlear implant users: On the vulnerability of syntax	Hahne, A.; Wolf, A.; Muller, J.; Murbe, D.; Friederici, A. D.	2012
Spectrum of immune-mediated inner ear disease and cochlear implant results	Malik, M. U.; Pandian, V.; Masood, H.; Diaz, D. A.; Varela, V.; Davalos-Balderas, A. J.; Parra-Cardenas, M.; Seo, P.; Francis, H. W.	2012
Elderly patients benefit from cochlear implantation regarding auditory rehabilitation, quality of life, tinnitus, and stress	Olze, H.; Grabel, S.; Forster, U.; Zirke, N.; Huhnd, L. E.; Haupt, H.; Mazurek, B.	2012
Hearing aid satisfaction and use in the advanced digital era	Kaplan-Neeman, R.; Muchnik, C.; Hildesheimer, M.; Henkin, Y.	2012

Cochlear implant performance in geriatric patients	Lenarz, M.; Sonmez, H.; Joseph, G.; Buchner, A.; Lenarz, T.	2012
Effect of gender on the hearing performance of adult cochlear implant patients	Lenarz, M.; Sonmez, H.; Joseph, G.; Buchner, A.; Lenarz, T.	2012
Hearing handicap, rather than measured hearing impairment, predicts poorer quality of life over 10 years in older adults	Gopinath, B.; Schneider, J.; Hickson, L.; McMahon, C. M.; Burlutsky, G.; Leeder, S. R.; Mitchell, P.	2012
Cochlear implantation in older adults	Lin, F. R.; Chien, W. W.; Li, L.; Clarrett, D. M.; Niparko, J. K.; Francis, H. W.	2012
Treatment of chronic tinnitus with theta burst stimulation: A randomized controlled trial	Plewnia, C.; Vonthein, R.; Wasserka, B.; Arfeller, C.; Naumann, A.; Schraven, S. P.; Plontke, S. K.	2012
Long-term monaural auditory deprivation and bilateral cochlear implants	Boisvert, I.; McMahon, C. M.; Dowell, R. C.	2012
Validation of a questionnaire to identify hearing loss among farm operators	McCullagh, M. C.	2012
Temporal and speech processing skills in normal hearing individuals exposed to occupational noise	Kumar, U. A.; Ameenudin, S.; Sangamanatha, A. V.	2012

Clinical trial results with the med-el fine structure processing coding strategy in experienced cochlear implant users	Mller, J.; Brill, S.; Hagen, R.; Moeltner, A.; Brockmeier, S. J.; Stark, T.; Helbig, S.; Maurer, J.; Zahnert, T.; Zierhofer, C.; Nopp, P.; Anderson, I.	2012
Bone-anchored implantation for single-sided deafness in patients with less than profound hearing loss	Zeitler, D. M.; Snapp, H. A.; Telischi, F. F.; Angeli, S. I.	2012
Mobile and landline telephone performance outcomes among telephone-using cochlear implant recipients	Tan, B. Y.; Gluth, M. B.; Statham, E. L.; Eikelboom, R. H.; Atlas, M. D.	2012
Long-term performance of cochlear implants in postlingually deafened adults	Lenarz, M.; Sonmez, H.; Joseph, G.; Buchner, A.; Lenarz, T.	2012
Intratympanic treatment of intractable unilateral meniere disease: Gentamicin or dexamethasone? A randomized controlled trial	Casani, A. P.; Piaggi, P.; Cerchiai, N.; Seccia, V.; Franceschini, S. S.; Dallan, I.	2012
Bimodal hearing on music and speech with FSP and HDCIS	Atwood, K.; Runge, C.; Friedland, D. R.	2012
Cochlear implant in far advanced otosclerosis	Ruiz, H. E.; Curet, C.; Salvadores, M. I.; Romani, C.; Rubino, L.; Queirolo, A.; Dotto, G.	2012
Results with cochlear implantation in adults with speech recognition scores exceeding current criteria	Amoodi, H. A.; Mick, P. T.; Shipp, D. B.; Friesen, L. M.; Nedzelski, J. M.; Chen, J. M.; Lin, V. Y.	2012
Postoperative validation of bone-anchored implants in the single-sided deafness population	Snapp, H.; Angeli, S.; Telischi, F. F.; Fabry, D.	2012

Choice of ear for cochlear implantation in adults with monaural sound-deprivation and unilateral hearing aid	Boisvert, I.; Lyxell, B.; Maki-Torkko, E.; McMahon, C. M.; Dowell, R. C.	2012
Oral steroid treatment for hearing improvement in Meniere's disease and endolymphatic hydrops	Fisher, L. M.; Derebery, M. J.; Friedman, R. A.	2012
Spectral and temporal measures in hybrid cochlear implant users: on the mechanism of electroacoustic hearing benefits	Golub, J. S.; Won, J. H.; Drennan, W. R.; Worman, T. D.; Rubinstein, J. T.	2012
The round window: is it the "cochleostomy" of choice? Experience in 130 consecutive cochlear implants	Gudis, D. A.; Montes, M.; Bigelow, D. C.; Ruckenstein, M. J.	2012
Cochlear implantation in patients profoundly deafened after head injury	Khwaja, S.; Mawman, D.; Nichani, J.; Bruce, I.; Green, K.; Lloyd, S.	2012
Recovery from forward masking in elderly cochlear implant users	Lee, E. R.; Friedland, D. R.; Runge, C. L.	2012
Third-generation bisphosphonates for treatment of sensorineural hearing loss in otosclerosis	Quesnel, A. M.; Seton, M.; Merchant, S. N.; Halpin, C.; McKenna, M. J.	2012
Auditory brainstem implants in NF2 patients: results and review of the literature	Sanna, M.; Di Lella, F.; Guida, M.; Merkus, P.	2012
Hearing outcomes after surgical plugging of the superior semicircular canal by a middle cranial fossa approach	Ward, B. K.; Agrawal, Y.; Nguyen, E.; Della Santina, C. C.; Limb, C. J.; Francis, H. W.; Minor, L. B.; Carey, J. P.	2012

Benefit of a commercially available cochlear implant processor with dual-microphone beamforming: a multi-center study	Wolfe, J.; Parkinson, A.; Schafer, E. C.; Gilden, J.; Rehwinkel, K.; Mansanares, J.; Coughlan, E.; Wright, J.; Torres, J.; Gannaway, S.	2012
Clinical selection criteria for a second cochlear implant for bimodal listeners	Yoon, Y. S.; Shin, Y. R.; Fu, Q. J.	2012
Speech-in-noise and subjective benefit with active middle ear implant omnidirectional and directional microphones: a within-subjects comparison	Wolframm, M. D.; Giarbini, N.; Streitberger, C.	2012
Comparison of 2 implantable bone conduction devices in patients with single-sided deafness using a daily alternating method	Desmet, J. B.; Wouters, K.; De Bodt, M.; Van de Heyning, P.	2012
Extra benefit of a second cochlear implant with respect to health-related quality of life and tinnitus	Olze, H.; Grabel, S.; Haupt, H.; Forster, U.; Mazurek, B.	2012
Audiological and subjective benefit results in bone-anchored hearing device users	Boleas-Aguirre, M. S.; Bulnes Plano, M. D.; de Erenchun Lasa, I. R.; Ibanez Beroiz, B.	2012
The role of hearing preservation on electrical thresholds and speech performances in cochlear implantation	D'Elia, A.; Bartoli, R.; Giagnotti, F.; Quaranta, N.	2012
Stapes surgery in profound hearing loss due to otosclerosis	Lachance, S.; Bussieres, R.; Cote, M.	2012
Hearing ability with age in northern European women: a new web-based approach to genetic studies	Wolber, L. E.; Steves, C. J.; Spector, T. D.; Williams, F. M.	2012

Pre-, Per- and Postoperative Factors Affecting Performance of Postlinguistically Deaf Adults Using Cochlear Implants: A New Conceptual Model over Time	Lazard, D. S.; Vincent, C.; Venail, F.; van de Heyning, P.; Truy, E.; Sterkers, O.; Skarzynski, P. H.; Skarzynski, H.; Schauwers, K.; O'Leary, S.; Mawman, D.; Maat, B.; Kleine-Punte, A.; Huber, A. M.; Green, K.; Govaerts, P. J.; Fraysse, B.; Dowell, R.; Dillier, N.; Burke, E.; Beynon, A.; Bergeron, F.; Baskent, D.; Artieres, F.; Blamey, P. J.	2012
Frequent false hearing by older adults: The role of age differences in metacognition	Rogers, C. S.; Jacoby, L. L.; Sommers, M. S.	2012
Musicians experience less age-related decline in central auditory processing	Zendel, B. R.; Alain, C.	2012
The effectiveness of audiometric evaluation in drug treatment for otospongiosis	Vicente Ade, O.; Yamashita, H. K.; Cruz, O. L.; Suzuki, F. B.; Penido Nde, O.	2012
Factors affecting older adults' hearing-aid use	Solheim, J.; Kvaerner, K. J.; Sandvik, L.; Falkenberg, E. S.	2012
Preliminary identification of central auditory processing screening tests for individuals with multiple sclerosis	Lewis, M. S.; Wilmington, D.; Hutter, M.; McMillan, G.; Casiana, L.; Fitzpatrick, M.; Lilly, D. J.; Bourdette, D.; Folmer, R.; Fausti, S.	2012

Speech-perception-in-noise and bilateral spatial abilities in adults with delayed sequential cochlear implant	Oosthuizen, I.; Swanepoel de, W.; van Dijk, C.	2012
Patient-Reported Auditory Functions After Stroke of the Central Auditory Pathway	Bamiou, D. E.; Werring, D.; Cox, K.; Stevens, J.; Musiek, F. E.; Brown, M. M.; Luxon, L. M.	2012
Across-site patterns of modulation detection: relation to speech recognition	Garadat, S. N.; Zwolan, T. A.; Pfingst, B. E.	2012
Acoustic cue integration in speech intonation recognition with cochlear implants	Peng, S. C.; Chatterjee, M.; Lu, N.	2012
Audiologist-driven versus patient-driven fine tuning of hearing instruments	Boymans, M.; Dreschler, W. A.	2012
THE EVALUTION OF CENTRAL AUDITORY PROCESSING IN THE GERIATRIC POPULATION	Beriat, G. K.; Ozkan, S.	2012
Clinical manifestations of aural fullness	Park, M. S.; Lee, H. Y.; Kang, H. M.; Ryu, E. W.; Lee, S. K.; Yeo, S. G.	2012
Hearing-impaired adults are at increased risk of experiencing emotional distress and social engagement restrictions five years later	Gopinath, Bamini; Hickson, Louise; Schneider, Julie; McMahon, Catherine M; Burlutsky, George; Leeder, Stephen R; Mitchell, Paul %J Age; Ageing	2012
Word recognition within a linguistic context: Effects of age, hearing acuity, verbal ability and cognitive function	Benichov, Jonathan; Cox, L Clarke; Tun, Patricia A; Wingfield, Arthur %J Ear; Hearing	2012

New measures of masked text recognition in relation to speech-in-noise perception and their associations with age and cognitive abilities	Besser, Jana; Zekveld, Adriana A; Kramer, Sophia E; RoÃànnberg, Jerker; Festen, Joost M %J Journal of Speech, Language,; Hearing Research	2012
Perception of Synthetic Speech in Adult Users of Cochlear Implants	Nagao, K.; Paullin, M.; Polikoff, J. B.; Lilley, J.; Bunnell, H. T.; International Speech Communications, Association	2012
Word recognition for temporally and spectrally distorted materials: The effects of age and hearing loss	Smith, Sherri L; Pichora-Fuller, Margaret Kathleen; Wilson, Richard H; MacDonald, Ewen N %J Ear; hearing	2012
Audiovisual benefit for recognition of speech presented with single-talker noise in older listeners	Jesse, Alexandra; Janse, Esther %J Language; cognitive processes	2012
Hearing Loss and the Use of Acoustic Cues in Phonetic Categorisation of Fricatives	Scharenborg, O.; Janse, E.; International Speech Communications, Association	2012
Perceptual Learning of /f/-/s/ by Older Listeners	Scharenborg, O.; Janse, E.; Weber, A.; International Speech Communications, Association	2012
Concurrent Vowel Identification and Speech Perception in Noise in Individuals With Cochlear Hearing Loss	Kumar, U. A.; Rayanagoudar, P.; Nambi, A.	2013

Restoring speech perception with cochlear implants by spanning defective electrode contacts	Frijns, J. H. M.; Snel-Bongers, J.; Vellinga, D.; Schrage, E.; Vanpoucke, F. J.; Briaire, J. J.	2013
Cochlear implants with fine structure processing improve speech and tone perception in Mandarin-speaking adults	Chen, X. Q.; Liu, B.; Liu, S.; Mo, L. Y.; Li, Y. L.; Kong, Y.; Zheng, J.; Li, Y. X.; Gong, S. S.; Han, D. M.	2013
Evaluation of psychoacoustic tests and P300 event-related potentials in elderly patients with hyperhomocysteinemia	Diaz-Leines, S.; Penaloza-Lopez, Y. R.; Serrano-Miranda, T. A.; Flores-Avalos, B.; Vidal-Ixta, M. T.; Jimenez-Herrera, B.	2013
Tool kit for screening otologic function of older adults1	Weinstein, B. E.	2013
Learning to listen again: the role of compliance in auditory training for adults with hearing loss	Chisolm, T. H.; Saunders, G. H.; Frederick, M. T.; McArdle, R. A.; Smith, S. L.; Wilson, R. H.	2013
Semi-structured listening experience (listening training) in hearing aid fitting: influence on dichotic listening	Lavie, L.; Attias, J.; Karni, A.	2013
Masking release and modulation interference in cochlear implant and simulation listeners	Jin, S. H.; Nie, Y.; Nelson, P.	2013
Hearing status in older persons: a significant determinant of depression and loneliness? Results from the longitudinal aging study amsterdam	Pronk, M.; Deeg, D. J.; Kramer, S. E.	2013
Using the Speech Understanding in Noise (SUN) Test for Adult Hearing Screening	Paglialonga, A.; Grandori, F.; Tognola, G.	2013
Vestibular schwannoma in the only hearing ear: role of cochlear implants	Di Lella, F.; Merkus, P.; Di Trapani, G.; Taibah, A.; Guida, M.; Sanna, M.	2013
Impact of hearing loss on quality of life of Singapore patients and correlation between Audiogram and hearing disability	Zhang, H. Y.; Medapati, S. V. R.; Ho, E. C.; Li, K. X.; Ong, W. M. W.	2013

Comparison between bilateral cochlear implants and Neurelec Digisonic() SP Binaural cochlear implant: speech perception, sound localization and patient self-assessment	Bonnard, D.; Lautissier, S.; Bosset-Audoit, A.; Coriat, G.; Beraha, M.; Maunoury, A.; Martel, J.; Darrouzet, V.; Bebear, J. P.; Dauman, R.	2013
Factors affecting auditory performance of postlinguistically deaf adults using cochlear implants: an update with 2251 patients	Blamey, P.; Artieres, F.; Baskent, D.; Bergeron, F.; Beynon, A.; Burke, E.; Dillier, N.; Dowell, R.; Fraysse, B.; Gallego, S.; Govaerts, P. J.; Green, K.; Huber, A. M.; Kleine-Punte, A.; Maat, B.; Marx, M.; Mawman, D.; Mosnier, I.; O'Connor, A. F.; O'Leary, S.; Rousset, A.; Schauwers, K.; Skarzynski, H.; Skarzynski, P. H.; Sterkers, O.; Terranti, A.; Truy, E.; Van de Heyning, P.; Venail, F.; Vincent, C.; Lazard, D. S[illegible]	2013
Using temporal modulation sensitivity to select stimulation sites for processor MAPs in cochlear implant listeners	Garadat, S. N.; Zwolan, T. A.; Pfingst, B. E.	2013
Primary observation of early transtympanic steroid injection in patients with delayed treatment of noise-induced hearing loss	Zhou, Y.; Zheng, G.; Zheng, H.; Zhou, R.; Zhu, X.; Zhang, Q.	2013
Evaluation of speech intelligibility and sound localization abilities with hearing aids using binaural wireless technology	Ibrahim, I.; Parsa, V.; Macpherson, E.; Cheesman, M.	2013
Evaluation of the Hearing Aid Rehabilitation Questionnaire in Dutch: examination of its psychometric properties and potential use as a screening instrument	Chenault, M. N.; Anteunis, L. J. C.; Berger, M. P. F.	2013

Evolution of speech audiometry results in adult patients with cochlear implants	Hiel, A. L.; Gerard, J. M.; Doyen, A.; Castelein, S.; Decat, M.; Wiener, V.; Deggouj, N.	2013
Cochlear implantation in the elderly: surgical and hearing outcomes	Alice, B.; Silvia, M.; Laura, G.; Patrizia, T.; Roberto, B.	2013
Internet-based hearing screening using speech-in-noise: Validation and comparisons of self-reported hearing problems, quality of life and phonological representation	Molander, P.; Nordqvist, P.; Oberg, M.; Lunner, T.; Lyxell, B.; Andersson, G.	2013
Visual activity predicts auditory recovery from deafness after adult cochlear implantation	Strelnikov, K.; Rouger, J.; Demonet, J. F.; Lagleyre, S.; Fraysse, B.; Deguine, O.; Barone, P.	2013
Hearing aids and recovery times: A study according to cognitive status	Ghiringhelli, R.; Iorio, M. C. M.	2013
Cochlear dead region and word recognition of Mandarin Chinese in Taiwan	Tseng, L. M.; Lee, G. S.; Yang, E.; Young, N.; Hsu, C. Y.	2013
Is cochlear implantation a good treatment method for profoundly deafened elderly?	Lachowska, M.; Pastuszka, A.; Glinka, P.; Niemczyk, K.	2013
The effect of emphasis and position on word identification by adult cochlear implant listeners	Morris, D.; Magnusson, L.; Jonsson, R.	2013
Non-flat audiograms in sensorineural hearing loss and speech perception	Andrade, K. C.; Menezes Pde, L.; Carnauba, A. T.; Rodrigues, R. G.; Leal Mde, C.; Pereira, L. D.	2013
Cochlear implants in adults over 60: a study of communicative benefits and the impact on quality of life	Ramos, A.; Guerra-Jimenez, G.; Rodriguez, C.; Borkoski, S.; Falcon, J. C.; Perez, D.	2013

Postlingual adult performance in noise with HiRes 120 and ClearVoice Low, Medium, and High	Holden, L. K.; Brenner, C.; Reeder, R. M.; Firszt, J. B.	2013
Clinical use of electrode differentiation to enhance programming of cochlear implants	Saleh, S. M.; Saeed, S. R.; Meerton, L.; Moore, D. R.; Vickers, D. A.	2013
Performance of the harmonyTM behind-the-ear processor with the first generation of Advanced BionicsTM implant systems	Brendel, M.; Rottmann, T.; Lenarz, T.; Buechner, A.	2013
Results of auditory rehabilitation in elderly users of hearing aids evaluated by a dichotic test	Lessa, A. H.; Hennig, T. R.; Costa, M. J.; Rossi, A. G.	2013
Association among depression, physical functioning, and hearing and vision impairment in adults with diabetes	Loprinzi, P. D.; Smit, E.; Pariser, G.	2013
Age-related changes in listening effort for various types of masker noises	Desjardins, J. L.; Doherty, K. A.	2013
The effects of hearing aid use on listening effort and mental fatigue associated with sustained speech processing demands	Hornsby, B. W.	2013
Aging and the perception of temporally interleaved words	Helfer, K. S.; Mason, C. R.; Marino, C.	2013
Working memory, age, and hearing loss: susceptibility to hearing aid distortion	Arehart, K. H.; Souza, P.; Baca, R.; Kates, J. M.	2013
Reconstructing wholes from parts: effects of modality, age, and hearing loss on word recognition	Krull, V.; Humes, L. E.; Kidd, G. R.	2013
The effect of hearing aid signal-processing schemes on acceptable noise levels: perception and prediction	Wu, Y. H.; Stangl, E.	2013
How hearing aids, background noise, and visual cues influence objective listening effort	Picou, E. M.; Ricketts, T. A.; Hornsby, B. W.	2013
An adaptive Australian Sentence Test in Noise (AuSTIN)	Dawson, P. W.; Hersbach, A. A.; Swanson, B. A.	2013
Localization and speech understanding by a patient with bilateral cochlear implants and bilateral hearing preservation	Dorman, M. F.; Spahr, A. J.; Loiselle, L.; Zhang, T.; Cook, S.; Brown, C.; Yost, W.	2013

Factors affecting open-set word recognition in adults with cochlear implants	Holden, L. K.; Finley, C. C.; Firszt, J. B.; Holden, T. A.; Brenner, C.; Potts, L. G.; Gotter, B. D.; Vanderhoof, S. S.; Mispagel, K.; Heydebrand, G.; Skinner, M. W.	2013
Optimizing the combination of acoustic and electric hearing in the implanted ear	Karsten, S. A.; Turner, C. W.; Brown, C. J.; Jeon, E. K.; Abbas, P. J.; Gantz, B. J.	2013
Effect of hearing aid bandwidth on speech recognition performance of listeners using a cochlear implant and contralateral hearing aid (bimodal hearing)	Neuman, A. C.; Svirsky, M. A.	2013
Effects of input processing and type of personal frequency modulation system on speech-recognition performance of adults with cochlear implants	Wolfe, J.; Schafer, E.; Parkinson, A.; John, A.; Hudson, M.; Wheeler, J.; Mucci, A.	2013
Relationship between auditory function of nonimplanted ears and bimodal benefit	Zhang, T.; Spahr, A. J.; Dorman, M. F.; Saoji, A.	2013
Cochlear implantation with hearing preservation yields significant benefit for speech recognition in complex listening environments	Gifford, R. H.; Dorman, M. F.; Skarzynski, H.; Lorens, A.; Polak, M.; Driscoll, C. L.; Roland, P.; Buchman, C. A.	2013
Efficient hearing screening in noise-exposed listeners using the digit triplet test	Jansen, S.; Luts, H.; Dejonckere, P.; van Wieringen, A.; Wouters, J.	2013
Speech perception with combined electric-acoustic stimulation and bilateral cochlear implants in a multisource noise field	Rader, T.; Fastl, H.; Baumann, U.	2013
Adaptation of scoring methods for testing cochlear implant users using the Cantonese Hearing In Noise Test (CHINT)	Wong, L. L.; Keung, S. K.	2013
Stages of change in adults with acquired hearing impairment seeking help for the first time: application of the transtheoretical model in audiologic rehabilitation	Laplante-Levesque, A.; Hickson, L.; Worrall, L.	2013

Effect of speaking rate on recognition of synthetic and natural speech by normal-hearing and cochlear implant listeners	Ji, C.; Galvin 3rd, J. J.; Xu, A.; Fu, Q. J.	2013
Decline in Older Persons' Ability to Recognize Speech in Noise: The Influence of Demographic, Health-Related, Environmental, and Cognitive Factors	Pronk, M.; Deeg, D. J. H.; Festen, J. M.; Twisk, J. W.; Smits, C.; Comijs, H. C.; Kramer, S. E.	2013
Xylene-induced auditory dysfunction in humans	Fuente, A.; McPherson, B.; Cardemil, F.	2013
Comparative efficacies of intratympanic steroid administration and classic therapy in the management of idiopathic sudden sensorineural hearing loss	Gumussoy, M.; Arslan, I. B.; Cukurova, I.	2013
Esteem(A (R)) middle ear device versus conventional hearing aids for rehabilitation of bilateral sensorineural hearing loss	Monini, S.; Biagini, M.; Atturo, F.; Barbara, M.	2013
The transtympanic promontory stimulation test in patients with auditory deprivation: correlations with electrical dynamics of cochlear implant and speech perception	Alfelasi, M.; Piron, J. P.; Mathiolon, C.; Lenel, N.; Mondain, M.; Uziel, A.; Venail, F.	2013
Intratympanic steroids as primary initial treatment of idiopathic sudden sensorineural hearing loss. The Hospital Universitario Ramon y Cajal experience and review of the literature	Labatut, T.; Daza, M. J.; Alonso, A.	2013
New cochlear implant technologies improve performance in post-meningitic deaf patients	Mosnier, I.; Felice, A.; Esquia, G.; Borel, S.; Bouccara, D.; Ambert-Dahan, E.; Smadja, M.; Ferrary, E.; Sterkers, O.	2013
Ondansetron in patients with tinnitus: randomized double-blind placebo-controlled study	Taslimi, S.; Vahidi, H.; Pourvaziri, A.; Modabbernia, A.; Fallah, A. Y.; Yazdani, N.; Taslimi, N.; Hosseini, M.; Zarandi, M. M.	2013
Audiological correlates of tumor parameters in acoustic neuromas	Tutar, H.; Duzlu, M.; Goksu, N.; Ustun, S.; Bayazit, Y.	2013

Indications and outcome of subtotal petrosectomy for active middle ear implants	Verhaert, N.; Mojallal, H.; Schwab, B.	2013
Expectation and entropy in spoken word recognition: effects of age and hearing acuity	Lash, A.; Rogers, C. S.; Zoller, A.; Wingfield, A.	2013
Evaluating the use of an assistive listening device for communication efficiency using the Diapix task: a pilot study	McInerney, M.; Walden, P.	2013
Independent impacts of age and hearing loss on spatial release in a complex auditory environment	Gallun, F. J.; Diedesch, A. C.; Kampel, S. D.; Jakien, K. M.	2013
Training changes processing of speech cues in older adults with hearing loss	Anderson, S.; White-Schwoch, T.; Choi, H. J.; Kraus, N.	2013
Compensatory changes in cortical resource allocation in adults with hearing loss	Campbell, J.; Sharma, A.	2013
Upregulation of cognitive control networks in older adults' speech comprehension	Erb, J.; Obleser, J.	2013
Musicians change their tune: how hearing loss alters the neural code	Parbery-Clark, A.; Anderson, S.; Kraus, N.	2013
Multidimensional scaling between acoustic and electric stimuli in cochlear implant users with contralateral hearing	Vermeire, K.; Landsberger, D. M.; Schleich, P.; Van de Heyning, P. H.	2013
Progressive hereditary hearing impairment caused by a MYO6 mutation resembles presbyacusis	Oonk, A. M. M.; Leijendeckers, J. M.; Lammers, E. M.; Weegerink, N. J. D.; Oostrik, J.; Beynon, A. J.; Huygen, P. L. M.; Kunst, H. P. M.; Kremer, H.; Snik, A. F. M.; Pennings, R. J. E.	2013

Clinical aspects of an autosomal dominantly inherited hearing impairment linked to the DFNA60 locus on chromosome 2q23.1-2q23.3	van Beelen, E.; Schraders, M.; Huygen, P. L. M.; Oostrik, J.; Plantinga, R. F.; van Drunen, W.; Collin, R. W. J.; Kooper, D. P.; Pennings, R. J. E.; Cremers, Cwrj; Kremer, H.; Kunst, H. P. M.	2013
Validation of a speech-in-noise test used for verification of hearing aid fitting	Grunditz, M.; Magnusson, L.	2013
Acceptable noise level in Danish adult subjects diagnosed with unilateral Meniere's disease	Olsen, S. O.; Lantz, J.; Brannstrom, K. J.; Nielsen, L. H.	2013
Evaluation of the Freiburg monosyllabic speech test in background noise. [German, English]	Lohler, J.; Akcicek, B.; Pilnik, M.; Saager-Post, K.; Dazert, S.; Biedron, S.; Oeken, J.; Murbe, D.; Lobert, J.; Laszig, R.; Wesarg, T.; Langer, C.; Plontke, S.; Rahne, T.; Machate, U.; Noppeney, R.; Schultz, K.; Plinkert, P.; Hoth, S.; Praetorius, M.; Schlattmann, P.; Pau, H. W.; Ehrt, K.; Hagen, R.; Shehata-Dieler, W.; Cebulla, M.; Walther, L. E.; Ernst, A.	2013
Application of new speech intelligibility tests in silence for the assessment of acquired hearing losses. [German, English]	Sukowski, H.; Wagener, K. C.; Thiele, C.; Uppenkamp, S.; Kollmeier, B.	2013

Phoneme-based self hearing assessment on a smartphone	Choi, J. M.; Sohn, J.; Ku, Y.; Kim, D.; Lee, J.	2013
A short form of the Speech, Spatial and Qualities of Hearing scale suitable for clinical use: The SSQ12	Noble, W.; Jensen, N. S.; Naylor, G.; Bhullar, N.; Akeroyd, M. A.	2013
Placebo effects in hearing-aid trials are reliable	Dawes, P.; Hopkins, R.; Munro, K. J.	2013
Prevalence & characteristics of severe and profound hearing loss in adults in a UK National Health Service clinic	Turton, L.; Smith, P.	2013
A comparative study of hearing aids and round window application of the vibrant sound bridge (VSB) for patients with mixed or conductive hearing loss	Marino, R.; Linton, N.; Eikelboom, R. H.; Statham, E.; Rajan, G. P.	2013
Hearing-aid users' voices: a factor that could affect directional benefit	Wu, Y. H.; Stangl, E.; Bentler, R. A.	2013
Evaluation of the preliminary auditory profile test battery in an international multi-centre study	Van Esch, T. E. M.; Kollmeier, B.; Vormann, M.; Lyzenga, J.; Houtgast, T.; Hallgren, M.; Larsby, B.; Athalye, S. P.; Lutman, M. E.; Dreschler, W. A.	2013
Acceptable noise level (ANL) and real-world hearing-aid success in Taiwanese listeners	Ho, H. C.; Wu, Y. H.; Hsiao, S. H.; Zhang, X.	2013
European multi-centre study of the Nucleus Hybrid L24 cochlear implant	Lenarz, T.; James, C.; Cuda, D.; Fitzgerald O'Connor, A.; Frachet, B.; Frijns, J. H.; Klenzner, T.; Laszig, R.; Manrique, M.; Marx, M.; Merkus, P.; Mylanus, E. A.; Offeciers, E.; Pesch, J.; Ramos-Macias, A.; Robier, A.; Sterkers, O.; Uziel, A.	2013

Otoacoustic emissions, pure-tone audiometry, and self-reported hearing	Engdahl, B.; Tambs, K.; Hoffman, H. J.	2013
Quality of life, effort and disturbance perceived in noise: A comparison between employees with aided hearing impairment and normal hearing	Hua, H.; Karlsson, J.; Widen, S.; Moller, C.; Lyxell, B.	2013
Speech recognition in noise using bilateral open-fit hearing aids: the limited benefit of directional microphones and noise reduction	Magnusson, L.; Claesson, A.; Persson, M.; Tengstrand, T.	2013
Hearing of 75-year old persons over three decades: Has hearing changed?	Rosenhall, U.; Moller, C.; Hederstierna, C.	2013
Effects of noise and working memory capacity on memory processing of speech for hearing-aid users	Ng, E. H. N.; Rudner, M.; Lunner, T.; Pedersen, M. S.; Ronnberg, J.	2013
The applicability of a speech-in-noise screening test in occupational hearing conservation	Leensen, M. C. J.; Dreschler, W. A.	2013
Auditory memory deficit in elderly people with hearing loss	Shahidipour, Z.; Geshani, A.; Jafari, Z.; Jalaie, S.; Khosravifard, E.	2013
Long-term speech perception in elderly cochlear implant users	Dillon, M. T.; Buss, E.; Adunka, M. C.; King, E. R.; Pillsbury, H. C., 3rd; Adunka, O. F.; Buchman, C. A.	2013
An energetic limit on spatial release from masking	Best, V.; Thompson, E. R.; Mason, C. R.; Kidd, G., Jr.	2013
Perception of vowels and prosody by cochlear implant recipients in noise	Van Zyl, M.; Hanekom, J. J.	2013
Working memory compensates for hearing related phonological processing deficit	Classon, E.; Rudner, M.; Ronnberg, J.	2013
Can audiometric results predict qualitative hearing improvements in bone-Anchored hearing aid recipients?	McNeil, M. L.; Gulliver, M.; Morris, D. P.; Makki, F. M.; Bance, M.	2013

Evaluation of antioxidant treatment in presbyacusis: prospective, placebo-controlled, double-blind, randomised trial	Polanski, J. F.; Cruz, O. L.	2013
Hearing preservation in patients with vestibular schwannoma treated with Gamma Knife surgery	Baschnagel, A. M.; Chen, P. Y.; Bojrab, D.; Pieper, D.; Kartush, J.; Didyuk, O.; Naumann, I. C.; Maitz, A.; Grills, I. S.	2013
Long-term hearing outcomes following stereotactic radiosurgery for vestibular schwannoma: Patterns of hearing loss and variables influencing audiometric decline	Carlson, M. L.; Jacob, J. T.; Pollock, B. E.; Neff, B. A.; Tombers, N. M.; Driscoll, C. L. W.; Link, M. J.	2013
Minimal effects of visual memory training on auditory performance of adult cochlear implant users	Oba, S. I.; Galvin, J. J., 3rd; Fu, Q. J.	2013
Exploring the limits of frequency lowering	Souza, P. E.; Arehart, K. H.; Kates, J. M.; Croghan, N. B.; Gehani, N.	2013
Auditory brainstem response to complex sounds predicts self-reported speech-in-noise performance	Anderson, S.; Parbery-Clark, A.; White-Schwoch, T.; Kraus, N.	2013
Cognitive abilities relate to self-reported hearing disability	Zekveld, A. A.; George, E. L.; Houtgast, T.; Kramer, S. E.	2013
Recognition of accented and unaccented speech in different maskers by younger and older listeners	Gordon-Salant, S.; Yeni-Komshian, G. H.; Fitzgibbons, P. J.; Cohen, J. I.; Waldroup, C.	2013
Unilateral and bilateral hearing aids, spatial release from masking and auditory acclimatization	Dawes, P.; Munro, K. J.; Kalluri, S.; Edwards, B.	2013
Predicting the effect of hearing loss and audibility on amplified speech reception in a multi-talker listening scenario	Woods, W. S.; Kalluri, S.; Pentony, S.; Nooraei, N.	2013
Reverberation suppression in cochlear implants using a blind channel-selection strategy	Hazrati, O.; Loizou, P. C.	2013

Vowel identification by cochlear implant users: contributions of static and dynamic spectral cues	Donaldson, G. S.; Rogers, C. L.; Cardenas, E. S.; Russell, B. A.; Hanna, N. H.	2013
Spatial release from masking as a function of the spectral overlap of competing talkers	Best, V.; Thompson, E. R.; Mason, C. R.; Kidd, G., Jr.	2013
Effect of spectral change enhancement for the hearing impaired using parameter values selected with a genetic algorithm	Chen, J.; Baer, T.; Moore, B. C.	2013
Behavioral measures of cochlear compression and temporal resolution as predictors of speech masking release in hearing-impaired listeners	Gregan, M. J.; Nelson, P. B.; Oxenham, A. J.	2013
An algorithm to improve speech recognition in noise for hearing-impaired listeners	Healy, E. W.; Yoho, S. E.; Wang, Y.; Wang, D.	2013
A beamformer post-filter for cochlear implant noise reduction	Hersbach, A. A.; Grayden, D. B.; Fallon, J. B.; McDermott, H. J.	2013
Recognition of speech in noise after application of time-frequency masks: dependence on frequency and threshold parameters	Sinex, D. G.	2013
Multichannel compression hearing aids: effect of channel bandwidth on consonant and vowel identification by hearing-impaired listeners	Strelcyk, O.; Li, N.; Rodriguez, J.; Kalluri, S.; Edwards, B.	2013
Compensation for channel interaction in a simultaneous cochlear implant coding strategy	Bader, P.; Kals, M.; Schatzer, R.; Griessner, A.; Zierhofer, C.	2013
Comparison of multichannel wide dynamic range compression and ChannelFree processing in open canal hearing instruments	Plyler, P. N.; Reber, M. B.; Kovach, A.; Galloway, E.; Humphrey, E.	2013
An initial-fit comparison of two generic hearing aid prescriptive methods (NAL-NL2 and CAM2) to individuals having mild to moderately severe high-frequency hearing loss	Johnson, E. E.	2013
Sentence recognition in noise and perceived benefit of noise reduction on the receiver and transmitter sides of a BICROS hearing aid	Oeding, K.; Valente, M.	2013
The effectiveness of the directional microphone in the Oticon Medical Ponto Pro in participants with unilateral sensorineural hearing loss	Oeding, K.; Valente, M.	2013

The effect of LACE DVD training in new and experienced hearing aid users	Olson, A. D.; Preminger, J. E.; Shinn, J. B.	2013
The contribution of a frequency-compression hearing aid to contralateral cochlear implant performance	Perreau, A. E.; Bentler, R. A.; Tyler, R. S.	2013
Efficacy of hearing-aid based telephone strategies for listeners with moderate-to-severe hearing loss	Picou, E. M.; Ricketts, T. A.	2013
The advantage of knowing the talker	Souza, P.; Gehani, N.; Wright, R.; McCloy, D.	2013
Reliability measure of a clinical test: Appreciation of Music in Cochlear Implantees (AMICI)	Cheng, M. Y.; Spitzer, J. B.; Shafiro, V.; Sheft, S.; Mancuso, D.	2013
Understanding excessive SNR loss in hearing-impaired listeners	Grant, K. W.; Walden, T. C.	2013
Factors influencing individual variation in perceptual directional microphone benefit	Keidser, G.; Dillon, H.; Convery, E.; Mejia, J.	2013
The effects of noise reduction technologies on the acceptance of background noise	Lowery, K. J.; Plyler, P. N.	2013
The effect of presentation level on normal-hearing and hearing-impaired listeners' acceptable speech and noise levels	Recker, K. L.; Edwards, B. W.	2013
Spectrotemporal modulation sensitivity as a predictor of speech intelligibility for hearing-impaired listeners	Bernstein, J. G.; Mehraei, G.; Shamma, S.; Gallun, F. J.; Theodoroff, S. M.; Leek, M. R.	2013
Effects of a transient noise reduction algorithm on speech understanding, subjective preference, and preferred gain	Korhonen, P.; Kuk, F.; Lau, C.; Keenan, D.; Schumacher, J.; Nielsen, J.	2013
Suprathreshold auditory processing and speech perception in noise: hearing-impaired and normal-hearing listeners	Summers, V.; Makashay, M. J.; Theodoroff, S. M.; Leek, M. R.	2013
The effect of hearing aid technologies on listening in an automobile	Wu, Y. H.; Stangl, E.; Bentler, R. A.; Stanziola, R. W.	2013
Evaluation of a new powerful bone-anchored hearing system: a comparison study	Bosman, A. J.; Snik, A. F.; Hol, M. K.; Mylanus, E. A.	2013

Identification of vowel length, word stress, and compound words and phrases by postlingually deafened cochlear implant listeners	Morris, D.; Magnusson, L.; Faulkner, A.; Jonsson, R.; Juul, H.	2013
Hearing loss and depression in older adults	Mener, D. J.; Betz, J.; Genther, D. J.; Chen, D.; Lin, F. R.	2013
The SoundBite hearing system: Patient-assessed safety and benefit study	Gurgel, R. K.; Shelton, C.	2013
Hearing preservation and speech perception outcomes with electric-acoustic stimulation after 12 months of listening experience	Adunka, O. F.; Dillon, M. T.; Adunka, M. C.; King, E. R.; Pillsbury, H. C.; Buchman, C. A.	2013
The addition of a contralateral routing of signals microphone to a unilateral cochlear implant system - A prospective study in speech outcomes	Arora, R.; Amoodi, H.; Stewart, S.; Friesen, L.; Lin, V.; Nedzelski, J.; Chen, J.	2013
Cochlear implantation in patients with vestibular schwannoma: a single United Kingdom center experience	Pai, I.; Dhar, V.; Kelleher, C.; Nunn, T.; Connor, S.; Jiang, D.; O'Connor, A. F.	2013
Differential cochlear implant outcomes in older adults	Roberts, D. S.; Lin, H. W.; Herrmann, B. S.; Lee, D. J.	2013
Comparison of intermittent intratympanic steroid injection and near-continual transtympanic steroid perfusion as salvage treatments for sudden sensorineural hearing loss	Chou, Y. F.; Chen, P. R.; Kuo, I. J.; Yu, S. H.; Wen, Y. H.; Wu, H. P.	2013
Monitoring the capacity of working memory: Executive control and effects of listening effort	Amichetti, N. M.; Stanley, R. S.; White, A. G.; Wingfield, A.	2013
The effect of intratympanic methylprednisolone and gentamicin injection on Meniere's disease	Gabra, N.; Saliba, I.	2013
Therapeutic efficacy of the combination of intratympanic methylprednisolone and oral steroid for idiopathic sudden deafness	Gundogan, O.; Pinar, E.; Imre, A.; Ozturkcan, S.; Cokmez, O.; Yigiter, A. C.	2013
Retrospective study comparing audiologic performance and surgeon learning curve between cochlear implant devices	Mandour, M.; Samy, R. N.; Houston, L.	2013

Binaural interlevel differences and alternate binaural loudness balance in cochlear implant recipients	Praetorius, M.; Khabas, R.; Hoth, S.	2013
Preliminary evaluation of a light-based contact hearing device for the hearing impaired	Fay, J. P.; Perkins, R.; Levy, S. C.; Nilsson, M.; Puria, S.	2013
Speech perception performance of double array multichannel cochlear implant users with standard and duplicated maps in each of the arrays	Bento, R. F.; Goffi-Gomez, M. V.; Tsuji, R. K.; Fonseca, A. C.; Ikari, L. S.; Brito Neto, R. V.	2013
The effects of residual hearing in traditional cochlear implant candidates after implantation with a conventional electrode	Cosetti, M. K.; Friedmann, D. R.; Zhu, B. Z.; Heman-Ackah, S. E.; Fang, Y.; Keller, R. G.; Shapiro, W. H.; Roland, J. T., Jr.; Waltzman, S. B.	2013
Outcomes after cochlear implantation for patients with single-sided deafness, including those with recalcitrant Meniere's disease	Hansen, M. R.; Gantz, B. J.; Dunn, C.	2013
Cochlear implants to treat deafness caused by vestibular schwannomas	Mukherjee, P.; Ramsden, J. D.; Donnelly, N.; Axon, P.; Saeed, S.; Fagan, P.; Irving, R. M.	2013
Hearing preservation surgery for cochlear implantation--hearing and quality of life after 2 years	Santa Maria, P. L.; Domville-Lewis, C.; Sucher, C. M.; Chester-Browne, R.; Atlas, M. D.	2013
A New Semi-Implantable Transcutaneous Bone Conduction Device: Clinical, Surgical, and Audiologic Outcomes in Patients With Congenital Ear Canal Atresia	Siegert, R.; Kanderske, J.	2013
Auditory profile in superficial siderosis of the central nervous system: a prospective study	Sydlowski, S. A.; Levy, M.; Hanks, W. D.; Clark, M. D.; Ackley, R. S.	2013
Evaluation of preoperative hearing-in-noise protocol for osseointegrated hearing implants	Whited, C. W.; Unrein, S. C.; Tucci, D. L.; Kaylie, D. M.	2013

Comparison of sound processing strategies for osseointegrated bone conduction implants in mixed hearing loss: multiple-channel nonlinear versus single-channel linear processing	Desmet, J. B.; Bosman, A. J.; Snik, A. F.; Lambrechts, P.; Hol, M. K.; Mylanus, E. A.; De Bodt, M.; Van de Heyning, P.	2013
First clinical experiences with a direct acoustic cochlear stimulator in comparison to preoperative fitted conventional hearing aids	Busch, S.; Kruck, S.; Spickers, D.; Leuwer, R.; Hoth, S.; Praetorius, M.; Plinkert, P. K.; Mojallal, H.; Schwab, B.; Maier, H.; Lenarz, T.	2013
Predictors of listening capabilities and patient satisfaction after stapes surgery in otosclerosis	Hazenberg, A. J.; Minovi, A.; Dazert, S.; Hoppe, F. F.	2013
Multicenter study with a direct acoustic cochlear implant	Lenarz, T.; Zwartenkot, J. W.; Stieger, C.; Schwab, B.; Mylanus, E. A.; Caversaccio, M.; Kompis, M.; Snik, A. F.; D'Hondt, C.; Mojallal, H.	2013
Hearing performance with 2 different high-power sound processors for osseointegrated auditory implants	Kurz, A.; Caversaccio, M.; Kompis, M.	2013
First European multicenter results with a new transcutaneous bone conduction hearing implant system: short-term safety and efficacy	Sprinzl, G.; Lenarz, T.; Ernst, A.; Hagen, R.; Wolf-Magele, A.; Mojallal, H.; Todt, I.; Mlynski, R.; Wolframm, M. D.	2013
Experience with the active middle ear implant in patients with moderate-to-severe mixed hearing loss: indications and results	de Abajo, J.; Sanhueza, I.; Giron, L.; Manrique, M.	2013
Intratympanic and systemic steroids for sudden hearing loss	Koltsidopoulos, P.; Bibas, A.; Sismanis, A.; Tzonou, A.; Seggas, I.	2013

Tinnitus in a single-sided deaf ear reduces speech reception in the nontinnitus ear	Mertens, G.; Kleine Punte, A.; De Ridder, D.; Van de Heyning, P.	2013
Stability of audiometric measures and challenges in long-term management of the elderly cochlear implant patient	Spitzer, J. B.; Cellum, I. P.; Bosworth, C.	2013
Comparison of speech discrimination in noise and directional hearing with 2 different sound processors of a bone-anchored hearing system in adults with unilateral severe or profound sensorineural hearing loss	Wesarg, T.; Aschendorff, A.; Laszig, R.; Beck, R.; Schild, C.; Hassepass, F.; Kroeger, S.; Hocke, T.; Arndt, S.	2013
Comparison of intratympanic steroid injection, hyperbaric oxygen and combination therapy in refractory sudden sensorineural hearing loss	Yang, C. H.; Wu, R. W.; Hwang, C. F.	2013
Long-term benefit and sound localization in patients with single-sided deafness rehabilitated with an osseointegrated bone-conduction device	Nicolas, S.; Mohamed, A.; Yoann, P.; Laurent, G.; Thierry, M.	2013
Neural adaptation and behavioral measures of temporal processing and speech perception in cochlear implant recipients	Zhang, F.; Benson, C.; Murphy, D.; Boian, M.; Scott, M.; Keith, R.; Xiang, J.; Abbas, P.	2013
Development and preliminary verification of a Mandarin-based hearing-aid fitting strategy	Lai, Y. H.; Liu, T. C.; Li, P. C.; Shih, W. T.; Young, S. T.	2013
Internet Video Telephony Allows Speech Reading by Deaf Individuals and Improves Speech Perception by Cochlear Implant Users	Mantokoudis, G.; Dahler, C.; Dubach, P.; Kompis, M.; Caversaccio, M. D.; Senn, P.	2013
Reversal of age-related neural timing delays with training	Anderson, S.; White-Schwoch, T.; Parbery-Clark, A.; Kraus, N.	2013
Effect of hospital noise on patients' ability to hear, understand, and recall speech	Pope, D. S.; Gallun, F. J.; Kampel, S.	2013
The impact of speech rate on sentence recognition by elderly individuals	Lessa, A. H.; Costa, M. J.	2013
Individual variability in unaided and aided measurement of the acceptable noise level	Eddins, D. A.; Arnold, M.; Klein, A.; Ellison, J.	2013

Individual variability in benefit from fixed and adaptive directional microphones	Galster, J. A.; Rodemerk, K. S.	2013
Relationships between self-report and cognitive measures of hearing aid outcome	Ng, E. H. N., Rudner, M., Lunner, T., & Rönnberg, J.	2013
Developing an appropriate digital hearing aid for low-resource countries: A case study	Israsena, P.; Isaradisaikul, S.; Noymai, A.; Boonyanukul, S.; Hemakom, A.; Chinnarat, C.; Navacharoen, N.; Lekagul, S.	2013
Psychoacoustic and phoneme identification measures in cochlear-implant and normal-hearing listeners	Goldsworthy, R. L.; Delhorne, L. A.; Braida, L. D.; Reed, C. M.	2013
The perception of telephone-processed speech by combined electric and acoustic stimulation	Hu, Y.; Tahmina, Q.; Runge, C.; Friedland, D. R.	2013
Laboratory and field study of the potential benefits of pinna cue-preserving hearing aids	Jensen, N. S.; Neher, T.; Laugesen, S.; Johannesson, R. B.; Kragelund, L.	2013
The relationship between hearing status and the participation in different categories of work: demographics	Stam, M.; Kostense, P. J.; Festen, J. M.; Kramer, S. E.	2013
Auditory and cognitive factors underlying individual differences in aided speech-understanding among older adults	Humes, Larry E; Kidd, Gary R; Lentz, Jennifer J %J Frontiers in systems neuroscience	2013
Predicting the Bilateral Advantage in Cochlear Implantees using a Non-Intrusive Speech Intelligibility Measure	Cosentino, S.; Falk, T. H.; McAlpine, D.; Bimbot, F.; Cerisara, C.; Fougeron, C.; Gravier, G.; Lamel, L.; Pellegrino, F.; Perrier, P.	2013

Active middle ear implantation for patients with sensorineural hearing loss and external otitis: long-term outcome in patient satisfaction	Zwartenkot, Joost W; Hashemi, Javad; Cremers, Cor WRJ; Mulder, Jef JS; Snik, Ad FM %J Otology; Neurotology	2013
Hearing preservation and clinical outcome of 32 consecutive electric acoustic stimulation (EAS) surgeries	Usami, S.; Moteki, H.; Tsukada, K.; Miyagawa, M.; Nishio, S. Y.; Takumi, Y.; Iwasaki, S.; Kumakawa, K.; Naito, Y.; Takahashi, H.; Kanda, Y.; Tono, T.	2014
Development and validation of a questionnaire for hearing implant users to self-assess their auditory abilities in everyday communication situations: the Hearing Implant Sound Quality Index (HISQUI(19))	Amann, E.; Anderson, I.	2014
Adaptation of cochlear implant fitting to various telecommunication systems: a proposal for a 'telephone map'	Giannantonio, S.; Di Nardo, W.; Schinaia, L.; Paludetti, G.	2014
Analysis of the performance of post-lingually deafened patients with nurotron VenusTM cochlear implants	Li, J.; Ji, F.; Chen, W.; Zhao, H.; Han, D.; Yang, S.	2014
Is there a deafness duration limit for cochlear implants in post-lingual deaf adults?	Moon, I. S.; Park, S.; Kim, H. N.; Lee, W. S.; Kim, S. H.; Kim, J. H.; Choi, J. Y.	2014
Quantitative enhancement of speech in noise through a wireless equipped hearing aid	Ciorba, A.; Zattara, S.; Loroni, G.; Prosser, S.	2014
Central auditory function in patients with mild alzheimer disease	Pai, M. C.; Chiu, C.; Wu, J. L.	2014
Validating the Thinking Styles Inventory-Revised II among Chinese university students with hearing impairment through test accommodations	Cheng, S.; Zhang, L. F.	2014
Better together: reduced compliance after sequential versus simultaneous bilateral hearing aids fitting	Lavie, L.; Banai, K.; Attias, J.; Karni, A.	2014
Self-Reported Spatial Hearing Abilities Across Different Cochlear Implant Profiles	Perreau, A. E.; Ou, H.; Tyler, R.; Dunn, C.	2014

Relating working memory to compression parameters in clinically fit hearing AIDS	Souza, P. E.; Sirow, L.	2014
Identification of acoustically similar and dissimilar vowels in profoundly deaf adults who use hearing aids and/or cochlear implants: some preliminary findings	Hay-McCutcheon, M. J.; Peterson, N. R.; Rosado, C. A.; Pisoni, D. B.	2014
The effect of visual cues on scoring of clinical word-recognition tests	Han, H. J.; Schlauch, R. S.; Rao, A.	2014
Hearing gain with a BAHA test-band in patients with single-sided deafness	Kim, D. Y.; Kim, T. S.; Shim, B. S.; Jin, I. S.; Ahn, J. H.; Chung, J. W.; Yoon, T. H.; Park, H. J.	2014
Cochlear implantation outcomes in advanced otosclerosis	Castillo, F.; Polo, R.; Gutierrez, A.; Reyes, P.; Royuela, A.; Alonso, A.	2014
Hearing loss and auditory processing ability in people with aphasia	Rankin, E.; Newton, C.; Parker, A.; Bruce, C.	2014
The prediction of disability by self-reported physical frailty components of the Tilburg Frailty Indicator (TFI)	Gobbens, R. J. J.; van Assen, Malm; Schalk, M. J. D.	2014
Clinical evaluation of an image-guided cochlear implant programming strategy	Noble, J. H.; Gifford, R. H.; Hedley-Williams, A. J.; Dawant, B. M.; Labadie, R. F.	2014
Benefits of cochlear implantation in deafened adults	Lachowska, M.; Pastuszka, A.; Glinka, P.; Niemczyk, K.	2014
Availability of binaural cues for bilateral implant recipients and bimodal listeners with and without preserved hearing in the implanted ear	Gifford, R. H.; Dorman, M. F.; Sheffield, S. W.; Teece, K.; Olund, A. P.	2014
The benefits of bimodal hearing: effect of frequency region and acoustic bandwidth	Sheffield, S. W.; Gifford, R. H.	2014
Improvement of telephone communication in elderly cochlear implant patients	Di Nardo, W.; Anzivino, R.; Gambini, G.; De Corso, E.; Paludetti, G.	2014

Quality of life outcomes for cochlear implantation in the elderly	Huarte, A.; Lezaun, R.; Manrique, M.	2014
Predictive factors of cochlear implant outcomes in the elderly	Mosnier, I.; Bebear, J. P.; Marx, M.; Fraysse, B.; Truy, E.; Lina-Granade, G.; Mondain, M.; Sterkers-Artieres, F.; Bordure, P.; Robier, A.; Godey, B.; Meyer, B.; Frachet, B.; Poncet, C.; Bouccara, D.; Sterkers, O.	2014
Speech performance and training effects in the cochlear implant elderly	Schumann, A.; Hast, A.; Hoppe, U.	2014
Baha-Mediated Rehabilitation of Patients with Unilateral Deafness: Selection Criteria	Saroul, N.; Akkari, M.; Pavier, Y.; Gilain, L.; Mom, T.	2014
A Comparative Study on Speech in Noise Understanding with a Direct Acoustic Cochlear Implant in Subjects with Severe to Profound Mixed Hearing Loss	Lenarz, T.; Verhaert, N.; Desloovere, C.; Desmet, J.; D'Hondt, C.; Gonzalez, J. C. F.; Kludt, E.; Macias, A. R.; Skarzynski, H.; Van de Heyning, P.; Vyncke, C.; Wasowski, A.	2014
Experiences and Results from Cochlear Implantation in Patients with Long Duration of Deafness	Lundin, K.; Stillesjo, F.; Rask-Andersen, H.	2014
Evaluation of temporal difference limen in preoperative non-invasive ear canal audiometry as a predictive factor for speech perception after cochlear implantation	Sinkkonen, S. T.; Kronlund, L.; Hautamaki, J.; Jero, J.; Aarnisalo, A. A.; Kentala, E.	2014
Screening of cognitive function and hearing impairment in older adults: A preliminary study	Wong, L. L. N.; Yu, J. K. Y.; Chan, S. S.; Tong, M. C. F.	2014
Development of a test of suprathreshold acuity in noise in Brazilian Portuguese: a new method for hearing screening and surveillance	Vaez, N.; Desgualdo-Pereira, L.; Paglialonga, A.	2014

Speech understanding with a new implant technology: a comparative study with a new nonskin penetrating Baha system	Kurz, A.; Flynn, M.; Caversaccio, M.; Kompis, M.	2014
Comparison of the benefits of cochlear implantation versus contra-lateral routing of signal hearing aids in adult patients with single-sided deafness: study protocol for a prospective within-subject longitudinal trial	Kitterick, P. T.; O'Donoghue, G. M.; Edmondson-Jones, M.; Marshall, A.; Jeffs, E.; Craddock, L.; Riley, A.; Green, K.; O'Driscoll, M.; Jiang, D.; Nunn, T.; Saeed, S.; Aleksy, W.; Seeber, B. U.	2014
Use of the 'patient journey' model in the internet-based pre-fitting counseling of a person with hearing disability: Lessons from a failed clinical trial	Manchaiah, V.; Ronnberg, J.; Andersson, G.; Lunner, T.	2014
Validation of the self-reported hearing questions in the Irish Longitudinal Study on Ageing against the Whispered Voice Test	Kenny Gibson, W.; Cronin, H.; Kenny, R. A.; Setti, A.	2014
An adapted adult day centre for older adults with sensory impairment	Wittich, W.; Murphy, C.; Mulrooney, D.	2014
Investigation of auditory thresholds in type 2 diabetic patients compared to non-diabetic cases	Kiakojouri, K.; Monadi, M.; Sheikhzadeh, M.; Omran, P. T.; Bayani, M. A.; Khafri, S.	2014
Advantages of Binaural Amplification to Acceptable Noise Level of Directional Hearing Aid Users	Kim, J. H.; Lee, J. H.; Lee, H. K.	2014
Electrophysiological signatures of plasticity in the visual and auditory cortex after cochlear implantation	Sandmann, P.; Plotz, K.; Schoenfeld, R.; Debener, S.	2014
An objective auditory measure to assess speech recognition in adult cochlear implant users	Turgeon, C.; Lazzouni, L.; Lepore, F.; Ellemberg, D.	2014
Patients with osteoporosis have higher incidence of sensorineural hearing loss	Kahveci, O. K.; Demirdal, U. S.; Yucedag, F.; Cerci, U.	2014

Audiological manifestations in HIV-positive adults	Matas, C. G.; Angrisani, R. G.; Magliaro, F. C.; Segurado, A. A.	2014
The experiences of adults assessed for cochlear implantation who did not proceed	Athalye, S.; Mulla, I.; Archbold, S.	2014
The role of post-operative imaging in cochlear implant surgery: a review of 220 adult cases	Coombs, A.; Clamp, P. J.; Armstrong, S.; Robinson, P. J.; Hajioff, D.	2014
Everyday listening questionnaire: correlation between subjective hearing and objective performance	Brendel, M.; Frohne-Buechner, C.; Lesinski-Schiedat, A.; Lenarz, T.; Buechner, A.	2014
Auditory rehabilitation after cochlear implantation in adults with hearing impairment after head trauma	Alves, M.; Martins, J. H.; Moura, J. E.; Ramos, D.; Alves, H.; Oliveira, G.; Magalhaes, I.; Silva, L.; Ribeiro, C.; Paiva, A. D.	2014
Is complex signal processing for bone conduction hearing aids useful?	Kompis, M.; Kurz, A.; Pfiffner, F.; Senn, P.; Arnold, A.; Caversaccio, M.	2014
FS4 for partial deafness treatment	Lorens, A.; Zgoda, M.; Polak, M.; Skarzynski, H.	2014
Sound localization with bilateral cochlear implants in noise: how much do head movements contribute to localization?	Mueller, M. F.; Meisenbacher, K.; Lai, W. K.; Dillier, N.	2014
Remote programming of cochlear implants	Samuel, P. A.; Goffi-Gomez, M. V.; Bittencourt, A. G.; Tsuji, R. K.; Brito, Rd	2014
A user-operated test of suprathreshold acuity in noise for adult hearing screening: The SUN (Speech Understanding in Noise) test	Paglialonga, A.; Tognola, G.; Grandori, F.	2014
Communicating with assistive listening devices and age-related hearing loss: Perceptions of older Australians	Aberdeen, L.; Fereiro, D.	2014

Acclimatization to hearing aids	Dawes, P.; Munro, K. J.; Kalluri, S.; Edwards, B.	2014
Benefits of phoneme discrimination training in a randomized controlled trial of 50- to 74-year-olds with mild hearing loss	Ferguson, M. A.; Henshaw, H.; Clark, D. P.; Moore, D. R.	2014
The effect of hearing aid noise reduction on listening effort in hearing-impaired adults	Desjardins, J. L.; Doherty, K. A.	2014
Potential benefits and limitations of three types of directional processing in hearing aids	Picou, E. M.; Aspell, E.; Ricketts, T. A.	2014
Psychometric properties of the self-efficacy for situational communication management questionnaire (SESMQ)	Jennings, M. B.; Cheesman, M. F.; Laplante-Levesque, A.	2014
Auditory impairments in HIV-infected individuals in Tanzania	Maro,, II; Moshi, N.; Clavier, O. H.; MacKenzie, T. A.; Kline-Schoder, R. J.; Wilbur, J. C.; Chambers, R. D.; Fellows, A. M.; Jastrzembski, B. G.; Mascari, J. E.; Bakari, M.; Matee, M.; Musiek, F. E.; Waddell, R. D.; von Reyn, C. F.; Buckey, J. C.	2014
Effects of unilateral input and mode of hearing in the better ear: self-reported performance using the speech, spatial and qualities of hearing scale	Dwyer, N. Y.; Firszt, J. B.; Reeder, R. M.	2014
Cochlear dead regions constrain the benefit of combining acoustic stimulation with electric stimulation	Zhang, T.; Dorman, M. F.; Gifford, R.; Moore, B. C.	2014
The effect of changing the secondary task in dual-task paradigms for measuring listening effort	Picou, E. M.; Ricketts, T. A.	2014
Measuring listening effort: driving simulator versus simple dual-task paradigm	Wu, Y. H.; Aksan, N.; Rizzo, M.; Stangl, E.; Zhang, X.; Bentler, R.	2014
Mitigation of informational masking in individuals with single-sided deafness by integrated bone conduction hearing aids	May, B. J.; Bowditch, S.; Liu, Y.; Eisen, M.; Niparko, J. K.	2014

Effects of frequency compression and frequency transposition on fricative and affricate perception in listeners with normal hearing and mild to moderate hearing loss	Alexander, J. M.; Kopun, J. G.; Stelmachowicz, P. G.	2014
Hearing in middle age: a population snapshot of 40- to 69-year olds in the United Kingdom	Dawes, P.; Fortnum, H.; Moore, D. R.; Emsley, R.; Norman, P.; Cruickshanks, K.; Davis, A.; Edmondson-Jones, M.; McCormack, A.; Lutman, M.; Munro, K.	2014
Validation of a clinical assessment of spectral-ripple resolution for cochlear implant users	Drennan, W. R.; Anderson, E. S.; Won, J. H.; Rubinstein, J. T.	2014
Competing speech perception in older and younger adults: behavioral and eye-movement evidence	Helfer, K. S.; Staub, A.	2014
Fundamental frequency information for speech recognition via bimodal stimulation: cochlear implant in one ear and hearing aid in the other	Shpak, T.; Most, T.; Luntz, M.	2014
Perceptual consequences of different signal changes due to binaural noise reduction: do hearing loss and working memory capacity play a role?	Neher, T.; Grimm, G.; Hohmann, V.	2014
Perceptions of age and brain in relation to hearing help-seeking and rehabilitation	Preminger, J. E.; Laplante-Levesque, A.	2014
FS4, FS4-p, and FSP: a 4-month crossover study of 3 fine structure sound-coding strategies	Riss, D.; Hamzavi, J. S.; Blineder, M.; Honeder, C.; Ehrenreich, I.; Kaider, A.; Baumgartner, W. D.; Gstoettner, W.; Arnoldner, C.	2014
Comparison between self-reported hearing and measured hearing thresholds of the elderly in China	Diao, M.; Sun, J.; Jiang, T.; Tian, F.; Jia, Z.; Liu, Y.; Chen, D.	2014

Hearing function in patients living with HIV/AIDS	Luque, A. E.; Orlando, M. S.; Leong, U. C.; Allen, P. D.; Guido, J. J.; Yang, H.; Wu, H.	2014
Do hearing loss and cognitive function modulate benefit from different binaural noise-reduction settings?	Neher, T.; Grimm, G.; Hohmann, V.; Kollmeier, B.	2014
Spatial separation benefit for unaided and aided listening	Ahlstrom, J. B.; Horwitz, A. R.; Dubno, J. R.	2014
Intelligibility of Emotional Speech in Younger and Older Adults	Dupuis, K.; Pichora-Fuller, M. K.	2014
Automated Screening for High-Frequency Hearing Loss	Vlaming, Msmg; MacKinnon, R. C.; Jansen, M.; Moore, D. R.	2014
Auditory disorders in patients with systemic lupus erythematosus: Relation to clinical parameters	Mokbel, A. N.; Hassan, S. Z.; Zohdi, M. I.; ElShennawy, A. M.	2014
Neutrophil-lymphocyte ratio as a new predictive and prognostic factor at the hearing loss of diabetic patients	Ulu, S.; Bucak, A.; Ulu, M. S.; Ahsen, A.; Duran, A.; Yucedag, F.; Aycicek, A.	2014
Long-term functional outcome and satisfaction of patients with an active middle ear implant for sensorineural hearing loss compared to a matched population with conventional hearing aids	Ihler, F.; Bewarder, J.; Blum, J.; Matthias, C.; Canis, M.	2014
The effects of cochlear implantation on quality of life in the elderly	Di Nardo, W.; Anzivino, R.; Giannantonio, S.; Schinaia, L.; Paludetti, G.	2014
Using whisper voice test for early detection of hearing loss in Galician goverment nursing homes	Vazquez, C.; Gigirey, L. M.; Del Oro, C. P.; Seoane, S.	2014
The effect of functional hearing loss and age on long- and short-term visuospatial memory: Evidence from the UK biobank resource	Ronnberg, J.; Hygge, S.; Keidser, G.; Rudner, M.	2014
Cognitive spare capacity in older adults with hearing loss	Mishra, S.; Stenfelt, S.; Lunner, T.; Ronnberg, J.; Rudner, M.	2014

Sensitivity to temporal fine structure and hearing-aid outcomes in older adults	Perez, E.; McCormack, A.; Edmonds, B. A.	2014
Decreased ability in the segregation of dynamically changing vowel-analog streams: A factor in the age-related cocktail-party deficit?	Divenyi, P.	2014
Relating hearing loss and executive functions to hearing aid users' preference for, and speech recognition with, different combinations of binaural noise reduction and microphone directionality	Neher, T.	2014
Impact of advanced hearing aid technology on speech understanding for older listeners with mild to moderate, adult-onset, sensorineural hearing loss	Cox, R. M.; Johnson, J. A.; Xu, J.	2014
The effects of noise vocoding on speech quality perception	Anderson, M. C.; Arehart, K. H.; Kates, J. M.	2014
Spatial and temporal modifications of multitalker speech can improve speech perception in older adults	Gygi, B.; Shafiro, V.	2014
Top-down restoration of speech in cochlear-implant users	Bhargava, P.; Gaudrain, E.; Baskent, D.	2014
Localization and interaural time difference (ITD) thresholds for cochlear implant recipients with preserved acoustic hearing in the implanted ear	Gifford, R. H.; Grantham, D. W.; Sheffield, S. W.; Davis, T. J.; Dwyer, R.; Dorman, M. F.	2014
Event-related potentials for better speech perception in noise by cochlear implant users	Soshi, T.; Hisanaga, S.; Kodama, N.; Kanekama, Y.; Samejima, Y.; Yumoto, E.; Sekiyama, K.	2014
The psychological process from avoidance to acceptance in adults with acquired hearing impairment	Wanstrom, G.; Oberg, M.; Rydberg, E.; Lunner, T.; Laplante-Levesque, A.; Andersson, G.	2014
Beta-band activity in auditory pathways reflects speech localization and recognition in bilateral cochlear implant users	Senkowski, D.; Pomper, U.; Fitzner, I.; Engel, A. K.; Kral, A.	2014

The Relationship Between Metabolic Presbycusis and Serum Paraoxonase/Arylesterase Activity	Keles, E.; Kapusuz, Z.; Guursu, M. F.; Karlidag, T.; Kaygusuz, I.; Bulmus, F. G.; Yalcin, S.	2014
Symptom Burden and Quality of Life in Advanced Head and Neck Cancer Patients: AIIMS Study of 100 Patients	Gandhi, A. K.; Roy, S.; Thakar, A.; Sharma, A.; Mohanti, B. K.	2014
Use of hearing aids and functional capacity in middle-aged and elderly individuals	Carioli, J.; Teixeira, A. R.	2014
Level of user satisfaction with hearing aids and environment: The international outcome inventory for hearing aids	Kozlowski, L.; Almeida, G.; Ribas, A.	2014
Influence of mild hearing loss on sustained auditory attention in the elderly	Jose, M. R.; Mondelli, M. F. C. G.; Feniman, M. R.	2014
Outcomes in patients with long time of hearing deprivation	Hoshino, A. C. H.; Dherte, A. T. M. M.; Gomez, M. V. S. G.; Reis, M.; Bento, R. F.; Tsuji, R. K.	2014
Results of speech recognition index done via live voice or recorded material in individuals with normal hearing and with sloping sensorineural hearing loss	Ribas, A.; Chiesorin, A.; Ataide, A.; Dandrea, K. K.	2014
Sun test: A new proposal on hearing screening for adults	Vaez, N.; Paglialonga, A.; Pereira, L. D.	2014
Factors associated with success with hearing aids in older adults	Hickson, L.; Meyer, C.; Lovelock, K.; Lampert, M.; Khan, A.	2014
Identifying the barriers and facilitators to optimal hearing aid self-efficacy	Meyer, C.; Hickson, L.; Fletcher, A.	2014
The ICF core sets for hearing loss project: Functioning and disability from the patient perspective	Granberg, S.; Pronk, M.; Swanepoel, D.; Kramer, S. E.; Hagsten, H.; Hjaldahl, J.; Moller, C.; Danermark, B.	2014

A factor analysis of the SSQ (Speech, Spatial, and Qualities of Hearing Scale)	Akeroyd, M. A.; Guy, F. H.; Harrison, D. L.; Suller, S. L.	2014
Conversation tactics in persons with normal hearing and hearing-impairment	Hallam, R. S.; Corney, R.	2014
The benefits of using bluetooth accessories with hearing aids	Smith, P.; Davis, A.	2014
Proposed norms for the Glasgow hearing-aid benefit profile (Ghabp) questionnaire	Whitmer, W. M.; Howell, P.; Akeroyd, M. A.	2014
An Australian version of the acceptable noise level test and its predictive value for successful hearing aid use in an older population	Walravens, E.; Keidser, G.; Hartley, D.; Hickson, L.	2014
Clinical assessment of spectral modulation detection for adult cochlear implant recipients: a non-language based measure of performance outcomes	Gifford, R. H.; Hedley-Williams, A.; Spahr, A. J.	2014
Benefit from non-linear frequency compression hearing aids in a clinical setting: the effects of duration of experience and severity of high-frequency hearing loss	Hopkins, K.; Khanom, M.; Dickinson, A. M.; Munro, K. J.	2014
Clinical evaluation of the Nucleus (R) 6 cochlear implant system: Performance improvements with SmartSound iQ	Mauger, S. J.; Warren, C. D.; Knight, M. R.; Goorevich, M.; Nel, E.	2014
Psychometric properties of a revised Danish translation of the international outcome inventory for hearing aids (IOI-HA)	Jespersen, C. T.; Bille, M.; Legarth, J. V.	2014
Role of slow temporal modulations in speech identification for cochlear implant users	Gnansia, D.; Lazard, D. S.; Leger, A. C.; Fugain, C.; Lancelin, D.; Meyer, B.; Lorenzi, C.	2014
Investigation of a matrix sentence test in noise: reproducibility and discrimination function in cochlear implant patients	Hey, M.; Hocke, T.; Hedderich, J.; Muller-Deile, J.	2014
Exploring the sensitivity of speech-in-noise tests for noise-induced hearing loss	Jansen, S.; Luts, H.; Dejonckere, P.; van Wieringen, A.; Wouters, J.	2014
Self assessment of hearing quality and noise-related attitudes among traffic policemen of Patiala, India	Gupta, S.; Mittal, S.; Kumar, A.; Singh, K. D.	2014

Diagnostic utility of Stenger test: Reappraisal of its value	Arslan, H. H.; Edizer, D. T.; Cebeci, S.; Erdal, M.	2014
Cochlear Implantation in Meniere's Disease	Fife, T. A.; Lewis, M. P.; May, J. S.; Oliver, E. R.	2014
Cigarette smoking, passive smoking, alcohol consumption, and hearing loss	Dawes, P.; Cruickshanks, K. J.; Moore, D. R.; Edmondson-Jones, M.; McCormack, A.; Fortnum, H.; Munro, K. J.	2014
Gender categorization is abnormal in cochlear implant users	Fuller, C. D.; Gaudrain, E.; Clarke, J. N.; Galvin, J. J.; Fu, Q. J.; Free, R. H.; Baskent, D.	2014
Examining the electro-neural interface of cochlear implant users using psychophysics, CT scans, and speech understanding	Long, C. J.; Holden, T. A.; McClelland, G. H.; Parkinson, W. S.; Shelton, C.; Kelsall, D. C.; Smith, Z. M.	2014
Hearing Loss in Older Persons: Does the Rate of Decline Affect Psychosocial Health?	Pronk, M.; Deeg, D. J. H.; Smits, C.; Twisk, J. W.; van Tilburg, T. G.; Festen, J. M.; Kramer, S. E.	2014
How difficult is difficult? Speech perception in noise in the elderly hearing impaired	Lavie, L.; Banai, K.; Attias, J.; Karni, A.	2014
Analysis of wave III of brain stem auditory evoked potential waveforms during microvascular decompression of cranial nerve VII for hemifacial spasm	Thirumala, P. D.; Krishnaiah, B.; Crammond, D. J.; Habeych, M. E.; Balzer, J. R.	2014
Relationship between cognitive anxiety level and client variables at initial consultation for adults with hearing impairment	Kelly-Campbell, R. J.; Parry, D. C.	2014
Hearing Loss Related with Type 2 Diabetes in an Elderly Population	Cayonu, M.; Capraz, M.; Acar, A.; Altundag, A.; Salihoglu, M.	2014

Speech Audiometry Tests in Noise Are Impaired in Older Patients with Mild Cognitive Impairment: A Pilot Study	Aimoni, C.; Prosser, S.; Ciorba, A.; Menozzi, L.; Soavi, C.; Zuliani, G.	2014
Digital processing technology for bone-anchored hearing aids: randomised comparison of two devices in hearing aid users with mixed or conductive hearing loss	Hill-Feltham, P.; Roberts, S. A.; Gladdis, R.	2014
Intratympanic steroid injection as a salvage treatment for sudden sensorineural hearing loss	Belhassen, S.; Saliba, I.	2014
Audiological evaluation in patients with Behcet's disease	Sonbay, N. D.; Saka, C.; Tatlican, S.; Vuralkan, E.; Aygener, N.; Eren, C.; Akin, I.	2014
Long-term follow up of sudden sensorineural hearing loss patients treated with intratympanic steroids: Audiological and quality of life evaluation	Dallan, I.; Fortunato, S.; Casani, A. P.; Bernardini, E.; Sellari-Franceschini, S.; Berrettini, S.; Nacci, A.	2014
An auditory profile of sclerosteosis	Potgieter, J. M.; Swanepoel, D. W.; Heinze, B. M.; Hofmeyr, L. M.; Burger, A. A. S.; Hamersma, H.	2014
Sensory impairments in community health care: A descriptive study of hearing and vision among elderly Norwegians living at home	Haanes, G. G.; Kirkevold, M.; Horgen, G.; Hofoss, D.; Eilertsen, G.	2014
Auditory brainstem implants in neurofibromatosis Type 2: is open speech perception feasible?	Matthies, C.; Brill, S.; Varallyay, C.; Solymosi, L.; Gelbrich, G.; Roosen, K.; Ernestus, R. I.; Helms, J.; Hagen, R.; Mlynski, R.; Shehata-Dieler, W.; Muller, J.	2014

Investigations into audiovestibular manifestations in patients with psoriatic arthritis	Amor-Dorado, J. C.; Barreira-Fernandez, M. P.; Pina, T.; Vazquez-Rodriguez, T. R.; Llorca, J.; Gonzalez-Gay, M. A.	2014
A longitudinal study in adults with sequential bilateral cochlear implants: time course for individual ear and bilateral performance	Reeder, R. M.; Firszt, J. B.; Holden, L. K.; Strube, M. J.	2014
Identification and multiplicity of double vowels in cochlear implant users	Kwon, B. J.; Perry, T. T.	2014
Do adults with cochlear implants rely on different acoustic cues for phoneme perception than adults with normal hearing?	Moberly, A. C.; Lowenstein, J. H.; Tarr, E.; Caldwell-Tarr, A.; Welling, D. B.; Shahin, A. J.; Nittrouer, S.	2014
Perception of a sung vowel as a function of frequency-modulation rate and excursion in listeners with normal hearing and hearing impairment	Vatti, M.; Santurette, S.; Pontoppidan, N. H.; Dau, T.	2014
Development of a self-report tool to evaluate hearing aid outcomes among Chinese speakers	Wong, L. L.; Hang, N.	2014
Head shadow, squelch, and summation effects with an energetic or informational masker in bilateral and bimodal CI users	Pyschny, V.; Landwehr, M.; Hahn, M.; Lang-Roth, R.; Walger, M.; Meister, H.	2014
Perception of speech produced by native and nonnative talkers by listeners with normal hearing and listeners with cochlear implants	Ji, C.; Galvin, J. J.; Chang, Y.; Xu, A.; Fu, Q. J.	2014
Validation of remote mapping of cochlear implants	Eikelboom, R. H.; Jayakody, D. M. P.; Swanepoel, D. W.; Chang, S.; Atlas, M. D.	2014
Stimulus and listener factors affecting age-related changes in competing speech perception	Helfer, K. S.; Freyman, R. L.	2014
Optimizing frequency-to-electrode allocation for individual cochlear implant users	Grasmeder, M. L.; Verschuur, C. A.; Batty, V. B.	2014
Effects of interferer facing orientation on speech perception by normal-hearing and hearing-impaired listeners	Strelcyk, O.; Pentony, S.; Kalluri, S.; Edwards, B.	2014

Evaluation of adaptive dynamic range optimization in adverse listening conditions for cochlear implants	Ali, H.; Hazrati, O.; Tobey, E. A.; Hansen, J. H.	2014
Auditory acclimatization and hearing aids: late auditory evoked potentials and speech recognition following unilateral and bilateral amplification	Dawes, P.; Munro, K. J.; Kalluri, S.; Edwards, B.	2014
Acoustic correlates of vowel intelligibility in clear and conversational speech for young normal-hearing and elderly hearing-impaired listeners	Ferguson, S. H.; Quene, H.	2014
Speech-cue transmission by an algorithm to increase consonant recognition in noise for hearing-impaired listeners	Healy, E. W.; Yoho, S. E.; Wang, Y. X.; Apoux, F.; Wang, D. L.	2014
The effects of dosage and duration of auditory training for older adults with hearing impairment	Humes, L. E.; Kinney, D. L.; Brown, S. E.; Kiener, A. L.; Quigley, T. M.	2014
A method for measuring the intelligibility of uninterrupted, continuous speech	MacPherson, A.; Akeroyd, M. A.	2014
Spectrotemporal modulation sensitivity for hearing-impaired listeners: Dependence on carrier center frequency and the relationship to speech intelligibility	Mehraei, G.; Gallun, F. J.; Leek, M. R.; Bernstein, J. G. W.	2014
Phoneme recognition in vocoded maskers by normal-hearing and aided hearing-impaired listeners	Phatak, S. A.; Grant, K. W.	2014
The influence of informational masking on speech perception and pupil response in adults with hearing impairment	Koelewijn, T.; Zekveld, A. A.; Festen, J. M.; Kramer, S. E.	2014
Prediction of consonant recognition in quiet for listeners with normal and impaired hearing using an auditory model	Jurgens, T.; Ewert, S. D.; Kollmeier, B.; Brand, T.	2014
A Danish open-set speech corpus for competing-speech studies	Nielsen, J. B.; Dau, T.; Neher, T.	2014
Characterizing the Speech Reception Threshold in hearing-impaired listeners in relation to masker type and masker level	Rhebergen, K. S.; Pool, R. E.; Dreschler, W. A.	2014
What Is Important for Hearing Aid Satisfaction? Application of the Expectancy-Disconfirmation Model	Meyer, C.; Hickson, L.; Khan, A.; Walker, D.	2014
Development of the Device-Oriented Subjective Outcome (DOSO) Scale	Cox, R. M.; Alexander, G. C.; Xu, J. J.	2014

Subjective assessment of cochlear implant users' signal-to-noise ratio requirements for different levels of wireless device usability	Julstrom, S.; Kozma-Spytek, L.	2014
Amplification for listeners with a moderately severe high-frequency hearing loss	Lau, C. C.; Kuk, F.; Keenan, D.; Schumacher, J.	2014
The role of spectral resolution, working memory, and audibility in explaining variance in susceptibility to temporal envelope distortion	Davies-Venn, E.; Souza, P.	2014
The Impact of Different Background Noises: Effects on Cognitive Performance and Perceived Disturbance in Employees with Aided Hearing Impairment and Normal Hearing	Hua, H.; Emilsson, M.; Kahari, K.; Widen, S.; Moller, C.; Lyxell, B.	2014
Directional benefit is present with audiovisual stimuli: Limiting ceiling effects	Aspell, E.; Picou, E.; Ricketts, T.	2014
The effects of aging on speech perception in noise: Comparison between normal-hearing and cochlear-implant listeners	Jin, S. H.; Liu, C.; Sladen, D. P.	2014
Normative data for the Maryland CNC Test	Mendel, L. L.; Mustain, W. D.; Magro, J.	2014
Effect of different signal-processing options on speech-in-noise recognition for Cochlear implant recipients with the Cochlear CP810 speech processor	Potts, L. G.; Kolb, K. A.	2014
Variables that influence the recognition performance of interrupted words: Rise-fall shape and temporal location of the interruptions	Wilson, R. H.	2014
Prediction of IOI-HA scores using speech reception thresholds and speech discrimination scores in quiet	Brannstrom, K. J.; Lantz, J.; Nielsen, L. H.; Olsen, S. O.	2014
Short- and long-term effects of the modified Swedish version of the Active Communication Education (ACE) program for adults with hearing loss	Oberg, M.; Bohn, T.; Larsson, U.	2014
A preliminary evaluation of the active communication education program in a sample of 87-year-old hearing impaired individuals	Oberg, M.; Bohn, T.; Larsson, U.; Hickson, L.	2014
Validation of a screening test of auditory function using the telephone	Williams-Sanchez, V.; McArdle, R. A.; Wilson, R. H.; Kidd, G. R.; Watson, C. S.; Bourne, A. L.	2014

Prevalence and correlates of hearing and visual impairments in European nursing homes: results from the SHELTER study	Yamada, Y.; Vlachova, M.; Richter, T.; Finne-Soveri, H.; Gindin, J.; van der Roest, H.; Denkinger, M. D.; Bernabei, R.; Onder, G.; Topinkova, E.	2014
Prevalence of communication disorders in HIV-infected adults	Kallail, K. J.; Downs, D.; Scherz, J.; Sweet, D.; Zackula, R. E.	2014
Correlation of otologic complaints in soldiers with speech disorders after traumatic brain injury	Dion, G. R.; Miller, C. L.; O'Connor, P. D.; Howard, N. S.	2014
Sensory Function: Insights From Wave 2 of the National Social Life, Health, and Aging Project	Pinto, J. M.; Kern, D. W.; Wroblewski, K. E.; Chen, R. C.; Schumm, L. P.; McClintock, M. K.	2014
Differences in perception of hearing handicap between cochlear implant users and their spouses	Mistry, D.; Ryan, J.; Maessen, H.; Bance, M.	2014
Impact of electrode design and surgical approach on scalar location and cochlear implant outcomes	Wanna, G. B.; Noble, J. H.; Carlson, M. L.; Gifford, R. H.; Dietrich, M. S.; Haynes, D. S.; Dawant, B. M.; Labadie, R. F.	2014
Mastoid cavity obliteration and Vibrant Soundbridge implantation for patients with mixed hearing loss	Ihler, F.; Kohler, S.; Meyer, A. C.; Blum, J.; Strenzke, N.; Matthias, C.; Canis, M.	2014
The impact of hearing loss in older adults: a tertiary care hospital based study	Shrestha, K. K.; Shah, S.; Malla, N. S.; Jha, A. K.; Joshi, R. R.; Rijal, A. S.; Dhungana, A.	2014
Auditory processing abilities of parkinson disease patients	Folmer, R.; Vachhani, J.	2014

Partial maintenance of auditory-based cognitive training benefits in older adults	Anderson, S.; White-Schwoch, T.; Choi, H. J.; Kraus, N.	2014
Significance of cochlear dose in the radiosurgical treatment of vestibular schwannoma: controversies and unanswered questions	Jacob, J. T.; Carlson, M. L.; Schiefer, T. K.; Pollock, B. E.; Driscoll, C. L.; Link, M. J.	2014
How to address the communication needs of older patients with hearing loss	Holmes, E.	2014
Vision impairment and dual sensory problems in middle age	Dawes, P.; Dickinson, C.; Emsley, R.; Bishop, P. N.; Cruickshanks, K. J.; Edmondson-Jones, M.; McCormack, A.; Fortnum, H.; Moore, D. R.; Norman, P.; Munro, K.	2014
Outcomes following Semicircular Canal Plugging	Goddard, J. C.; Wilkinson, E. P.	2014
Hearing aid use prevents and reverses loss of speech understanding in adults with hearing loss	Astrachan, D. I.; Davis, M.	2014
OCTO "Outcomes of cochlear implant for the octogenarians: audiologic and quality-of-life"	Cloutier, F.; Bussieres, R.; Ferron, P.; Cote, M.	2014
Influence of cochlear implant insertion depth on performance: a prospective randomized trial	Buchman, C. A.; Dillon, M. T.; King, E. R.; Adunka, M. C.; Adunka, O. F.; Pillsbury, H. C.	2014
A new comprehensive cochlear implant questionnaire for measuring quality of life after sequential bilateral cochlear implantation	King, N.; Nahm, E. A.; Liberatos, P.; Shi, Q.; Kim, A. H.	2014
Enhanced hearing in noise for cochlear implant recipients: clinical trial results for a commercially available speech-enhancement strategy	Koch, D. B.; Quick, A.; Osberger, M. J.; Saoji, A.; Litvak, L.	2014
Willingness-to-accept Gamma knife radiosurgery for tinnitus among career San Francisco firefighters	Pross, S. E.; Allen, C. A.; Hong, O. S.; Cheung, S. W.	2014

Cochlear implantation in patients with advanced Meniere's disease	Mick, P.; Amoodi, H.; Arnoldner, C.; Shipp, D.; Friesen, L.; Lin, V.; Nedzelski, J.; Chen, J.	2014
A prospective, multi-centered study of the treatment of idiopathic sudden sensorineural hearing loss with combination therapy versus high-dose prednisone alone: a 139 patient follow-up	Battaglia, A.; Lualhati, A.; Lin, H.; Burchette, R.; Cueva, R.	2014
New outcomes with auditory brainstem implants in NF2 patients	Behr, R.; Colletti, V.; Matthies, C.; Morita, A.; Nakatomi, H.; Dominique, L.; Darrouzet, V.; Brill, S.; Shehata-Dieler, W.; Lorens, A.; Skarzynski, H.	2014
Round window stimulation for conductive and mixed hearing loss	Dillon, M. T.; Tubbs, R. S.; Adunka, M. C.; King, E. R.; Hillman, T. A.; Adunka, O. F.; Chen, D. A.; Buchman, C. A.	2014
Revision stapedectomy with bone cement: are results comparable to those of standard techniques?	Hudson, S. K.; Gurgel, R. K.; Shelton, C.	2014
Utility of MRIs in adult cochlear implant evaluations	Jiang, Z. Y.; Odiase, E.; Isaacson, B.; Roland, P. S.; Kutz, J. W., Jr.	2014
Risk factors for loss of ipsilateral residual hearing after hybrid cochlear implantation	Kopelovich, J. C.; Reiss, L. A.; Oleson, J. J.; Lundt, E. S.; Gantz, B. J.; Hansen, M. R.	2014
Speech perception performance as a function of age at implantation among postlingually deaf adult cochlear implant recipients	Mahmoud, A. F.; Ruckenstein, M. J.	2014

Outcomes of cochlear reimplantation in adults	Mahtani, S.; Glynn, F.; Mawman, D. J.; O'Driscoll, M. P.; Green, K.; Bruce, I.; Freeman, S. R.; Lloyd, S. K.	2014
Comparing the performance plateau in adult cochlear implant patients using HINT and AzBio	Massa, S. T.; Ruckenstein, M. J.	2014
Cochlear implantation in Meniere's disease patients	McRackan, T. R.; Gifford, R. H.; Kahue, C. N.; Dwyer, R.; Labadie, R. F.; Wanna, G. B.; Haynes, D. S.; Bennett, M. L.	2014
Cochlear implantation versus auditory brainstem implantation in bilateral total deafness after head trauma: personal experience and review of the literature	Medina, M.; Di Lella, F.; Di Trapani, G.; Prasad, S. C.; Bacciu, A.; Aristegui, M.; Russo, A.; Sanna, M.	2014
Can unaided non-linguistic measures predict cochlear implant candidacy?	Shim, H. J.; Won, J. H.; Moon, I. J.; Anderson, E. S.; Drennan, W. R.; McIntosh, N. E.; Weaver, E. M.; Rubinstein, J. T.	2014
Efficacy and safety of AM-111 in the treatment of acute sensorineural hearing loss: a double-blind, randomized, placebo-controlled phase II study	Suckfuell, M.; Lisowska, G.; Domka, W.; Kabacinska, A.; Morawski, K.; Bodlaj, R.; Klimak, P.; Kostrica, R.; Meyer, T.	2014
Cochlear implantation in patients with chronic suppurative otitis media	Wong, M. C.; Shipp, D. B.; Nedzelski, J. M.; Chen, J. M.; Lin, V. Y.	2014
The role of age on cochlear implant performance, use, and health utility: a multicenter clinical trial	Zwolan, T. A.; Henion, K.; Segel, P.; Runge, C.	2014

From hearing with a cochlear implant and a contralateral hearing aid (CI/HA) to hearing with two cochlear implants (CI/CI): a within-subject design comparison	Luntz, M.; Egra-Dagan, D.; Attias, J.; Yehudai, N.; Most, T.; Shpak, T.	2014
Cochlear implantation in late-implanted prelingually deafened adults: changes in quality of life	Straatman, L. V.; Huinck, W. J.; Langereis, M. C.; Snik, A. F.; Mulder, J. J.	2014
Evaluation of the bimodal benefit in a large cohort of cochlear implant subjects using a contralateral hearing aid	Illg, A.; Bojanowicz, M.; Lesinski-Schiedat, A.; Lenarz, T.; Buchner, A.	2014
Relation between speech-in-noise threshold, hearing loss and cognition from 40-69 years of age	Moore, D. R.; Edmondson-Jones, M.; Dawes, P.; Fortnum, H.; McCormack, A.; Pierzycki, R. H.; Munro, K. J.	2014
Tinnitus and other auditory problems - occupational noise exposure below risk limits may cause inner ear dysfunction	Lindblad, A. C.; Rosenhall, U.; Olofsson, A.; Hagerman, B.	2014
Advanced beamformers for cochlear implant users: acute measurement of speech perception in challenging listening conditions	Buechner, A.; Dyballa, K. H.; Hehrmann, P.; Fredelake, S.; Lenarz, T.	2014
Mandarin speech perception in combined electric and acoustic stimulation	Li, Y.; Zhang, G.; Galvin, J. J., 3rd; Fu, Q. J.	2014
Speech-perception training for older adults with hearing loss impacts word recognition and effort	Kuchinsky, S. E.; Ahlstrom, J. B.; Cute, S. L.; Humes, L. E.; Dubno, J. R.; Eckert, M. A.	2014
Sensory impairments and their associations with functional disability in a sample of the oldest-old	Cimarolli, V. R.; Jopp, D. S.	2014
Working memory affects older adults' use of context in spoken-word recognition	Janse, E.; Jesse, A.	2014
Cochlear implantation in patients with chronic otitis media and radical mastoidectomy: A single-stage procedure	Piche, M.; Cote, M.; Philippon, D.; Laflamme, N.; Bussieres, R.	2014

Hearing loss within a marriage: Perceptions of the spouse with normal hearing	Govender, N. G.; Maistry, N.; Soomar, N.; Paken, J.	2014
Hypofractionated stereotactic radiotherapy of acoustic neuroma: volume changes and hearing results after 89-month median follow-up	Kranzinger, M.; Zehentmayr, F.; Fastner, G.; Oberascher, G.; Merz, F.; Nairz, O.; Rahim, H.; Sedlmayer, F.	2014
Cochlear implant programming: A global survey on the state of the art	Vaerenberg, B.; Smits, C.; De Ceulaer, G.; Zir, E.; Harman, S.; Jaspers, N.; Tam, Y.; Dillon, M.; Wesarg, T.; Martin-Bonniot, D.; Gartner, L.; Cozma, S.; Kosaner, J.; Prentiss, S.; Sasidharan, P.; Briaire, J. J.; Bradley, J.; Debruyne, J.; Hollow, R.; Patadia, R.; Mens, L.; Veekmans, K.; Greisiger, R.; Harboun-Cohen, E.; Borel, S.; Tavora-Vieira, D.; Mancini, P.; Cullington, H.; Ng, A. H. C.; Walkowiak, A.; Shapiro, W. H.; Govaerts, P. J.	2014
Speech perception in tones and noise via cochlear implants reveals influence of spectral resolution on temporal processing	Oxenham, A. J.; Kreft, H. A.	2014
Gated auditory speech perception in elderly hearing aid users and elderly normal-hearing individuals: effects of hearing impairment and cognitive capacity	Moradi, S.; Lidestam, B.; Hallgren, M.; Ronnberg, J.	2014

Dynamic relation between working memory capacity and speech recognition in noise during the first 6 months of hearing aid use	Ng, E. H.; Classon, E.; Larsby, B.; Arlinger, S.; Lunner, T.; Rudner, M.; Ronnberg, J.	2014
Effects of noise reduction on speech intelligibility, perceived listening effort, and personal preference in hearing-impaired listeners	Brons, I.; Houben, R.; Dreschler, W. A.	2014
Investigating interaural frequency-place mismatches via bimodal vowel integration	Guerit, F.; Santurette, S.; Chalupper, J.; Dau, T.	2014
Using prophylactic antioxidants to prevent noise-induced hearing damage in young adults: a protocol for a double-blind, randomized controlled trial	Gilles, A.; Ihtijarevic, B.; Wouters, K.; Van de Heyning, P.	2014
Adaptive expert system for digital hearing aids gain recommendations using linear and non linear prescriptive procedures	Rajkumar, S.; Muttan, S.; Pillai, B.; Jaya, V.; Vignesh, S. S.	2014
Hearing loss is associated with poorer ratings of patient,Äìphysician communication and healthcare quality	Mick, Paul; Foley, Danielle M; Lin, Frank R %J Journal of the American Geriatrics Society	2014
Association between hearing impairment and self,Äêreported difficulty in physical functioning	Chen, David S; Genther, Dane J; Betz, Joshua; Lin, Frank R %J Journal of the American Geriatrics Society	2014

Relationship of hearing loss and dementia: a prospective, population-based study	Gurgel, Richard K; Ward, P Daniel; Schwartz, Sarah; Norton, Maria C; Foster, Norman L; Tschanz, JoAnn T %J Otology; neurotology: official publication of the American Otological Society, American Neurotology Society European Academy of Otology; Neurotology	2014
Round window electrocochleography and speech perception outcomes in adult cochlear implant subjects: comparison with audiometric and biographical information	McClellan, Joseph H; Formeister, Eric J; Merwin III, William H; Dillon, Margaret T; Calloway, Nathan; Iseli, Claire; Buchman, Craig A; Fitzpatrick, Douglas C; Adunka, Oliver F %J Otology; Neurotology	2014
Cochleostomy versus round window insertions: influence on functional outcomes in electric-acoustic stimulation of the auditory system	Adunka, Oliver F; Dillon, Margaret T; Adunka, Marcia C; King, English R; Pillsbury, Harold C; Buchman, Craig A %J Otology; Neurotology	2014
Auditory function following post-dural puncture headache treated with epidural blood patch. A long-term follow-up	Darvish, B.; Dahlgren, G.; Irestedt, L.; Magnuson, A.; Moller, C.; Gupta, A.	2015

Cone beam computed tomography after round window vibroplasty: do the radiological findings match the auditory outcome?	Barbara, M.; Volpini, L.; Ciotti, M.; Filippi, C.; Covelli, E.; Monini, S.; D'Ambrosio, F.	2015
Sound quality in adult cochlear implant recipients using the HISQUI19	Mertens, G.; Kleine Punte, A.; De Bodt, M.; Van de Heyning, P.	2015
Sequential bilateral cochlear implantation improves working performance, quality of life, and quality of hearing	Harkonen, K.; Kivekas, I.; Rautiainen, M.; Kotti, V.; Sivonen, V.; Vasama, J. P.	2015
Which ear should we choose for cochlear implantation in the elderly: The poorer or the better? Audiometric outcomes, quality of sound, and quality-of-life results	Lassaletta, L.; Calvino, M.; Sanchez-Cuadrado, I.; Perez-Mora, R. M.; Gavilan, J.	2015
Clinical observation on hearing conditions of centenarians in northern district of China	Liu, C. Q.; Cheng, X. T.; Zhu, Y. H.; Shen, W. D.; Bian, B. W.; Cao, J. Y.; Zhai, S. Q.; Chen, C. X.; Yang, S. M.; Yuan, H. J.; Yu, N.	2015
Steroid-based treatments for patients with total sudden sensorineural hearing loss	Nakache, G.; Migirov, L.; Trommer, S.; Drendel, M.; Wolf, M.; Henkin, Y.	2015
Outcomes of consonant-vowel monosyllable perception and word recognition after cochlear implantation in elderly Japanese patients	Omichi, R.; Maeda, Y.; Sugaya, A.; Kataoka, Y.; Kariya, S.; Fukushima, K.; Nishizaki, K.	2015
Speech, Spatial and Qualities of Hearing Scale (SSQ) and Spatial Hearing Questionnaire (SHQ) Changes Over Time in Adults With Simultaneous Cochlear Implants	Zhang, J.; Tyler, R.; Ji, H. H.; Dunn, C.; Wang, N. Y.; Hansen, M.; Gantz, B.	2015
An Investigation of Measurement Equivalence in Hearing Response Scales: Refinement of a Questionnaire for Use in Hearing Screening	Chenault, M.; Anteunis, L.; Kremer, B.; Berger, M.	2015

Information Retention and Overload in First-Time Hearing Aid Users: An Interactive Multimedia Educational Solution	Ferguson, M.; Brandreth, M.; Brassington, W.; Wharrad, H.	2015
Older and younger adult cochlear implant users: speech recognition in quiet and noise, quality of life, and music perception	Sladen, D. P.; Zappler, A.	2015
Can a Remotely Delivered Auditory Training Program Improve Speech-in-Noise Understanding?	Abrams, H. B.; Bock, K.; Ireya, R. L.	2015
Influence of Test Condition on Speech Perception With Electric-Acoustic Stimulation	Dillon, M. T.; Buss, E.; Adunka, O. F.; Buchman, C. A.; Pillsbury, H. C.	2015
Speech Recognition Across the Life Span: Longitudinal Changes From Middle-Age to Older Adults	Dubno, J. R.	2015
A Speech Perception Test in Simulated Reverberation Conditions	Spitzer, J. B.; Sandridge, S. A.; Newman, C. W.; Sydlowski, S.; Ghent, R. M., Jr.	2015
Evaluation of Performance With an Adaptive Digital Remote Microphone System and a Digital Remote Microphone Audio-Streaming Accessory System	Wolfe, J.; Duke, M. M.; Schafer, E.; Jones, C.; Mulder, H. E.; John, A.; Hudson, M.	2015
Recovery of Hearing in Meniere's Disease after Antiviral Treatment	Gacek, R. R.	2015
Hearing in static unilateral vestibular schwannoma declines more than in the contralateral ear	Patel, N. B.; Nieman, C. L.; Redleaf, M.	2015
Contralateral routing of signal hearing aid versus transcutaneous bone conduction in single-sided deafness	Leterme, G.; Bernardeschi, D.; Bensemman, A.; Coudert, C.; Portal, J. J.; Ferrary, E.; Sterkers, O.; Vicaut, E.; Frachet, B.; Bozorg Grayeli, A.	2015
Quality of life and auditory performance in adults with asymmetric hearing loss	Vannson, N.; James, C.; Fraysse, B.; Strelnikov, K.; Barone, P.; Deguine, O.; Marx, M.	2015

Cochlear implants as a treatment option for unilateral hearing loss, severe tinnitus and hyperacusis	Ramos Macias, A.; Falcon Gonzalez, J. C.; Manrique, M.; Morera, C.; Garcia-Ibanez, L.; Cenjor, C.; Coudert-Koall, C.; Killian, M.	2015
Benefit of a contralateral routing of signal device for unilateral cochlear implant users	Weder, S.; Kompis, M.; Caversaccio, M.; Stieger, C.	2015
Bimodal Hearing Aid Retention after Unilateral Cochlear Implantation	Devocht, E. M.; George, E. L.; Janssen, A. M.; Stokroos, R. J.	2015
Long-term results of incus vibroplasty in patients with moderate-to-severe sensorineural hearing loss	Maier, H.; Hinze, A. L.; Gerdes, T.; Busch, S.; Salcher, R.; Schwab, B.; Lenarz, T.	2015
Medication Use in Adults with and without Hearing Impairment	Stam, M.; Spooren, A.; Merkus, P.; Festen, J. M.; Smits, C.; Kramer, S. E.	2015
The influence of cochlear implant electrode position on performance	van der Marel, K. S.; Briaire, J. J.; Verbist, B. M.; Muurling, T. J.; Frijns, J. H.	2015
Binaural integration of periodically alternating speech following cochlear implantation in subjects with profound sensorineural unilateral hearing loss	Wesarg, T.; Richter, N.; Hessel, H.; Gunther, S.; Arndt, S.; Aschendorff, A.; Laszig, R.; Hassepass, F.	2015
Binaural Auditory Outcomes in Patients with Postlingual Profound Unilateral Hearing Loss: 3 Years after Cochlear Implantation	Mertens, G.; Punte, A. K.; De Bodt, M.; Van de Heyning, P.	2015
Is electrode-modiolus distance a prognostic factor for hearing performances after cochlear implant surgery?	Esquia Medina, G. N.; Borel, S.; Nguyen, Y.; Ambert-Dahan, E.; Ferrary, E.; Sterkers, O.; Grayeli, A. B.	2015

Validation of the U-STARR with the AB-York crescent of sound, a new instrument to evaluate speech intelligibility in noise and spatial hearing skills	Smulders, Y. E.; Rinia, A. B.; Pourier, V. E. C.; Van Zon, A.; Van Zanten, G. A.; Stegeman, I.; Scherf, F. W. A. C.; Smit, A. L.; Topsakal, V.; Tange, R. A.; Grolman, W.	2015
Evaluation of a transient noise reduction algorithm in cochlear implant users	Dyballa, K. H.; Hehrmann, P.; Hamacher, V.; Nogueira, W.; Lenarz, T.; Buchner, A.	2015
One-year follow up of auditory performance in post-lingually deafened adults implanted with the Neurelec Digisonic (R) SP/Saphyr (R) Neo cochlear implant system	Borger, D.; Lina-Granade, G.; Verneyre, S.; Thai-Van, H.; Saai, S.; Hoen, M.; Gnansia, D.; Truy, E.	2015
Comparison of performance of transcranial contralateral routing of signal, pre-implanted trimmer digital and digital bone anchored hearing aid in adults with unilateral hearing loss	Devi, N.; Chatni, S.; Ramadevi, K. J. S.; Fakruddin, D. B.	2015
Speech intelligibility in various noise conditions with the nucleus 5 CP810 sound processor	Dillier, N.; Lai, W. K.	2015
Melodic Contour Training and Its Effect on Speech in Noise, Consonant Discrimination, and Prosody Perception for Cochlear Implant Recipients	Lo, C. Y.; McMahon, C. M.; Looi, V.; Thompson, W. F.	2015
Evaluating the prevalence of sensorineural hearing loss in chronic renal failure patients undergoing hemodialysis in Imam Khomeini Hospital, Ahvaz, 2012-2014	Sarafraz, M.; Musavi, S. S. B.; Araghi, S.; Manesh, S. A.	2015
CINGLE-trial: Cochlear implantation for siNGLE-sided deafness, a randomised controlled trial and economic evaluation	Peters, J. P. M.; Van Zon, A.; Smit, A. L.; Van Zanten, G. A.; De Wit, G. A.; Stegeman, I.; Grolman, W.	2015

Effects of congruent and incongruent visual cues on speech perception and brain activity in cochlear implant users	Song, J. J.; Lee, H. J.; Kang, H.; Lee, D. S.; Chang, S. O.; Oh, S. H.	2015
Hearing aid fitting in older persons with hearing impairment: the influence of cognitive function, age, and hearing loss on hearing aid benefit	Meister, H.; Rahlmann, S.; Walger, M.; Margolf-Hackl, S.; Kiesling, J.	2015
The impact of hearing loss on language performance in older adults with different stages of cognitive function	Lodeiro-Fernandez, L.; Lorenzo-Lopez, L.; Maseda, A.; Nunez-Naveira, L.; Rodriguez-Villamil, J. L.; Millan-Calenti, J. C.	2015
Music therapy as specific and complementary training for adults after cochlear implantation: A pilot study	Hutter, E.; Argstatter, H.; Grapp, M.; Plinkert, P. K.	2015
An aging concern: A retrospective study comparing the audiological and speech outcome measures along with the surgical and quality-of-life outcomes in a group of geriatric patients with those of an adult control group	Speers, A.; George, A.; Toner, J.	2015
Conversion of adult Nucleus 5 cochlear implant users to the Nucleus 6 system	De Ceulaer, G.; Swinnen, F.; Pascoal, D.; Philips, B.; Killian, M.; James, C.; Govaerts, P. J.; Dhooge, I.	2015
Comparison of eSRTs and comfort levels in users of Digisonic SP cochlear implants	Bergeron, F.; Hotton, M.	2015
Effects of loss of residual hearing on speech performance with the CI422 and the Hybrid-L electrode	Friedmann, D. R.; Peng, R.; Fang, Y.; McMenomey, S. O.; Roland, J. T.; Waltzman, S. B.	2015
Cochlear implantation in patients with Meniere's disease	Samy, R. N.; Houston, L.; Scott, M.; Choo, D. I.; Meinzen-Derr, J.	2015
Bimodal benefit depends on the performance difference between a cochlear implant and a hearing aid	Yoon, Y. S.; Shin, Y. R.; Gho, J. S.; Fu, Q. J.	2015

Evaluation of the 'Fitting to Outcomes eXpert' (FOX) with established cochlear implant users	Buechner, A.; Vaerenberg, B.; Gazibegovic, D.; Brendel, M.; De Ceulaer, G.; Govaerts, P.; Lenarz, T.	2015
Speech reception threshold benefits in cochlear implant users with an adaptive beamformer in real life situations	Geisler, G.; Arweiler, I.; Hehrmann, P.; Lenarz, T.; Hamacher, V.; Buchner, A.	2015
Deficits in auditory frequency discrimination and speech recognition in cochlear implant users	Turgeon, C.; Champoux, F.; Lepore, F.; Ellemberg, D.	2015
Study of the long-term effects of frequency compression by behavioral verbal tests in adults	Marchesin, V. C.; Iorio, M. C.	2015
Effect of sound amplification in speech perception in elderly with and without tinnitus	Araujo Td.e, M.; Iorio, M. C.	2015
Higher social distress and lower psycho-social wellbeing: examining the coping capacity and health of people with hearing impairment	Hogan, A.; Phillips, R. L.; Brumby, S. A.; Williams, W.; Mercer-Grant, C.	2015
From isolation and dependence to autonomy – expectations before and experiences after cochlear implantation in adult cochlear implant users and their significant others	Mäki-Torkko, E.M.; Vestergren, S.; Harder, H.; Lyxell, B.;	2015
Factors associated with the accuracy of subjective assessments of hearing impairment	Kamil, R. J.; Genther, D. J.; Lin, F. R.	2015
Effects of hearing loss on heart rate variability and skin conductance measured during sentence recognition in noise	Mackersie, C. L.; MacPhee, I. X.; Heldt, E. W.	2015
Relationship Among Signal Fidelity, Hearing Loss, and Working Memory for Digital Noise Suppression	Arehart, K.; Souza, P.; Kates, J.; Lunner, T.; Pedersen, M. S.	2015
Trimodal speech perception: how residual acoustic hearing supplements cochlear-implant consonant recognition in the presence of visual cues	Sheffield, B. M.; Schuchman, G.; Bernstein, J. G.	2015

A Retrospective Multicenter Study Comparing Speech Perception Outcomes for Bilateral Implantation and Bimodal Rehabilitation	Blamey, P. J.; Maat, B.; Baskent, D.; Mawman, D.; Burke, E.; Dillier, N.; Beynon, A.; Kleine-Punte, A.; Govaerts, P. J.; Skarzynski, P. H.; Huber, A. M.; Sterkers-Artieres, F.; Van de Heyning, P.; O'Leary, S.; Fraysse, B.; Green, K.; Sterkers, O.; Venail, F.; Skarzynski, H.; Vincent, C.; Truy, E.; Dowell, R.; Bergeron, F.; Lazard, D. S.	2015
Effects of WDRC release time and number of channels on output SNR and speech recognition	Alexander, J. M.; Masterson, K.	2015
Electrophysiology and Perception of Speech in Noise in Older Listeners: Effects of Hearing Impairment and Age	Billings, C. J.; Penman, T. M.; McMillan, G. P.; Ellis, E. M.	2015
Cortical Auditory Evoked Potentials Recorded From Nucleus Hybrid Cochlear Implant Users	Brown, C. J.; Jeon, E. K.; Chiou, L. K.; Kirby, B.; Karsten, S. A.; Turner, C. W.; Abbas, P. J.	2015
Extended High-Frequency Bandwidth Improves Speech Reception in the Presence of Spatially Separated Masking Speech	Levy, S. C.; Freed, D. J.; Nilsson, M.; Moore, B. C.; Puria, S.	2015
Pitch adaptation patterns in bimodal cochlear implant users: over time and after experience	Reiss, L. A.; Ito, R. A.; Eggleston, J. L.; Liao, S.; Becker, J. J.; Lakin, C. E.; Warren, F. M.; McMenomey, S. O.	2015
Relationships Among Peripheral and Central Electrophysiological Measures of Spatial and Spectral Selectivity and Speech Perception in Cochlear Implant Users	Scheperle, R. A.; Abbas, P. J.	2015

Application of Noise Reduction Algorithm ClearVoice in Cochlear Implant Processing: Effects on Noise Tolerance and Speech Intelligibility in Noise in Relation to Spectral Resolution	Dingemanse, J. G.; Goedegebure, A.	2015
Deterioration of Speech Recognition Ability Over a Period of 5 Years in Adults Ages 18 to 70 Years: Results of the Dutch Online Speech-in-Noise Test	Stam, M.; Smits, C.; Twisk, J. W.; Lemke, U.; Festen, J. M.; Kramer, S. E.	2015
Stages of change in adults who have failed an online hearing screening	Laplante-Levesque, A.; Brannstrom, K. J.; Ingo, E.; Andersson, G.; Lunner, T.	2015
Noise reduction improves memory for target language speech in competing native but not foreign language speech	Ng, E. H.; Rudner, M.; Lunner, T.; Ronnberg, J.	2015
Speech prosody perception in cochlear implant users with and without residual hearing	Marx, M.; James, C.; Foxton, J.; Capber, A.; Fraysse, B.; Barone, P.; Deguine, O.	2015
Self-Reported Hearing Difficulties Among Adults With Normal Audiograms: The Beaver Dam Offspring Study	Tremblay, K. L.; Pinto, A.; Fischer, M. E.; Klein, B. E. K.; Klein, R.; Levy, S.; Tweed, T. S.; Cruickshanks, K. J.	2015
Clinical outcomes for adult cochlear implant recipients experiencing loss of usable acoustic hearing in the implanted ear	Plant, K. L.; van Hoesel, R. J.; McDermott, H. J.; Dawson, P. W.; Cowan, R. S.	2015
Social Support Predicts Hearing Aid Satisfaction	Singh, G.; Lau, S. T.; Pichora-Fuller, M. K.	2015
Effects of a Dual Sensory Loss Protocol on Hearing Aid Outcomes: A Randomized Controlled Trial	Vreeken, H. L.; van Rens, Ghmb; Kramer, S. E.; Knol, D. L.; van Nispen, R. M. A.	2015
Exploring the Effects of the Narrative Embodied in the Hearing Aid Fitting Process on Treatment Outcomes	Naylor, G.; Oberg, M.; Wanstrom, G.; Lunner, T.	2015

Amplitude Modulation Detection and Speech Recognition in Late-Implanted Prelingually and Postlingually Deafened Cochlear Implant Users	De Ruiter, A. M.; Debruyne, J. A.; Chenault, M. N.; Francart, T.; Brokx, J. P. L.	2015
Otolith function assessment in patients with systemic sclerosis	El-Wakd, M. M.; El-Gazzar, I. I.; Hosni, N. A.; El-Din Teleb, D. A. G.	2015
Quality of life in bimodal hearing users (unilateral cochlear implants and contralateral hearing aids)	Farinetti, A.; Roman, S.; Mancini, J.; Baumstarck-Barrau, K.; Meller, R.; Lavieille, J. P.; Triglia, J. M.	2015
Cochlear implantation in patients with Cogan syndrome: long-term results	Bacciu, A.; Pasanisi, E.; Di Lella, F.; Guida, M.; Bacciu, S.; Vincenti, V.	2015
Delaying partial stapedectomy for otosclerosis: effects on long-term hearing outcomes following surgery	Maniakas, A.; Nehme, J.; Dufour, J. J.; Saliba, I.	2015
Quality of life and audiologic performance through the ability to phone of cochlear implant users	Rumeau, C.; Frere, J.; Montaut-Verient, B.; Lion, A.; Gauchard, G.; Parietti-Winkler, C.	2015
Clinical effectiveness of wireless CROS (contralateral routing of offside signals) hearing aids	Ryu, N. G.; Moon, I. J.; Byun, H.; Jin, S. H.; Park, H.; Jang, K. S.; Cho, Y. S.	2015
Use of data mining to predict significant factors and benefits of bilateral cochlear implantation	Ramos-Miguel, A.; Perez-Zaballos, T.; Perez, D.; Falconb, J. C.; Ramosb, A.	2015

Results in using the Freiburger monosyllabic speech test in noise without and with hearing aids	Lohler, J.; Akcicek, B.; Wollenberg, B.; Schonweiler, R.; Verges, L.; Langer, Ch; Machate, U.; Noppeney, R.; Schultz, K.; Kleeberg, J.; Junge-Hulsing, B.; Walther, L. E.; Schlattmann, P.; Ernst, A.	2015
Efficacy of auditory training in elderly subjects	Morais, A. A.; Rocha-Muniz, C. N.; Schochat, E.	2015
Lexical influences on spoken spondaic word recognition in hearing-impaired patients	Moulin, A.; Richard, C.	2015
Auditory training can improve working memory, attention, and communication in adverse conditions for adults with hearing loss	Ferguson, M. A.; Henshaw, H.	2015
The relationship of speech intelligibility with hearing sensitivity, cognition, and perceived hearing difficulties varies for different speech perception tests	Heinrich, A.; Henshaw, H.; Ferguson, M. A.	2015
The benefit of amplification on auditory working memory function in middle-aged and young-older hearing impaired adults	Doherty, K. A.; Desjardins, J. L.	2015
Associations between speech understanding and auditory and visual tests of verbal working memory: effects of linguistic complexity, task, age, and hearing loss	Smith, S. L.; Pichora-Fuller, M. K.	2015
Working memory and intelligibility of hearing-aid processed speech	Souza, P. E.; Arehart, K. H.; Shen, J.; Anderson, M.; Kates, J. M.	2015
How does susceptibility to proactive interference relate to speech recognition in aided and unaided conditions ?	Ellis, R. J.; Ronnberg, J.	2015
Walking speed is associated with self-perceived hearing handicap in high-functioning older adults: The Fujiwara-kyo study	Tomioka, K.; Harano, A.; Hazaki, K.; Morikawa, M.; Iwamoto, J.; Saeki, K.; Okamoto, N.; Kurumatani, N.	2015
Hearing Aid Use in Everyday Life: Managing Contextual Variability	Williger, B.; Lang, F. R.	2015

Evaluation of speech perception via the use of hearing loops and telecoils	Alfakir, R.; Holmes, A. E.; Kricos, P. B.; Gaeta, L.; Martin, S.	2015
Prevalence of hearing loss among Canadians aged 20 to 79: Audiometric results from the 2012/2013 Canadian Health Measures Survey	Feder, K.; Michaud, D.; Ramage-Morin, P.; McNamee, J.; Beauregard, Y.	2015
Factors constraining the benefit to speech understanding of combining information from low-frequency hearing and a cochlear implant	Dorman, M. F.; Cook, S.; Spahr, A.; Zhang, T.; Loiselle, L.; Schramm, D.; Whittingham, J.; Gifford, R.	2015
Effects of steep high-frequency hearing loss on speech recognition using temporal fine structure in low-frequency region	Li, B.; Hou, L.; Xu, L.; Wang, H.; Yang, G.; Yin, S.; Feng, Y.	2015
Speech perception with interaction-compensated simultaneous stimulation and long pulse durations in cochlear implant users	Schatzer, R.; Koroleva, I.; Griessner, A.; Levin, S.; Kusovkov, V.; Yanov, Y.; Zierhofer, C.	2015
Cochlear implantation outcomes in older adults	Castiglione, A.; Benatti, A.; Girasoli, L.; Caserta, E.; Montino, S.; Pagliaro, M.; Bovo, R.; Martini, A.	2015
Link between angiographic extent and severity of coronary artery disease and degree of sensorineural hearing loss	Erkan, A. F.; Beriat, G. K.; Ekici, B.; Dogan, C.; Kocaturk, S.; Tore, H. F.	2015
Satisfaction with use of hearing aids and speech perception test in subjects with sensorineural bilateral hearing loss	De Marchi Dos Santos, M.; Mariano, T. C. B.; Jose, M. R.; Mondelli, M. F. C.	2015

Using data mining to predict bilateral implantation outcomes [General]	Perez Zaballos, M. T.; Ramos De Miguel, A.; Perez Plasencia, D.; Falcon Gonzalez, J. C.; Ramos Maclas, A.	2015
Transcutaneous osseointegrated implant system with magnetic holding without pedestal. Results in our Center [otology, neuro-otology and skull base surgery]	Borkoski Barreiro, S. A.; Perez Plasencia, D.; Cuyas Lazarich, J. M.; Falcon Gonzalez, J. C.; Ramos Maclas, A.	2015
Cochlear implant in far advanced otosclerosis-performance-complications-long term results [otology, neuro-otology and skull base surgery]	Curet, C.; Salvadores, M.; Romani, C.; Rubino, L.; Robledo, H.; Muino, J. C.; Ruiz, H.; Dotto, G.; Queirolo, A.	2015
The influence of the minimum current levels on the hearing thresholds and on the speech recognition in cochlear implant users [Audiology]	Martins, K. V. C.; Magalhaes, A. T. M.; Porto, B. L.; Goffi-Gomez, M. V. S.; Tsuji, R. K.	2015
Benefits of active middle ear implant vibrant soundbridge in sensorineural hearing loss [Audiology]	Miranda, C.; Silva, V.; Peixoto, C.	2015
Cochlear implant intraoperative telemetry are important? [otology, neuro-otology and skull base surgery]	Muruyama, N. C.; Damico, T. A.; Massuda, E. T.; Hyppolito, M. A.	2015
Comparative study between the auditory findings in individuals with and without diabetes mellitus [Audiology]	Nunes, D. P. D. R. M.; Sanders, A. P.; Vieira, L. F.; Maruiti, G.	2015
Experience with the new SAMBA audio processor for the Vibrant Soundbridge [otology, neuro-otology and skull base surgery]	Ruiz De Erenchun, I.; Boleas, M. S.; Ansorena, P.; Bulnes, M. D.; Del Carmen, M.	2015
Audiometric thresholds and speech perception sentence test outcomes in adults and elderly after cochlear implantation [otology, neuro-otology and skull base surgery]	Santos, M. D.; Guimaraes, A. C.; De Carvalho, G. M.; Rocha, V. B. C.; Castilho, A. M.	2015

Dichotic sentence identification test in elderly	Schwantes, A. L.; Peixe, B. P.; Vellozo, F. F.; Martins, Q. P.; Sanguebuche, T. R.; Garcia, M. V.	2015
Auditory deprivation in monitoring audiological ten elderly patient with sensorineural hearing loss in over six years [audiology]	Silveira, A. L.; Teixeira, A.; Paniagua, L. M.; Faria, M. R. D.	2015
Two dichotic tests comparison in elderly	Vellozo, F. F.; Martins, Q. P.; Schwantes, A. L.; Sanguebuche, T. R.; Garcia, M. V.; Peixe, B. P.	2015
The effects of unilateral adaptation of hearing AIDS on symptoms of depression and social activity constraints of elderly	Dos Santos, F. D.; Teixeira, A. R.	2015
Hearing loss and cognitive effects in sentence recognition in aged	Sanguebuche, T. R.; Da Silva, D. D.; Pinheiro, M. M. C.; Bruckmann, M.; Bruno, R. S.	2015
Radiological and audiological aspects of postlingual patients with meningitis submitted to cochlear implant [otology, neuro-otology and skull base surgery]	Damico, T. A.; Oliveira, A. A.; Isaac, M. L.; Hyppolito, M. A.; Massuda, E. T.	2015
Changes in hearing thresholds in cancer treatment priscila feliciano de oliveira	De Lima, E. C. O.; De Oliveira Barreto, A. C.; Habib, N. C. S.; De Jesus, V. M.; Magna, C.	2015
Hearing-aid use and long-term health outcomes: Hearing handicap, mental health, social engagement, cognitive function, physical health, and mortality	Dawes, P.; Cruickshanks, K. J.; Fischer, M. E.; Klein, B. E.; Klein, R.; Nondahl, D. M.	2015
An evaluation of the performance of two binaural beamformers in complex and dynamic multitalker environments	Best, V.; Mejia, J.; Freeston, K.; van Hoesel, R. J.; Dillon, H.	2015

On the relationship between functional hearing and depression	Keidser, G.; Seeto, M.; Rudner, M.; Hygge, S.; Ronnberg, J.	2015
Hearing aid and hearing assistance technology use in Aotearoa/New Zealand	Kelly-Campbell, R. J.; Lessoway, K.	2015
Benefit from, and acclimatization to, frequency compression hearing aids in experienced adult hearing-aid users	Ellis, R. J.; Munro, K. J.	2015
Functional performance in older adults with hearing loss: Application of the International Classification of Functioning brief core set for hearing loss: A pilot study	Alfakir, R.; Holmes, A. E.; Noreen, F.	2015
Predictors of aided speech recognition, with and without frequency compression, in older adults	Ellis, R. J.; Munro, K. J.	2015
An examination of speech reception thresholds measured in a simulated reverberant cafeteria environment	Best, V.; Keidser, G.; Buchholz, J. M.; Freeston, K.	2015
The NTID speech recognition test: NSRT()	Bochner, J. H.; Garrison, W. M.; Doherty, K. A.	2015
Applications of the NTID speech recognition test (NSRT())	Garrison, W. M.; Bochner, J. H.	2015
The interaction of hearing loss and level-dependent hearing protection on speech recognition in noise	Giguere, C.; Laroche, C.; Vaillancourt, V.	2015
Evaluation of the effects of nonlinear frequency compression on speech recognition and sound quality for adults with mild to moderate hearing loss	Picou, E. M.; Marcrum, S. C.; Ricketts, T. A.	2015
Recognition performance on words interrupted (10 ips, 50% duty cycle) with two interruption patterns referenced to word onset: Young listeners with normal hearing for pure tones and older listeners with sensorineural hearing loss	Wilson, R. H.; Irish, S. E.	2015
Relations between psychophysical measures of spatial hearing and self-reported spatial-hearing abilities	Van Esch, T. E. M.; Lutman, M. E.; Vormann, M.; Lyzenga, J.; Hallgren, M.; Larsby, B.; Athalye, S. P.; Houtgast, T.; Kollmeier, B.; Dreschler, W. A.	2015
Wireless and acoustic hearing with bone-anchored hearing devices	Bosman, A. J.; Mylanus, E. A.; Hol, M. K.; Snik, A. F.	2015
Validation of a French translation of the Speech, Spatial, and Qualities of Hearing Scale (SSQ) and comparison with other language versions	Moulin, A.; Pauzie, A.; Richard, C.	2015

Hearing-related, health-related quality of life in patients who have undergone otosclerosis surgery: a long-term follow-up study	Redfors, Y. D.; Olaison, S.; Karlsson, J.; Hellgren, J.; Moller, C.	2015
Cross-cultural adaptation of an Arabic version of the 10-item hearing handicap inventory	Weinstein, B. E.; Rasheedy, D.; Taha, H. M.; Fatouh, F. N.	2015
Sex-specific predictors of hearing-aid use in older persons: The age, gene/environment susceptibility - Reykjavik study	Fisher, D. E.; Li, C. M.; Hoffman, H. J.; Chiu, M. S.; Themann, C. L.; Petersen, H.; Jonsson, P. V.; Jonsson, H.; Jonasson, F.; Sverrisdottir, J. E.; Launer, L. J.; Eiriksdottir, G.; Gudnason, V.; Cotch, M. F.	2015
Assessing speech recognition abilities with digits in noise in cochlear implant and hearing aid users	Kaandorp, M. W.; Smits, C.; Merkus, P.; Goverts, S. T.; Festen, J. M.	2015
Characteristics and international comparability of the Finnish matrix sentence test in cochlear implant recipients	Dietz, A.; Buschermohle, M.; Sivonen, V.; Willberg, T.; Aarnisalo, A. A.; Lenarz, T.; Kollmeier, B.	2015
Retrospective audiological analysis of bone conduction versus round window vibratory stimulation in patients with mixed hearing loss	Mojallal, H.; Schwab, B.; Hinze, A. L.; Giere, T.; Lenarz, T.	2015
Computer-based auditory phoneme discrimination training improves speech recognition in noise in experienced adult cochlear implant listeners	Schumann, A.; Serman, M.; Gefeller, O.; Hoppe, U.	2015

The safety and efficacy of PF-04958242 in age-related sensorineural hearing loss a randomized clinical trial	Bednar, M. M.; De Martinis, N.; Banerjee, A.; Bowditch, S.; Gaudreault, F.; Zumpano, L.; Lin, F. R.	2015
Improvement of cognitive function after cochlear implantation in elderly patients	Mosnier, I.; Bebear, J. P.; Marx, M.; Fraysse, B.; Truy, E.; Lina-Granade, G.; Mondain, M.; Sterkers-Artieres, F.; Bordure, P.; Robier, A.; Godey, B.; Meyer, B.; Frachet, B.; Poncet-Wallet, C.; Bouccara, D.; Sterkers, O.	2015
Correlations Between Pitch and Phoneme Perception in Cochlear Implant Users and Their Normal Hearing Peers	Goldsworthy, R. L.	2015
Error Patterns Analysis of Hearing Aid and Cochlear Implant Users as a Function of Noise	Chun, H.; Ma, S.; Han, W.; Chun, Y.	2015
Functional assessment of elderly patients with hearing impairment: A preliminary evaluation	Sogebi, O. A.; Oluwole, L. O.; Mabifah, T. O.	2015
Understanding Language, Hearing Status, and Visual-Spatial Skills	Marschark, M.; Spencer, L. J.; Durkin, A.; Borgna, G.; Convertino, C.; Machmer, E.; Kronenberger, W. G.; Trani, A.	2015
Long-term outcome of cochlear implant in patients with chronic otitis media: one-stage surgery is equivalent to two-stage surgery	Jang, J. H.; Park, M. H.; Song, J. J.; Lee, J. H.; Oh, S. H.; Kim, C. S.; Chang, S. O.	2015
Utility of electronystagmography in the prediction of post-operative outcome following cochlear implantation	Spitzer, J. B.; Chari, D.; Machmer, E.; Lipson, S.; Rouse, L.; Lalwani, A. K.	2015

Cortical Activity Predicts Which Older Adults Recognize Speech in Noise and When	Vaden, K. I.; Kuchinsky, S. E.; Ahlstrom, J. B.; Dubno, J. R.; Eckert, M. A.	2015
Influence of Tinnitus on Auditory Spectral and Temporal Resolution and Speech Perception in Tinnitus Patients	Moon, I. J.; Won, J. H.; Kang, H. W.; Kim, D. H.; An, Y. H.; Shim, H. J.	2015
Improved hearing in noise using new signal processing algorithms with the Cochlear Nucleus 6 sound processor	Gilden, J.; Lewis, K.; Grant, G.; Crosson, J.	2015
The Effects of Linguistic Context on Word Recognition in Noise by Elderly Listeners Using Spanish Sentence Lists (SSL)	Cervera, T.; Rosell, V.	2015
Auditory difficulties in blast-exposed Veterans with clinically normal hearing	Saunders, G. H.; Frederick, M. T.; Arnold, M.; Silverman, S.; Chisolm, T. H.; Myers, P.	2015
Environmental sound training in cochlear implant users	Shafiro, V.; Sheft, S.; Kuvadia, S.; Gygi, B.	2015
Hearing Aid-Induced Plasticity in the Auditory System of Older Adults: Evidence From Speech Perception	Lavie, L.; Banai, K.; Karni, A.; Attias, J.	2015
Acoustical and Perceptual Comparison of Noise Reduction and Compression in Hearing Aids	Brons, I.; Houben, R.; Dreschler, W. A.	2015
The Effect of Age on Listening Effort	Degeest, S.; Keppler, H.; Corthals, P.	2015
Auditory Spatial Deficits in the Early Stage of Ischemic Cerebral Stroke	Przewozny, T.; Gojska-Grymajlo, A.; Gasecki, D.	2015
Effects of age and hearing loss on the intelligibility of interrupted speech	Shafiro, V.; Sheft, S.; Risley, R.; Gygi, B.	2015
Effects of age and hearing loss on recognition of unaccented and accented multisyllabic words	Gordon-Salant, S.; Yeni-Komshian, G. H.; Fitzgibbons, P. J.; Cohen, J. I.	2015
Lexical influences on competing speech perception in younger, middle-aged, and older adults	Helfer, K. S.; Jesse, A.	2015
Better-ear glimpsing in hearing-impaired listeners	Best, V.; Mason, C. R.; Kidd, G., Jr.; Iyer, N.; Brungart, D. S.	2015

Comparing auditory filter bandwidths, spectral ripple modulation detection, spectral ripple discrimination, and speech recognition: Normal and impaired hearing	Davies-Venn, E.; Nelson, P.; Souza, P.	2015
Sentence intelligibility during segmental interruption and masking by speech-modulated noise: Effects of age and hearing loss	Fogerty, D.; Ahlstrom, J. B.; Bologna, W. J.; Dubno, J. R.	2015
Effect of audibility on spatial release from speech-on-speech masking	Glyde, H.; Buchholz, J. M.; Nielsen, L.; Best, V.; Dillon, H.; Cameron, S.; Hickson, L.	2015
An algorithm to increase speech intelligibility for hearing-impaired listeners in novel segments of the same noise type	Healy, E. W.; Yoho, S. E.; Chen, J.; Wang, Y.; Wang, D.	2015
Cochlear implant users' spectral ripple resolution	Jeon, E. K.; Turner, C. W.; Karsten, S. A.; Henry, B. A.; Gantz, B. J.	2015
Evaluation of a spectral subtraction strategy to suppress reverberant energy in cochlear implant devices	Kokkinakis, K.; Runge, C.; Tahmina, Q.; Hu, Y.	2015
Neural-scaled entropy predicts the effects of nonlinear frequency compression on speech perception	Rallapalli, V. H.; Alexander, J. M.	2015
Using speech sounds to test functional spectral resolution in listeners with cochlear implants	Winn, M. B.; Litovsky, R. Y.	2015
Age and hearing loss and the use of acoustic cues in fricative categorization	Scharenborg, O.; Weber, A.; Janse, E.	2015
Speech perception at positive signal-to-noise ratios using adaptive adjustment of time compression	Schlueter, A.; Brand, T.; Lemke, U.; Nitzschner, S.; Kollmeier, B.; Holube, I.	2015
What Can We Learn about Auditory Processing from Adult Hearing Questionnaires?	Bamiou, D. E.; Iliadou, V. V.; Zanchetta, S.; Spyridakou, C.	2015
The Effect of a High Upper Input Limiting Level on Word Recognition in Noise, Sound Quality Preferences, and Subjective Ratings of Real-World Performance	Oeding, K.; Valente, M.	2015
Comparison of Multichannel Wide Dynamic Range Compression and ChannelFree Processing Strategies on Consonant Recognition	Plyler, P.; Hedrick, M.; Rinehart, B.; Tripp, R.	2015

Evaluation of a BICROS System with a Directional Microphone in the Receiver and Transmitter	Valente, M.; Oeding, K.	2015
Construct Validity of the Ecological Momentary Assessment in Audiology Research	Wu, Y. H.; Stangl, E.; Zhang, X.; Bentler, R. A.	2015
Benefits of Nonlinear Frequency Compression in Adult Hearing Aid Users	Kokx-Ryan, M.; Cohen, J.; Cord, M. T.; Walden, T. C.; Makashay, M. J.; Sheffield, B. M.; Brungart, D. S.	2015
Discrimination of Stochastic Frequency Modulation by Cochlear Implant Users	Sheft, S.; Cheng, M. Y.; Shafiro, V.	2015
Speech Recognition at the Acceptable Noise Level	Gordon-Hickey, S.; Morlas, H.	2015
The Effects of Audiovisual Stimulation on the Acceptance of Background Noise	Plyler, P. N.; Lang, R.; Monroe, A. L.; Gaudiano, P.	2015
The homogeneity with respect to intelligibility of recorded word-recognition materials	Wilson, R. H.; McArdle, R.	2015
Speech Perception Ability in Noise is Correlated with Auditory Brainstem Response Wave I Amplitude	Bramhall, N.; Ong, B.; Ko, J.; Parker, M.	2015
Comparison of Clinical and Traditional Gap Detection Tests	Hoover, E.; Pasquesi, L.; Souza, P.	2015
Cochlear implant microphone location affects speech recognition in diffuse noise	Kolberg, E. R.; Sheffield, S. W.; Davis, T. J.; Sunderhaus, L. W.; Gifford, R. H.	2015
Speech intelligibility benefits of hearing AIDS at various input levels	Kuk, F.; Lau, C. C.; Korhonen, P.; Crose, B.	2015
The Benefit of Remote Microphones Using Four Wireless Protocols	Rodemerk, K. S.; Galster, J. A.	2015
Improving Hearing Performance for Cochlear Implant Recipients with Use of a Digital, Wireless, Remote-Microphone, Audio-Streaming Accessory	Wolfe, J.; Morais, M.; Schafer, E.	2015
Perception of Hearing Aid-Processed Speech in Individuals with Late-Onset Auditory Neuropathy Spectrum Disorder.[Erratum appears in J Am Acad Audiol. 2016 Feb;27(2):157; PMID: 26905534]	Mathai, J. P.; Appu, S.	2015

Self-Reported Hearing Status Is Associated with Lower Limb Physical Performance, Perceived Mobility, and Activities of Daily Living in Older Community-Dwelling Men and Women	Mikkola, T. M.; Polku, H.; Portegijs, E.; Rantakokko, M.; Rantanen, T.; Viljanen, A.	2015
Self-Reported Hearing Loss, Hearing Aids, and Cognitive Decline in Elderly Adults: A 25-Year Study	Amieva, H.; Ouvrard, C.; Giulioli, C.; Meillon, C.; Rullier, L.; Dartigues, J. F.	2015
The effect of hearing on cognitive evaluation in patients with age-related macular degeneration	Chandramohan, A.; Wright, J.; Duong Fernandez, X.; Zhuang, J.; Lad, E.; Cousins, S.; Madden, D.; Piker, E.; Tucci, D.; Whitson, H.	2015
Bedside testing for auditive impairment in the elderly	Lerch, M.	2015
Self-Reported Hearing Loss Predicts 5-Year Decline in Higher-Level Functional Capacity in High-Functioning Elderly Adults: The Fujiwara-Kyo Study	Tomioka, K.; Okamoto, N.; Morikawa, M.; Kurumatani, N.	2015
Effectiveness of oral zinc supplementation in the treatment of idiopathic sudden sensorineural hearing loss (ISSNHL)	Hunchaisri, N.; Chantapant, S.; Sirirattanapan, J.	2015
Study of hearing aid effectiveness and patient satisfaction	Kayabasoglu, G.; Kaymaz, R.; Erkorkmaz, U.; Guven, M.	2015
Etiological classification of presbycusis in Turkish population according to audiogram configuration	Kaya, K. H.; Karaman Koc, A.; Sayin, I.; Gunes, S.; Canpolat, S.; Simsek, B.; Kayhan, F. T.	2015
The role of a new contralateral routing of signal microphone in established unilateral cochlear implant recipients	Grewal, A. S.; Kuthubutheen, J.; Smilsky, K.; Nedzelski, J. M.; Chen, J. M.; Friesen, L.; Lin, V. Y. W.	2015

Bone-anchored hearing implants in single-sided deafness patients: Long-term use and satisfaction by gender	Faber, H. T.; Nelissen, R. C.; Kramer, S. E.; Cremers, C. W. R. J.; Snik, A. F. M.; Hol, M. K. S.	2015
Is human T-lymphotropic virus type 1 infection associated with hearing loss?	Bakhshaee, M.; Sorouri, A.; Shoeibi, A.; Boustani, R.; Golhasani-Keshtan, F.; Amali, A.; Rajati, M.	2015
Clinical and psychosocial risk factors of hearing outcome in older adults with cochlear implants	Francis, H. W.; Yeagle, J. A.; Thompson, C. B.	2015
Successful treatment of sudden sensorineural hearing loss assures improvement of accompanying tinnitus	Rah, Y. C.; Park, K. T.; Yi, Y. J.; Seok, J.; Kang, S. I.; Kim, Y. H.	2015
Cross-modal reorganization in cochlear implant users: Auditory cortex contributes to visual face processing	Stropahl, M.; Plotz, K.; Schonfeld, R.; Lenarz, T.; Sandmann, P.; Yovel, G.; De Vos, M.; Debener, S.	2015
Long-term Auditory Symptoms in Patients With Sporadic Vestibular Schwannoma: An International Cross-Sectional Study	Tveiten, O. V.; Carlson, M. L.; Goplen, F.; Vassbotn, F.; Link, M. J.; Lund-Johansen, M.	2015
Hearing loss associated with US military combat deployment	Wells, T. S.; Seelig, A. D.; Ryan, M. A. K.; Jones, J. M.; Hooper, T. I.; Jacobson, I. G.; Boyko, E. J.	2015

Quality of life and hearing after cochlear implant placement in patients over 60 years of age	Czerniejewska-Wolska, H.; Kalos, M.; Sekula, A.; Piszczatowski, B.; Rutkowska, J.; Rogowski, M.; Zadrozniak, M.; Szymanski, M.; Klatka, J.; Durko, M.; Pietruszewska, W.; Gawlowska, M. B.; Kusmierczyk, J.; Kruk-Krzemien, A.; Wiskirska-Woznica, B.	2015
Quality of Life (QoL) Assessment in Patients with Neurofibromatosis Type 2 (NF2)	Cosetti, M. K.; Golfinos, J. G.; Roland, J. T., Jr.	2015
Hearing Outcomes after Revision Stapedectomy Managed with Total Ossicular Prostheses	Lupo, J. E.; Strickland, B. M.; House, J. W.	2015
Endolymphatic duct blockage: a randomized controlled trial of a novel surgical technique for Meniere's disease treatment	Saliba, I.; Gabra, N.; Alzahrani, M.; Berbiche, D.	2015
Long-term results and prognostic factors in adult cochlear implants	Anderson, S.; Dornhoffer, J.; Cox, M. D.; Moore, P.	2015
Initial UK Experience With a Novel Magnetic Transcutaneous Bone Conduction Device	Carr, S. D.; Moraleda, J.; Procter, V.; Wright, K.; Ray, J.	2015
Temporal Cortical Plasticity in Single-Sided Deafness: A Functional Imaging Study	Pross, S. E.; Chang, J. L.; Mizuiri, D.; Findlay, A. M.; Nagarajan, S. S.; Cheung, S. W.	2015
Dose Effect of Intratympanic Dexamethasone for Idiopathic Sudden Sensorineural Hearing Loss: 24 mg/mL Is Superior to 10 mg/mL	Alexander, T. H.; Harris, J. P.; Nguyen, Q. T.; Vorasubin, N.	2015
Straight versus modiolar hugging electrodes: does one perform better than the other?	Doshi, J.; Johnson, P.; Mawman, D.; Green, K.; Bruce, I. A.; Freeman, S.; Lloyd, S. K.	2015

Outcomes of cochlear implantation in adults with asymmetric hearing loss	Franko-Tobin, E.; Camilon, P. R.; Camposeo, E.; Holcomb, M. A.; Meyer, T. A.	2015
Audiometry-Based Screening Procedure for Cochlear Implant Candidacy	Hoppe, U.; Hast, A.; Hocke, T.	2015
Decisive criteria between stapedotomy and cochlear implantation in patients with far advanced otosclerosis	Kabbara, B.; Gauche, C.; Calmels, M. N.; Lepage, B.; Escude, B.; Deguine, O.; Fraysse, B.; Marx, M.	2015
Does Coupling and Positioning in Vibroplasty Matter? A Prospective Cohort Study	Marino, R.; Lampacher, P.; Dittrich, G.; Tavora-Vieira, D.; Kuthubutheen, J.; Rajan, G. P.	2015
Clinical performance of a new magnetic bone conduction hearing implant system: results from a prospective, multicenter, clinical investigation	Briggs, R.; Van Hasselt, A.; Luntz, M.; Goycoolea, M.; Wigren, S.; Weber, P.; Smeds, H.; Flynn, M.; Cowan, R.	2015
Hearing performance in single-sided deaf cochlear implant users after upgrade to a single-unit speech processor	Mertens, G.; Hofkens, A.; Punte, A. K.; De Bodt, M.; Van de Heyning, P.	2015
Comparison of audiologic results and patient satisfaction for two osseointegrated bone conduction devices: results of a prospective study	Busch, S.; Giere, T.; Lenarz, T.; Maier, H.	2015
Transcutaneous Bone-anchored Hearing Aids Versus Percutaneous Ones: Multicenter Comparative Clinical Study	Iseri, M.; Orhan, K. S.; Tuncer, U.; Kara, A.; Durgut, M.; Guldiken, Y.; Surmelioglu, O.	2015
Pros and Cons of Round Window Vibroplasty in Open Cavities: Audiological, Surgical, and Quality of Life Outcomes	Lassaletta, L.; Calvino, M.; Sanchez-Cuadrado, I.; Perez-Mora, R. M.; Munoz, E.; Gavilan, J.	2015

Radiologic Results and Hearing Preservation With a Straight Narrow Electrode via Round Window Versus Cochleostomy Approach at Initial Activation	Hassepass, F.; Aschendorff, A.; Bulla, S.; Arndt, S.; Maier, W.; Laszig, R.; Beck, R.	2015
Speech Perception of Elderly Cochlear Implant Users Under Different Noise Conditions	Hast, A.; Schlucker, L.; Digeser, F.; Liebscher, T.; Hoppe, U.	2015
Hearing preservation and improved speech perception with a flexible 28-mm electrode	Helbig, S.; Helbig, M.; Leinung, M.; Stover, T.; Baumann, U.; Rader, T.	2015
Relationship between auditory and cognitive abilities in older adults	Sheft, S.; Shafiro, V.; Wang, E.; Barnes, L. L.; Shah, R. C.	2015
Contributions of electric and acoustic hearing to bimodal speech and music perception	Crew, J. D.; Galvin, J. J., 3rd; Landsberger, D. M.; Fu, Q. J.	2015
Functional changes in the human auditory cortex in ageing	Profant, O.; Tintera, J.; Balogova, Z.; Ibrahim, I.; Jilek, M.; Syka, J.	2015
Aided and unaided speech perception by older hearing impaired listeners	Woods, D. L.; Arbogast, T.; Doss, Z.; Younus, M.; Herron, T. J.; Yund, E. W.	2015
Speech perception in older hearing impaired listeners: benefits of perceptual training	Woods, D. L.; Doss, Z.; Herron, T. J.; Arbogast, T.; Younus, M.; Ettlinger, M.; Yund, E. W.	2015
Design and evaluation of a cochlear implant strategy based on a "Phantom" channel	Nogueira, W.; Litvak, L. M.; Saoji, A. A.; Buchner, A.	2015
Impact of a moving noise masker on speech perception in cochlear implant users	Weissgerber, T.; Rader, T.; Baumann, U.	2015

Spectrotemporal Modulation Detection and Speech Perception by Cochlear Implant Users	Won, J. H.; Moon, I. J.; Jin, S.; Park, H.; Woo, J.; Cho, Y. S.; Chung, W. H.; Hong, S. H.	2015
Open fitting: performance verification of receiver in the ear and receiver in the aid	Mondelli, M. F.; Garcia, T. M.; Hashimoto, F. M.; Rocha, A. V.	2015
Evaluation of Speech-Perception Training for Hearing Aid Users: A Multisite Study in Progress	Miller, J. D.; Watson, C. S.; Dubno, J. R.; Leek, M. R.	2015
The Effects of Meaning-Based Auditory Training on Behavioral Measures of Perceptual Effort in Individuals with Impaired Hearing	Sommers, M. S.; Tye-Murray, N.; Barcroft, J.; Spehar, B. P.	2015
Factors Contributing to Speech Performance in Elderly Cochlear Implanted Patients: An FDG-PET Study: A Preliminary Study	Suh, M. W.; Park, K. T.; Lee, H. J.; Lee, J. H.; Chang, S. O.; Oh, S. H.	2015
Sensorineural hearing loss in non-depressed essential tremor cases and controls: A clinical and audiometric study	Helvaci Yilmaz, N.; Cenk Akbostanci, M.; Yilmaz, N.	2015
Native and Non-native Speech Perception by Hearing-Impaired Listeners in Noise- and Speech Maskers	Kilman, L.; Zekveld, A.; Hallgren, M.; Ronnberg, J.	2015
Relations Between the Intelligibility of Speech in Noise and Psychophysical Measures of Hearing Measured in Four Languages Using the Auditory Profile Test Battery	Van Esch, T. E. M.; Dreschler, W. A.	2015
Comparing Binaural Pre-processing Strategies III: Speech Intelligibility of Normal-Hearing and Hearing-Impaired Listeners	Volker, C.; Warzybok, A.; Ernst, S. M.	2015
Enlargement of the Internal Auditory Canal and Hearing Preservation in the Middle Fossa Approach for Intracanalicular Vestibular Schwannomas	Aihara, N.; Murakami, S.	2015
Speech-in-speech listening on the LiSN-S test by older adults with good audiograms depends on cognition and hearing acuity at high frequencies	Besser, Jana; Festen, Joost M; Goverts, S Theo; Kramer, Sophia E; Pichora-Fuller, M Kathleen %J Ear; hearing	2015

Robust relationship between reading span and speech recognition in noise	Souza, Pamela; Arehart, Kathryn %J International journal of audiology	2015
Intelligibility Enhancement of Vocal Announcements for Public Address Systems: A design For All through a Presbycusis Pre-compensation Filter	Ben Jemaa, A.; Mechergui, N.; Courtois, G.; Mudry, A.; Djaziri-Larbi, S.; Turki, M.; Lissek, H.; Jaidane, M.; Isca-Int Speech Commun, Assoc	2015
Iranian Version of Speech, Spatial, and Qualities of Hearing Scale: A Psychometric Study	Lotfi, Y.; Nazeri, A. R.; Asgari, A.; Moosavi, A.; Bakhshi, E.	2016
Cochlear function tests in estimation of speech dynamic range	Han, J. J.; Park, S. Y.; Park, S. N.; Na, M. S.; Lee, P.; Han, J. S.	2016
A hearing self-reported survey in people over 80 years of age in China by hearing handicap inventory for the elderly-complete version vs screening version	Liu, X. Y.; Han, Y.; Yang, S. M.	2016
Subjective hearing-related quality-of-life is a major factor in the decision to continue using hearing aids among older persons	Maeda, Y.; Sugaya, A.; Nagayasu, R.; Nakagawa, A.; Nishizaki, K.	2016
Frequency-dependent loudness balancing in bimodal cochlear implant users	Veugen, L. C. E.; Chalupper, J.; Snik, A. F. M.; van Opstal, A. J.; Mens, L. H. M.	2016
Factors associated with self-reported outcome in adaptation of hearing aid	Chang, Y. S.; Choi, J.; Moon, I. J.; Hong, S. H.; Chung, W. H.; Cho, Y. S.	2016
Validation of the Hearing Implant Sound Quality Index (HISQUI) to assess Spanish-speaking cochlear implant users auditory abilities in everyday communication situations	Calvino, M.; Gavilan, J.; Sanchez-Cuadrado, I.; Perez-Mora, R. M.; Munoz, E.; Lassaletta, L.	2016

Prognostic value of psychological state in cochlear implantation	Han, J. J.; Shin, M. S.; Song, J. J.; Pai, I.; Oh, S.; Kim, B.; Park, J. H.; Lee, J. H.; Oh, S. H.	2016
Estimating the benefit of a second bone anchored hearing implant in unilaterally implanted users with a testband	Kompis, M.; Kurz, A.; Flynn, M.; Caversaccio, M.	2016
Is dehydration test using isosorbide useful in Meniere's disease?	Lee, J. D.; Kim, H. J.; Jung, J.; Kim, S. H.; Kim, B. G.; Kim, K. S.	2016
Value of pre-operative caloric test in predicting speech perception after cochlear implantation in adults with post-lingual hearing loss	Yang, C. J.; Lee, J. Y.; Ahn, J. H.; Lee, K. S.	2016
Idiopathic sensorineural hearing loss in the only hearing ear	Berrettini, S.; De Vito, A.; Bruschini, L.; Fortunato, S.; Forli, F.	2016
Cochlear implantation in post-lingually deafened adults and elderly patients: analysis of audiometric and speech perception outcomes during the first year of use	Ghiselli, S.; Nedic, S.; Montino, S.; Astolfi, L.; Bovo, R.	2016
On Detectable and Meaningful Speech-Intelligibility Benefits	Whitmer, W. M.; McShefferty, D.; Akeroyd, M. A.	2016
On the Contribution of Target Audibility to Performance in Spatialized Speech Mixtures	Best, V.; Mason, C. R.; Swaminathan, J.; Kidd, G., Jr.; Jakien, K. M.; Kampel, S. D.; Gallun, F. J.; Buchholz, J. M.; Glyde, H.	2016
Deactivating cochlear implant electrodes based on pitch information for users of the ACE strategy	Vickers, D.; Degun, A.; Canas, A.; Stainsby, T.; Vanpoucke, F.	2016
The relative contributions of temporal envelope and fine structure to Mandarin lexical tone perception in auditory neuropathy spectrum disorder	Wang, S.; Dong, R.; Liu, D.; Zhang, L.; Xu, L.	2016
Hearing and vision impairment and the 5-year incidence of falls in older adults	Gopinath, B.; McMahon, C. M.; Burlutsky, G.; Mitchell, P.	2016

Is social handicap the link between hearing loss and cognitive decline? development and validation of a new tool: The social and emotional associations of hearing loss (SEAH)	Littlejohn, J. L.; Blackburn, D. J.; Venneri, A.	2016
Phoneme and Word Scoring in Speech-in-Noise Audiometry	Billings, C. J.; Penman, T. M.; Ellis, E. M.; Baltzell, L. S.; McMillan, G. P.	2016
Can a Commercially Available Auditory Training Program Improve Audiovisual Speech Performance?	Rishiq, D.; Rao, A.; Koerner, T.; Abrams, H.	2016
Hypertension, Diuretic Use, and Risk of Hearing Loss	Lin, B. M.; Curhan, S. G.; Wang, M.; Eavey, R.; Stankovic, K. M.; Curhan, G. C.	2016
Depth of Cochlear Implant Array Within the Cochlea and Performance Outcome	Hilly, O.; Smith, L.; Hwang, E.; Shipp, D.; Symons, S.; Nedzelski, J. M.; Chen, J. M.; Lin, V. Y.	2016
Subclinical hearing loss in systemic sclerosis patients: Relation to disease severity	Gheita, T. A.; Fathi, H.; El-Akkad, M.	2016
Does increasing the intelligibility of a competing sound source interfere more with speech comprehension in older adults than it does in younger adults?	Lu, Z.; Daneman, M.; Schneider, B. A.	2016
Long-Term Outcome Data in Patients following One Year's Use of a Fully Implantable Active Middle Ear Implant	Uhler, K.; Anderson, M. C.; Jenkins, H. A.	2016
Aging, Cognitive Decline and Hearing Loss: Effects of Auditory Rehabilitation and Training with Hearing Aids and Cochlear Implants on Cognitive Function and Depression among Older Adults	Castiglione, A.; Benatti, A.; Velardita, C.; Favaro, D.; Padoan, E.; Severi, D.; Pagliaro, M.; Bovo, R.; Vallesi, A.; Gabelli, C.; Martini, A.	2016
Effects of Age and Implanted Ear on Speech Recognition in Adults with Unilateral Cochlear Implants	Sharpe, R. A.; Camposeo, E. L.; Muzaffar, W. K.; Holcomb, M. A.; Dubno, J. R.; Meyer, T. A.	2016

Transcranial Attenuation in Patients with Single-Sided Deafness	Snapp, H. A.; Morgenstein, K. E.; Telischi, F. F.; Angeli, S.	2016
Simultaneous versus Sequential Intratympanic Steroid Treatment for Severe-to-Profound Sudden Sensorineural Hearing Loss	Yoo, M. H.; Lim, W. S.; Park, J. H.; Kwon, J. K.; Lee, T. H.; An, Y. H.; Kim, Y. J.; Kim, J. Y.; Lim, H. W.; Park, H. J.	2016
Rapid Positive Influence of Cochlear Implantation on the Quality of Life in Adults 70 Years and Older	Olze, H.; Knopke, S.; Grabel, S.; Szczepek, A. J.	2016
Multicenter Clinical Trial of Vibroplasty Couplers to Treat Mixed/Conductive Hearing Loss: First Results	Zahnert, T.; Lowenheim, H.; Beutner, D.; Hagen, R.; Ernst, A.; Pau, H. W.; Zehlicke, T.; Kuhne, H.; Friese, N.; Tropitzsch, A.; Luers, J. C.; Mlynski, R.; Todt, I.; Huttenbrink, K. B.	2016
Health-Related Quality of Life in Adult Cochlear Implant Users: A Descriptive Observational Study	Ramos-Macias, A.; Falcon Gonzalez, J. C.; Borkoski-Barreiro, S. A.; Ramos de Miguel, A.; Batista, D. S.; Perez Plasencia, D.	2016
The Effect of Aging and the High-Frequency Auditory Threshold on Speech-Evoked Mismatch Negativity in a Noisy Background	Chen, J.; Chen, S.; Zheng, Y.; Ou, Y.	2016
The Development of Remote Speech Recognition Tests for Adult Cochlear Implant Users: The Effect of Presentation Mode of the Noise and a Reliable Method to Deliver Sound in Home Environments	de Graaff, F.; Huysmans, E.; Qazi, O. U.; Vanpoucke, F. J.; Merkus, P.; Goverts, S. T.; Smits, C.	2016

Five-Year Hearing Outcomes in Bilateral Simultaneously Cochlear-Implanted Adult Patients	De Seta, D.; Nguyen, Y.; Vanier, A.; Ferrary, E.; Bebear, J. P.; Godey, B.; Robier, A.; Mondain, M.; Deguine, O.; Sterkers, O.; Mosnier, I.	2016
Speech Perception and Information-Carrying Capacity for Hearing Aid Users of Different Ages	Hoppe, U.; Hocke, T.; Muller, A.; Hast, A.	2016
Treatment for Hearing Loss among the Elderly: Auditory Outcomes and Impact on Quality of Life	Manrique-Huarte, R.; Calavia, D.; Irujo, A. H.; Giron, L.; Manrique-Rodriguez, M.	2016
Transient noise reduction in cochlear implant users: A multi-band approach	Dyballa, K. H.; Hehrmann, P.; Hamacher, V.; Lenarz, T.; Buechner, A.	2016
Item response theory applied to factors affecting the patient journey towards hearing rehabilitation	Chenault, M.; Berger, M.; Kremer, B.; Anteunis, L.	2016
Discrimination of Japanese monosyllables in patients with high-frequency hearing loss	Karino, S.; Usami, S.; Kumakawa, K.; Takahashi, H.; Tono, T.; Naito, Y.; Doi, K.; Ito, K.; Suzuki, M.; Sakata, H.; Takumi, Y.; Iwasaki, S.; Kakigi, A.; Yamasoba, T.	2016
Delayed restoration of maximum speech discrimination scores in patients with idiopathic sudden sensorineural hearing loss	Noguchi, Y.; Takahashi, M.; Ito, T.; Fujikawa, T.; Kawashima, Y.; Kitamura, K.	2016

Predictive factors of speech understanding in adults with cochlear implants	Dierickx, C.; Jacquemin, L.; Boon, E.; Dierckx, A.; Debruyne, F.; Wouters, J.; Desloovere, C.; Verhaert, N.	2016
Speech-in-noise testing as a marker for noise-induced hearing loss and tinnitus	Van Eynde, C.; Denys, S.; Desloovere, C.; Wouters, J.; Verhaert, N.	2016
Clinical evaluation of an over-the-counter hearing aid (TEO First) in elderly patients suffering of mild to moderate hearing loss	Sacco, G.; Gonfrier, S.; Teboul, B.; Gahide, I.; Prate, F.; Demory-Zory, M.; Turpin, J. M.; Vuagnoux, C.; Genovese, P.; Schneider, S.; Guerin, O.; Guevara, N.	2016
Hearing loss and use of health services: a population-based cross-sectional study among Finnish older adults	Mikkola, T. M.; Polku, H.; Sainio, P.; Koponen, P.; Koskinen, S.; Viljanen, A.	2016
Vision and hearing impairments, cognitive impairment and mortality among long-term care recipients: a population-based cohort study	Mitoku, K.; Masaki, N.; Ogata, Y.; Okamoto, K.	2016
Personalised long-term follow-up of cochlear implant patients using remote care, compared with those on the standard care pathway: study protocol for a feasibility randomised controlled trial	Cullington, H.; Kitterick, P.; DeBold, L.; Weal, M.; Clarke, N.; Newberry, E.; Aubert, L.	2016
Speech recognition in individuals with sensorineural hearing loss	de Andrade, A. N.; Iorio, M. C. M.; Gil, D.	2016
Cancer treatment in determination of hearing loss	de Oliveira, P. F.; Oliveira, C. S.; Andrade, J. S.; do Carmo Santos, T. F.; de Oliveira-Barreto, A. C.	2016

The development and standardization of self-assessment for hearing screening of the elderly	Na, W.; Kim, G.; Han, W.; Kim, J.	2016
Auditory cortical activity to different voice onset times in cochlear implant users	Han, J. H.; Zhang, F.; Kadis, D. S.; Houston, L. M.; Samy, R. N.; Smith, M. L.; Dimitrijevic, A.	2016
Prospective case-controlled sound localisation study after cochlear implantation in adults with single-sided deafness and ipsilateral tinnitus	Mertens, G.; Desmet, J.; De Bodt, M.; Van de Heyning, P.	2016
Factors that influence outcomes in cochlear implantation in adults, based on patient-related characteristics - a retrospective study	Kraaijenga, V. J. C.; Smit, A. L.; Stegeman, I.; Smilde, J. J. M.; van Zanten, G. A.; Grolman, W.	2016
Cochlear implants for pre-lingually profoundly deaf adults	Craddock, L.; Cooper, H.; Riley, A.; Wright, T.	2016
Cochlear implantation in early deafened, late implanted adults: Do they benefit?	Kumar, R. S.; Mawman, D.; Sankaran, D.; Melling, C.; O'Driscoll, M.; Freeman, S. M.; Lloyd, S. K.	2016
Perspectives of adults with cochlear implants on current CI services and daily life	Ng, Z. Y.; Lamb, B.; Harrigan, S.; Archbold, S.; Athalye, S.; Allen, S.	2016
Comparison of two cochlear implant coding strategies on speech perception	Dillon, M. T.; Buss, E.; King, E. R.; Deres, E. J.; Obarowski, S. N.; Anderson, M. L.; Adunka, M. C.	2016
Mono-syllabic word test score as a pre-operative assessment criterion for cochlear implant candidature in adults with acquired hearing loss	Doran, M.; Jenkinson, L.	2016
Predicting speech perception outcomes following cochlear implantation in adults with unilateral deafness or highly asymmetric hearing loss	Kitterick, P. T.; Lucas, L.	2016

Outcomes of cochlear implantation in patients with neurofibromatosis type 2	North, H. J.; Mawman, D.; O'Driscoll, M.; Freeman, S. R.; Rutherford, S. A.; King, A. T.; Hammerbeck-Ward, C.; Evans, D. G.; Lloyd, S. K.	2016
Cochlear implantation in recipients with single-sided deafness: Audiological performance	Hoth, S.; Rosli-Khabas, M.; Herisanu, I.; Plinkert, P. K.; Praetorius, M.	2016
Quality of life in adult cochlear implant users	Angelo, T. C.; Moret, A. L.; Costa, O. A.; Nascimento, L. T.; Alvarenga Kd.e, F.	2016
Effects of hearing and cognitive impairment in sentence recognition	Bruckmann, M.; Pinheiro, M. M.	2016
Smartphone-based noise adaptive speech enhancement for hearing aid applications	Panahi, I.; Kehtarnavaz, N.; Thibodeau, L.	2016
Evaluation of audiometric thresholds and speech perception sentence test in adults and elderly after cochlear implantation	Carvalho, G. M.; Santos, M. D. C. F.; Pauna, H. F.; Guimaraes, A. C.; Curi, S. B.; Jeronymo, D.; Porto, P. R. C.; Bianchini, W. A.; Castilho, A. M.; Sartorato, E. L.	2016
Auditory profiling and hearing-aid satisfaction in hearing-aid candidates	Thorup, N.; Santurette, S.; Jorgensen, S.; Kjaerbol, E.; Dau, T.; Friis, M.	2016
The Effect of Functional Hearing and Hearing Aid Usage on Verbal Reasoning in a Large Community-Dwelling Population	Keidser, G.; Rudner, M.; Seeto, M.; Hygge, S.; Ronnberg, J.	2016

A Randomized Control Trial: Supplementing Hearing Aid Use with Listening and Communication Enhancement (LACE) Auditory Training	Saunders, G. H.; Smith, S. L.; Chisolm, T. H.; Frederick, M. T.; McArdle, R. A.; Wilson, R. H.	2016
Tinnitus and Sleep Difficulties After Cochlear Implantation	Pierzycki, R. H.; Edmondson-Jones, M.; Dawes, P.; Munro, K. J.; Moore, D. R.; Kitterick, P. T.	2016
Impact of Hearing Aid Technology on Outcomes in Daily Life II: Speech Understanding and Listening Effort	Johnson, J. A.; Xu, J.; Cox, R. M.	2016
Revealing Hearing Loss: A Survey of How People Verbally Disclose Their Hearing Loss	West, J. S.; Low, J. C.; Stankovic, K. M.	2016
Text as a Supplement to Speech in Young and Older Adults	Krull, V.; Humes, L. E.	2016
Word Recognition Variability With Cochlear Implants: "Perceptual Attention" Versus "Auditory Sensitivity"	Moberly, A. C.; Lowenstein, J. H.; Nittrouer, S.	2016
Development of the Word Auditory Recognition and Recall Measure: A Working Memory Test for Use in Rehabilitative Audiology	Smith, S. L.; Pichora-Fuller, M. K.; Alexander, G.	2016
Psychometric Functions of Dual-Task Paradigms for Measuring Listening Effort	Wu, Y. H.; Stangl, E.; Zhang, X.; Perkins, J.; Eilers, E.	2016
Processing Mechanisms in Hearing-Impaired Listeners: Evidence from Reaction Times and Sentence Interpretation	Carroll, R.; Uslar, V.; Brand, T.; Ruigendijk, E.	2016
Assessment of Spectral and Temporal Resolution in Cochlear Implant Users Using Psychoacoustic Discrimination and Speech Cue Categorization	Winn, M. B.; Won, J. H.; Moon, I. J.	2016
Having Two Ears Facilitates the Perceptual Separation of Concurrent Talkers for Bilateral and Single-Sided Deaf Cochlear Implantees	Bernstein, J. G.; Goupell, M. J.; Schuchman, G. I.; Rivera, A. L.; Brungart, D. S.	2016
Nonlinguistic Outcome Measures in Adult Cochlear Implant Users Over the First Year of Implantation	Drennan, W. R.; Won, J. H.; Timme, A. O.; Rubinstein, J. T.	2016

Effects of Modified Hearing Aid Fittings on Loudness and Tone Quality for Different Acoustic Scenes	Moore, B. C.; Baer, T.; Ives, D. T.; Marriage, J.; Salorio-Corbetto, M.	2016
Factors Affecting Speech Perception Improvement Post Implantation in Congenitally Deaf Adults	O'Gara, S. J.; Cullington, H. E.; Grasmeder, M. L.; Adamou, M.; Matthews, E. S.	2016
Cortical Activation Patterns Correlate with Speech Understanding After Cochlear Implantation	Olds, C.; Pollonini, L.; Abaya, H.; Larky, J.; Loy, M.; Bortfeld, H.; Beauchamp, M. S.; Oghalai, J. S.	2016
Factors Predicting Postoperative Unilateral and Bilateral Speech Recognition in Adult Cochlear Implant Recipients with Acoustic Hearing	Plant, K.; McDermott, H.; van Hoesel, R.; Dawson, P.; Cowan, R.	2016
Effects of Reverberation and Compression on Consonant Identification in Individuals with Hearing Impairment	Reinhart, P. N.; Souza, P. E.; Srinivasan, N. K.; Gallun, F. J.	2016
Validation of a French-Language Version of the Spatial Hearing Questionnaire, Cluster Analysis and Comparison with the Speech, Spatial, and Qualities of Hearing Scale	Moulin, A.; Richard, C.	2016
Matching Automatic Gain Control Across Devices in Bimodal Cochlear Implant Users	Veugen, L. C.; Chalupper, J.; Snik, A. F.; Opstal, A. J.; Mens, L. H.	2016
Cortisol, Chromogranin A, and Pupillary Responses Evoked by Speech Recognition Tasks in Normally Hearing and Hard-of-Hearing Listeners: A Pilot Study	Kramer, S. E.; Teunissen, C. E.; Zekveld, A. A.	2016
Change in Psychosocial Health Status Over 5 Years in Relation to Adults' Hearing Ability in Noise	Stam, M.; Smit, J. H.; Twisk, J. W.; Lemke, U.; Smits, C.; Festen, J. M.; Kramer, S. E.	2016
Predicting Speech-in-Noise Recognition From Performance on the Trail Making Test: Results From a Large-Scale Internet Study	Ellis, R. J.; Molander, P.; Ronnberg, J.; Lyxell, B.; Andersson, G.; Lunner, T.	2016

Using Speech Recall in Hearing Aid Fitting and Outcome Evaluation Under Ecological Test Conditions	Lunner, T.; Rudner, M.; Rosenbom, T.; Agren, J.; Ng, E. H.	2016
The Influence of Linguistic Proficiency on Masked Text Recognition Performance in Adults With and Without Congenital Hearing Impairment	Huysmans, E.; Bolk, E.; Zekveld, A. A.; Festen, J. M.; de Groot, A. M.; Goverts, S. T.	2016
Using the Digits-In-Noise Test to Estimate Age-Related Hearing Loss	Koole, A.; Nagtegaal, A. P.; Homans, N. C.; Hofman, A.; Baatenburg de Jong, R. J.; Goedegebure, A.	2016
Hearing and Vestibular Function After Preoperative Intratympanic Gentamicin Therapy for Vestibular Schwanomma as Part of Vestibular Prehab	Tjernstrom, F.; Fransson, P. A.; Kahlon, B.; Karlberg, M.; Lindberg, S.; Siesjo, P.; Magnusson, M.	2016
Effects of Threshold Adjustment on Speech Perception in Nucleus Cochlear Implant Recipients	Busby, P. A.; Arora, K.	2016
Impact of Hearing Aid Technology on Outcomes in Daily Life I: The Patients' Perspective	Cox, R. M.; Johnson, J. A.; Xu, J. J.	2016
Intelligibility of the Patient's Speech Predicts the Likelihood of Cochlear Implant Success in Prelingually Deaf Adults	van Dijkhuizen, J. N.; Boermans, Ppbm; Briaire, J. J.; Frijns, J. H. M.	2016
Listening Effort in Younger and Older Adults: A Comparison of Auditory-Only and Auditory-Visual Presentations	Sommers, M. S.; Phelps, D.	2016
Technical aids for speech understanding in cochlear implanted adults using cell-phones	Rey, P.; Cochard, N.; Rizzoli, M.; Laborde, M. L.; Tartayre, M.; Mondain, M.; Deguine, O.	2016
Benefit of contralateral hearing aid in adult cochlear implant bearers	Bouccara, D.; Blanchet, E.; Waterlot, P. E.; Smadja, M.; Frachet, B.; Meyer, B.; Sterkers, O.	2016
Perception in noise with the Digisonic SP cochlear implant: Clinical trial of Saphyr processor's upgraded signal processing	Bergeron, F.; Hotton, M.	2016

Validation of the French-language version of the OTOSPEECH automated scoring software package for speech audiometry	Venail, F.; Legris, E.; Vaerenberg, B.; Puel, J. L.; Govaerts, P. J.; Ceccato, J. C.	2016
Smartphone-based audiometric test for screening hearing loss in the elderly	Abu-Ghanem, S.; Handzel, O.; Ness, L.; Ben-Artzi-Blima, M.; Fait-Ghelbendorf, K.; Himmelfarb, M.	2016
Satisfaction and compliance of adult patients using hearing aid and evaluation of factors affecting them	Korkmaz, M. H.; Bayir, O.; Er, S.; Isik, E.; Saylam, G.; Tatar, E. C.; Ozdek, A.	2016
Is age a limiting factor for adaptation to cochlear implant?	Hiel, A. L.; Gerard, J. M.; Decat, M.; Deggouj, N.	2016
Cross-cultural adaptation and validation of the Nijmegen Cochlear Implant Questionnaire into Italian	Ottaviani, F.; Iacona, E.; Sykopetrites, V.; Schindler, A.; Mozzanica, F.	2016
Clinical evaluation of the xDP output compression strategy for cochlear implants	Bozorg-Grayeli, A.; Guevara, N.; Bebear, J. P.; Ardoint, M.; Saai, S.; Hoen, M.; Gnansia, D.; Romanet, P.; Lavieille, J. P.	2016
The influence of frequency-dependent hearing loss to unaided APHAB scores	Lohler, J.; Akcicek, B.; Wollenberg, B.; Kappe, T.; Schlattmann, P.; Schonweiler, R.	2016
The Carina¬© middle ear implant: surgical and functional outcomes	Bruschini, L.; Berrettini, S.; Forli, F.; Murri, A.; Cuda, D.	2016
Health-related quality of life and mental distress in patients with partial deafness: preliminary findings	Ciesla, K.; Lewandowska, M.; Skarzynski, H.	2016

Surgical, functional and audiological evaluation of new Baha Attract system implantations	Gawecki, W.; Stieler, O. M.; Balcerowiak, A.; Komar, D.; Gibasiewicz, R.; Karlik, M.; Szyfter-Harris, J.; Wrobel, M.	2016
Using the HISQUI29 to assess the sound quality levels of Spanish adults with unilateral cochlear implants and no contralateral hearing	Calvino, M.; Gavilan, J.; Sanchez-Cuadrado, I.; Perez-Mora, R. M.; Munoz, E.; Diez-Sebastian, J.; Lassaletta, L.	2016
The efficacy and safety of systemic injection of Ginkgo biloba extract, EGb761, in idiopathic sudden sensorineural hearing loss: a randomized placebo-controlled clinical trial	Koo, J. W.; Chang, M. Y.; Yun, S. C.; Kim, T. S.; Kong, S. K.; Chung, J. W.; Goh, E. K.	2016
Speech understanding in noise with the Roger Pen, Naida CI Q70 processor, and integrated Roger 17 receiver in a multi-talker network	De Ceulaer, G.; Bestel, J.; Mulder, H. E.; Goldbeck, F.; de Varebeke, S. P.; Govaerts, P. J.	2016
Ipsilateral cochlear implantation in patients with sporadic vestibular schwannoma in the only or best hearing ear and in patients with NF2	Lassaletta, L.; Aristegui, M.; Medina, M.; Aranguez, G.; Perez-Mora, R. M.; Falcioni, M.; Gavilan, J.; Piazza, P.; Sanna, M.	2016
Subtotal petrosectomy and CodacsTM: new possibilities in ears with chronic infection	Schwab, B.; Kludt, E.; Maier, H.; Lenarz, T.; Teschner, M.	2016
Bilateral use of active middle ear implants: speech discrimination results in noise	Wolf-Magele, A.; Koci, V.; Schnabl, J.; Zorowka, P.; Riechelmann, H.; Sprinzl, G. M.	2016
Working memory training and speech in noise comprehension in older adults	Wayne, R. V.; Hamilton, C.; Huyck, J. J.; Johnsrude, I. S.	2016

Speech comprehension difficulties in chronic tinnitus and its relation to hyperacusis	Vielsmeier, V.; Kreuzer, P. M.; Haubner, F.; Steffens, T.; O. Semmler P.R; Kleinjung, T.; Schlee, W.; Langguth, B.; Schecklmann, M.	2016
Speech discrimination difficulties in high-functioning autism spectrum disorder are likely independent of auditory hypersensitivity	Dunlop, W. A.; Enticott, P. G.; Rajan, R.	2016
The Repeatable Battery for the Assessment of Neuropsychological Status for Hearing Impaired Individuals (RBANS-H) before and after Cochlear Implantation: A Protocol for a Prospective, Longitudinal Cohort Study	Claes, A. J.; Mertens, G.; Gilles, A.; Hofkens-van den Brandt, A.; Fransen, E.; Van Rompaey, V.; Van de Heyning, P.	2016
Decreased speech-in-noise understanding in young adults with tinnitus	Gilles, A.; Schlee, W.; Rabau, S.; Wouters, K.; Fransen, E.; Van de Heyning, P.	2016
Only Behavioral But Not Self-Report Measures of Speech Perception Correlate with Cognitive Abilities	Heinrich, A.; Henshaw, H.; Ferguson, M. A.	2016
Cognitive Load on Speech Recognition with Competing Talkers	Meister, H.; Schreitmuller, S.; Ortmann, M.; Rahlmann, S.; Walger, M.	2016
The Auditory-Visual Speech Benefit on Working Memory in Older Adults with Hearing Impairment	Frtusova, J. B.; Phillips, N. A.	2016
Multiple Solutions to the Same Problem: Utilization of Plausibility and Syntax in Sentence Comprehension by Older Adults with Impaired Hearing	Amichetti, N. M.; Whiteand, A. G.; Wingfield, A.	2016
The Two Sides of Sensory-Cognitive Interactions: Effects of Age, Hearing Acuity, and Working Memory Span on Sentence Comprehension	DeCaro, R.; Peelle, J. E.; Grossman, M.; Wingfield, A.	2016
Auditory Perceptual Learning in Adults with and without Age-Related Hearing Loss	Karawani, H.; Bitan, T.; Attias, J.; Banai, K.	2016

Information theoretic evaluation of a noiseband-based cochlear implant simulator	Aguiar, D. E.; Taylor, N. E.; Li, J.; Gazanfari, D. K.; Talavage, T. M.; Laflen, J. B.; Neuberger, H.; Svirsky, M. A.	2016
Indication of direct acoustical cochlea stimulation in comparison to cochlear implants	Kludt, E.; Buchner, A.; Schwab, B.; Lenarz, T.; Maier, H.	2016
Primary auditory symptoms in patients with normal peripheral hearing sensitivity: Redefining hearing loss	Shinn, J.; Long, A.; Rayle, C.; Bush, M.	2016
Vestibular schwannoma diagnosis: evaluation of a neuro-otological test battery	Di Giustino, F.; Pecci, R.; Vannucchi, P.	2016
Prevalence, awareness, and preventive practices of noise-induced hearing loss in a plywood industry	Edward, M.; Manohar, S.; Somayaji, G.; Kallikkadan, H. H.	2016
Auditory Speech Perception Tests in Relation to the Coding Strategy in Cochlear Implant	Bazon, A. C.; Mantello, E. B.; Goncales, A. S.; Isaac, M. D. L.; Hyppolito, M. A.; Reis, A. C. M. B.	2016
Relationship between speech perception and level of satisfaction of hearing aid users	Mantello, E. B.; Da Silva, C. D.; Massuda, E. T.; Hyppolito, M. A.; Dos Reis, A. C. M. B.	2016
The efficiency of auditory training in elderly hearing aids users	Bertagnolli, A. P.; Buss, L. H.	2016
Hearing aid fitting when the indication is controversial	Ferreira, G. C.; Dos Santos, S. N.; Costa, M. J.; Stumpp, L. Z. L.; Costa, L. D.; Muller, M. D.	2016
Vibrant soundbridge and bone conduction hearing aid in patients with bilateral malformation of external ear	Garcia Mondelli, M. F. C.; Mariano, T. C. B.; Honorio, H. M.; De Brito, R. V.	2016

Development of Telscreen: a telephone-based speech-in-noise hearing screening test with a novel masking noise and scoring procedure	Dillon, H.; Beach, E. F.; Seymour, J.; Carter, L.; Golding, M.	2016
Fitting recommendations and clinical benefit associated with use of the NAL-NL2 hearing-aid prescription in Nucleus cochlear implant recipients	English, R.; Plant, K.; Maciejczyk, M.; Cowan, R.	2016
Utility of bilateral acoustic hearing in combination with electrical stimulation provided by the cochlear implant	Plant, K.; Babic, L.	2016
Influence of contralateral acoustic hearing on adult bimodal outcomes after cochlear implantation	Plant, K.; van Hoesel, R.; McDermott, H.; Dawson, P.; Cowan, R.	2016
A comparison of an audiometric screening survey with an in-depth research questionnaire for hearing loss and hearing loss risk factors	Mosites, E.; Neitzel, R.; Galusha, D.; Trufan, S.; Dixon-Ernst, C.; Rabinowitz, P.	2016
Motivational engagement in first-time hearing aid users: A feasibility study	Ferguson, M.; Maidment, D.; Russell, N.; Gregory, M.; Nicholson, R.	2016
Understanding the psychosocial experiences of adults with mild-moderate hearing loss: An application of Leventhal's self-regulatory model	Heffernan, E.; Coulson, N. S.; Henshaw, H.; Barry, J. G.; Ferguson, M. A.	2016
Factors influencing pursuit of hearing evaluation: Enhancing the health belief model with perceived burden from hearing loss on communication partners	Schulz, K. A.; Modeste, N.; Lee, J.; Roberts, R.; Saunders, G. H.; Witsell, D. L.	2016
The effects of frequency lowering on speech perception in noise with adult hearing-aid users	Miller, C. W.; Bates, E.; Brennan, M.	2016
Development and preliminary evaluation of a new test of ongoing speech comprehension	Best, V.; Keidser, G.; Buchholz, J. M.; Freeston, K.	2016
Evidence-based guidelines for recommending cochlear implantation for postlingually deafened adults	Leigh, J. R.; Moran, M.; Hollow, R.; Dowell, R. C.	2016
Adult hearing-aid users with cochlear dead regions restricted to high frequencies: Implications for amplification	Pepler, A.; Lewis, K.; Munro, K. J.	2016

Receptive language as a predictor of cochlear implant outcome for prelingually deaf adults	Rousset, A.; Dowell, R.; Leigh, J.	2016
Evaluation of a wireless audio streaming accessory to improve mobile telephone performance of cochlear implant users	Wolfe, J.; Morais Duke, M.; Schafer, E.; Cire, G.; Menapace, C.; O'Neill, L.	2016
Hearing impairment, cognition and speech understanding: exploratory factor analyses of a comprehensive test battery for a group of hearing aid users, the n200 study	Ronnberg, J.; Lunner, T.; Ng, E. H.; Lidestam, B.; Zekveld, A. A.; Sorqvist, P.; Lyxell, B.; Traff, U.; Yumba, W.; Classon, E.; Hallgren, M.; Larsby, B.; Signoret, C.; Pichora-Fuller, M. K.; Rudner, M.; Danielsson, H.; Stenfelt, S.	2016
The Voice Track multiband single-channel modified Wiener-filter noise reduction system for cochlear implants: patients' outcomes and subjective appraisal	Guevara, N.; Bozorg-Grayeli, A.; Bebear, J. P.; Ardoint, M.; Saai, S.; Gnansia, D.; Hoen, M.; Romanet, P.; Lavieille, J. P.	2016
Sources of variability of speech, spatial, and qualities of hearing scale (SSQ) scores in normal-hearing and hearing-impaired populations	Moulin, A.; Richard, C.	2016
Perceived listening effort and speech intelligibility in reverberation and noise for hearing-impaired listeners	Schepker, H.; Haeder, K.; Rennies, J.; Holube, I.	2016
Validating self-reporting of hearing-related symptoms against pure-tone audiometry, otoacoustic emission, and speech audiometry	Fredriksson, S.; Hammar, O.; Magnusson, L.; Kahari, K.; Waye, K. P.	2016
Perception of hearing impairment and the willingness to use hearing aids in an elderly population in southern Taiwan: A community-based study	Chang, N. C.; Dai, C. Y.; Lin, W. Y.; Chien, C. Y.; Hsieh, M. H.; Ho, K. Y.	2016

Amplification of transcutaneous and percutaneous bone-conduction devices with a test-band in an induced model of conductive hearing loss	Park, M. J.; Lee, J. R.; Yang, C. J.; Yoo, M. H.; Jin, I. S.; Choi, C. H.; Park, H. J.	2016
Adjustments of the amplitude mapping function: Sensitivity of cochlear implant users and effects on subjective preference and speech recognition	Theelen-van den Hoek, F. L.; Boymans, M.; van Dijk, B.; Dreschler, W. A.	2016
The effect of fluctuating maskers on speech understanding of high-performing cochlear implant users	Zirn, S.; Polterauer, D.; Keller, S.; Hemmert, W.	2016
The effect of intratympanic gentamicin for treatment of Meniere's disease on lower frequency hearing	Sam, G.; Chung, D. W.; van der Hoeven, R.; Verweij, S.; Becker, M.	2016
Comparison of Bilateral and Unilateral Cochlear Implantation in Adults: A Randomized Clinical Trial	Smulders, Y. E.; van Zon, A.; Stegeman, I.; Rinia, A. B.; Van Zanten, G. A.; Stokroos, R. J.; Hendrice, N.; Free, R. H.; Maat, B.; Frijns, J. H.; Briaire, J. J.; Mylanus, E. A.; Huinck, W. J.; Smit, A. L.; Topsakal, V.; Tange, R. A.; Grolman, W.	2016
The Role of Age-Related Declines in Subcortical Auditory Processing in Speech Perception in Noise	Schoof, T.; Rosen, S.	2016
The Intelligibility of Interrupted Speech: Cochlear Implant Users and Normal Hearing Listeners	Bhargava, P.; Gaudrain, E.; Baskent, D.	2016
Assessing the Electrode-Neuron Interface with the Electrically Evoked Compound Action Potential, Electrode Position, and Behavioral Thresholds	DeVries, L.; Scheperle, R.; Bierer, J. A.	2016
Two Ears Are Not Always Better than One: Mandatory Vowel Fusion Across Spectrally Mismatched Ears in Hearing-Impaired Listeners	Reiss, L. A.; Eggleston, J. L.; Walker, E. P.; Oh, Y.	2016

A Comparison of Self-Report and Audiometric Measures of Hearing and Their Associations With Functional Outcomes in Older Adults	Choi, J. S.; Betz, J.; Deal, J.; Contrera, K. J.; Genther, D. J.; Chen, D. S.; Gispen, F. E.; Lin, F. R.	2016
Association of Hearing Loss and Loneliness in Older Adults	Sung, Y. K.; Li, L.; Blake, C.; Betz, J.; Lin, F. R.	2016
Speech perception and quality of life of open-fit hearing aid users	Garcia, T. M.; Jacob, R. T.; Mondelli, M. F.	2016
How does difficulty communicating affect the social relationships of older adults? An exploration using data from a national survey	Palmer, A. D.; Newsom, J. T.; Rook, K. S.	2016
Speech audiometry findings from HIV plus and HIV- adults in the MACS and WIHS longitudinal cohort studies	Torre, P.; Hoffman, H. J.; Springer, G.; Cox, C.; Young, M. A.; Margolick, J. B.; Plankey, M.	2016
Cochlear Implant Evaluation: Prognosis Estimation by Data Mining System	Guerra-Jimenez, G.; De Miguel, A. R.; Gonzalez, J. C. F.; Barreiro, S. A. B.; Plasencia, D. P.; Macias, A. R.	2016
Cochlear implantation in elderly patients: stability of outcome over time	Hilly, O.; Hwang, E.; Smith, L.; Shipp, D.; Nedzelski, J. M.; Chen, J. M.; Lin, V. W. Y.	2016
Initial clinical experience with the nucleus CI532 cochlear implant electrode	Briggs, R.; Gavrilis, J.; Morgan, C.; Risi, F.; Aschendorff, A.; James, C.; Cowan, R.	2016
Efficacy of a parametric assistive listening system to enhance the audibility and intelligibility of speech	Ho, E. C.; Reddy, M. V.; Peksi, S.; Gan, W. S.	2016
Baha Attract System: 6-month results of a multicentre, open, prospective clinical investigation	Hol, M.; Monksfield, P.; Skarzynski, P.; Green, K.; Runge, C.; Wigren, S.; Blechert, J. I.; Flynn, M.; Bosman, A.; Mylanus, E.	2016

Codacs as new treatment option for patients with severe and profound mixed hearing loss including cases with chronic otitis and cholesteatoma	Lenarz, T.	2016
Couplers for vibrant Soundbridge implant vs no-coupler-vibrant Soundbridge implant	Manrique, M.	2016
VORP 503 in mixed hearing loss and radical cavities	Mewes, T.	2016
Can the softband BAHA indicate the prospective improvement in hearing with middle ear implants?	Roplekar, R.; Patrick, S.; Jones, S.	2016
Effect of speaking rate on recognition of natural fast speech by cochlear implant users	Sakamoto, K.; Obuchi, C.; Matsuda, H.; Araki, R.; Shiroma, M.; Ikezono, T.	2016
Translation and cross-cultural adaptation of hearing handicap inventory for the elderly-screening (HHIE-S) in the bilingual singapore population-a pilot study	Ho, E. C.; Lim, D.; Yeak, S.	2016
Hearing impairment among nasopharyngeal cancer (NPC) survivors	Ho, E. C.; Yu, Y.; Yeak, S.	2016
Middle ear implants-technique and outcome	Doi, K.	2016
Management of mastoid cavities with Vibrant Soundbridge	Gavilan, J.; Lassaletta, L.	2016
Hearing subclassification may predict long-term auditory outcomes after radiosurgery for vestibular schwannoma patients with good hearing	Mousavi, S. H.; Niranjan, A.; Akpinar, B.; Huang, M.; Kano, H.; Tonetti, D.; Flickinger, J. C.; Dade Lunsford, L.	2016
Stability of hearing preservation and regeneration capacity of the cochlear nerve following vestibular schwannoma surgery via a retrosigmoid approach	Scheller, C.; Wienke, A.; Tatagiba, M.; Gharabaghi, A.; Ramina, K. F.; Ganslandt, O.; Bischoff, B.; Matthies, C.; Westermaier, T.; Antoniadis, G.; Pedro, M. T.; Rohde, V.; von Eckardstein, K.; Kretschmer, T.; Zenk, J.; Strauss, C.	2016

Real-time intraoperative monitoring of brainstem auditory evoked potentials during microvascular decompression for hemifacial spasm	Joo, B. E.; Park, S. K.; Cho, K. R.; Kong, D. S.; Seo, D. W.; Park, K.	2016
Visual impairment screening at the Geriatric Frailty Clinic for Assessment of Frailty and Prevention of Disability at the Gerontopole	Soler, V.; Sourdet, S.; Balardy, L.; Van Kan, G. A.; Brechemier, D.; Bugat, M. E. R.; Tavassoli, N.; Cassagne, M.; Malecaze, F.; Nourhashemi, F.; Vellas, B.	2016
Experiments on Auditory-Visual Perception of Sentences by Users of Unilateral, Bimodal, and Bilateral Cochlear Implants	Dorman, M. F.; Liss, J.; Wang, S.; Berisha, V.; Ludwig, C.; Natale, S. C.	2016
Examination of Individual Differences in Outcomes From a Randomized Controlled Clinical Trial Comparing Formal and Informal Individual Auditory Training Programs	Smith, S. L.; Saunders, G. H.; Chisolm, T. H.; Frederick, M.; Bailey, B. A.	2016
Auditory Training With Frequent Communication Partners	Tye-Murray, N.; Spehar, B.; Sommers, M.; Barcroft, J.	2016
Hearing Loss and Cognitive-Communication Test Performance of Long-Term Care Residents With Dementia: Effects of Amplification	Hopper, T.; Slaughter, S. E.; Hodgetts, B.; Ostevik, A.; Ickert, C.	2016
Intelligibility and Clarity of Reverberant Speech: Effects of Wide Dynamic Range Compression Release Time and Working Memory	Reinhart, P. N.; Souza, P. E.	2016
Task- and Talker-Specific Gains in Auditory Training	Barcroft, J.; Spehar, B.; Tye-Murray, N.; Sommers, M.	2016
Glimpsing Speech in the Presence of Nonsimultaneous Amplitude Modulations From a Competing Talker: Effect of Modulation Rate, Age, and Hearing Loss	Fogerty, D.; Ahlstrom, J. B.; Bologna, W. J.; Dubno, J. R.	2016

Effects of Removing Low-Frequency Electric Information on Speech Perception With Bimodal Hearing	Fowler, J. R.; Eggleston, J. L.; Reavis, K. M.; McMillan, G. P.; Reiss, L. A.	2016
Using ILD or ITD Cues for Sound Source Localization and Speech Understanding in a Complex Listening Environment by Listeners With Bilateral and With Hearing-Preservation Cochlear Implants	Loiselle, L. H.; Dorman, M. F.; Yost, W. A.; Cook, S. J.; Gifford, R. H.	2016
Seeing the Talker's Face Improves Free Recall of Speech for Young Adults With Normal Hearing but Not Older Adults With Hearing Loss	Rudner, M.; Mishra, S.; Stenfelt, S.; Lunner, T.; Ronnberg, J.	2016
The Use of Voice Cues for Speaker Gender Recognition in Cochlear Implant Recipients	Meister, H.; Fursen, K.; Streicher, B.; Lang-Roth, R.; Walger, M.	2016
The Relationship Between Speech Production and Speech Perception Deficits in Parkinson's Disease	De Keyser, K.; Santens, P.; Bockstael, A.; Botteldooren, D.; Talsma, D.; De Vos, S.; Van Cauwenberghe, M.; Verheugen, F.; Corthals, P.; De Letter, M.	2016
Perception of contrastive bi-syllabic lexical stress in unaccented and accented words by younger and older listeners	Gordon-Salant, S.; Yeni-Komshian, G. H.; Pickett, E. J.; Fitzgibbons, P. J.	2016
Nonlinear frequency compression: Influence of start frequency and input bandwidth on consonant and vowel recognition	Alexander, J. M.	2016
Large-scale training to increase speech intelligibility for hearing-impaired listeners in novel noises	Chen, J.; Wang, Y.; Yoho, S. E.; Wang, D.; Healy, E. W.	2016
Head orientation benefit to speech intelligibility in noise for cochlear implant users and in realistic listening conditions	Grange, J. A.; Culling, J. F.	2016
The benefit of head orientation to speech intelligibility in noise	Grange, J. A.; Culling, J. F.	2016
Better-ear glimpsing at low frequencies in normal-hearing and hearing-impaired listeners	Rana, B.; Buchholz, J. M.	2016
Speech rate effects on the processing of conversational speech across the adult life span	Koch, X.; Janse, E.	2016

Spectral contrast enhancement improves speech intelligibility in noise for cochlear implants	Nogueira, W.; Rode, T.; Buchner, A.	2016
The Effects of Hearing Aid Directional Microphone and Noise Reduction Processing on Listening Effort in Older Adults with Hearing Loss	Desjardins, J. L.	2016
Clinical Outcomes of the CochlearTM Nucleus() 5 Cochlear Implant System and SmartSoundTM 2 Signal Processing	Runge, C. L.; Henion, K.; Tarima, S.; Beiter, A.; Zwolan, T. A.	2016
The Effect of Lexical Content on Dichotic Speech Recognition in Older Adults	Findlen, U. M.; Roup, C. M.	2016
Evaluation of Loudspeaker-Based Virtual Sound Environments for Testing Directional Hearing Aids	Oreinos, C.; Buchholz, J. M.	2016
Behavioral Measures of Temporal Processing and Speech Perception in Cochlear Implant Users	Blankenship, C.; Zhang, F. W.; Keith, R.	2016
Recognition of Speech from the Television with Use of a Wireless Technology Designed for Cochlear Implants	Duke, M. M.; Wolfe, J.; Schafer, E.	2016
A Phoneme Perception Test Method for High-Frequency Hearing Aid Fitting	Schmitt, N.; Winkler, A.; Boretzki, M.; Holube, I.	2016
Directional Processing and Noise Reduction in Hearing Aids: Individual and Situational Influences on Preferred Setting	Neher, T.; Wagener, K. C.; Fischer, R. L.	2016
Validity and Reliability of the Hearing Handicap Inventory for Elderly: Version Adapted for Use on the Portuguese Population	de Paiva, S. M. M.; Simoes, J.; Paiva, A.; Newman, C.; Sousa, F. C. E.; Bebear, J. P.	2016
Gender Differences in Audiological Findings and Hearing Aid Benefit in 255 Individuals with Auditory Neuropathy Spectrum Disorder: A Retrospective Study	Narne, V. K.; Prabhu, P.; Chandan, H. S.; Deepthi, M.	2016
Multicenter clinical trial of the Nucleus Hybrid S8 cochlear implant: Final outcomes	Gantz, B. J.; Dunn, C.; Oleson, J.; Hansen, M.; Parkinson, A.; Turner, C.	2016

United States multicenter clinical trial of the cochlear nucleus hybrid implant system	Roland, J. T., Jr.; Gantz, B. J.; Waltzman, S. B.; Parkinson, A. J.; Multicenter Clinical Trial, Group	2016
Monaural or binaural sound deprivation in postlingual hearing loss: Cochlear implant in the worse ear	Canale, A.; Dalmasso, G.; Dagna, F.; Lacilla, M.; Montuschi, C.; Di Rosa, R.; Albera, R.	2016
Pulse steroid therapy in idiopathic sudden sensorineural hearing loss: A randomized controlled clinical trial	Eftekharian, A.; Amizadeh, M.	2016
Asymmetric hearing loss is common and benign in patients aged 95 years and older	Leskowitz, M. J.; Caruana, F. F.; Siedlecki, B.; Qian, Z. J.; Spitzer, J. B.; Lalwani, A. K.	2016
Validation of a Self-Administered Audiometry Application: An Equivalence Study	Whitton, J. P.; Hancock, K. E.; Shannon, J. M.; Polley, D. B.	2016
Effect of low frequency on speech performance with bimodal hearing in bilateral severe hearing loss	Choi, S. J.; Lee, J. B.; Bahng, J.; Lee, W. K.; Park, C. H.; Kim, H. J.; Lee, J. H.	2016
Impact of cochlear implantation on quality of life and mental comorbidity in patients aged 80 years	Knopke, S.; Grabel, S.; Forster-Ruhrmann, U.; Mazurek, B.; Szczepek, A. J.; Olze, H.	2016
Audiologic limitations of Vibrant Soundbridge device: Is the contralateral hearing aid fitting indispensable?	Jung, J.; Roh, K. J.; Moon, I. S.; Kim, S. H.; Hwang, K. R.; Lee, J. M.; Hou, J. H.; Choi, J. Y.	2016
Effect of comorbid diabetes and hypercholesterolemia on the prognosis of idiopathic sudden sensorineural hearing loss	Lin, C. F.; Lee, K. J.; Yu, S. S.; Lin, Y. S.	2016
Postoperative Rehabilitation Strategies Used by Adults With Cochlear Implants: A Pilot Study	Harris, M. S.; Capretta, N. R.; Henning, S. C.; Feeney, L.; Pitt, M. A.; Moberly, A. C.	2016

Non-auditory neurocognitive skills contribute to speech recognition in adults with cochlear implants	Moberly, A. C.; Houston, D. M.; Castellanos, I.	2016
Auditory function and speech discrimination abilities are impaired in progressive supranuclear palsy	Vitale, C.; Santangelo, G.; Allocca, R.; Abbate, T.; Peluso, S.; De Michele, G.; Moccia, M.; Picillo, M.; Tafuri, D.; Barone, P.; Cavaliere, M.	2016
Cross-Modal Functional Reorganization of Visual and Auditory Cortex in Adult Cochlear Implant Users Identified with fNIRS	Chen, L. C.; Sandmann, P.; Thorne, J. D.; Bleichner, M. G.; Debener, S.	2016
Age-Related Trajectories of Memory Function in Middle-Aged and Older Adults with and without Hearing Impairment	Wu, S. T.; Chiu, C. J.	2016
Laterality and unilateral deafness: Patients with congenital right ear deafness do not develop atypical language dominance	Van der Haegen, L.; Acke, F.; Vingerhoets, G.; Dhooge, I.; De Leenheer, E.; Cai, Q.; Brysbaert, M.	2016
On the relationship between auditory cognition and speech intelligibility in cochlear implant users: An ERP study	Finke, M.; Buchner, A.; Ruigendijk, E.; Meyer, M.; Sandmann, P.	2016
Internal Auditory Canal Decompression for Hearing Maintenance in Neurofibromatosis Type 2 Patients	Bernardeschi, D.; Peyre, M.; Collin, M.; Smail, M.; Sterkers, O.; Kalamarides, M.	2016
Contralateral Bimodal Stimulation: A Way to Enhance Speech Performance in Arabic-Speaking Cochlear Implant Patients	Abdeltawwab, M. M.; Khater, A.; El-Anwar, M. W.	2016

The Role of Electrode Placement in Bilateral Simultaneously Cochlear-Implanted Adult Patients	De Seta, D.; Nguyen, Y.; Bonnard, D.; Ferrary, E.; Godey, B.; Bakhos, D.; Mondain, M.; Deguine, O.; Sterkers, O.; Bernardeschi, D.; Mosnier, I.	2016
Treatment of sudden SNHL: Patient characteristics and outcomes	Ahmad, F.; Goddard, J. C.	2016
Diverse functional outcome spectrum of incus vibroplasty	Choi, B. Y.; Rhee, J.; Han, J. J.	2016
The effect of ITS injection frequency in sudden hearing loss	Evans, M.; Neumann, M.; Sugihara, E.; Babu, S.	2016
Correlating audiometric and vestibular function in Meniere's	McMullen, K.; Lin, C.; Harris, M.; Adunka, O. F.	2016
Cochlear implantation in patients with NF2: The UK experience	North, H. J. D.; Mawman, D.; Freeman, S. R. M.; Jiang, D.; Ramsden, J. D.; Axon, P.; Lloyd, S. K.	2016
Participant-generated Cochlear Implant Programs: Speech Recognition, Sound Quality, and Satisfaction	Dwyer, R. T.; Spahr, T.; Agrawal, S.; Hetlinger, C.; Holder, J. T.; Gifford, R. H.	2016
Results of Postoperative, CT-based, Electrode Deactivation on Hearing in Prelingually Deafened Adult Cochlear Implant Recipients	Labadie, R. F.; Noble, J. H.; Hedley-Williams, A. J.; Sunderhaus, L. W.; Dawant, B. M.; Gifford, R. H.	2016
Outcomes of Bone Anchored Hearing Aids (BAHA) for Single Sided Deafness in Nontraditional Candidates	Schwartz, S. R.; Kobylk, D.	2016
Modification of Osseointegrated Device Parameters to Improve Speech in Noise and Localization Ability: Clinical Recommendations	Hillyer, J.; Buchanan, P. C.; Elkins, E.; Watson, S. D.; Cloutier, F.; Backous, D. D.; Parbery-Clark, A.	2016

Word Recognition Variability With Cochlear Implants: The Degradation of Phonemic Sensitivity	Moberly, A. C.; Lowenstein, J. H.; Nittrouer, S.	2016
Cochlear Implants in Adults: Effects of Age and Duration of Deafness on Speech Recognition	Beyea, J. A.; McMullen, K. P.; Harris, M. S.; Houston, D. M.; Martin, J. M.; Bolster, V. A.; Adunka, O. F.; Moberly, A. C.	2016
Factors Affecting Outcomes in Cochlear Implant Recipients Implanted With a Perimodiolar Electrode Array Located in Scala Tympani	Holden, L. K.; Firszt, J. B.; Reeder, R. M.; Uchanski, R. M.; Dwyer, N. Y.; Holden, T. A.	2016
Hearing Preservation Outcomes With a Mid-Scala Electrode in Cochlear Implantation	Hunter, J. B.; Gifford, R. H.; Wanna, G. B.; Labadie, R. F.; Bennett, M. L.; Haynes, D. S.; Rivas, A.	2016
Long-term Incidence and Degree of Sensorineural Hearing Loss in Otosclerosis	Ishai, R.; Halpin, C. F.; Shin, J. J.; McKenna, M. J.; Quesnel, A. M.	2016
Computed Tomography Estimation of Cochlear Duct Length Can Predict Full Insertion in Cochlear Implantation	Johnston, J. D.; Scoffings, D.; Chung, M.; Baguley, D.; Donnelly, N. P.; Axon, P. R.; Gray, R. F.; Tysome, J. R.	2016
Clinical Implications of Word Recognition Differences in Earphone and Aided Conditions	McRackan, T. R.; Ahlstrom, J. B.; Clinkscales, W. B.; Meyer, T. A.; Dubno, J. R.	2016
Diagnostic Criteria for Detection of Vestibular Schwannomas in the VA Population	Pena, I.; Chew, E. Y.; Landau, B. P.; Breen, J. T.; Zevallos, J. P.; Vrabec, J. T.	2016

Speech Recognition of Bimodal Cochlear Implant Recipients Using a Wireless Audio Streaming Accessory for the Telephone	Wolfe, J.; Morais, M.; Schafer, E.	2016
Outcomes After Cochlear Implantation in the Very Elderly	Wong, D. J. Y.; Moran, M.; O'Leary, S. J.	2016
Objective and Subjective Outcome of a New Transcutaneous Bone Conduction Hearing Device: Half-year Follow-up of the First 12 Nordic Implantations	Eberhard, K. E.; Olsen, S. O.; Miyazaki, H.; Bille, M.; Caye-Thomasen, P.	2016
Cost-Utility of Bilateral Versus Unilateral Cochlear Implantation in Adults: A Randomized Controlled Trial	Smulders, Y. E.; van Zon, A.; Stegeman, I.; van Zanten, G. A.; Rinia, A. B.; Stokroos, R. J.; Free, R. H.; Maat, B.; Frijns, J. H.; Mylanus, E. A.; Huinck, W. J.; Topsakal, V.; Grolman, W.	2016
Mandarin Tone Identification in Cochlear Implant Users Using Exaggerated Pitch Contours	He, A.; Deroche, M. L.; Doong, J.; Jiradejvong, P.; Limb, C. J.	2016
Clinical Implication of the Threshold Equalizing Noise Test in Patients With Sudden Sensorineural Hearing Loss	Choi, J. E.; Lee, J. J.; Chung, W. H.; Cho, Y. S.; Hong, S. H.; Moon, I. J.	2016
Hearing Preservation After Cochlear Implantation May Improve Long-term Word Perception in the Electric-only Condition	Dalbert, A.; Huber, A.; Baumann, N.; Veraguth, D.; Roosli, C.; Pfiffner, F.	2016
Cochlear Implantation Improves Spatial Release From Masking and Restores Localization Abilities in Single-sided Deaf Patients	Grossmann, W.; Brill, S.; Moeltner, A.; Mlynski, R.; Hagen, R.; Radeloff, A.	2016
Long-term Hearing Preservation Outcomes After Cochlear Implantation for Electric-Acoustic Stimulation	Helbig, S.; Adel, Y.; Rader, T.; Stover, T.; Baumann, U.	2016

Development of a Squelch Effect in Adult Patients After Simultaneous Bilateral Cochlear Implantation	Kraaijenga, V. J. C.; van Zon, A.; Smulders, Y. E.; Ramakers, G. G. J.; Van Zanten, G. A.; Stokroos, R. J.; Huinck, W. J.; Frijns, J. H. M.; Free, R. H.; Grolman, W.	2016
Management of Cochlear Implant Electrode Migration	Rader, T.; Baumann, U.; Stover, T.; Weissgerber, T.; Adel, Y.; Leinung, M.; Helbig, S.	2016
Long-term Stability of the Active Middle-ear Implant with Floating-mass Transducer Technology: A Single-center Study	Schraven, S. P.; Gromann, W.; Rak, K.; Shehata-Dieler, W.; Hagen, R.; Mlynski, R.	2016
Speech Intelligibility in Noise With a Pinna Effect Imitating Cochlear Implant Processor	Wimmer, W.; Weder, S.; Caversaccio, M.; Kompis, M.	2016
Audiometric Comparison Between the First Patients With the Transcutaneous Bone Conduction Implant and Matched Percutaneous Bone Anchored Hearing Device Users	Rigato, C.; Reinfeldt, S.; Hakansson, B.; Jansson, K. J. F.; Hol, M. K.; Eeg-Olofsson, M.	2016
Comparing Loudness Tolerance and Acceptable Noise Level in Listeners With Hearing Loss	Franklin, C. A.; White, L. J.; Franklin, T. C.; Livengood, L. G.	2016
A Large Genome-Wide Association Study of Age-Related Hearing Impairment Using Electronic Health Records	Hoffmann, T. J.; Keats, B. J.; Yoshikawa, N.; Schaefer, C.; Risch, N.; Lustig, L. R.	2016
Monopolar Detection Thresholds Predict Spatial Selectivity of Neural Excitation in Cochlear Implants: Implications for Speech Recognition	Zhou, N.	2016

Monaural Beamforming in Bimodal Cochlear Implant Users: Effect of (A)symmetric Directivity and Noise Type	Devocht, E. M.; Janssen, A. M.; Chalupper, J.; Stokroos, R. J.; George, E. L.	2016
Cross-Modal and Intra-Modal Characteristics of Visual Function and Speech Perception Performance in Postlingually Deafened, Cochlear Implant Users	Kim, M. B.; Shim, H. Y.; Jin, S. H.; Kang, S.; Woo, J.; Han, J. C.; Lee, J. Y.; Kim, M.; Cho, Y. S.; Moon, I. J.; Hong, S. H.	2016
The impact of a support centre for people with sensory impairment living in rural Scotland	Smith, A.; Shepherd, A.; Jepson, R.; Mackay, S.	2016
Effects of sound amplification in self-perception of tinnitus and hearing loss in the elderly	Araujo Tde, M.; Iorio, M. C.	2016
Presbycusis: do we have a third ear?	Reis, L. R.; Escada, P.	2016
Speech perception in noise in unilateral hearing loss	Mondelli, M. F.; Dos Santos Mde, M.; Jose, M. R.	2016
Evaluation of Cochlear Implant Candidates using a Non-linguistic Spectrotemporal Modulation Detection Test	Choi, J. E.; Hong, S. H.; Won, J. H.; Park, H. S.; Cho, Y. S.; Chung, W. H.; Cho, Y. S.; Moon, I. J.	2016
The Effect of Short-Term Auditory Training on Speech in Noise Perception and Cortical Auditory Evoked Potentials in Adults with Cochlear Implants	Barlow, N.; Purdy, S. C.; Sharma, M.; Giles, E.; Narne, V.	2016
Change in Speech Perception and Auditory Evoked Potentials over Time after Unilateral Cochlear Implantation in Postlingually Deaf Adults	Purdy, S. C.; Kelly, A. S.	2016
An audiological profile of patients infected with multi-drug resistant tuberculosis at a district hospital in KwaZulu-Natal	Appana, D.; Joseph, L.; Paken, J.	2016
Assessing the relationship between locus of control and continued hearing aid use: Application of the Origin and Pawn Scales	Kelly-Campbell, R.; Allan, L.	2016
Executive functions and working memory capacity in speech communication under adverse conditions	Stenback, V.; Hallgren, M.; Larsby, B.	2016

Bimodal Stimulation with Cochlear Implant and Hearing Aid in Cases of Highly Asymmetrical Hearing Loss	Sanhueza, I.; Manrique, R.; Huarte, A.; de Erenchun, I. R.; Manrique, M.	2016
Recognition of Deep Band Modulated Consonants in Quiet and Noise in Older Individuals with and Without Hearing Loss	Shetty, H. N.; Kooknoor, V.	2016
Are Experienced Hearing Aid Users Faster at Grasping the Meaning of a Sentence Than Inexperienced Users? An Eye-Tracking Study	Habicht, J.; Kollmeier, B.; Neher, T.	2016
Spectrotemporal Modulation Sensitivity as a Predictor of Speech-Reception Performance in Noise With Hearing Aids	Bernstein, J. G. W.; Danielsson, H.; Hallgren, M.; Stenfelt, S.; Ronnberg, J.; Lunner, T.	2016
An Examination of Sources of Variability Across the Consonant-Nucleus-Consonant Test in Cochlear Implant Listeners	Bierer, J. A.; Spindler, E.; Bierer, S. M.; Wright, R.	2016
The Just-Meaningful Difference in Speech-to-Noise Ratio	McShefferty, D.; Whitmer, W. M.; Akeroyd, M. A.	2016
Rapid Release From Listening Effort Resulting From Semantic Context, and Effects of Spectral Degradation and Cochlear Implants	Winn, M. B.	2016
Subjective Listening Effort and Electrodermal Activity in Listening Situations with Reverberation and Noise	Holube, I.; Haeder, K.; Imbery, C.; Weber, R.	2016
The Influence of Cochlear Mechanical Dysfunction, Temporal Processing Deficits, and Age on the Intelligibility of Audible Speech in Noise for Hearing-Impaired Listeners	Johannesen, P. T.; Perez-Gonzalez, P.; Kalluri, S.; Blanco, J. L.; Lopez-Poveda, E. A.	2016
Comparison of Gated Audiovisual Speech Identification in Elderly Hearing Aid Users and Elderly Normal-Hearing Individuals: Effects of Adding Visual Cues to Auditory Speech Stimuli	Moradi, S.; Lidestam, B.; Ronnberg, J.	2016
ACEMg-mediated hearing preservation in cochlear implant patients receiving different electrode lengths (PROHEARING): Study protocol for a randomized controlled trial	Scheper, V.; Leifholz, M.; von der Leyen, H.; Keller, M.; Denkena, U.; Koch, A.; Karch, A.; Miller, J.; Lenarz, T.	2016

Cochlear implant in prelingually deafened oralist adults: Speech perception outcome, subjective benefits and quality of life improvement	Bellelli, S.; Forli, F.; Berrettini, S.; Turchetti, G.	2016
Cingulo-opercular function during word recognition in noise for older adults with hearing loss	Vaden Jr, Kenneth I; Kuchinsky, Stefanie E; Ahlstrom, Jayne B; Teubner-Rhodes, Susan E; Dubno, Judy R; Eckert, Mark A %J Experimental aging research	2016
Using speech recall in hearing aid fitting and outcome evaluation under ecological test conditions	Lunner, Thomas; Rudner, Mary; Rosenbom, Tove; √Ögren, Jessica; Ng, Elaine Hoi Ning %J Ear; hearing	2016
Effects of hearing loss and cognitive load on speech recognition with competing talkers	Meister, Hartmut; Schreitm√ºller, Stefan; Ortmann, Magdalene; R√§hlmann, Sebastian; Walger, Martin %J Frontiers in psychology	2016
Do Hearing Aids Improve Affect Perception?	Schmidt, J.; Herzog, D.; Scharenborg, O.; Janse, E.; VanDijk, P.; Baskent, D.; Gaudrain, E.; DeKleine, E.; Wagner, A.; Lanting, C.	2016

Speech recognition in noise by younger and older adults: effects of age, hearing loss, and temporal resolution	Vermeire, Katrien; Knoop, Allart; Boel, Carolien; Auwers, Silke; Schenus, Laura; Talaveron-Rodriguez, Marilina; De Boom, Chlo√´; De Sloovere, Marleen %J Annals of Otology, Rhinology; Laryngology	2016
Optimal treatment of jugular foramen schwannomas: long-term outcome of a multidisciplinary approach for a series of 29 cases in a single institute	Ryu, S. M.; Lee, J. I.; Park, K.; Choi, J. W.; Kong, D. S.; Nam, D. H.; Jeong, H. S.; Cho, Y. S.; Seol, H. J.	2017
Patient satisfaction after auditory implant surgery: ten-year experience from a single implanting unit center	Monini, S.; Bianchi, A.; Talamonti, R.; Atturo, F.; Filippi, C.; Barbara, M.	2017
The hearing benefit of cochlear implantation for individuals with unilateral hearing loss, but no tinnitus	Skarzynski, H.; Lorens, A.; Kruszynska, M.; Obrycka, A.; Pastuszak, D.; Skarzynski, P. H.	2017
Long term benefit of bone anchored hearing systems in single sided deafness	Kompis, M.; Wimmer, W.; Caversaccio, M.	2017
Long-term results of hearing preservation cochlear implant surgery in patients with residual low frequency hearing	Moteki, H.; Nishio, S. Y.; Miyagawa, M.; Tsukada, K.; Iwasaki, S.; Usami, S. I.	2017
Speech recognition in bilaterally cochlear implanted adults in Tokyo, Japan	Ohta, Y.; Kawano, A.; Kawaguchi, S.; Shirai, K.; Tsukahara, K.	2017

Work activity in patients treated with cochlear implants	Huarte, A.; Martinez-Lopez, M.; Manrique-Huarte, R.; Erviti, S.; Calavia, D.; Alonso, C.; Manrique, M.	2017
Changes in Psychosocial Measures After a 6-Week Field Trial	Desjardins, J. L.; Doherty, K. A.	2017
Exploring the Relevance of Items in the Communicative Participation Item Bank (CPIB) for Individuals With Hearing Loss	Miller, C. W.; Baylor, C. R.; Birch, K.; Yorkston, K. M.	2017
Ecological Momentary Assessment: Feasibility, Construct Validity, and Future Applications	Timmer, B. H. B.; Hickson, L.; Launer, S.	2017
Relation Between Listening Effort and Speech Intelligibility in Noise	Krueger, M.; Schulte, M.; Zokoll, M. A.; Wagener, K. C.; Meis, M.; Brand, T.; Holube, I.	2017
The Effects of Service-Delivery Model and Purchase Price on Hearing-Aid Outcomes in Older Adults: A Randomized Double-Blind Placebo-Controlled Clinical Trial	Humes, L. E.; Rogers, S. E.; Quigley, T. M.; Main, A. K.; Kinney, D. L.; Herring, C.	2017
Development of a Shortened Version of the Spatial Hearing Questionnaire (SHQ-S) for Screening Spatial-Hearing Ability	Ou, H.; Perreau, A.; Tyler, R. S.	2017
Do Older Listeners With Hearing Loss Benefit From Dynamic Pitch for Speech Recognition in Noise?	Shen, J.; Souza, P. E.	2017
A "Goldilocks" Approach to Hearing-Aid Self-Fitting: User Interactions	Boothroyd, A.; Mackersie, C.	2017
Autonomic Nervous System Responses to Hearing-Related Demand and Evaluative Threat	Mackersie, C. L.; Kearney, L.	2017
Diagnosing and Screening in a Minority Language: A Validation Study	Zokoll, M. A.; Wagener, K. C.; Kollmeier, B.	2017
Cochlear implantation for single-sided deafness and tinnitus suppression	Holder, J. T.; O'Connell, B.; Hedley-Williams, A.; Wanna, G.	2017
Long-term outcomes of endolymphatic sac shunting with local steroids for Meniere's disease	Wick, C. C.; Manzoor, N. F.; McKenna, C.; Semaan, M. T.; Megerian, C. A.	2017

Initial Results of the Early Auditory Referral-Primary Care (EAR-PC) Study	Zazove, P.; Plegue, M. A.; Kileny, P. R.; McKee, M. M.; Schleicher, L. S.; Green, L. A.; Sen, A.; Rapai, M. E.; Guetterman, T. C.; Mulhem, E.	2017
How your ears can tell what is hidden in your heart: wild-type transthyretin amyloidosis as potential cause of sensorineural hearing loss inelderly-AmyloDEAFNESS pilot study	Bequignon, E.; Guellich, A.; Barthier, S.; Raynal, M.; Pruliere-Escabasse, V.; Canoui-Poitrine, F.; Coste, A.; Damy, T.	2017
Sensorineural hearing loss in takayasu's arteritis	Kimyon, U.; Esatoglu, S. N.; Kara, E.; Atas, A.; Gunay, E. E.; Gozen, E. D.; Karaman, E.; Hamuryudan, V.; Yazici, H.; Seyahi, E.	2017
Impact of Lipid-Inflammatory-Oxidative Metabolism on Auditory Skills after Hearing Aid Fitting in the Elderly	Lessa, A. H.; Cruz, Ibmd; Manica-Cattani, M. F.; Moresco, R. N.; Duarte, Mmmf; Costa, M. J.	2017
Vestibular schwannoma resection with ipsilateral simultaneous cochlear implantation in patients with normal contralateral hearing	Sanna, M.; Del Mar Medina, M.; Macak, A.; Rossi, G.; Sozzi, V.; Prasad, S. C.	2017
Consequences of stimulus type on higher-order processing in single-sided deaf cochlear implant users	Finke, M.; Sandmann, P.; Bonitz, H.; Kral, A.; Buchner, A.	2017
Comparison of the HiFocus Mid-Scala and HiFocus 1J electrode array: Angular insertion depths and speech perception outcomes	Van Der Jagt, M. A.; Briaire, J. J.; Verbist, B. M.; Frijns, J. H. M.	2017
Take-Home Trial Comparing Fast Fourier Transformation-Based and Filter Bank-Based Cochlear Implant Speech Coding Strategies	De Jong, M. A. M.; Briaire, J. J.; Frijns, J. H. M.	2017

Direct acoustic stimulation with the codacsTM	Maier, H.; Busch, S.; Kludt, E.; Grossohmichen, M.; Lenarz, T.	2017
Correlation between subjective and objective hearing tests after unilateral and bilateral cochlear implantation	Ramakers, G. G. J.; Smulders, Y. E.; Van Zon, A.; Van Zanten, G. A.; Grolman, W.; Stegeman, I.	2017
Promoting independence, health and wellbeing for older people: a feasibility study of computer-aided health and social risk appraisal system in primary care	Walters, K.; Kharicha, K.; Goodman, C.; Handley, M.; Manthorpe, J.; Cattan, M.; Morris, S.; Clarke, C. S.; Round, J.; Iliffe, S.	2017
Translation of the international outcome inventory for hearing aids into Portuguese from Portugal	Paiva, S. M.; Simoes, J. F.; Paiva, A. M.; Sousa, F. J.; Bebear, J. P.	2017
Evaluating the short-term and long-term effects of an internet-based aural rehabilitation programme for hearing aid users in general clinical practice: a randomised controlled trial	Malmberg, M.; Lunner, T.; Kahari, K.; Andersson, G.	2017
Evaluation of vestibular evoked myogenic potentials (VEMP) and electrocochleography for the diagnosis of Meniere's disease	Lamounier, P.; de Souza, T. S. A.; Gobbo, D. A.; Bahmad Jr, F.	2017
Profile and prevalence of hearing complaints in the elderly	Bauer, M. A.; Zanella, A. K.; Filho, I. G.; de Carli, G.; Teixeira, A. R.; Bos, A. J. G.	2017
Hearing handicap in patients with chronic kidney disease: a study of the different classifications of the degree of hearing loss	Costa, K. V. T. D.; Ferreira, S. M. S.; Menezes, P. D. L.	2017
Speech perception in noise in the elderly: interactions between cognitive performance, depressive symptoms, and education	de Carvalho, L. M. A.; Gonsalez, E. C. D. M.; Iorio, M. C. M.	2017

Is there a best side for cochlear implants in post-lingual patients?	Amaral, M. S. A. D.; Damico, T. A.; Goncales, A. S.; Reis, A. C. M. B.; Isaac, M. D. L.; Massuda, E. T.; Hyppolito, M. A.	2017
Screening of hearing in elderly people: assessment of accuracy and reproducibility of the whispered voice test	Labanca, L.; Guimaraes, F. S.; Costa-Guarisco, L. P.; Couto, E. D. B.; Goncalves, D. U.	2017
Effects of hearing loss on speech recognition under distracting conditions and working memory in the elderly	Na, W.; Kim, G.; Kim, G.; Han, W.; Kim, J.	2017
Auditory dysfunction in patients with Huntington's disease	Profant, O.; Roth, J.; Bures, Z.; Balogova, Z.; Liskova, I.; Betka, J.; Syka, J.	2017
Development of a home-based auditory training to improve speech recognition on the telephone for patients with cochlear implants: A randomised trial	Ihler, F.; Blum, J.; Steinmetz, G.; Weiss, B. G.; Zirn, S.; Canis, M.	2017
Hearing aid fitting for visual and hearing impaired patients with Usher syndrome type IIa	Hartel, B. P.; Agterberg, M. J. H.; Snik, A. F.; Kunst, H. P. M.; van Opstal, A. J.; Bosman, A. J.; Pennings, R. J. E.	2017
Diabetes mellitus and sensorineural hearing loss: is there an association? Baseline of the Brazilian Longitudinal Study of Adult Health (ELSA-Brasil)	Samelli, A. G.; Santos, I. S.; Moreira, R. R.; Rabelo, C. M.; Rolim, L. P.; Bensenor, I. J.; Lotufo, P. A.	2017
Person-centred cochlear implant care: Assessing the need for clinic intervention in adults with cochlear implants using a dual approach of an online speech recognition test and a questionnaire	Cullington, H. E.; Agyemang-Prempeh, A.	2017
Is the digit triplet test an effective and acceptable way to assess speech recognition in adults using cochlear implants in a home environment?	Cullington, H. E.; Aidi, T.	2017

Health-related quality of life outcomes following adult cochlear implantation: A prospective cohort study	Sladen, D. P.; Peterson, A.; Schmitt, M.; Olund, A.; Teece, K.; Dowling, B.; DeJong, M.; Breneman, A.; Beatty, C. W.; Carlson, M. L.; Neff, B. A.; Hughes-Borst, B.; Driscoll, C. L.	2017
Impact of cognition and noise reduction on speech perception in adults with unilateral cochlear implants	Purdy, S. C.; Welch, D.; Giles, E.; Morgan, C. L.; Tenhagen, R.; Kuruvilla-Mathew, A.	2017
Cochlear implants in the management of hearing loss in Neurofibromatosis Type 2	Harris, F.; Tysome, J. R.; Donnelly, N.; Durie-Gair, J.; Crundwell, G.; Tam, Y. C.; Knight, R. D.; Vanat, Z. H.; Folland, N.; Axon, P.	2017
Auditory performance and subjective benefits in adults with congenital or prelinguistic deafness who receive cochlear implants during adulthood	Duchesne, L.; Millette, I.; Bherer, M.; Gobeil, S.	2017
Effect of extreme adaptive frequency compression in bimodal listeners on sound localization and speech perception	Veugen, L. C. E.; Chalupper, J.; Mens, L. H. M.; Snik, A. F. M.; van Opstal, A. J.	2017
Time-compressed speech test in the elderly	Arceno, R. S.; Scharlach, R. C.	2017
TELEGRAM: contribution in assistive technology indication for individuals with hearing impairment	Jacob, R. T.; Lopes, N. B.; Cruz, A. D.; Alves, T. K.; Santos, L. G.; Angelo, T. C.; Mondelli, M. F.; Moret, A. L.	2017
Using prosody to infer discourse prominence in cochlear-implant users and normal-hearing listeners	Huang, Y. T.; Newman, R. S.; Catalano, A.; Goupell, M. J.	2017

Hearing Loss and Risk of Mild Cognitive Impairment and Dementia: Findings from the Singapore Longitudinal Ageing Study	Heywood, R.; Gao, Q.; Nyunt, M. S. Z.; Feng, L.; Chong, M. S.; Lim, W. S.; Yap, P.; Lee, T. S.; Yap, K. B.; Wee, S. L.; Ng, T. P.	2017
Auditory Distraction and Acclimatization to Hearing Aids	Dawes, P.; Munro, K. J.	2017
Combined Electric and Acoustic Stimulation With Hearing Preservation: Effect of Cochlear Implant Low-Frequency Cutoff on Speech Understanding and Perceived Listening Difficulty	Gifford, R. H.; Davis, T. J.; Sunderhaus, L. W.; Menapace, C.; Buck, B.; Crosson, J.; O'Neill, L.; Beiter, A.; Segel, P.	2017
Rehabilitation and Psychosocial Determinants of Cochlear Implant Outcomes in Older Adults	Tang, L.; Thompson, C. B.; Clark, J. H.; Ceh, K. M.; Yeagle, J. D.; Francis, H. W.	2017
The Effect of Aging and Priming on Same/Different Judgments Between Text and Partially Masked Speech	Freyman, R. L.; Terpening, J.; Costanzi, A. C.; Helfer, K. S.	2017
Neural Correlates of Selective Attention With Hearing Aid Use Followed by ReadMyQuips Auditory Training Program	Rao, A.; Rishiq, D.; Yu, L.; Zhang, Y.; Abrams, H.	2017
Visual Temporal Acuity Is Related to Auditory Speech Perception Abilities in Cochlear Implant Users	Jahn, K. N.; Stevenson, R. A.; Wallace, M. T.	2017
The Benefits of Increased Sensation Level and Bandwidth for Spatial Release From Masking	Jakien, K. M.; Kampel, S. D.; Gordon, S. Y.; Gallun, F. J.	2017
Comparison of the Spectral-Temporally Modulated Ripple Test With the Arizona Biomedical Institute Sentence Test in Cochlear Implant Users	Lawler, M.; Yu, J.; Aronoff, J. M.	2017
Pre- and Postoperative Binaural Unmasking for Bimodal Cochlear Implant Listeners	Sheffield, B. M.; Schuchman, G.; Bernstein, J. G. W.	2017

Self-Adjustment of Upper Electrical Stimulation Levels in CI Programming and the Effect on Auditory Functioning	Vroegop, J. L.; Dingemanse, J. G.; van der Schroeff, M. P.; Metselaar, R. M.; Goedegebure, A.	2017
Evaluation of Long-Term Cochlear Implant Use in Subjects With Acquired Unilateral Profound Hearing Loss: Focus on Binaural Auditory Outcomes	Mertens, G.; De Bodt, M.; Van de Heyning, P.	2017
Improving Mobile Phone Speech Recognition by Personalized Amplification: Application in People with Normal Hearing and Mild-to-Moderate Hearing Loss	Kam, A. C.; Sung, J. K.; Lee, T.; Wong, T. K.; van Hasselt, A.	2017
Directional Microphone Contralateral Routing of Signals in Cochlear Implant Users: A Within-Subjects Comparison	Wimmer, W.; Kompis, M.; Stieger, C.; Caversaccio, M.; Weder, S.	2017
Impact of Hearing Aid Technology on Outcomes in Daily Life III: Localization	Johnson, J. A.; Xu, J. J.; Cox, R. M.	2017
Prospective Study of Gastroesophageal Reflux, Use of Proton Pump Inhibitors and H2-Receptor Antagonists, and Risk of Hearing Loss	Lin, B. M.; Curhan, S. G.; Wang, M. L.; Jacobson, B. C.; Eavey, R.; Stankovic, K. M.; Curhan, G. C.	2017
Burden of Hearing Loss on Communication Partners and Its Influence on Pursuit of Hearing Evaluation	Schulz, K. A.; Modeste, N.; Lee, J. W.; Roberts, R.; Saunders, G. H.; Witsell, D. L.	2017
Speech Intelligibility as a Cue for Acceptable Noise Levels	Recker, K. L.; Micheyl, C.	2017
Speech perception and auditory performance following cochlear implantation in elderly Koreans	Lee, S.; Park, H. J.; Cho, H. H.; Cho, Y. B.	2017
Hearing loss and disability exit: Measurement issues and coping strategies	Christensen, V. T.; Datta Gupta, N.	2017
Baha implant as a hearing solution for single-sided deafness after retrosigmoid approach for the vestibular schwannoma: audiological results	Boucek, J.; Vokral, J.; Cerny, L.; Chovanec, M.; Zabrodsky, M.; Zverina, E.; Betka, J.; Skrivan, J.	2017

Safety and effectiveness of the Bonebridge transcutaneous active direct-drive bone-conduction hearing implant at 1-year device use	Schmerber, S.; Deguine, O.; Marx, M.; Van de Heyning, P.; Sterkers, O.; Mosnier, I.; Garin, P.; Godey, B.; Vincent, C.; Venail, F.; Mondain, M.; Deveze, A.; Lavieille, J. P.; Karkas, A.	2017
Cochlear implantation with the nucleus slim modiolar electrode (CI532): a preliminary experience	Cuda, D.; Murri, A.	2017
Feasibility of an implanted microphone for cochlear implant listening	Gerard, J. M.; Demanez, L.; Salmon, C.; Vanpoucke, F.; Walraevens, J.; Plasmans, A.; De Siati, D.; Lefebvre, P.	2017
A multicenter study on objective and subjective benefits with a transcutaneous bone-anchored hearing aid device: first Nordic results	Hougaard, D. D.; Boldsen, S. K.; Jensen, A. M.; Hansen, S.; Thomassen, P. C.	2017
Benefit of the UltraZoom beamforming technology in noise in cochlear implant users	Mosnier, I.; Mathias, N.; Flament, J.; Amar, D.; Liagre-Callies, A.; Borel, S.; Ambert-Dahan, E.; Sterkers, O.; Bernardeschi, D.	2017
Off the ear with no loss in speech understanding: comparing the RONDO and the OPUS 2 cochlear implant audio processors	Dazert, S.; Thomas, J. P.; Buchner, A.; Muller, J.; Hempel, J. M.; Lowenheim, H.; Mlynski, R.	2017
Sensitivity and specificity of the abbreviated profile of hearing aid benefit (APHAB)	Lohler, J.; Grabner, F.; Wollenberg, B.; Schlattmann, P.; Schonweiler, R.	2017

Associations between the probabilities of frequency-specific hearing loss and unaided APHAB scores	Lohler, J.; Wollenberg, B.; Schlattmann, P.; Hoang, N.; Schonweiler, R.	2017
Patient benefit from Cochlear implantation in single-sided deafness: a 1-year follow-up	Louza, J.; Hempel, J. M.; Krause, E.; Berghaus, A.; Muller, J.; Braun, T.	2017
Improvement of speech perception in quiet and in noise without decreasing localization abilities with the bone conduction device Bonebridge	Weiss, R.; Leinung, M.; Baumann, U.; Weisgerber, T.; Rader, T.; Stover, T.	2017
Bone anchored hearing implants without skin thinning: the Gruppo Otologico surgical and audiological experience	Caruso, A.; Giannuzzi, A. L.; Sozzi, V.; Sanna, M.	2017
Effect of hearing aid release time and presentation level on speech perception in noise in elderly individuals with hearing loss	Pottackal Mathai, J.; Mohammed, H.	2017
Audiometric evaluation in patients with Alzheimer's disease	Villeneuve, A.; Hommet, C.; Aussedat, C.; Lescanne, E.; Reffet, K.; Bakhos, D.	2017
Variability of word discrimination scores in clinical practice and consequences on their sensitivity to hearing loss	Moulin, A.; Bernard, A.; Tordella, L.; Vergne, J.; Gisbert, A.; Martin, C.; Richard, C.	2017
Cochlear implantation in patients with definite Meniere's disease	Prenzler, N. K.; Bultmann, E.; Giourgas, A.; Steffens, M.; Salcher, R. B.; Stolle, S.; Lesinski-Schiedat, A.; Lenarz, T.; Durisin, M.	2017
Age-related changes in auditory processing and speech perception: cross-sectional and longitudinal analyses	Babkoff, H.; Fostick, L.	2017
Effort not speed characterizes comprehension of spoken sentences by older adults with mild hearing impairment	Ayasse, N. D.; Lash, A.; Wingfield, A.	2017

In patients undergoing cochlear implantation, psychological burden affects tinnitus and the overall outcome of auditory rehabilitation	Bruggemann, P.; Szczepek, A. J.; Klee, K.; Grabel, S.; Mazurek, B.; Olze, H.	2017
Analysis of Audiometric Differences of Patients with and without Tinnitus in a Large Clinical Database	Gollnast, D.; Tziridis, K.; Krauss, P.; Schilling, A.; Hoppe, U.; Schulze, H.	2017
Outer hair cell and auditory nerve function in speech recognition in quiet and in background noise	Hoben, R.; Easow, G.; Pevzner, S.; Parker, M. A.	2017
Intracochlear recordings of acoustically and electrically evoked potentials in nucleus hybrid L24 cochlear implant users and their relationship to speech perception	Kim, J. R.; Tejani, V. D.; Abbas, P. J.; Brown, C. J.	2017
Auditory and Non-Auditory Contributions for Unaided Speech Recognition in Noise as a Function of Hearing Aid Use	Gieseler, A.; Tahden, M. A. S.; Thiel, C. M.; Wagener, K. C.; Meis, M.; Colonius, H.	2017
Partial Visual Loss Affects Self-reports of Hearing Abilities Measured Using a Modified Version of the Speech, Spatial, and Qualities of Hearing Questionnaire	Kolarik, A. J.; Raman, R.; Moore, B. C. J.; Cirstea, S.; Gopalakrishnan, S.; Pardhan, S.	2017
Cognitive Processing Speed, Working Memory, and the Intelligibility of Hearing Aid-Processed Speech in Persons with Hearing Impairment	Yumba, W. K.	2017
The Efficacy of Short-term Gated Audiovisual Speech Training for Improving Auditory Sentence Identification in Noise in Elderly Hearing Aid Users	Moradi, S.; Wahlin, A.; Hallgren, M.; Ronnberg, J.; Lidestam, B.	2017
Speech perception enhancement in elderly hearing aid users using an auditory training program for mobile devices	Yu, J.; Jeon, H.; Song, C.; Han, W.	2017
The studying multiple outcomes after aural rehabilitative treatment study: Study design and baseline results	Li, L.; Blake, C.; Sung, Y.; Shpritz, B.; Chen, D.; Genther, D. J.; Betz, J.; Lin, F. R.	2017

Psychometric characteristics of the chronic Otitis media questionnaire 12 (COMQ - 12): Stability of factor structure and replicability shown by the Serbian version	Bukurov, B.; Arsovic, N.; Grujicic, S. S.; Haggard, M.; Spencer, H.; Marinkovic, J. E.	2017
Speech intelligibility and subjective benefit in single-sided deaf adults after cochlear implantation	Finke, M.; Strauss-Schier, A.; Kludt, E.; Buchner, A.; Illg, A.	2017
Early cortical metabolic rearrangement related to clinical data in idiopathic sudden sensorineural hearing loss	Micarelli, A.; Chiaravalloti, A.; Viziano, A.; Danieli, R.; Schillaci, O.; Alessandrini, M.	2017
The pattern and degree of capsular fibrous sheaths surrounding cochlear electrode arrays	Ishai, R.; Herrmann, B. S.; Nadol, J. B.; Quesnel, A. M.	2017
Adding simultaneous stimulating channels to reduce power consumption in cochlear implants	Langner, F.; Saoji, A. A.; Buchner, A.; Nogueira, W.	2017
Genetic variants in the peripheral auditory system significantly affect adult cochlear implant performance	Shearer, A. E.; Eppsteiner, R. W.; Frees, K.; Tejani, V.; Sloan-Heggen, C. M.; Brown, C.; Abbas, P.; Dunn, C.; Hansen, M. R.; Gantz, B. J.; Smith, R. J. H.	2017
Masked speech perception across the adult lifespan: Impact of age and hearing impairment	Goossens, T.; Vercammen, C.; Wouters, J.; van Wieringen, A.	2017
Effects of attention on the speech reception threshold and pupil response of people with impaired and normal hearing	Koelewijn, T.; Versfeld, N. J.; Kramer, S. E.	2017
Speech reception with different bilateral directional processing schemes: Influence of binaural hearing, audiometric asymmetry, and acoustic scenario	Neher, T.; Wagener, K. C.; Latzel, M.	2017

Masking release with changing fundamental frequency: Electric acoustic stimulation resembles normal hearing subjects	Auinger, A. B.; Riss, D.; Liepins, R.; Rader, T.; Keck, T.; Keintzel, T.; Kaider, A.; Baumgartner, W. D.; Gstoettner, W.; Arnoldner, C.	2017
Speech enhancement based on neural networks improves speech intelligibility in noise for cochlear implant users	Goehring, T.; Bolner, F.; Monaghan, J. J. M.; van Dijk, B.; Zarowski, A.; Bleeck, S.	2017
Impact of stimulus-related factors and hearing impairment on listening effort as indicated by pupil dilation	Ohlenforst, B.; Zekveld, A. A.; Lunner, T.; Wendt, D.; Naylor, G.; Wang, Y.; Versfeld, N. J.; Kramer, S. E.	2017
A physiologically-inspired model reproducing the speech intelligibility benefit in cochlear implant listeners with residual acoustic hearing	Zamaninezhad, L.; Hohmann, V.; Buchner, A.; Schadler, M. R.; Jurgens, T.	2017
Cochlear implant treatment of patients with single-sided deafness or asymmetric hearing loss	Arndt, S.; Laszig, R.; Aschendorff, A.; Hassepass, F.; Beck, R.; Wesarg, T.	2017
Adult speech recognition index using new lists of monosyllables and disyllables	Costa, L. D.; Hennig, T. R.; Costa, M. J.; De Almeida Vaucher, A. V.	2017
New lists of monosyllables and disyllables in the evaluation of speech recognition in adults and the elderly	Dalla Costa, L.; De Almeida Vaucher, A. V.; Costa, M. J.; Hennig, T. R.	2017
Dichotic hearing in elderly hearing aid users who choose to use a single-ear device	Ribas, A.; Mafra, N.; Marques, J.; Mottecy, C.; Silvestre, R.; Kozlowski, L.	2017

Evaluation of Participation Restriction: The self-perception of elderly people with hearing impairment	De Noronha Souza, A. E. L.; Zampronio, C. D. P.; Melo, I. M. M.	2017
Correlation of the performance of patients with cochlear implants in speech perception tests and time of sensory deprivation	De Campos Salvato, C.; De Araujo, S. R. S.; De Souza Vieira, S.; Soares, A. D.; Chiari, B. M.; Muller, R.	2017
Threshold of recognition of judgments in silence and noise in a non-institutionalized group of elderly	Pinto, M. F.; Lessa, A. H.; Olchik, M. R.; Teixeira, A. R.; Santos, J. P. N. A.; Neves, C. Z.	2017
Clinical outcomes with the KansoTM off-the-ear cochlear implant sound processor	Mauger, S. J.; Jones, M.; Nel, E.; Del Dot, J.	2017
French-Canadian translation and validation of four questionnaires assessing hearing impairment and handicap	Vincent, C.; Gagne, J. P.; Leroux, T.; Clothier, A.; Lariviere, M.; Dumont, F. S.; Gendron, M.	2017
How directional microphones affect speech recognition, listening effort and localisation for listeners with moderate-to-severe hearing loss	Picou, E. M.; Ricketts, T. A.	2017
Referral rates of postlingually deafened adult hearing aid users for a cochlear implant candidacy assessment	Looi, V.; Bluett, C.; Boisvert, I.	2017
Output signal-to-noise ratio and speech perception in noise: effects of algorithm	Miller, C. W.; Bentler, R. A.; Wu, Y. H.; Lewis, J.; Tremblay, K.	2017
Development and evaluation of the British English coordinate response measure speech-in-noise test as an occupational hearing assessment tool	Semeraro, H. D.; Rowan, D.; van Besouw, R. M.; Allsopp, A. A.	2017
Analysing use of the Chinese HHIE-S for hearing screening of elderly in a northeastern industrial area of China	Wang, Y.; Mo, L. Y.; Li, Y. G.; Zheng, Z. W.; Qi, Y.	2017
Cochlear implant effectiveness in postlingual single-sided deaf individuals: what's the point?	Finke, M.; Bonitz, H.; Lyxell, B.; Illg, A.	2017

Evaluation of a wireless remote microphone in bimodal cochlear implant recipients	Vroegop, J. L.; Dingemanse, J. G.; Homans, N. C.; Goedegebure, A.	2017
Stages of change in audiology: comparison of three self-assessment measures	Ingo, E.; Brannstrom, K. J.; Andersson, G.; Lunner, T.; Laplante-Levesque, A.	2017
A multicentre study evaluating the effects of the Swedish ACE programme	Oberg, M.	2017
Psychometric properties of the hearing handicap questionnaire: a Kannada (South-Indian) translation	Thammaiah, S.; Manchaiah, V.; Easwar, V.; Krishna, R.; McPherson, B.	2017
The use of cochlear's SCAN and wireless microphones to improve speech understanding in noise with the Nucleus6 CP900 processor	De Ceulaer, G.; Pascoal, D.; Vanpoucke, F.; Govaerts, P. J.	2017
Laboratory evaluation of an optimised internet-based speech-in-noise test for occupational high-frequency hearing loss screening: Occupational Earcheck	Rashid, M. S.; Leensen, M. C. J.; de Laat, Japm; Dreschler, W. A.	2017
Selection of test-setup parameters to target specific signal-to-noise regions in speech-on-speech intelligibility testing	Ronne, F. M.; Laugesen, S.; Jensen, N. S.	2017
Hearing impairment is common among Saami adults in Northern Finland	Lohi, V.; Ohtonen, P.; Aikio, P.; Sorri, M.; Maki-Torkko, E.; Hannula, S.	2017
Cochlear implantation improves hearing and vertigo in patients after removal of vestibular schwannoma	Roemer, A.; Lenarz, T.; Lesinski-Schiedat, A.	2017
Intratympanic injection of dexamethasone and electrocochleographic data in cases of definite one sided refractory meniere's disease	Satri, S. D.; Gharibi, R.; Nejadian, F.; Yazdani, N.; Hoseinabadi, R.; Rezazadeh, N.; Firouzifar, M. R.; Babaei, S.	2017
Personal Sound Amplification Products vs a Conventional Hearing Aid for Speech Understanding in Noise	Reed, N. S.; Betz, J.; Kendig, N.; Korczak, M.; Lin, F. R.	2017

Use of Adult Patient Focus Groups to Develop the Initial Item Bank for a Cochlear Implant Quality-of-Life Instrument	McRackan, T. R.; Velozo, C. A.; Holcomb, M. A.; Camposeo, E. L.; Hatch, J. L.; Meyer, T. A.; Lambert, P. R.; Melvin, C. L.; Dubno, J. R.	2017
Objective and Subjective Measures of Simultaneous vs Sequential Bilateral Cochlear Implants in Adults: A Randomized Clinical Trial	Kraaijenga, V. J. C.; Ramakers, G. G. J.; Smulders, Y. E.; van Zon, A.; Stegeman, I.; Smit, A. L.; Stokroos, R. J.; Hendrice, N.; Free, R. H.; Maat, B.; Frijns, J. H. M.; Briaire, J. J.; Mylanus, E. A. M.; Huinck, W. J.; Van Zanten, G. A.; Grolman, W.	2017
Factors Affecting Sentence-in-Noise Recognition for Normal Hearing Listeners and Listeners with Hearing Loss	Hwang, J. S.; Kim, K. H.; Lee, J. H.	2017
Substitution Patterns of Phoneme Errors in Hearing Aid and Cochlear Implant Users	Han, W.; Chun, H.; Kim, G.; Jin, I. K.	2017
Improvement of older-person-specific QOL after hearing aid fitting and its relation to social interaction	Yamada, Y.; Svejdikova, B.; Kisvetrova, H.	2017
Auditory changes in acromegaly	Tabur, S.; Korkmaz, H.; Baysal, E.; Hatipoglu, E.; Aytac, I.; Akarsu, E.	2017
Hearing status in patients with rheumatoid arthritis	Ahmadzadeh, A.; Daraei, M.; Jalessi, M.; Peyvandi, A. A.; Amini, E.; Ranjbar, L. A.; Daneshi, A.	2017
Genetic severity score predicts clinical phenotype in NF2	Halliday, D.; Emmanouil, B.; Pretorius, P.; MacKeith, S.; Painter, S.; Tomkins, H.; Evans, D. G.; Parry, A.	2017

High volume multidisciplinary surgical team experience: Reduced operative times and better patient outcomes	Eisert, C.; Tai, T.; Fisher, L. M.; Giannotta, S. L.; Friedman, R. A.	2017
Auditory brainstem implants in neurofibromatosis type 2: Early and long-term results	Matthies, C.; Gelbrich, G.; Mlynski, R.; Hagen, R.; Shehata-Dieler, W.	2017
Magnified Neural Envelope Coding Predicts Deficits in Speech Perception in Noise	Millman, R. E.; Mattys, S. L.; Gouws, A. D.; Prendergast, G.	2017
Suprathreshold compound action potential amplitude as a measure of auditory function in cochlear implant users	Scheperle, R. A.	2017
Motivation to Address Self-Reported Hearing Problems in Adults With Normal Hearing Thresholds	Alicea, C. C. M.; Doherty, K. A.	2017
Self-Assessed Hearing Handicap in Older Adults With Poorer-Than-Predicted Speech Recognition in Noise	Eckert, M. A.; Matthews, L. J.; Dubno, J. R.	2017
Judgments of Emotion in Clear and Conversational Speech by Young Adults With Normal Hearing and Older Adults With Hearing Impairment	Morgan, S. D.; Ferguson, S. H.	2017
Speech Recognition and Cognitive Skills in Bimodal Cochlear Implant Users	Hua, H.; Johansson, B.; Magnusson, L.; Lyxell, B.; Ellis, R. J.	2017
Working Memory and Speech Recognition in Noise Under Ecologically Relevant Listening Conditions: Effects of Visual Cues and Noise Type Among Adults With Hearing Loss	Miller, C. W.; Stewart, E. K.; Wu, Y. H.; Bishop, C.; Bentler, R. A.; Tremblay, K.	2017
Speech Recognition in Adults With Cochlear Implants: The Effects of Working Memory, Phonological Sensitivity, and Aging	Moberly, A. C.; Harris, M. S.; Boyce, L.; Nittrouer, S.	2017
Working Memory and Speech Comprehension in Older Adults With Hearing Impairment	Nagaraj, N. K.	2017
Auditory Training for Adults Who Have Hearing Loss: A Comparison of Spaced Versus Massed Practice Schedules	Tye-Murray, N.; Spehar, B.; Barcroft, J.; Sommers, M.	2017
Speech Rate Normalization and Phonemic Boundary Perception in Cochlear-Implant Users	Jaekel, B. N.; Newman, R. S.; Goupell, M. J.	2017

Speech Understanding in Noise by Patients With Cochlear Implants Using a Monaural Adaptive Beamformer	Dorman, M. F.; Natale, S.; Spahr, A.; Castioni, E.	2017
Recognition and Comprehension of "Narrow Focus" by Young Adults With Prelingual Hearing Loss Using Hearing Aids or Cochlear Implants	Segal, O.; Kishon-Rabin, L.	2017
Visual Cues Contribute Differentially to Audiovisual Perception of Consonants and Vowels in Improving Recognition and Reducing Cognitive Demands in Listeners With Hearing Impairment Using Hearing Aids	Moradi, S.; Lidestam, B.; Danielsson, H.; Ng, E. H. N.; Ronnberg, J.	2017
Auditory Processing of Older Adults With Probable Mild Cognitive Impairment	Edwards, J. D.; Lister, J. J.; Elias, M. N.; Tetlow, A. M.; Sardina, A. L.; Sadeq, N. A.; Brandino, A. D.; Bush, A. L. H.	2017
Speech Understanding in Complex Listening Environments by Listeners Fit With Cochlear Implants	Dorman, M. F.; Gifford, R. H.	2017
Continued search for better prediction of aided speech understanding in multi-talker environments	Xia, J.; Kalluri, S.; Micheyl, C.; Hafter, E.	2017
Cognitive factors as predictors of accented speech perception for younger and older adults	Ingvalson, E. M.; Lansford, K. L.; Fedorova, V.; Fernandez, G.	2017
Adaptation to novel foreign-accented speech and retention of benefit following training: Influence of aging and hearing loss	Bieber, R. E.; Gordon-Salant, S.	2017
Recognition of asynchronous auditory-visual speech by younger and older listeners: A preliminary study	Gordon-Salant, S.; Yeni-Komshian, G. H.; Fitzgibbons, P. J.; Willison, H. M.; Freund, M. S.	2017
Use of a glimpsing model to understand the performance of listeners with and without hearing loss in spatialized speech mixtures	Best, V.; Mason, C. R.; Swaminathan, J.; Roverud, E.; Kidd, G.	2017
Simultaneous and forward masking of vowels and stop consonants: Effects of age, hearing loss, and spectral shaping	Fogerty, D.; Bologna, W. J.; Ahlstrom, J. B.; Dubno, J. R.	2017

Syllable-constituent perception by hearing-aid users: Common factors in quiet and noise	Miller, J. D.; Watson, C. S.; Leek, M. R.; Dubno, J. R.; Wark, D. J.; Souza, P. E.; Gordon-Salante, S.; Ahlstrom, J. B.	2017
The effect of nearby maskers on speech intelligibility in reverberant, multi-talker environments	Westermann, A.; Buchholz, J. M.	2017
Characterizing the binaural contribution to speech-in-noise reception in elderly hearing-impaired listeners	Neher, T.	2017
Working Memory, Sleep, and Hearing Problems in Patients with Tinnitus and Hearing Loss Fitted with Hearing Aids	Zarenoe, R.; Hallgren, M.; Andersson, G.; Ledin, T.	2017
Validation of a Computer-Administered Version of the Digits-in-Noise Test for Hearing Screening in the United States	Folmer, R. L.; Vachhani, J.; McMillan, G. P.; Watson, C.; Kidd, G. R.; Feeney, M. P.	2017
Auditory and Cognitive Factors Associated with Speech-in-Noise Complaints following Mild Traumatic Brain Injury	Hoover, E. C.; Souza, P. E.; Gallun, F. J.	2017
Listening Effort Measured in Adults with Normal Hearing and Cochlear Implants	Perreau, A. E.; Wu, Y. H.; Tatge, B.; Irwin, D.; Corts, D.	2017
Evaluation of a Wind Noise Attenuation Algorithm on Subjective Annoyance and Speech-in-Wind Performance	Korhonen, P.; Kuk, F.; Seper, E.; Morkebjerg, M.; Roikjer, M.	2017
Outcomes of Hearing Aid Use by Individuals with Unilateral Sensorineural Hearing Loss (USNHL)	Bishop, C. E.; Hamadain, E.; Galster, J. A.; Johnson, M. F.; Spankovich, C.; Windmill, I.	2017
Is the Device-Oriented Subjective Outcome (DOSO) Independent of Personality?	Wu, Y. H.; Dumanch, K.; Stangl, E.; Miller, C.; Tremblay, K.; Bentler, R.	2017

Development and Pilot Evaluation of a Novel Theory-Based Intervention to Encourage Help-Seeking for Adult Hearing Loss	Saunders, G. H.; Frederick, M. T.; Silverman, S. C.; Nielsen, C.; Laplante-Levesque, A.	2017
The Effect of Signal-to-Noise Ratio on Linguistic Processing in a Semantic Judgment Task: An Aging Study	Stanley, N.; Davis, T.; Estis, J.	2017
The Effect of Conventional and Transparent Surgical Masks on Speech Understanding in Individuals with and without Hearing Loss	Atcherson, S. R.; Mendel, L. L.; Baltimore, W. J.; Patro, C.; Lee, S.; Pousson, M.; Spann, M. J.	2017
Binaural Interference and the Effects of Age and Hearing Loss	Mussoi, B. S.; Bentler, R. A.	2017
Interrupted Monosyllabic Words: The Effects of Ten Interruption Locations on Recognition Performance by Older Listeners with Sensorineural Hearing Loss	Wilson, R. H.; Sharrett, K. C.	2017
Personalized Object-Based Audio for Hearing Impaired TV Viewers	Shirley, B.; Meadows, M.; Malak, F.; Woodcock, J.; Tidball, A.	2017
Subjective hearing impairment after subarachnoid haemorrhage: Prevalence and risk factors	Vos, E. M.; Greebe, P.; Visser-Meily, J. M. A.; Rinkel, G. J. E.; Vergouwen, M. D. I.	2017
Chronic Conditions, New Onset, and Persistent Disability in the Ibadan Study of Aging	Ojagbemi, A.; Bello, T.; Luo, Z. H.; Gureje, O.	2017
Evaluation of a revised indication for determining adult cochlear implant candidacy	Sladen, D. P.; Gifford, R. H.; Haynes, D.; Kelsall, D.; Benson, A.; Lewis, K.; Zwolan, T.; Fu, Q. J.; Gantz, B.; Gilden, J.; Westerberg, B.; Gustin, C.; O'Neil, L.; Driscoll, C. L.	2017
Can routine office-based audiometry predict cochlear implant evaluation results?	Gubbels, S. P.; Gartrell, B. C.; Ploch, J. L.; Hanson, K. D.	2017

Insertion depth impacts speech perception and hearing preservation for lateral wall electrodes	O'Connell, B. P.; Hunter, J. B.; Haynes, D. S.; Holder, J. T.; Dedmon, M. M.; Noble, J. H.; Dawant, B. M.; Wanna, G. B.	2017
Risk of progressive hearing loss in untreated superior semicircular canal dehiscence	Patel, N. S.; Hunter, J. B.; O'Connell, B. P.; Bertrand, N. M.; Wanna, G. B.; Carlson, M. L.	2017
Stable benefits of bilateral over unilateral cochlear implantation after two years: A randomized controlled trial	van Zon, A.; Smulders, Y. E.; Stegeman, I.; Ramakers, G. G. J.; Kraaijenga, V. J. C.; Koenraads, S. P. C.; Zanten, G. A. V.; Rinia, A. B.; Stokroos, R. J.; Free, R. H.; Frijns, J. H. M.; Huinck, W. J.; Mylanus, E. A. M.; Tange, R. A.; Smit, A. L.; Thomeer, H. G. X. M.; Topsakal, V.; Grolman, W.	2017
Benefits of active middle ear implants in mixed hearing loss: Stapes versus round window	Lee, J. M.; Jung, J.; Moon, I. S.; Kim, S. H.; Choi, J. Y.	2017
Verbal working memory and inhibition-concentration in adults with cochlear implants	Moberly, A. C.; Houston, D. M.; Harris, M. S.; Adunka, O. F.; Castellanos, I.	2017
Menopause and postmenopausal hormone therapy and risk of hearing loss	Curhan, S. G.; Eliassen, A. H.; Eavey, R. D.; Wang, M.; Lin, B. M.; Curhan, G. C.	2017

Vital Signs: Noise-Induced Hearing Loss Among Adults - United States 2011-2012	Carroll, Y. I.; Eichwald, J.; Scinicariello, F.; Hoffman, H. J.; Deitchman, S.; Radke, M. S.; Themann, C. L.; Breysse, P.	2017
Effects of Various Extents of High-Frequency Hearing Loss on Speech Recognition and Gap Detection at Low Frequencies in Patients with Sensorineural Hearing Loss	Li, B.; Guo, Y.; Yang, G.; Feng, Y.; Yin, S.	2017
Sensory-motor relationships in speech production in post-lingually deaf cochlear-implanted adults and normal-hearing seniors: Evidence from phonetic convergence and speech imitation	Scarbel, L.; Beautemps, D.; Schwartz, J. L.; Sato, M.	2017
Speech-in-noise perception in unilateral hearing loss: Relation to pure-tone thresholds and brainstem plasticity	Vannson, N.; James, C. J.; Fraysse, B.; Lescure, B.; Strelnikov, K.; Deguine, O.; Barone, P.; Marx, M.	2017
Ten-Year Follow-up on Tumor Growth and Hearing in Patients Observed With an Intracanalicular Vestibular Schwannoma	Kirchmann, M.; Karnov, K.; Hansen, S.; Dethloff, T.; Stangerup, S. E.; Caye-Thomasen, P.	2017
Cognitive Function Predicts Listening Effort Performance During Complex Tasks in Normally Aging Adults	Harvey, J.; von Hapsburg, D.; Seeman, S.	2017
Effect of speechreading in presbycusis: Do we have a third ear?	Reis, L. R.; Escada, P.	2017
Implementation of Image-Guided Cochlear Implant Programming at a Distant Site	McRackan, T. R.; Noble, J. H.; Wilkinson, E. P.; Mills, D.; Dietrich, M. S.; Dawant, B. M.; Gifford, R. H.; Labadie, R. F.	2017
Daily cochlear implant use in adults: An objective measure	Campelo, P.; Ribeiro, D.; Tinoco, C.; Caroca, C. P. C.; Estibeiro, H.; Paco, J.	2017

Speech perception gap in patients qualifying for cochlear implant	Franks, Z.; Reghunathan, S.; Jacob, A.	2017
Auditory processing impairment and cardiometabolic risk	Smith, E. J.; Bishop, C. E.; Hamadain, E.; Spankovich, C.; Schweinfurth, J. M.	2017
Effect of radiation and chemoradiation on hearing	Dell Aringa, A. H. B.; Isaac, M. L.; Arruda, G. V.; Dell Aringa, A. R.	2017
Acoustic stimulation in cases with less residual hearing	Moteki, H.; Nishio, S. Y.; Miyagawa, M.; Tsukada, K.; Noguchi, Y.; Usami, S. I.	2017
Quality of Life after Sequential Bilateral Cochlear Implantation: An Updated Comprehensive Cochlear Implant Questionnaire	Nahm, E. A.; Liberatos, P.; Shi, Q. H.; Lai, E.; Kim, A. H.	2017
Cognitive Abilities and Quality of Life After Cochlear Implantation in the Elderly	Sonnet, M. H.; Montaut-Verient, B.; Niemier, J. Y.; Hoen, M.; Ribeyre, L.; Parietti-Winkler, C.	2017
Isolated Second Implant Adaptation Period in Sequential Cochlear Implantation in Adults	Smilsky, K.; Dixon, P. R.; Smith, L.; Shipp, D.; Ng, A.; Millman, T.; Stewart, S.; Nedzelski, J. M.; Lin, V. Y.; Chen, J. M.	2017
Light-Driven Contact Hearing Aid for Broad-Spectrum Amplification: Safety and Effectiveness Pivotal Study	Gantz, B. J.; Perkins, R.; Murray, M.; Levy, S. C.; Puria, S.	2017
Patient-Reported Outcomes From the United States Clinical Trial for a Hybrid Cochlear Implant	Kelsall, D. C.; Arnold, R. J. G.; Lionnet, L.	2017
Comparison of Speech-in-Noise and Localization Benefits in Unilateral Hearing Loss Subjects Using Contralateral Routing of Signal Hearing Aids or Bone-Anchored Implants	Snapp, H. A.; Holt, F. D.; Liu, X.; Rajguru, S. M.	2017

The Relationship Between Environmental Sound Awareness and Speech Recognition Skills in Experienced Cochlear Implant Users	Harris, M. S.; Boyce, L.; Pisoni, D. B.; Shafiro, V.; Moberly, A. C.	2017
Impact of Cochlear Implantation on Cognitive Functions of Older Adults: Pilot Test Results	Jayakody, D. M. P.; Friedland, P. L.; Nel, E.; Martins, R. N.; Atlas, M. D.; Sohrabi, H. R.	2017
Head Shadow and Binaural Squelch for Unilaterally Deaf Cochlear Implantees	Bernstein, J. G. W.; Schuchman, G. I.; Rivera, A. L.	2017
Assessing Cochlear Implant Outcomes in Older Adults Using HERMES: A National Web-based Database	Chen, S. Y.; Grisel, J. J.; Lam, A.; Golub, J. S.	2017
Performance Plateau in Prelingually and Postlingually Deafened Adult Cochlear Implant Recipients	Cusumano, C.; Friedmann, D. R.; Fang, Y. X.; Wang, B. H.; Roland, J. T.; Waltzman, S. B.	2017
Paget's Disease of the Temporal Bone: A Single-Institution Contemporary Review of 27 Patients	Deep, N. L.; Besch-Stokes, J. G.; Lane, J. I.; Driscoll, C. L. W.; Carlson, M. L.	2017
Older Individuals Meeting Medicare Cochlear Implant Candidacy Criteria in Noise but Not in Quiet: Are These Patients Improved by Surgery?	Mudery, J. A.; Francis, R.; McCrary, H.; Jacob, A.	2017
Cochlear Implantation in Meniere's Disease With and Without Labyrinthectomy	Mukherjee, P.; Eykamp, K.; Brown, D.; Curthoys, I.; Flanagan, S.; Biggs, N.; McNeill, C.; Gibson, W.	2017
Multifactor Influences of Shared Decision-Making in Acoustic Neuroma Treatment	Nellis, J. C.; Sharon, J. D.; Pross, S. E.; Ishii, L. E.; Ishii, M.; Dey, J. K.; Francis, H. W.	2017
Treatment of Lateral Skull Base and Posterior Cranial Fossa Lesions Utilizing the Extended Middle Cranial Fossa Approach	Roche, J. P.; Goates, A. J.; Hasan, D. M.; Howard, M. A.; Menezes, A. H.; Hansen, M. R.; Gantz, B. J.	2017

Musical Rehabilitation in Adult Cochlear Implant Recipients With a Self-administered Software	Smith, L.; Bartel, L.; Joglekar, S.; Chen, J.	2017
Effectiveness in Rehabilitation of Current Wireless CROS Technology in Experienced Bone-Anchored Implant Users	Snapp, H. A.; Hoffer, M. E.; Liu, X.; Rajguru, S. M.	2017
Cochlear Implantation in Otosclerosis: Surgical and Auditory Outcomes With a Brief on Facial Nerve Stimulation	Vashishth, A.; Fulcheri, A.; Rossi, G.; Prasad, S. C.; Caruso, A.; Sanna, M.	2017
The Impact of Comorbidities in the Aging Population on Cochlear Implant Outcomes	Wilkerson, B. J.; Porps, S. F.; Babu, S. C.	2017
Audiological Results in SSD With an Active Transcutaneous Bone Conduction Implant at a Retrosigmoidal Position	Salcher, R.; Zimmermann, D.; Giere, T.; Lenarz, T.; Maier, H.	2017
Round Window Application of an Active Middle Ear Implant: A Comparison With Hearing Aid Usage in Japan	Iwasaki, S.; Usami, S. I.; Takahashi, H.; Kanda, Y.; Tono, T.; Doi, K.; Kumakawa, K.; Gyo, K.; Naito, Y.; Kanzaki, S.; Yamanaka, N.; Kaga, K.	2017
The Effect of Citalopram Versus a Placebo on Central Auditory Processing in the Elderly	Polanski, J. F.; Soares, A. D.; Pereira, L. D.; Laercio de Mendonca Cruz, O.	2017
Single-Sided Deafness: Impact of Cochlear Implantation on Speech Perception in Complex Noise and on Auditory Localization Accuracy	Doge, J.; Baumann, U.; Weissgerber, T.; Rader, T.	2017
Usefulness of Electrical Auditory Brainstem Responses to Assess the Functionality of the Cochlear Nerve Using an Intracochlear Test Electrode	Lassaletta, L.; Polak, M.; Huesers, J.; Diaz-Gomez, M.; Calvino, M.; Varela-Nieto, I.; Gavilan, J.	2017
Evaluation of Maximal Speech Intelligibility With Vibrant Soundbridge in Patients With Sensorineural Hearing Loss	Lee, H. J.; Lee, J. M.; Choi, J. Y.; Jung, J.	2017

Cochlear Implantation in Postlingual Adult Patients With Long-Term Auditory Deprivation	Medina, M. D. M.; Polo, R.; Gutierrez, A.; Muriel, A.; Vaca, M.; Perez, C.; Cordero, A.; Cobeta, I.	2017
Influence of Floating-Mass Transducer Coupling Efficiency for Active Middle-Ear Implants on Speech Recognition	Muller, A.; Mir-Salim, P.; Zellhuber, N.; Helbig, R.; Bloching, M.; Schmidt, T.; Koscielny, S.; Dziemba, O. C.; Plontke, S. K.; Rahne, T.	2017
Stable Longitudinal Performance of Adult Cochlear Implant Users for More Than 10 Years	Rak, K.; Schraven, S. P.; Schendzielorz, P.; Kurz, A.; Shehata-Dieler, W.; Hagen, R.; Radeloff, A.	2017
Cochlear Implantation in the Elderly: Does Age Matter?	Rohloff, K.; Koopmann, M.; Wei, D.; Rudack, C.; Savvas, E.	2017
Effectiveness of Directional Microphones in Bilateral/Bimodal Cochlear Implant Users-Impact of Spatial and Temporal Noise Characteristics	Weissgerber, T.; Rader, T.; Baumann, U.	2017
Evaluation of the Ototoxicity Potential of Once-Daily, Single-Entity Hydrocodone in Patients with Chronic Pain: Results of Two Phase-3 Clinical Studies	Campbell, K.; Kutz, J. W.; Shoup, A.; Wen, W.; Lynch, S. Y.; He, E. L.; Ripa, S. R.	2017
Discrepancy between self-assessed hearing status and measured audiometric evaluation	Kim, S. Y.; Kim, H. J.; Kim, M. S.; Park, B.; Kim, J. H.; Choi, H. G.	2017
Investigation of the effect of cochlear implant electrode length on speech comprehension in quiet and noise compared with the results with users of electro-acoustic-stimulation, a retrospective analysis	Buchner, A.; Illg, A.; Majdani, O.; Lenarz, T.	2017
Adaptive benefit of cross-modal plasticity following cochlear implantation in deaf adults	Anderson, C. A.; Wiggins, I. M.; Kitterick, P. T.; Hartley, D. E. H.	2017

Cross-sectional evaluation of an internet-based hearing screening test in an occupational setting	Rashid, M. S.; Leensen, M. C.; De Laat, J. A.; Dreschler, W. A.	2017
Increased cross-modal functional connectivity in cochlear implant users	Chen, L. C.; Puschmann, S.; Debener, S.	2017
Effects of Recreational Noise on Threshold and Suprathreshold Measures of Auditory Function	Fulbright, A. N. C.; Le Prell, C. G.; Griffiths, S. K.; Lobarinas, E.	2017
Evaluation of Hearing Handicap in Adults with Auditory Neuropathy Spectrum Disorder	Prabhu, P.	2017
Speech Perception in Quiet and in Noise Condition in Individuals with Auditory Neuropathy Spectrum Disorder	Apeksha, K.; Kumar, A. U.	2017
Evaluation of the Relationship Between the Air-Bone Gap and Prolonged ABR Latencies in Mixed-Type Hearing Loss	Birkent, O. F.; Karlidag, T.; Basar, F.; Yalcin, S.; Kaygusuz, I.; Keles, E.; Akyigit, A.	2017
Efficacy of Directional Microphones in Hearing Aids Equipped with Wireless Synchronization Technology	Geetha, C.; Tanniru, K.; Rajan, R. R.	2017
Self-Reported Usage, Functional Benefit, and Audiologic Characteristics of Cochlear Implant Patients Who Use a Contralateral Hearing Aid	Neuman, A. C.; Waltzman, S. B.; Shapiro, W. H.; Neukam, J. D.; Zeman, A. M.; Svirsky, M. A.	2017
Predictors of Hearing-Aid Outcomes	Lopez-Poveda, E. A.; Johannesen, P. T.; Perez-Gonzalez, P.; Blanco, J. L.; Kalluri, S.; Edwards, B.	2017
The Benefits of Bimodal Aiding on Extended Dimensions of Speech Perception: Intelligibility, Listening Effort, and Sound Quality	Devocht, E. M. J.; Janssen, A. M. L.; Chalupper, J.; Stokroos, R. J.; George, E. L. J.	2017
Predictors of Entering a Hearing Aid Evaluation Period: A Prospective Study in Older Hearing-Help Seekers	Pronk, M.; Deeg, D. J. H.; Versfeld, N. J.; Heymans, M. W.; Naylor, G.; Kramer, S. E.	2017

Lexical-Access Ability and Cognitive Predictors of Speech Recognition in Noise in Adult Cochlear Implant Users	Kaandorp, M. W.; Smits, C.; Merkus, P.; Festen, J. M.; Goverts, S. T.	2017
Targeting functional fitness, hearing and health-related quality of life in older adults with hearing loss: Walk, Talk 'n' Listen, study protocol for a pilot randomized controlled trial	Lambert, J.; Ghadry-Tavi, R.; Knuff, K.; Jutras, M.; Siever, J.; Mick, P.; Roque, C.; Jones, G.; Little, J.; Miller, H.; Van Bergen, C.; Kurtz, D.; Murphy, M. A.; Jones, C. A.	2017
Visual working memory span in adults with cochlear implants: Some preliminary findings	Moberly, A. C.; Pisoni, D. B.; Harris, M. S.	2017
Validation of the Korean Version of the Spatial Hearing Questionnaire for Assessing the Severity and Symmetry of Hearing Impairment	Kong, T. H.; Park, Y. A.; Bong, J. P.; Park, S. Y.	2017
Development and Evaluation of the Program for Auditory Training in the Correction of Central Auditory Processing Disorders	Kaplun, D. I.; Gnezdilov, D. V.; Efimenko, G. A.; Pochechuev, A. A.; Ogorodnikova, E. A.; Boboshko, M. Y.; Ieee,	2017
Influence of age on speech intelligibility in babble noise	Cerny, L.; Vokral, J.; Dlouha, O.	2018
Hearing loss before and after cisplatin-based chemotherapy in testicular cancer survivors: a longitudinal study	Haugnes, H. S.; Stenklev, N. C.; Brydoy, M.; Dahl, O.; Wilsgaard, T.; Laukli, E.; Fossa, S. D.	2018
BoneBridge implantation in patients with single-sided deafness resulting from vestibular schwannoma resection: objective and subjective benefit evaluations	Yang, J.; Wang, Z.; Huang, M.; Chai, Y.; Jia, H.; Wu, Y.; Dai, Y.; Li, Y.; Wu, H.	2018
Cochlear implantation outcome in straightforward cases: can we do more for adults who cannot understand speech using hearing aids alone?	Luntz, M.; Khuri, M.; Khayr, R.; Gedaon Khuri, W.; Yehudai, N.; Shpak, T.	2018

Ten years of active middle ear implantation for sensorineural hearing loss	Barbara, M.; Filippi, C.; Covelli, E.; Volpini, L.; Monini, S.	2018
Categorical perception of lexical tones in native Mandarin-speaking listeners with sensorineural hearing loss	Qi, B.; Liu, P.; Gu, X.; Dong, R.; Liu, B.	2018
Diagnostic role of cone beam computed tomography for the position of straight array	An, S. Y.; An, C. H.; Lee, K. Y.; Jang, J. H.; Choung, Y. H.; Lee, S. H.	2018
Bimodal benefit for cochlear implant listeners with different grades of hearing loss in the opposite ear	Hoppe, U.; Hocke, T.; Digeser, F.	2018
Relationship between pure-tone audiogram findings and speech perception among older Japanese persons	Maeda, Y.; Takao, S.; Sugaya, A.; Kataoka, Y.; Kariya, S.; Tanaka, S.; Nagayasu, R.; Nakagawa, A.; Nishizaki, K.	2018
Transcutaneous bone conductive implants in patients with conductive/mixed hearing loss: audiological outcomes in noise condition	Magliulo, G.; Iannella, G.; De Vincentiis, M.; Turchetta, R.; Portanova, G.; Angeletti, D.; Mancini, P.	2018
Hearing preservation and clinical outcome of 32 consecutive electric acoustic stimulation (EAS) surgeries	Usami, S. I.; Moteki, H.; Tsukada, K.; Miyagawa, M.; Nishio, S. Y.; Takumi, Y.; Iwasaki, S.; Kumakawa, K.; Naito, Y.; Takahashi, H.; Kanda, Y.; Tono, T.	2018
Transtympanic Hearing Aid: exploratory study on a new device	Berrettini, S.; Bruschini, L.; D. E. Vito A; Gnocco, T.; Rosica, N. C.; Pizzoli, L.; Forli, F.	2018

Cochlear implantation in far-advanced otosclerosis: hearing results and complications	Dumas, A. R.; Schwalje, A. T.; Franco-Vidal, V.; Bebear, J. P.; Darrouzet, V.; Bonnard, D.	2018
Auditory temporal processing, reading, and phonological awareness among aging adults	Ronen, M.; Lifshitz-Ben-Basat, A.; Taitelbaum-Swead, R.; Fostick, L.	2018
The development of a new tool for the evaluation of handicap in elderly: the Geriatric Handicap Scale (GHS)	Verrusio, W.; Renzi, A.; Spallacci, G.; Pecci, M. T.; Pappada, M. A.; Cacciafesta, M.	2018
Development of an Interprofessional Resource on the Effects of Hearing Loss on Cognitive Assessment	Vitkus, L. M.; Marrone, N. L.	2018
Retained capacity for perceptual learning of degraded speech in primary progressive aphasia and Alzheimer's disease	Hardy, C. J. D.; Marshall, C. R.; Bond, R. L.; Russell, L. L.; Dick, K.; Ariti, C.; Thomas, D. L.; Ross, S. J.; Agustus, J. L.; Crutch, S. J.; Rohrer, J. D.; Bamiou, D. E.; Warren, J. D.	2018
Empowering Senior Cochlear Implant Users at Home via a Tablet Computer Application	Philips, B.; Smits, C.; Govaerts, P. J.; Doorn, I.; Vanpoucke, F.	2018
User-Innovated eHealth Solutions for Service Delivery to Older Persons With Hearing Impairment	Nielsen, A. C.; Rotger-Griful, S.; Kanstrup, A. M.; Laplante-Levesque, A.	2018
An Application of the Medical Research Council's Guidelines for Evaluating Complex Interventions: A Usability Study Assessing Smartphone-Connected Listening Devices in Adults With Hearing Loss	Maidment, D. W.; Ferguson, M.	2018
A Comparison of Personal Sound Amplification Products and Hearing Aids in Ecologically Relevant Test Environments	Brody, L.; Wu, Y. H.; Stangl, E.	2018
The Effects of Varying Directional Bandwidth in Hearing Aid Users' Preference and Speech-in-Noise Performance	Goyette, A.; Crukley, J.; Galster, J.	2018

The Acoustic Environments in Which Older Adults Wear Their Hearing Aids: Insights From Datalogging Sound Environment Classification	Humes, L. E.; Rogers, S. E.; Main, A. K.; Kinney, D. L.	2018
Binaural Speech Understanding With Bilateral Cochlear Implants in Reverberation	Kokkinakis, K.	2018
Isolated Internal Auditory Canal Diverticula: A Normal Anatomic Variant Not Associated with Sensorineural Hearing Loss	Mihal, D. C.; Feng, Y.; Kodet, M. L.; Lohse, C. M.; Carlson, M. L.; Lane, J. I.	2018
Morphology and cochlear implantation in enlarged vestibular aqueduct	Patel, N. D.; Ascha, M. S.; Manzoor, N. F.; Gupta, A.; Semaan, M.; Megerian, C.; Otteson, T. D.	2018
The effect of intratympanic steroid injection frequency in idiopathic sudden sensorineural hearing loss	Sugihara, E. M.; Evans, M. A.; Neumann, M.; Babu, S. C.	2018
The Role of Hyperbaric Oxygen as Salvage Therapy for Sudden Sensorineural Hearing Loss	Almosnino, G.; Holm, J. R.; Schwartz, S. R.; Zeitler, D. M.	2018
The Role of Hyperbaric Oxygen as Salvage Therapy for Sudden Sensorineural Hearing Loss	Almosnino, G.; Holm, J. R.; Schwartz, S. R.; Zeitler, D. M.	2018
Sensorineural hearing loss in takayasu's arteritis	Kimyon, U.; Esatoglu, S. N.; Kara, E.; Atas, A.; Gunay, E. E.; Karaman, E.; Gozen, E. D.; Hamuryudan, V.; Yazici, H.; Seyahi, E.	2018
The role of social frailty in explaining the association between hearing problems and mild cognitive impairment in older adults	Bae, S.; Lee, S.; Jung, S.; Makino, K.; Park, H.; Shimada, H.	2018

Benefits of incorporating the adaptive dynamic range optimization amplification scheme into an assistive listening device for people with mild or moderate hearing loss	Chang, H. Y.; Luo, C. H.; Lo, T. S.; Chen, H. C.; Huang, K. Y.; Liao, W. H.; Su, M. C.; Liu, S. Y.; Wang, N. M.	2018
Stapes Surgery for Otosclerosis in Patients Presenting with Mixed Hearing Loss	Salmon, C.; Barriat, S.; Lefebvre, P. P.	2018
A Role for Acoustic Stimulation in Advanced Otosclerosis: Direct Acoustic Cochlear Implant versus Cochlear Implant	Verhaert, N.; Borgers, C.; De Voecht, K.; Boon, E.; Desloovere, C.	2018
Binaural Perception in Single-Sided Deaf Cochlear Implant Users with Unrestricted or Restricted Acoustic Hearing in the Non-Implanted Ear	Dorbeau, C.; Galvin, J.; Fu, Q. J.; Legris, E.; Marx, M.; Bakhos, D.	2018
Late Cochlear Implantation in Early-Deafened Adults: A Detailed Analysis of Auditory and Self-Perceived Benefits	Debruyne, J.; Janssen, M.; Brokx, J.	2018
Effect of Cochlear Implantation on Quality of Life in Adults with Unilateral Hearing Loss	Dillon, M. T.; Buss, E.; Rooth, M. A.; King, E. R.; Deres, E. J.; Buchman, C. A.; Pillsbury, H. C.; Brown, K. D.	2018
Comparison of two different treatment protocols using systemic and intratympanic steroids with and without hyperbaric oxygen therapy in patients with severe to profound idiopathic sudden sensorineural hearing loss: A randomized controlled trial	Cho, I.; Lee, H. M.; Choi, S. W.; Kong, S. K.; Lee, I. W.; Goh, E. K.; Oh, S. J.	2018
Cortical Processing of Frequency Changes Reflected by the Acoustic Change Complex in Adult Cochlear Implant Users	Liang, C.; Houston, L. M.; Samy, R. N.; Abedelrehim, L. M. I.; Zhang, F.	2018
One-Year Results for Patients with Unilateral Hearing Loss and Accompanying Severe Tinnitus and Hyperacusis Treated with a Cochlear Implant	Ramos Macias, A.; Falcon-Gonzalez, J. C.; Manrique Rodriguez, M.; Morera Perez, C.; Garcia-Ibanez, L.; Cenjor Espanol, C.; Coudert-Koall, C.; Killian, M.	2018

Hearing Preservation with the Slim Modiolar Electrode Nucleus CI532 Cochlear Implant: A Preliminary Experience	Ramos-Macias, A.; Borkoski-Barreiro, S. A.; Falcon-Gonzalez, J. C.; Ramos-De Miguel, A.	2018
The Effect of Binaural Beamforming Technology on Speech Intelligibility in Bimodal Cochlear Implant Recipients	Vroegop, J. L.; Homans, N. C.; Goedegebure, A.; Dingemanse, J. G.; Van Immerzeel, T.; Van Der Schroeff, M. P.	2018
Language-specific strategy for programming hearing aids - A double-blind randomized controlled crossover study	Matsumoto, N.; Suzuki, N.; Iwasaki, S.; Ishikawa, K.; Tsukiji, H.; Higashino, Y.; Tabuki, T.; Nakagawa, T.	2018
Our auditory results using the Vibrant Soundbridge on the long process of the incus: 20 years of data	Gregoire, A.; Van Damme, J. P.; Gilain, C.; Bihin, B.; Garin, P.	2018
Intratympanic steroid delivery by an indwelling catheter in refractory severe sudden sensorineural hearing loss	Zanetti, D.; Di Berardino, F.; Nassif, N.; Redaelli De Zinis, L. O.	2018
Audiometric findings in elderly of 80 years and older and the potential link between audiometric parameters and cognition	De Raedemaeker, K.; Foulon, I.; Gordts, F.	2018
Stapes surgery for otosclerosis in patients presenting with mixed hearing loss	Thys, D.; Salmon, C.; Barriat, S.; Lefebvre, P. P.	2018
Qualitative, multimethod study of behavioural and attitudinal responses to cochlear implantation from the patient and healthcare professional perspective in Australia and the UK: Study protocol	Rapport, F.; Bierbaum, M.; McMahon, C.; Boisvert, I.; Lau, A.; Braithwaite, J.; Hughes, S.	2018
Feasibility of personalised remote long-term follow-up of people with cochlear implants: a randomised controlled trial	Cullington, H.; Kitterick, P.; Weal, M.; Margol-Gromada, M.	2018

Quality of life and cochlear implant: results in adults with postlingual hearing loss	de Sousa, A. F.; Couto, M. I. V.; Martinho-Carvalho, A. C.	2018
Intratympanic steroid injection and hyperbaric oxygen therapy for the treatment of refractory sudden hearing loss	Gulustan, F.; Yazici, Z. M.; Alakhras, W. M. E.; Erdur, O.; Acipayam, H.; Kufeciler, L.; Kayhan, F. T.	2018
Cross-cultural adaptation of the Amsterdam inventory for auditory disability and handicap to Brazilian Portuguese	Zanchetta, S.; Simoes, H. O.; Lunardelo, P. P.; Canavezi, M. D. O.; Reis, A. C. M. B.; Massuda, E. T.	2018
Relative contributions of auditory and cognitive functions on speech recognition in quiet and in noise among older adults	Mukari, S. Z. M. S.; Yusof, Y.; Ishak, W. S.; Maamor, N.; Chellapan, K.; Dzulkifli, M. A.	2018
Associations between sensory loss and social networks, participation, support, and loneliness Analysis of the Canadian Longitudinal Study on Aging	Mick, P.; Parfyonov, M.; Wittich, W.; Phillips, N.; Pichora-Fuller, M. K.	2018
Internet Usage and Loneliness in Older Hearing Aid Wearers	Simpson, A.; Clarke, S.; Sarkic, B.; Smullen, J. B.; Pereira, C. J.	2018
Association between patient-reported hearing and visual impairments and functional, psychological, and cognitive status among older adults with cancer	Soto-Perez-de-Celis, E.; Sun, C. L.; Tew, W. P.; Mohile, S. G.; Gajra, A.; Klepin, H. D.; Owusu, C.; Gross, C. P.; Muss, H. B.; Lichtman, S. M.; Chapman, A. E.; Cohen, H. J.; Dale, W.; Kim, H.; Fernandes, S.; Katheria, V.; Hurria, A.	2018

Saliency of Vowel Features in Neural Responses of Cochlear Implant Users	Prevost, F.; Lehmann, A.	2018
Saliency of Vowel Features in Neural Responses of Cochlear Implant Users	Prevost, F.; Lehmann, A.	2018
Categorization of sentence recognition for older adults under noisy and time-altered conditions	Kim, S.; Ma, S.; Lee, J.; Han, W.	2018
Can cochlear implantation improve neurocognition in the aging population?	Volter, C.; Gotze, L.; Dazert, S.; Falkenstein, M.; Thomas, J. P.	2018
Neural and behavioral changes after the use of hearing aids	Karawani, H.; Jenkins, K. A.; Anderson, S.	2018
The critical warning sign of real-time brainstem auditory evoked potentials during microvascular decompression for hemifacial spasm	Park, S. K.; Joo, B. E.; Lee, S.; Lee, J. A.; Hwang, J. H.; Kong, D. S.; Seo, D. W.; Park, K.; Lee, H. T.	2018
Hearing aids in patients with vestibular schwannoma: Interest of the auditory brainstem responses	Reffet, K.; Lescanne, E.; Bobillier, C.; Aussedat, C.; Bakhos, D.	2018
On the evaluation of a superpower sound processor for bone-anchored hearing	Bosman, A. J.; Kruyt, I. J.; Mylanus, E. A. M.; Hol, M. K. S.; Snik, A. F. M.	2018
Evaluation of an abutment-level superpower sound processor for bone-anchored hearing	Bosman, A. J.; Kruyt, I. J.; Mylanus, E. A. M.; Hol, M. K. S.; Snik, A. F. M.	2018
Psychosocial health of cochlear implant users compared to that of adults with and without hearing aids: Results of a nationwide cohort study	Bosdriesz, J. R.; Stam, M.; Smits, C.; Kramer, S. E.	2018
The Otology Questionnaire Amsterdam: a generic patient reported outcome measure about the severity and impact of ear complaints. A cross-sectional study on the development of this questionnaire	Bruinewoud, E. M.; Kraak, J. T.; van Leeuwen, L. M.; Kramer, S. E.; Merkus, P.	2018

Predicting sequential bilateral cochlear implantation performance in postlingually deafened adults; A retrospective cohort study	Smulders, Y. E.; Hendriks, T.; Stegeman, I.; Eikelboom, R. H.; Sucher, C.; Upson, G.; Chester Browne, R.; Jayakody, D.; Santa Maria, P. L.; Atlas, M. D.; Friedland, P. L.	2018
Early hearing loss detection in rheumatoid arthritis and primary Sjogren syndrome using extended high frequency audiometry	Galarza-Delgado, D. A.; Villegas Gonzalez, M. J.; Riega Torres, J.; Soto-Galindo, G. A.; Mendoza Flores, L.; Trevino Gonzalez, J. L.	2018
Outcome evaluation on cochlear implant users with residual hearing	Neben, N.; Buechner, A.; Schuessler, M.; Lenarz, T.	2018
Investigating Speech Recognition and listening effort with different device configurations in adult cochlear implant users	Sladen, D. P.; Nie, Y.; Berg, K.	2018
Facial nerve stimulation after cochlear implantation: Our experience in 448 adult patients	Pires, J. S.; Melo, A. S.; Caiado, R.; Martins, J. H.; Eloi Moura, J.; Silva, L. F.	2018
Effects of cochlear implantation on adulthood	Vieira, S. D.; Dupas, G.; Chiari, B. M.	2018
Preservation of Hearing and Facial Nerve Function with the Microsurgical Excision of Large Vestibular Schwannomas: Experience with the Retrosigmoid Approach	Rajput, M. S. A.; Ahmad, A. N.; Arain, A. A.; Adeel, M.; Akram, S.; Awan, M. S.; Bari, M. E.	2018
Association between diabetes and changes in hearing: A cross-sectional study	Lebech Cichosz, S.; Hejlesen, O.	2018

Hearing impairment and type 1 diabetes in the diabetes Control and complications trial/ epidemiology of diabetes interventions and complications (DCCT/EDIC) cohort	Schade, D. S.; Lorenzi, G. M.; Braffett, B. H.; Gao, X.; Bainbridge, K. E.; Barnie, A.; Cruickshanks, K. J.; Dalton, D.; Diminick, L.; Gubitosi-Klug, R.; Kramer, J. R.; Lachin, J. M.; Larkin, M. E.; Cowie, C. C.	2018
The Participation Scale: psychometric properties of a South Indian translation with hearing-impaired respondents	Thammaiah, S.; Manchaiah, V.; Easwar, V.; Krishna, R.; McPherson, B.	2018
Speech Perception in Noise and Listening Effort of Older Adults With Nonlinear Frequency Compression Hearing Aids	Shehorn, J.; Marrone, N.; Muller, T.	2018
A Dynamically Focusing Cochlear Implant Strategy Can Improve Vowel Identification in Noise	Arenberg, J. G.; Parkinson, W. S.; Litvak, L.; Chen, C.; Kreft, H. A.; Oxenham, A. J.	2018
Temporal Fine Structure Processing, Pitch, and Speech Perception in Adult Cochlear Implant Recipients	Dincer D'Alessandro, H.; Ballantyne, D.; Boyle, P. J.; De Seta, E.; DeVincentiis, M.; Mancini, P.	2018
Hearing Difficulty Is Associated With Injuries Requiring Medical Care	Mick, P.; Foley, D.; Lin, F.; Pichora-Fuller, M. K.	2018
Hearing Handicap and Speech Recognition Correlate With Self-Reported Listening Effort and Fatigue	Alhanbali, S.; Dawes, P.; Lloyd, S.; Munro, K. J.	2018
Social Connectedness and Perceived Listening Effort in Adult Cochlear Implant Users: A Grounded Theory to Establish Content Validity for a New Patient-Reported Outcome Measure	Hughes, S. E.; Hutchings, H. A.; Rapport, F. L.; McMahon, C. M.; Boisvert, I.	2018

Results in Adult Cochlear Implant Recipients With Varied Asymmetric Hearing: A Prospective Longitudinal Study of Speech Recognition, Localization, and Participant Report	Firszt, J. B.; Reeder, R. M.; Holden, L. K.; Dwyer, N. Y.	2018
Development of a Test Battery for Evaluating Speech Perception in Complex Listening Environments: Effects of Sensorineural Hearing Loss	Phatak, S. A.; Sheffield, B. M.; Brungart, D. S.; Grant, K. W.	2018
Characteristics of Real-World Signal to Noise Ratios and Speech Listening Situations of Older Adults With Mild to Moderate Hearing Loss	Wu, Y. H.; Stangl, E.; Chipara, O.; Hasan, S. S.; Welhaven, A.; Oleson, J.	2018
Linguistic Context Versus Semantic Competition in Word Recognition by Younger and Older Adults With Cochlear Implants	Amichetti, N. M.; Atagi, E.; Kong, Y. Y.; Wingfield, A.	2018
Tinnitus and Auditory Perception After a History of Noise Exposure: Relationship to Auditory Brainstem Response Measures	Bramhall, N. F.; Konrad-Martin, D.; McMillan, G. P.	2018
Evaluating the Performance of a Visually Guided Hearing Aid Using a Dynamic Auditory-Visual Word Congruence Task	Roverud, E.; Best, V.; Mason, C. R.; Streeter, T.; Kidd, G.	2018
Auditory and Audiovisual Close Shadowing in Post-Lingually Deaf Cochlear-Implanted Patients and Normal-Hearing Elderly Adults	Scarbel, L.; Beautemps, D.; Schwartz, J. L.; Sato, M.	2018
Assessing the Relationship Between the Electrically Evoked Compound Action Potential and Speech Recognition Abilities in Bilateral Cochlear Implant Recipients	Schvartz-Leyzac, K. C.; Pfingst, B. E.	2018
Relations Between Self-Reported Daily-Life Fatigue, Hearing Status, and Pupil Dilation During a Speech Perception in Noise Task	Wang, Y.; Naylor, G.; Kramer, S. E.; Zekveld, A. A.; Wendt, D.; Ohlenforst, B.; Lunner, T.	2018
Effects of Cerebral Blood Flow and Vessel Conditions on Speech Recognition in Patients With Postlingual Adult Cochlear Implant: Predictable Factors for the Efficacy of Cochlear Implant	Ishino, T.; Ragaee, M. A.; Maruhashi, T.; Kajikawa, M.; Higashi, Y.; Sonoyama, T.; Takeno, S.; Hirakawa, K.	2018

Validating a Method to Assess Lipreading, Audiovisual Gain, and Integration During Speech Reception With Cochlear-Implanted and Normal-Hearing Subjects Using a Talking Head	Schreitmuller, S.; Frenken, M.; Bentz, L.; Ortmann, M.; Walger, M.; Meister, H.	2018
Evidence-Based Inclusion Criteria for Cochlear Implantation in Patients With Postlingual Deafness	Snel-Bongers, J.; Netten, A. P.; Boermans, Ppbm; Rotteveel, L. J. C.; Briaire, J. J.; Frijns, J. H. M.	2018
Clinical Effectiveness of an At-Home Auditory Training Program: A Randomized Controlled Trial	Humes, L. E.; Skinner, K. G.; Kinney, D. L.; Rogers, S. E.; Main, A. K.; Quigley, T. M.	2018
Audiologic profile in patients with ankylosing spondylitis: A controlled study of 30 patients	Yagueshita, L.; Lucinda, L. R.; Azevedo, V.; Wiemes, G. R.; Wiemes, N. R.; Polanski, J. F.	2018
Effect of auditory temporal processing training on behavioral and electrophysiological functions in central presbycusis	Asal, S. I.; Sobhy, O. A.; Morsy, H. M.	2018
Evaluation of the impact of hearing loss in adults: Validation of a quality of life questionnaire	Ambert-Dahan, E.; Laouenan, C.; Lebredonchel, M.; Borel, S.; Carillo, C.; Bouccara, D.; Sterkers, O.; Ferrary, E.; Mosnier, I.	2018
Asymmetric hearing loss and the benefit of cochlear implantation regarding speech perception, tinnitus burden and psychological comorbidities: a prospective follow-up study	Ketterer, M. C.; Knopke, S.; Hausler, S. M.; Hildenbrand, T.; Becker, C.; Grabel, S.; Olze, H.	2018

Living with otosclerosis: disease-specific health-related quality-of-life measurement in patients undergoing stapes surgery	Lailach, S.; Schenke, T.; Baumann, I.; Walter, H.; Praetorius, M.; Beleites, T.; Zahnert, T.; Neudert, M.	2018
Cochlear implantation in the elderly: outcomes, long-term evolution, and predictive factors	Garcia-Iza, L.; Martinez, Z.; Ugarte, A.; Fernandez, M.; Altuna, X.	2018
The importance of early audiological assessment and aural rehabilitation in stroke patients	Staikoudi, S.	2018
Effects of Phase-Locking Deficits on Speech Recognition in Older Adults With Presbycusis	Hao, W. Y.; Wang, Q.; Li, L.; Qiao, Y. F.; Gao, Z. Q.; Ni, D. F.; Shang, Y. Y.	2018
Patient benefit following Bimodal CI-provision: Self-reported Abilities vs. hearing status	Wallhausser-Franke, E.; Balkenhol, T.; Hetjens, S.; Rotter, N.; Servais, J. J.	2018
Impaired cognitive functioning in cochlear implant recipients over the age of 55 years: A cross-sectional study using the Repeatable Battery for the Assessment of Neuropsychological Status for Hearing-impaired individuals (RBANS-H)	Claes, A. J.; Van de Heyning, P.; Gilles, A.; Hofkens-Van den Brandt, A.; Van Rompaey, V.; Mertens, G.	2018
Gray Matter Atrophy Is Associated With Cognitive Impairment in Patients With Presbycusis: A Comprehensive Morphometric Study	Ren, F. X.; Ma, W.; Li, M. W.; Sun, H. Q.; Xin, Q.; Zong, W.; Chen, W. B.; Wang, G. B.; Gao, F.; Zhao, B.	2018
Impaired cognitive functioning in cochlear implant recipients over the age of 55 years: A cross-sectional study using the Repeatable Battery for the Assessment of Neuropsychological Status for Hearing-impaired individuals (RBANS-H)	Claes, A. J.; Van de Heyning, P.; Gilles, A.; Hofkens-Van den Brandt, A.; Van Rompaey, V.; Mertens, G.	2018
On Dynamic Pitch Benefit for Speech Recognition in Speech Masker	Shen, J.; Souza, P. E.	2018

Exploring the Link Between Cognitive Abilities and Speech Recognition in the Elderly Under Different Listening Conditions	Nuesse, T.; Steenken, R.; Neher, T.; Holube, I.	2018
Age-Related Differences in the Effects of Masker Cuing on Releasing Chinese Speech From Informational Masking	Feng, T. Q.; Chen, Q. R.; Xiao, Z. D.	2018
Impaired speech perception in noise with a normal audiogram: No evidence for cochlear synaptopathy and no relation to lifetime noise exposure	Guest, H.; Munro, K. J.; Prendergast, G.; Millman, R. E.; Plack, C. J.	2018
A perspective on brain-behavior relationships and effects of age and hearing using speech-in-noise stimuli	Billings, C. J.; Madsen, B. M.	2018
Electric and acoustic harmonic integration predicts speech-in-noise performance in hybrid cochlear implant users	Bonnard, D.; Schwalje, A.; Gantz, B.; Choi, I.	2018
Effect of age and hearing loss on auditory stream segregation of speech sounds	David, M.; Tausend, A. N.; Strelcyk, O.; Oxenham, A. J.	2018
Differential effects of hearing impairment and age on electrophysiological and behavioral measures of speech in noise	Koerner, T. K.; Zhang, Y.	2018
Auditory cortex responses to interaural time differences in the envelope of low-frequency sound, recorded with MEG in young and older listeners	Ross, B.	2018
The effect of reward on listening effort as reflected by the pupil dilation response	Koelewijn, T.; Zekveld, A. A.; Lunner, T.; Kramer, S. E.	2018
Speech perception in tinnitus is related to individual distress level - A neurophysiological study	Jagoda, L.; Giroud, N.; Neff, P.; Kegel, A.; Kleinjung, T.; Meyer, M.	2018
Impact of SNR, masker type and noise reduction processing on sentence recognition performance and listening effort as indicated by the pupil dilation response	Ohlenforst, B.; Wendt, D.; Kramer, S. E.; Naylor, G.; Zekveld, A. A.; Lunner, T.	2018
Restoration of spatial hearing in adult cochlear implant users with single-sided deafness	Litovsky, R. Y.; Moua, K.; Godar, S.; Kan, A.; Misurelli, S. M.; Lee, D. J.	2018

Neural envelope encoding predicts speech perception performance for normal-hearing and hearing-impaired adults	Goossens, T.; Vercammen, C.; Wouters, J.; van Wieringen, A.	2018
Speech audiometry and data logging in CI patients : Implications for adequate test levels	Hey, M.; Hocke, T.; Ambrosch, P.	2018
Perceptual and Model-Based Evaluation of Ideal Time-Frequency Noise Reduction in Hearing-Impaired Listeners	Koning, R.; Bruce, I. C.; Denys, S.; Wouters, J.	2018
The relative contribution of visual cues and acoustic enhancement strategies in improving speech perception of individuals with auditory neuropathy spectrum disorders	Balan, J. R.; Maruthy, S.	2018
Awareness of Hearing Loss in Older Adults: Results of a Survey Conducted in 500 Subjects Across 5 European Countries as a Basis for an Online Awareness Campaign	D'Haese, P. S. C.; De Bodt, M.; Van Rompaey, V.; Van de Heyning, P.	2018
P3 cognitive potential in cochlear implant users	Grasel, S.; Greters, M.; Goffi-Gomez, M. V. S.; Bittar, R.; Weber, R.; Oiticica, J.; Bento, R. F.	2018
Hearing loss in the elderly: Is the hearing handicap inventory for the elderly - Screening version effective in diagnosis when compared to the audiometric test?	Servidoni, A. B.; De Oliveira Conterno, L.	2018
P3 Cognitive Potential in Cochlear Implant Users	Grasel, S.; Greters, M.; Goffi-Gomez, M. V. S.; Bittar, R.; Weber, R.; Oiticica, J.; Bento, R. F.	2018
The relationship between speech recognition, behavioural listening effort, and subjective ratings	Picou, E. M.; Ricketts, T. A.	2018
Semantic context improves speech intelligibility and reduces listening effort for listeners with hearing impairment	Holmes, E.; Folkeard, P.; Johnsrude, I. S.; Scollie, S.	2018
Targeting the psychosocial and functional fitness challenges of older adults with hearing loss: a participatory approach to adaptation of the walk and talk for your life program	Jutras, M.; Lambert, J.; Hwang, J.; Wang, L.; Simon, S.; Del Medico, T.; Mick, P.; Miller, H.; Kurtz, D.; Murphy, M. A.; Jones, C. A.	2018

Comparison of single-microphone noise reduction schemes: can hearing impaired listeners tell the difference?	Huber, R.; Bisitz, T.; Gerkmann, T.; Kiessling, J.; Meister, H.; Kollmeier, B.	2018
Development and psychometric properties of the sound preference and hearing habits questionnaire (SP-HHQ)	Meis, M.; Huber, R.; Fischer, R. L.; Schulte, M.; Spilski, J.; Meister, H.	2018
The effect of tinnitus specific intracochlear stimulation on speech perception in patients with unilateral or asymmetric hearing loss accompanied with tinnitus and the effect of formal auditory training	Arts, Ragj; George, E. L. J.; Janssen, Maml; Griessner, A.; Zierhofer, C.; Stokroos, R. J.	2018
Tolerable delay for speech production and perception: effects of hearing ability and experience with hearing aids	Goehring, T.; Chapman, J. L.; Bleeck, S.; Monaghan, J. J. M.	2018
Development of the Social Participation Restrictions Questionnaire (SPaRQ) through consultation with adults with hearing loss, researchers, and clinicians: a content evaluation study	Heffernan, E.; Coulson, N. S.; Ferguson, M. A.	2018
The use of self-report measures to examine changes in perception in response to fittings using different signal processing parameters	Anderson, M.; Rallapalli, V.; Schoof, T.; Souza, P.; Arehart, K.	2018
Hearing loss and social support in urban and rural communities	Hay-McCutcheon, M. J.; Hyams, A.; Yang, X.; Parton, J.	2018
Evaluation of the NAL Dynamic Conversations Test in older listeners with hearing loss	Best, V.; Keidser, G.; Freeston, K.; Buchholz, J. M.	2018
Semantic context improves speech intelligibility and reduces listening effort for listeners with hearing impairment	Holmes, E.; Folkeard, P.; Johnsrude, I. S.; Scollie, S.	2018
Optimising the effect of noise reduction algorithm ClearVoice in cochlear implant users by increasing the maximum comfort levels	Dingemanse, J. G.; Goedegebure, A.	2018
Effects of a transient noise reduction algorithm on speech intelligibility in noise, noise tolerance and perceived annoyance in cochlear implant users	Dingemanse, J. G.; Vroegop, J. L.; Goedegebure, A.	2018
Evaluation of a multi-channel algorithm for reducing transient sounds	Keshavarzi, M.; Baer, T.; Moore, B. C. J.	2018

Cortical auditory evoked potentials in cochlear implant listeners via single electrode stimulation in relation to speech perception	Liebscher, T.; Alberter, K.; Hoppe, U.	2018
The performance of an automatic acoustic-based program classifier compared to hearing aid users' manual selection of listening programs	Searchfield, G. D.; Linford, T.; Kobayashi, K.; Crowhen, D.; Latzel, M.	2018
A directional remote-microphone for bimodal cochlear implant recipients	Vroegop, J. L.; Homans, N. C.; Goedegebure, A.; van der Schroeff, M. P.	2018
Assessing auditory nerve condition by tone decay in deaf subjects with a cochlear implant	Wasmann, J. W. A.; van Eijl, R. H. M.; Versnel, H.; van Zanten, G. A.	2018
Validation of self-reported hearing loss among adult Croatians: the performance of the Hearing Self-Assessment Questionnaire against audiometric evaluation	Bonetti, L.; Simunjak, B.; Franic, J.	2018
Hearing aid noise suppression and working memory function	Neher, T.; Wagener, K. C.; Fischer, R. L.	2018
The role of speech rate for Italian-speaking cochlear implant users: insights for everyday speech perception	D'Alessandro, H. D.; Boyle, P. J.; Ballantyne, D.; De Vincentiis, M.; Mancini, P.	2018
Assessment of speech recognition abilities in quiet and in noise: a comparison between self-administered home testing and testing in the clinic for adult cochlear implant users	de Graaff, F.; Huysmans, E.; Merkus, P.; Goverts, S. T.; Smits, C.	2018
Acoustic and perceptual effects of magnifying interaural difference cues in a simulated "binaural" hearing aid	de Taillez, T.; Grimm, G.; Kollmeier, B.; Neher, T.	2018
A method for determining precise electrical hearing thresholds in cochlear implant users	Rader, T.; Doms, P.; Adel, Y.; Weissgerber, T.; Strieth, S.; Baumann, U.	2018
Speech understanding in noise in elderly adults: the effect of inhibitory control and syntactic complexity	van Knijff, E. C.; Coene, M.; Govaerts, P. J.	2018

Electrical dynamic range is only weakly associated with auditory performance and speech recognition in long-term users of cochlear implants	Kim, S. Y.; Jeon, S. K.; Oh, S. H.; Lee, J. H.; Suh, M. W.; Lee, S. Y.; Lim, H. J.; Park, M. K.	2018
The effect of hearing impairment, hearing aid technology and task difficulty on listening effort as indicated by the pupillary response	Wendt, D.; Lunner, T.	2018
Hearing aid acclimatization in adults with hearing impairment	Mohanty, S. P.; Palanisamy, H. P.; Gundmi, A.; Ramesh, H.; Rajashekhar, B.	2018
Prevalence, characteristics, and treatment patterns of hearing difficulty in the United States	Mahboubi, H.; Lin, H. W.; Bhattacharyya, N.	2018
Association of cardiovascular comorbidities with hearing loss in the older old	Wattamwar, K.; Jason Qian, Z.; Otter, J.; Leskowitz, M. J.; Caruana, F. F.; Siedlecki, B.; Spitzer, J. B.; Lalwani, A. K.	2018
Right-Ear Advantage for Speech-in-Noise Recognition in Patients with Nonlateralized Tinnitus and Normal Hearing Sensitivity	Tai, Y.; Husain, F. T.	2018
Voice Discrimination by Adults with Cochlear Implants: the Benefits of Early Implantation for Vocal-Tract Length Perception	Zaltz, Y.; Goldsworthy, R. L.; Kishon-Rabin, L.; Eisenberg, L. S.	2018
Investigating the Impact of Hearing Aid Use and Auditory Training on Cognition, Depressive Symptoms, and Social Interaction in Adults With Hearing Loss: Protocol for a Crossover Trial	Nkyekyer, J.; Meyer, D.; Blamey, P. J.; Pipingas, A.; Bhar, S.	2018
Understanding Hearing Loss and Barriers to Hearing Health Care Among Korean American Older Adults: A Focus Group Study	Choi, J. S.; Shim, K. S.; Kim, K.; Nieman, C. L.; Mamo, S. K.; Lin, F. R.; Han, H. R.	2018

Impact of chemotherapy-induced neurotoxicities on adult cancer survivors' symptom burden and quality of life	Miaskowski, C.; Mastick, J.; Paul, S. M.; Abrams, G.; Cheung, S.; Sabes, J. H.; Kober, K. M.; Schumacher, M.; Conley, Y. P.; Topp, K.; Smoot, B.; Mausisa, G.; Mazor, M.; Wallhagen, M.; Levine, J. D.	2018
Assessment of the Self Perceived Hearing Handicap and its Associated Factors in Elderly People with Hearing Loss	Rajasekaran, V.; Gurulakshmi, R. B.	2018
2333 Impact of spoken sentence predictability on cognitive spare capacity in elderly adults with hearing loss	Hunter, C. R.; Pisoni, D. B.; Collins, D.; Humes, L. E.	2018
Association between frontal-executive dysfunction and speech-in-noise perception deficits in mild cognitive impairment	Lee, S. J.; Park, K. W.; Kim, L. S.; Kim, H.	2018
Communication skills of deaf and hard-of-hearing college students: Objective measures and self-assessment	Spencer, L. J.; Marschark, M.; Machmer, E.; Durkin, A.; Borgna, G.; Convertino, C.	2018
Performance variability on perceptual discrimination tasks in profoundly deaf adults with cochlear implants	Hay-McCutcheon, M. J.; Peterson, N. R.; Pisoni, D. B.; Kirk, K. I.; Yang, X.; Parton, J.	2018
Sounding out d/Deafness: the experiences of d/Deaf prisoners	Kelly, L. M.	2018
Evaluating the Usability and Acceptability of Communication Tools Among Older Adults	Nilsen, M. L.; Morrison, A.; Lingler, J. H.; Myers, B.; Johnson, J. T.; Happ, M. B.; Sereika, S. M.; DeVito Dabbs, A.	2018
Evaluating Auditory Pathway by Electrical Auditory Middle Latency Response and Postoperative Hearing Rehabilitation	Wang, B.; Cao, K.; Wei, C.; Gao, Z.; Li, H.	2018
Is the use of a bone conduction hearing device on a softband a useful tool in the pre-operative assessment of suitability for other hearing implants?	Spielmann, P. M.; Roplekar, R.; Rae, C.; Ahmed, F.; Jones, S. E. M.	2018

Effects of CyberKnife therapy for vestibular schwannoma on hearing: A retrospective study	Caklr, O.; Berkiten, G.; Tutar, B.; Yllmazer, A. B.; Kumral, T. L.; Salturk, Z.; Uyar, Y.	2018
Improvement in sudden sensorineural hearing loss with steroid therapy does not preclude the need for MRI to rule out vestibular schwannoma	Puccinelli, C.; Carlson, M. L.	2018
Sensorineural hearing loss in patients with vestibular schwannoma relies on the presence of utricular hydrops, as diagnosed with heavily weighted T2 sequences	Poillon, G.; Eliezer, M.; Gillibert, A.; Horion, J.; Gerardin, E.; Trintignac, A.; Magne, N.; Attye, A.	2018
Associations Between Perceived Stress and Chemotherapy-Induced Peripheral Neuropathy and Otoxicity in Adult Cancer Survivors	Miaskowski, C.; Paul, S. M.; Mastick, J.; Abrams, G.; Topp, K.; Smoot, B.; Kober, K. M.; Chesney, M.; Mazor, M.; Mausisa, G.; Schumacher, M.; Conley, Y. P.; Sabes, J. H.; Cheung, S.; Wallhagen, M.; Levine, J. D.	2018
Comparison of Subjective and Objective Measures of Hearing, Auditory Processing, and Cognition Among Older Adults With and Without Mild Cognitive Impairment	Fausto, B. A.; Badana, A. N. S.; Arnold, M. L.; Lister, J. J.; Edwards, J. D.	2018
A Potential Bias in Subjective Ratings of Mental Effort	Moore, T. M.; Picou, E. M.	2018
Perceived Voice Quality and Voice-Related Problems Among Older Adults With Hearing Impairments	Hengen, J.; Hammarstrom, I. L.; Stenfelt, S.	2018
Talker Differences in Clear and Conversational Speech: Perceived Sentence Clarity for Young Adults With Normal Hearing and Older Adults With Hearing Loss	Ferguson, S. H.; Morgan, S. D.	2018

The Impact of Age, Background Noise, Semantic Ambiguity, and Hearing Loss on Recognition Memory for Spoken Sentences	Koeritzer, M. A.; Rogers, C. S.; Van Engen, K. J.; Peelle, J. E.	2018
Spatial Release From Masking in Adults With Bilateral Cochlear Implants: Effects of Distracter Azimuth and Microphone Location	Davis, T. J.; Gifford, R. H.	2018
Speech Understanding in Noise for Adults With Cochlear Implants: Effects of Hearing Configuration, Source Location Certainty, and Head Movement	Gifford, R. H.; Loiselle, L.; Natale, S.; Sheffield, S. W.; Sunderhaus, L. W.; Dietrich, M. S.; Dorman, M. F.	2018
The Effects of Static and Moving Spectra Ripple Sensitivity on Unaided and Aided Speech Perception in Noise	Miller, C. W.; Bernstein, J. G. W.; Zhang, X. Y.; Wu, Y. H.; Bentler, R. A.; Tremblay, K.	2018
Gated Word Recognition by Postlingually Deafened Adults With Cochlear Implants: Influence of Semantic Context	Patro, C.; Mendel, L. L.	2018
Reading Behind the Lines: The Factors Affecting the Text Reception Threshold in Hearing Aid Users	Zekveld, A. A.; Pronk, M.; Danielsson, H.; Ronnberg, J.	2018
Explaining Discrepancies Between the Digit Triplet Speech-in-Noise Test Score and Self-Reported Hearing Problems in Older Adults	Pronk, M.; Deeg, D. J. H.; Kramer, S. E.	2018
Memory Span for Spoken Digits in Adults With Cochlear Implants or Typical Hearing: Effects of Age and Identification Ability	Cleary, M.; Wilkinson, T.; Wilson, L.; Goupell, M. J.	2018
The Effects of Static and Moving Spectral Ripple Sensitivity on Unaided and Aided Speech Perception in Noise	Miller, C. W.; Bernstein, J. G. W.; Zhang, X.; Wu, Y. H.; Bentler, R. A.; Tremblay, K.	2018
Low background noise increases cognitive load in older adults listening to competing speech	Meister, H.; Rahlmann, S.; Walger, M.	2018
A Randomized Controlled Trial to Evaluate Approaches to Auditory Rehabilitation for Blast-Exposed Veterans with Normal or Near-Normal Hearing Who Report Hearing Problems in Difficult Listening Situations	Saunders, G. H.; Frederick, M. T.; Arnold, M. L.; Silverman, S. C.; Chisolm, T. H.; Myers, P. J.	2018

Patterns of aided loudness growth in experienced adult listeners with early-onset severe-profound hearing loss	Gottermeier, L.; De Filippo, C.	2018
Investigation of extended bandwidth hearing aid amplification on speech intelligibility and sound quality in adults with mild-to-moderate hearing loss	Seeto, A.; Searchfield, G. D.	2018
Upgrade to nucleus 6 in previous generation cochleara sound processor recipients	Biever, A.; Gilden, J.; Zwolan, T.; Mears, M.; Beiter, A.	2018
Using a digital language processor to quantify the auditory environment and the effect of hearing AIDS for adults with hearing loss	Klein, K. E.; Wu, Y. H.; Stangl, E.; Bentler, R. A.	2018
The characteristics of adults with severe hearing loss	Souza, P.; Hoover, E.; Blackburn, M.; Gallun, F.	2018
Differences in word and phoneme recognition in quiet, sentence recognition in noise, and subjective outcomes between manufacturer first-fit and hearing aids programmed to NAL-NL2 using real-ear measures	Valente, M.; Oeding, K.; Brockmeyer, A.; Smith, S.; Kallogjeri, D.	2018
An argument for self-report as a reference standard in audiology	Vermiglio, A. J.; Soli, S. D.; Fang, X.	2018
Listener factors associated with individual susceptibility to reverberation	Reinhart, P. N.; Souza, P. E.	2018
Impact of hearing loss and amplification on performance on a cognitive screening test	Saunders, G. H.; Odgear, I.; Cosgrove, A.; Frederick, M. T.	2018
The influence of speech rate on acceptable noise levels	Tiffin, S.; Gordon-Hickey, S.	2018
Evaluation of a stereo music preprocessing scheme for cochlear implant users	Buyens, W.; Van Dijk, B.; Moonen, M.; Wouters, J.	2018
Speech understanding and sound source localization by cochlear implant listeners using a pinna-effect imitating microphone and an adaptive beamformer	Dorman, M. F.; Natale, S.; Loiselle, L.	2018
Clinical application and psychometric properties of a norwegian questionnaire for the self-assessment of communication in quiet and adverse conditions using two revised APHAB subscales	Laugen Heggdal, P. O.; Nordvik, O.; Brannstrom, J.; Vassbotn, F.; Aarstad, A. K.; Aarstad, H. J.	2018
Psychometric properties of the Turkish version of the Satisfaction with Amplification in Daily Living questionnaire in hearing aid users	Genc, M.; Cildir, B.; Kaya, M.	2018

Auditory processing performance of the middle-aged and elderly: Auditory or cognitive decline?	Murphy, C. F. B.; Rabelo, C. M.; Silagi, M. L.; Mansur, L. L.; Bamiou, D. E.; Schochat, E.	2018
Long-Term Cognitive Prognosis of Profoundly Deaf Older Adults After Hearing Rehabilitation Using Cochlear Implants	Mosnier, I.; Vanier, A.; Bonnard, D.; Lina-Granade, G.; Truy, E.; Bordure, P.; Godey, B.; Marx, M.; Lescanne, E.; Venail, F.; Poncet, C.; Sterkers, O.; Belmin, J.	2018
Influence of visual impairment and hearing impairment on functional dependence status among people in Taiwan-An evaluation using the WHODAS 2.0 score	Chang, K. F.; Chang, K. H.; Chi, W. C.; Huang, S. W.; Yen, C. F.; Liao, H. F.; Liou, T. H.; Chao, P. Z.; Lin, I. C.	2018
Extended bandwidth nonlinear frequency compression in Mandarin-speaking hearing-aid users	Tseng, W. H.; Hsieh, D. L.; Shih, W. T.; Liu, T. C.	2018
Hearing and Quality of Life Among Community-Dwelling Older Adults	Polku, H.; Mikkola, T. M.; Rantakokko, M.; Portegijs, E.; Tormakangas, T.; Rantanen, T.; Viljanen, A.	2018
First results with a new, pressure-free, adhesive bone conduction hearing aid	Dahm, V.; Baumgartner, W. D.; Arnoldner, C.; Riss, D.	2018
Speech recognition in Patients with Semicircular canals aplasia after Cochlear implantation	Ahmed, A.; Lesinski-Schiedat, A.; Giourgas, A.; Giesemann, A.; Lenarz, T.	2018
Experience with CI-Treatment in cases of longterm deafness	Niclaus, O.; Nguyen-Dalinger, D.; Mis, U.; Kulkens, C.; Preuss, M.	2018

Surgical experience and results in preservation of residual hearing in patients provided with 532 electrodes	Arweiler-Harbeck, D.; Hans, S.; Arnolds, J.; Riemann, N.; Ludwig, S.	2018
Genotype-phenotype correlation in hereditary hearing loss	Dofek, S.; Gamerdinger, P.; Fehr, S.; Biskup, S.; Muller, M.; Lowenheim, H.; Tropitzsch, A.	2018
Longterm results of the MET T1 and T2 transducers coupled to the incus	Giere, T.; Prenzler, N.; Salcher, R. B.; Kludt, E.; Maier, H.; Lenarz, T.	2018
Effect of insertion depth on hearing preservation after CI: Indication for electric or electric-acoustic stimulation	Helbig, S.; Adel, Y.; Weissgerber, T.; Baumann, U.; Stover, T.	2018
Impact of room acoustic parameters on speech and music perception on patients with cochlear implants	Klenzner, T.; Eurich, B.; Volpert, S.; Oehler, M.	2018
Two-year results with the biohybrid cochlear electrodes:safe electrode?	Mitovska, D.; Kludt, E.; Lesinski-Schiedat, A.; Lenarz, T.; Warnecke, A.	2018
The use of hearing aids as part of the therapy in patients with chronic tinnitus	Quaas, T.; Ivansic, D.; Guntinas-Lichius, O.	2018
Audiometric and caloric function in meniere disease	Sefa, A.; Sefa, M.	2018
Patient specific electrode location after CI surgery	Timm, M.; Boruchov, A.; Weller, T.; Lenarz, T.; Salcher, R. B.; Warnecke, A.; Andreas, B.	2018
A clinical tool for the prediction of cochlear implantation outcomes	Weller, T.; Timm, M.; Buchner, A.; Lenarz, T.	2018
Assessment of the quality of life of cochlear implant carriers of retirement age	Bajorath, W.; Lesinski Schiedat, A.; Giourgas, A.; Lenarz, T.; Illg, A.	2018

Rapid tinnitus relief and improvement of health related quality of life, psychological comorbidities and subjective speech perception after cochlear implantation in patients with single sided deafness	Haussler, S. M.; Kopke, V. A.; Grabel, S.; Knopke, S.; Olze, H.	2018
Influence of cochlear implantation on cognitive abilities in post-lingual hearing impaired people aged 70 years or older	Knopke, S.; Schubert, A.; Haussler, S.; Szczepek, A.; Grabel, S.; Olze, H.	2018
Longitudinal follow-up evaluation of cochlea implant rehabilitation at cochlear implant center cic schleswig-kiel	Brademann, G.; Bohnke, B.; Mewes, A.; Hey, M.; Ambrosch, P.	2018
Impact of different stimulation rates on speech understanding and frequency discrimination using the ace strategy	Buchner, A.; Kludt, E.; Schussler, M.; Lenarz, T.	2018
Postoperative speech comprehension of the cochlearTM nucleus profile implant with slim-modiolar-electrode (CI532)	Endemann, E.; Stover, T.; Helbig, S.	2018
Cochlear implantation in otosclerosis: Comparison of impedances and speech perception versus standard collective	Fenov, L.; Warnecke, A.; Kludt, E.; Lenarz, T.; Prenzler, N. K.	2018
The relationship between speech intelligibility and speech perception in cochlear implant patients	Freimann, N.; Polterauer, D.; Gollwitzer, S.; Muller, J.; Schuster, M. E.	2018
Speech discrimination score development over time after cochlear implantation	Kacker, C.; Praetorius, M.; Hoth, S.	2018
The first implanted ear profits from the rehabilitation of the contralateral side in sequential bilateral cochlear implantation	Leinung, M.; Linke, A.; Loth, A.; Groger, M.; Stover, T.	2018
Speech understanding dependent from electrode insertion depth within CI recipients	Lesinski-Schiedat, A.; Manecke, D.; Kludt, E.; Lenarz, T.; Bultmann, E.	2018
MRI-based prediction of audiological outcome in elderly CI-users	Raphael, F.; Bauknecht, H. C.; Schubert, A.; Haussler, S.; Szczepek, A.; Grabel, S.; Olze, H.; Knopke, S.	2018

Cochlear implant performance in patients with TMPRSS3 mutations	Tropitzsch, A.; Knoblich, N.; Miiller, M.; Biskup, S.; Lowenheim, H.; Holderried, M.; Rask-Andersen, H.	2018
Speechtest s for CI users without using hearing booth?	Volpert, S.; Klenzner, T.; Jansen, N.; Blumel, L.; Schipper, J.	2018
Influence of background noise level on speech reception in normal und hearing impaired persons	Wardenga, N.; Zokoll, M. A.; Kollmeier, B.; Maier, H.	2018
Relating quality of life to outcomes and predictors in adult cochlear implant users: Are we measuring the right things?	Moberly, A. C.; Harris, M. S.; Boyce, L.; Vasil, K.; Wucinich, T.; Pisoni, D. B.; Baxter, J.; Ray, C.; Shafiro, V.	2018
Long-term outcomes of cochlear implantation in patients with high-frequency hearing loss	Roland, J. T.; Gantz, B. J.; Waltzman, S. B.; Parkinson, A. J.	2018
Totally implantable hearing system: Five-year hearing results	Shohet, J. A.; Kraus, E. M.; Catalano, P. J.; Toh, E.	2018
How does aging affect recognition of spectrally degraded speech?	Moberly, A. C.; Vasil, K. J.; Wucinich, T. L.; Safdar, N.; Boyce, L.; Roup, C.; Holt, R. F.; Adunka, O. F.; Castellanos, I.; Shafiro, V.; Houston, D. M.; Pisoni, D. B.	2018
Automated smartphone audiometry: Validation of a word recognition test app	Dewyer, N. A.; Jiradejvong, P.; Sabes, J. H.; Limb, C. J.	2018

Evaluating speech perception of the MAXUM middle ear implant versus speech perception under inserts	Dyer, R. K.; Spearman, M.; Spearman, B.; McCraney, A.	2018
Acoustic plus electric speech processing: Long-term results	Gantz, B. J.; Dunn, C. C.; Oleson, J.; Hansen, M. R.	2018
Totally implantable active middle ear implant: Hearing and safety results in a large series	Shohet, J. A.; Gende, D. M.; Tanita, C. S.	2018
Cognitive Functions in Adult Cochlear Implant Users, Cochlear Implant Candidates, and Normal-Hearing Listeners	Kramer, S.; Vasil, K. J.; Adunka, O. F.; Pisoni, D. B.; Moberly, A. C.	2018
Factors associated with benefit of active middle ear implants compared to conventional hearing aids	McRackan, T. R.; Clinkscales, W. B.; Ahlstrom, J. B.; Nguyen, S. A.; Dubno, J. R.	2018
Combined effect of self-reported hearing problems and level of social activities on the risk of disability in Japanese older adults: A population-based longitudinal study	Bae, S.; Lee, S.; Lee, S.; Harada, K.; Makizako, H.; Park, H.; Shimada, H.	2018
Evaluation of mandarin chinese speech recognition in adults with cochlear implants using the spectral ripple discrimination test	Dai, C.; Zhao, Z.; Shen, W.; Zhang, D.; Lei, G.; Qiao, Y.; Yang, S.	2018
Anatomical and Functional MRI Changes after One Year of Auditory Rehabilitation with Hearing Aids	Pereira-Jorge, M. R.; Andrade, K. C.; Palhano-Fontes, F. X.; Diniz, P. R. B.; Sturzbecher, M.; Santos, A. C.; Araujo, D. B.	2018

Relationship between central and peripheral presbycusis and MCI in a population-based study of southern Italy: The "great age study"	Sardone, R.; Battista, P.; Tortelli, R.; Capozzo, R.; Piccininni, M.; Leo, A.; Coppola, F.; Guerra, V.; Abbrescia, D. I.; Grasso, A.; Barulli, O.; Di Dio, C.; Lozupone, M.; Seripa, D.; Panza, F.; Quaranta, N.; Logroscino, G.	2018
Analysis of the relationship between cognitive skills and unilateral sensory hearing loss	Calderon-Leyva, I.; Diaz-Leines, S.; Arch-Tirado, E.; Lino-Gonzalez, A. L.	2018
Quality of life in patients with unilateral vestibular schwannoma on wait and see - strategy	Klersy, P. C.; Arlt, F.; Hofer, M.; Meixensberger, J.	2018
Hearing impairment in patients with myotonic dystrophy type 2	Van Vliet, J.; Tieleman, A. A.; Van Engelen, B. G. M.; Bassez, G.; Servais, L.; Behin, A.; Stojkovic, T.; Meulstee, J.; Engel, J. A. M.; Lamas, G.; Eymard, B.; Verhagen, W. I. M.; Mamelle, E.	2018
Relationship between central and peripheral presbycusis and mild cognitive impairment in a population-based study of Southern Italy: The "Great Age Study"	Sardone, R.; Battista, P.; Tortelli, R.; Piccininni, M.; Coppola, F.; Guerra, V.; Abbrescia, D. I.; Grasso, A.; Barulli, O.; Didio, C.; Lozupone, M.; Seripa, D.; Panza, F.; Quaranta, N.; Logroscino, G.	2018

Restoration of sensory input may improve cognitive and neural function	Karawani, H.; Jenkins, K.; Anderson, S.	2018
Nationwide Prevalence of Self-Reported Serious Sensory Impairments and Their Associations with Self-Reported Cognitive and Functional Difficulties	Fuller, S. D.; Mudie, L. I.; Siordia, C.; Swenor, B. K.; Friedman, D. S.	2018
Comparable Electrode Impedance and Speech Perception at 12 Months after Cochlear Implantation Using Round Window versus Cochleostomy: An Analysis of 40 Patients	Cheng, X.; Wang, B.; Liu, Y.; Yuan, Y.; Shu, Y.; Chen, B.	2018
Fluctuating Hearing Loss in the Only Hearing Ear: Cochlear Implantation in the Contralateral Deaf Side	Russo, F. Y.; De Seta, D.; Lahlou, G.; Borel, S.; Nguyen, Y.; Bouccara, D.; Sterkers, O.; Bernardeschi, D.; Mosnier, I.	2018
Assessment of Disease-Specific and General Patient-Reported Outcome Measures of Hearing Health	Mohan, S.; Corrales, C. E.; Yueh, B.; Shin, J. J.	2018
Immediate and one-year outcomes of the new slim modiolar cochlear implant electrode array	Durakovic, N.; McJunkin, J.; Wick, C. C.; Kallogjeri, D.; Buchman, C. A.; Herzog, J. A.	2018
Comparison of in-person versus telemedicine cochlear implant evaluations: Pilot study	Fletcher, K.; Dicken, F. W.; Adkins, M. M.; Cline, T. A.; Shinn, J. B.; McNulty, B. N.; Bush, M. L.	2018
Novel computer-based therapy enhances speech perception in cochlear implant users	Narayan, A.	2018
Active middle ear implant totally implanted: Carina system first results	Peixoto, M. C.	2018
Intratympanic dexamethasone versus methylprednisolone for sudden hearing loss	Tadokoro, K.; Marzo, S. J.	2018
Asymmetric Hearing Loss Prompting MRI Referral in a Military Population: Redefining Audiometric Criteria	Tolisano, A. M.; Burgos, R. M.; Lustik, M. B.; Mitchell, L. A.; Littlefield, P. D.	2018

Do temporary threshold shifts occur following temporal bone fracture?	Wong, K.; Knoll, R. M.; Chen, J. X.; Khatib, D.; Jung, D. H.; Remenschneider, A. K.; Kozin, E. D.	2018
Improving Measurement Efficiency of the Inner EAR Scale with Item Response Theory	Jessen, A.; Ho, A. D.; Corrales, C. E.; Yueh, B.; Shin, J. J.	2018
Electrical dynamic range is only weakly associated with auditory performance and speech recognition in long-term users of cochlear implants	Lee, J. H.	2018
Steroid Use for Sudden Sensorineural Hearing Loss: A CHEER Network Study	Witsell, D. L.; Mulder, H.; Rauch, S.; Schulz, K. A.; Tucci, D. L.	2018
What to Do When Cochlear Implant Users Plateau in Performance: a Pilot Study of Clinician-guided Aural Rehabilitation	Moberly, A. C.; Vasil, K.; Baxter, J.; Ray, C.	2018
Predicting Performance and Non-Use in Prelingually Deaf and Late-Implanted Cochlear Implant Users	Lammers, M. J. W.; Versnel, H.; Topsakal, V.; van Zanten, G. A.; Grolman, W.	2018
Prevalence of Hearing Loss and Hearing Care Use Among Asian Americans: A Nationally Representative Sample	Choi, J. S.; Kari, E.; Friedman, R. A.; Fisher, L. M.	2018

Multicenter US Clinical Trial With an Electric-Acoustic Stimulation (EAS) System in Adults: Final Outcomes	Pillsbury, H. C.; Dillon, M. T.; Buchman, C. A.; Staecker, H.; Prentiss, S. M.; Ruckenstein, M. J.; Bigelow, D. C.; Telischi, F. F.; Martinez, D. M.; Runge, C. L.; Friedland, D. R.; Blevins, N. H.; Larky, J. B.; Alexiades, G.; Kaylie, D. M.; Roland, P. S.; Miyamoto, R. T.; Backous, D. D.; Warren, F. M.; El-Kashlan, H. K.; Slager, H. K.; Reyes, C.; Racey, A. I.; Adunka, O. F.	2018
Cochlear Implantation in Adults With Asymmetric Hearing Loss: Speech Recognition in Quiet and in Noise, and Health Related Quality of Life	Sladen, D. P.; Carlson, M. L.; Dowling, B. P.; Olund, A. P.; DeJong, M. D.; Breneman, A.; Hollander, S.; Beatty, C. W.; Neff, B. A.; Driscoll, C. L.	2018
Relations Between Self-reported Executive Functioning and Speech Perception Skills in Adult Cochlear Implant Users	Moberly, Aaron C.; Patel, Tirth R.; Castellanos, Irina	2018
Immediate and Long-term Hearing Outcomes With the Middle Cranial Fossa Approach for Vestibular Schwannoma Resection	Ahmed, S.; Arts, H. A.; El-Kashlan, H.; Basura, G. J.; Thompson, B. G.; Telian, S. A.	2018

Evaluation of Outcome Variability Associated With Lateral Wall, Mid-scalar, and Perimodiolar Electrode Arrays When Controlling for Preoperative Patient Characteristics	Fabie, J. E.; Keller, R. G.; Hatch, J. L.; Holcomb, M. A.; Camposeo, E. L.; Lambert, P. R.; Meyer, T. A.; McRackan, T. R.	2018
Hearing Outcomes in Conservatively Managed Vestibular Schwannoma Patients With Serviceable Hearing	Hunter, J. B.; Dowling, E. M.; Lohse, C. M.; O'Connell, B. P.; Tombers, N. M.; Lees, K. A.; Thompson, R. S.; Haynes, D. S.; Carlson, M. L.	2018
Direct Acoustic Cochlear Implants Lead to an Improved Speech Perception Gap Compared to Conventional Hearing Aid	Maier, H.; Lenarz, T.; Dollezal, L. V.; Busch, S.	2018
Early Outcomes With a Slim, Modiolar Cochlear Implant Electrode Array	McJunkin, J. L.; Durakovic, N.; Herzog, J.; Buchman, C. A.	2018
Earphone and Aided Word Recognition Differences in Cochlear Implant Candidates	McRackan, T. R.; Fabie, J. E.; Burton, J. A.; Munawar, S.; Holcomb, M. A.; Dubno, J. R.	2018
Hearing Outcomes After Stereotactic Radiosurgery for Jugular Paraganglioma	Patel, N. S.; Link, M. J.; Driscoll, C. L. W.; Pollock, B. E.; Lohse, C. M.; Carlson, M. L.	2018
Cochlear Implantation After Partial or Subtotal Cochleoectomy for Intracochlear Schwannoma RemovalA Technical Report	Plontke, S. K.; Kosling, S.; Rahne, T.	2018
Improved Speech Perception in Cochlear Implant Users With Interleaved High-Rate Pulse Trains	Runge, C. L.; Du, F.; Hu, Y.	2018
Preliminary Outcomes Report for CO2 Laser Assisted Electric-Acoustic Cochlear Implantation	Stevens, S. M.; Redmann, A.; Whitaker, K.; Ruotanen, A.; Houston, L.; Hammer, T.; Samy, R. N.	2018

Cochlear Implantation in Cochlear Ossification: Retrospective Review of Etiologies, Surgical Considerations, and Auditory Outcomes	Vashishth, A.; Fulcheri, A.; Prasad, S. C.; Bassi, M.; Rossi, G.; Caruso, A.; Sanna, M.	2018
Speech Perception in Quiet and Noise With an Off the Ear CI Processor Enabling Adaptive Microphone Directionality	Wesarg, T.; Voss, B.; Hassepass, F.; Beck, R.; Aschendorff, A.; Laszig, R.; Arndt, S.	2018
Beyond Sentence Recognition in Quiet for Older Adults: Implications for Cochlear Implant Candidacy	Zhang, E.; Coelho, D. H.	2018
Right Ear Advantage of Speech Audiometry in Single-sided Deafness	Wettstein, V. G.; Probst, R.	2018
Cognitive Performance of Severely Hearing-impaired Older Adults Before and After Cochlear Implantation: Preliminary Results of a Prospective, Longitudinal Cohort Study Using the RBANS-H	Claes, A. J.; Van de Heyning, P.; Gilles, A.; Van Rompaey, V.; Mertens, G.	2018
No Squelch Effect After Sequential Bilateral Cochlear Implantation in Postlingually Deafened Adults: Is There a First Ear Advantage?	Kraaijenga, V. J. C.; Smit, A. L.; Ramakers, G. G. J.; Stokroos, R. J.; van Zanten, G. A.	2018
Bilateral Cochlear Implantation Versus Bimodal Hearing in Patients With Functional Residual Hearing: A Within-subjects Comparison of Audiologic Performance and Quality of Life	Yawn, R. J.; O'Connell, B. P.; Dwyer, R. T.; Sunderhaus, L. W.; Reynolds, S.; Haynes, D. S.; Gifford, R. H.	2018
Neurocognitive Factors Contributing to Cochlear Implant Candidacy	Moberly, A. C.; Castellanos, I.; Mattingly, J. K.	2018
"Product" Versus "Process" Measures in Assessing Speech Recognition Outcomes in Adults With Cochlear Implants	Moberly, A. C.; Castellanos, I.; Vasil, K. J.; Adunka, O. F.; Pisoni, D. B.	2018
Audiologic Natural History of Small Volume Cochleovestibular Schwannomas in Neurofibromatosis Type 2	deTorres, A. T.; Brewer, C. C.; Zalewski, C. K.; King, K. A.; Walker, R.; Scott, G. C.; Asthagiri, A. R.; Chittiboina, P.; Kim, H. J.	2018

Nonverbal Reasoning as a Contributor to Sentence Recognition Outcomes in Adults With Cochlear Implants	Mattingly, J. K.; Castellanos, I.; Moberly, A. C.	2018
Cochlear Implantation in Chronic Otitis Media: Investigation of Long-term Speech Comprehension and Rate of Complications	Rak, K.; Volker, J.; Schendzielorz, P.; Kaulitz, S.; Steinbach, J.; Shehata-Dieler, W.; Schraven, S. P.; Mlynski, R.; Radeloff, A.; Hagen, R.	2018
Assessment of the State of the Natural Antioxidant Barrier of a Body in Patients Complaining about the Presence of Tinnitus	Pawlak-Osinska, K.; Kazmierczak, H.; Marzec, M.; Kupczyk, D.; Bilski, R.; Mikolajewska, E.; Mikolajewski, D.; Augustynska, B.	2018
Clinical study on the efficacy, acceptance, and safety of hearing aids in patients with mild to moderate presbyacusis	Romanet, P.; Guy, M.; Allaert, F. A.	2018
Combined impairments in vision, hearing and cognition are associated with greater levels of functional and communication difficulties than cognitive impairment alone: Analysis of interRAI data for home care and long-term care recipients in Ontario	Guthrie, D. M.; Davidson, J. G. S.; Williams, N.; Campos, J.; Hunter, K.; Mick, P.; Orange, J. B.; Pichora-Fuller, M. K.; Phillips, N. A.; Savundranayagam, M. Y.; Wittich, W.	2018
Dual sensory impairment: The association between glaucomatous vision loss and hearing impairment and function	Mudie, L. I.; Varadaraj, V.; Gajwani, P.; Munoz, B.; Ramulu, P.; Lin, F. R.; Swenor, B. K.; Friedman, D. S.; Zebardast, N.	2018
Barriers to and enablers of the implementation of an ICF-based intake tool in clinical otology and audiology practice-A qualitative pre-implementation study	Van Leeuwen, L. M.; Pronk, M.; Merkus, P.; Goverts, S. T.; Anema, J. R.; Kramer, S. E.	2018

TV listening and hearing aids	Strelcyk, O.; Singh, G.	2018
The effects of electrical field spatial spread and some cognitive factors on speech-in-noise performance of individual cochlear implant users-A computer model study	Jurgens, T.; Hohmann, V.; Buchner, A.; Nogueira, W.	2018
Fixed and adaptive beamforming improves speech perception in noise in cochlear implant recipients equipped with the MED-EL SONNET audio processor	Honeder, C.; Liepins, R.; Arnoldner, C.; Sinkovec, H.; Kaider, A.; Vyskocil, E.; Riss, D.	2018
Idiopathic sudden sensorineural hearing loss in dialysis patients	Kang, S. M.; Lim, H. W.; Yu, H.	2018
Is there a best side for cochlear implants in post-lingual patients?	Amaral, Msad; Damico, T. A.; Goncales, A. S.; Reis, Acmb; Isaac, M. L.; Massuda, E. T.; Hyppolito, M. A.	2018
Women's lived experiences of disabling hearing loss in daily life	Jonsson, I.; Hedelin, B.	2018
Audiovisual Temporal Processing in Postlingually Deafened Adults with Cochlear Implants	Butera, I. M.; Stevenson, R. A.; Mangus, B. D.; Woynaroski, T. G.; Gifford, R. H.; Wallace, M. T.	2018
Cochlear Implantation in Postlingually Deaf Adults is Time-sensitive Towards Positive Outcome: Prediction using Advanced Machine Learning Techniques	Kim, H.; Kang, W. S.; Park, H. J.; Lee, J. Y.; Park, J. W.; Kim, Y.; Seo, J. W.; Kwak, M. Y.; Kang, B. C.; Yang, C. J.; Duffy, B. A.; Cho, Y. S.; Lee, S. Y.; Suh, M. W.; Moon, I. J.; Ahn, J. H.; Cho, Y. S.; Oh, S. H.; Chung, J. W.	2018
Benefits from, Satisfaction with, and Self-Efficacy for Advanced Digital Hearing Aids in Users with Mild Sensorineural Hearing Loss	Johnson, C. E.; Jilla, A. M.; Danhauer, J. L.; Sullivan, J. C.; Sanchez, K. R.	2018

Interaction between speech variations and background noise on speech intelligibility by Mandarin-speaking cochlear implant patients	Shi, Y.; Peng, K. A.; Chen, B.; Gong, Y.; Chen, J. Y.; Li, Y. X.; Fu, Q. J.	2018
A survey and clinical evaluation of hearing aid data-logging: a valued but underutilized hearing aid fitting tool	McMillan, A.; Durai, M.; Searchfield, G. D.	2018
Age-corrected hearing loss after chemoradiation in cervical cancer patients	Marnitz, S.; Schermeyer, L.; Dommerich, S.; Kohler, C.; Olze, H.; Budach, V.; Martus, P.	2018
Vestibulo-Cochlear Function After Cochlear Implantation in Patients With Meniere's Disease	Manrique-Huarte, R.; Calavia, D.; Alvarez-Gomez, L.; Huarte, A.; Perez-Fernandez, N.; Manrique, M.	2018
Development and Clinical Introduction of the Nurotron Cochlear Implant Electrode Array	Rebscher, S.; Zhou, D. D.; Zeng, F. G.	2018
Intratympanic Methylprednisolone versus Dexamethasone for the Primary Treatment of Idiopathic Sudden Sensorineural Hearing Loss	Tarkan, O.; Dagkiran, M.; Surmelioglu, O.; Ozdemir, S.; Tuncer, U.; Dogrusoz, M.; Cetik, F.; Kiroglu, M.	2018
Talker identification: Effects of masking, hearing loss, and age	Best, V.; Ahlstrom, J. B.; Mason, C. R.; Roverud, E.; Perrachione, T. K.; Kidd, G.; Dubno, J. R.	2018
A deep learning based segregation algorithm to increase speech intelligibility for hearing-impaired listeners in reverberant-noisy conditions	Zhao, Y.; Wang, D.; Johnson, E. M.; Healy, E. W.	2018
Current Profile of Adults Presenting for Preoperative Cochlear Implant Evaluation	Holder, J. T.; Reynolds, S. M.; Sunderhaus, L. W.; Gifford, R. H.	2018
Comparison of Two Music Training Approaches on Music and Speech Perception in Cochlear Implant Users	Fuller, C. D.; Galvin, J. J.; Maat, B.; Baskent, D.; Free, R. H.	2018

Do Hearing Aids Address Real-World Hearing Difficulties for Adults With Mild Hearing Impairment? Results From a Pilot Study Using Ecological Momentary Assessment	Timmer, B. H. B.; Hickson, L.; Launer, S.	2018
Development and Application of an Annotation Procedure to Assess the Impact of Hearing Aid Amplification on Interpersonal Communication Behavior	Meis, M.; Krueger, M.; Gablenz, P. V.; Holube, I.; Gebhard, M.; Latzel, M.; Paluch, R.	2018
Current Focusing to Reduce Channel Interaction for Distant Electrodes in Cochlear Implant Programs	DeVries, L.; Arenberg, J. G.	2018
Emotional Responses to Pleasant Sounds Are Related to Social Disconnectedness and Loneliness Independent of Hearing Loss	Picou, E. M.; Buono, G. H.	2018
The Pupil Dilation Response During Speech Perception in Dark and Light: The Involvement of the Parasympathetic Nervous System in Listening Effort	Wang, Y.; Kramer, S. E.; Wendt, D.; Naylor, G.; Lunner, T.; Zekveld, A. A.	2018
Effect of Hearing Aid Directionality and Remote Microphone on Speech Intelligibility in Complex Listening Situations	Wagener, K. C.; Vormann, M.; Latzel, M.; Mulder, H. E.	2018
Hearing Aids Benefit Recognition of Words in Emotional Speech but Not Emotion Identification	Goy, H.; Pichora-Fuller, M. K.; Singh, G.; Russo, F. A.	2018
A "Buildup" of Speech Intelligibility in Listeners With Normal Hearing and Hearing Loss	Best, V.; Swaminathan, J.; Kopco, N.; Roverud, E.; Shinn-Cunningham, B.	2018
Effects of Cochlear Implantation on Binaural Hearing in Adults With Unilateral Hearing Loss	Buss, E.; Dillon, M. T.; Rooth, M. A.; King, E. R.; Deres, E. J.; Buchman, C. A.; Pillsbury, H. C.; Brown, K. D.	2018
Effects of Binaural Spatialization in Wireless Microphone Systems for Hearing Aids on Normal-Hearing and Hearing-Impaired Listeners	Courtois, G.; Lissek, H.; Estoppey, P.; Oesch, Y.; Gigandet, X.	2018
Speech Understanding With Various Maskers in Cochlear-Implant and Simulated Cochlear-Implant Hearing: Effects of Spectral Resolution and Implications for Masking Release	Croghan, N. B. H.; Smith, Z. M.	2018

Measuring the Impact of Tinnitus on Aided Listening Effort Using Pupillary Response	Jensen, J. J.; Callaway, S. L.; Lunner, T.; Wendt, D.	2018
Improving Speech Recognition in Bilateral Cochlear Implant Users by Listening With the Better Ear	Kan, A.	2018
Effects of Slow- and Fast-Acting Compression on Hearing-Impaired Listeners' Consonant-Vowel Identification in Interrupted Noise	Kowalewski, B.; Zaar, J.; Fereczkowski, M.; MacDonald, E. N.; Strelcyk, O.; May, T.; Dau, T.	2018
Effect of Tinnitus and Duration of Deafness on Sound Localization and Speech Recognition in Noise in Patients With Single-Sided Deafness	Liu, Y. W.; Cheng, X.; Chen, B.; Peng, K.; Ishiyama, A.; Fu, Q. J.	2018
Self-Adjusted Amplification Parameters Produce Large Between-Subject Variability and Preserve Speech Intelligibility	Nelson, P. B.; Perry, T. T.; Gregan, M.; VanTasell, D.	2018
Effects of Expanding Envelope Fluctuations on Consonant Perception in Hearing-Impaired Listeners	Wiinberg, A.; Zaar, J.; Dau, T.	2018
Pupillometry Reveals That Context Benefit in Speech Perception Can Be Disrupted by Later-Occurring Sounds, Especially in Listeners With Cochlear Implants	Winn, M. B.; Moore, A. N.	2018
Cortical Speech Processing in Postlingually Deaf Adult Cochlear Implant Users, as Revealed by Functional Near-Infrared Spectroscopy	Zhou, X.; Seghouane, A.; Shah, A.; Innes-Brown, H.; Cross, W.; Litovsky, R.; McKay, C. M.	2018
Objective Prediction of Hearing Aid Benefit Across Listener Groups Using Machine Learning: Speech Recognition Performance With Binaural Noise-Reduction Algorithms	Schadler, M. R.; Warzybok, A.; Kollmeier, B.	2018
Efficacy of a Hearing Aid Noise Reduction Function	Wong, L. L. N.; Chen, Y.; Wang, Q.; Kuehnel, V.	2018
Adjusting Expectations: Hearing Abilities in a Population-Based Sample Using an SSQ Short Form	von Gablenz, P.; Otto-Sobotka, F.; Holube, I.	2018
Verbal Response Times as a Potential Indicator of Cognitive Load During Conventional Speech Audiometry With Matrix Sentences	Meister, H.; Rahlmann, S.; Lemke, U.; Besser, J.	2018

Evaluation of Auditory Functioning and Rehabilitation Using Patient-Reported Outcome Measures	Lansbergen, S.; De Ronde-Brons, I.; Boymans, M.; Soede, W.; Dreschler, W. A.	2018
Age, Hearing, and the Perceptual Learning of Rapid Speech	Manheim, M.; Lavie, L.; Banai, K.	2018
Bilaterally Combined Electric and Acoustic Hearing in Mandarin-Speaking Listeners: The Population With Poor Residual Hearing	Tao, D. D.; Liu, J. S.; Yang, Z. D.; Wilson, B. S.; Zhou, N.	2018
Acupuncture as an early treatment for idiopathic sudden sensorineural hearing loss (ISSNHL) patients with flat or high-frequency drop audiograms: Study protocol for a randomized controlled trial	Shang, K.; Ma, X.; Liu, H. L.; Jing, Y. Y.; Zeng, L.; Li, N.; Zhou, D. A.; Wei, J.; Zhang, C.	2018
Comparison of Different Treatment Methods for Idiopathic Sudden Sensorineural Hearing Loss	Toroslu, T.; Erdogan, H.; Caglar, O.; GuClu, O.; Derekoy, F. S.	2018
Functional Results in Patients with Bone Anchored Hearing Aid	Bitere, O.; Martu, C.; Olariu, R.; Cobzeanu, B.; Martu, D.; Cozma, S.; Bertesteanu, S. V. G.; Grigore, R.	2018
Hearing preservation after removal of small vestibular schwannomas by retrosigmoid approach: comparison of two different ABR neuromonitoring techniques	Mastronardi, L.; Di Scipio, E.; Cacciotti, G.; Roperto, R.; Scavo, C. G.	2019
Influence of microphone soiling on syllable recognition in cochlear implants: simulation and recognition in noise	Cucis, P. A.; Berger-Vachon, C.; Truy, E.; Hermann, R.; Thai Van, H.; Gallego, S.	2019
Binaural advantages in using a cochlear implant for adults with profound unilateral hearing loss	Lorens, A.; Kruszynska, M.; Obrycka, A.; Skarzynski, P. H.; Wilson, B.; Skarzynski, H.	2019
Outcomes of the Baha Attract System combined with auricle reconstruction in mandarin speaking patients with bilateral microtia-atresia	Fan, X. M.; Chen, Y.; Niu, X. M.; Wang, Y. B.; Fan, Y.; Chen, X. W.	2019

Comparison of two different bone anchored hearing instruments: Baha-5 vs Ponto-plus	Kara, A.; Guven, M.; Sinan Yilmaz, M.; Demir, D.; Adigul, C.; Durgut, M.; Elden, H.; Mutlu, F.; Iseri, M.	2019
Assessing performance on the Montreal Cognitive Assessment (MoCA) in experienced cochlear implant users: use of alternative scoring guidelines	Hillyer, J.; Parada, J. C.; Parbery-Clark, A.	2019
The Relationship Between Hearing Loss Self-Management and Hearing Aid Benefit and Satisfaction	Convery, E.; Keidser, G.; Hickson, L.; Meyer, C.	2019
Musician and Nonmusician Hearing Aid Setting Preferences for Music and Speech Stimuli	D'Onofrio, K. L.; Gifford, R. H.; Ricketts, T. A.	2019
A Follow-Up Clinical Trial Evaluating the Consumer-Decides Service Delivery Mode	Humes, L. E.; Kinney, D. L.; Main, A. K.; Rogers, S. E.	2019
Comparing the Effect of Different Hearing Aid Fitting Methods in Bimodal Cochlear Implant Users	Vroegop, J. L.; Dingemanse, J. G.; van der Schroeff, M. P.; Goedegebure, A.	2019
Audiological outcomes in sudden sensorineural hearing loss with presumed inner ear hemorrhage	Chen, K.; Wen, L.; Zong, L.; Liu, M.; Sun, J.; Wu, X.	2019
The Relationship Between Hearing Loss and Substance Use Disorders Among Adults in the U.S	McKee, M. M.; Meade, M. A.; Zazove, P.; Stewart, H. J.; Jannausch, M. L.; Ilgen, M. A.	2019
Hearing impairment after subarachnoid hemorrhage	Campbell, N.; Verschuur, C.; Mitchell, S.; McCaffrey, O.; Deane, L.; Taylor, H.; Smith, R.; Foulkes, L.; Glazier, J.; Darekar, A.; Haacke, M. E.; Bulters, D.; Galea, I.	2019

Investigation of word recognition for the elderly in speech and noise spatial separation	Peng, J. X.; Zhao, L.; Jiang, Y. M.	2019
Association between self-reported hearing impairment, use of a hearing aid and performance of instrumental activities of daily living	Borda, M. G.; Reyes-Ortiz, C. A.; Heredia, R. A.; Castellanos-Perilla, N.; Ayala Copete, A. M.; Soennesyn, H.; Cano-Gutierrez, C. A.; Perez-Zepeda, M. U.	2019
Hearing-impaired elderly people have smaller social networks: A population-based aging study	Ogawa, T.; Uchida, Y.; Nishita, Y.; Tange, C.; Sugiura, S.; Ueda, H.; Nakada, T.; Suzuki, H.; Otsuka, R.; Ando, F.; Shimokata, H.	2019
Using the ICF to Identify Contextual Factors That Influence Participation of Persons With Deafblindness	Jaiswal, A.; Aldersey, H. M.; Wittich, W.; Mirza, M.; Finlayson, M.	2019
Cochlear Implant Users with Otosclerosis: Are Hearing and Quality of Life Outcomes Worse than in Cochlear Implant Users without Otosclerosis?	Calvino, M.; Sanchez-Cuadrado, I.; Gavilan, J.; Lassaletta, L.	2019
The value of unilateral cis, ci-cros and bilateral cis, with and without beamformer microphones, for speech understanding in a simulation of a restaurant environment	Dorman, M. F.; Cook Natale, S.; Agrawal, S.	2019
Round Window Niche Drilling with Intratympanic Steroid Is a Salvage Therapy of Sudden Hearing Loss	Si, Y.; Jiang, H. L.; Chen, Y. B.; Chu, Y. G.; Chen, S. J.; Chen, X. M.; He, W. H.; Zheng, Y. Q.; Zhang, Z. G.	2019
Long-Term Outcomes of Vibroplasty Coupler Implantations to Treat Mixed/Conductive Hearing Loss	Zahnert, T.; Mlynski, R.; Lowenheim, H.; Beutner, D.; Hagen, R.; Ernst, A.; Zehlicke, T.; Kuhne, H.; Friese, N.; Tropitzsch, A.; Luers, J. C.; Todt, I.; Huttenbrink, K. B.	2019

Progression of hearing loss in the aging population: Repeated auditory measurements in the Rotterdam study	Rigters, S. C.; Van Der Schroeff, M. P.; Papageorgiou, G.; Baatenburg De Jong, R. J.; Goedegebure, A.	2019
Cochlear Implant in the Elderly: Results in Terms of Speech Perception and Quality of Life	Forli, F.; Lazzerini, F.; Fortunato, S.; Bruschini, L.; Berrettini, S.	2019
The Bonebridge in Adults with Mixed and Conductive Hearing Loss: Audiological and Quality of Life Outcomes	Skarzynski, P. H.; Ratuszniak, A.; Krol, B.; Koziel, M.; Osinska, K.; Cywka, K. B.; Sztabnicka, A.; Skarzynski, H.	2019
How Does Nonverbal Reasoning Affect Sentence Recognition in Adults with Cochlear Implants and Normal-Hearing Peers?	Moberly, A. C.; Mattingly, J. K.; Castellanos, I.	2019
Effectiveness and efficiency of a dedicated bimodal fitting formula	Cuda, D.; Murri, A.; Mainardi, A.; Chalupper, J.	2019
Outcome prediction for Bonebridge candidates based on audiological indication criteria	Wimmer, W.; von Werdt, M.; Mantokoudis, G.; Anschuetz, L.; Kompis, M.; Caversaccio, M.	2019
The speech perception after cochlear implantation: The hearing gain difference according to the implant systems is important?	Jang, J. H.; Mun, H. A.; Choo, O. S.; Park, H. Y.; Choung, Y. H.	2019
Infuence of audio-visual integration abilities and residual hearing on performances with cochlear implant in post-lingually deafened adults	Seghers, N.; Wathour, J.; Deggouj, N.	2019
Comparison of various outcome measures of transcutaneous bone conduction devices: Passive (Baha Attract) versus an active implant (BONEBRIDGETM)	Levie, C.; Bouzegta, R.; Mertens, G.; Heyndrickx, K.; Vanderveken, O.; Van De Heyning, P.; Topsakal, V.; Van Rompaey, V.	2019

The effect of cochlear size on cochlear implantation outcomes	Kuthubutheen, J.; Grewal, A.; Symons, S.; Nedzelski, J.; Shipp, D.; Lin, V.; Chen, J.	2019
Middle Ear Transducer: Long Term Stability of the Latest Generation T2	Prenzler, N. K.; Kludt, E.; Giere, T.; Salcher, R.; Lenarz, T.; Maier, H.	2019
Effects of oral zinc supplementation on patients with noise-induced hearing loss associated tinnitus: A clinical trial	Yeh, C. W.; Tseng, L. H.; Yang, C. H.; Hwang, C. F.	2019
Cochlear implantation as a treatment for single-sided deafness and asymmetric hearing loss: A randomized controlled evaluation of cost-utility	Marx, M.; Costa, N.; Lepage, B.; Taoui, S.; Molinier, L.; Deguine, O.; Fraysse, B.	2019
Study protocol for the validation of a new patient-reported outcome measure (PROM) of listening effort in cochlear implantation: The Listening Effort Questionnaire-Cochlear Implant (LEQ-CI)	Hughes, S. E.; Rapport, F.; Watkins, A.; Boisvert, I.; McMahon, C. M.; Hutchings, H. A.	2019
Walk, Talk and Listen: A pilot randomised controlled trial targeting functional fitness and loneliness in older adults with hearing loss	Jones, C. A.; Siever, J.; Knuff, K.; Van Bergen, C.; Mick, P.; Little, J.; Jones, G.; Murphy, M. A.; Kurtz, D.; Miller, H.	2019
Hearing threshold levels and hearing loss among people in Zhejiang, China: A population-based cross-sectional study	Wang, D.; Zhang, H.; Ma, H.; Zhang, L.; Yang, L.; Xu, L.	2019
The cognitive and psychosocial effects of auditory training and hearing aids in adults with hearing loss	Nkyekyer, J.; Meyer, D.; Pipingas, A.; Reed, N. S.	2019
Reliability and usability of a weighted version of the functional comorbidity index	Kabboord, A. D.; van Eijk, M.; van Dingenen, L.; Wouters, M.; Koet, M.; van Balen, R.; Achterberg, W. P.	2019

Factors associated with the efficiency of hearing aids for patients with age-related hearing loss	Wu, X.; Ren, Y.; Wang, Q.; Li, B.; Wu, H.; Huang, Z.; Wang, X.	2019
Audiological and clinical outcomes of a transcutaneous bone conduction hearing implant: Six-month results from a multicentre study	den Besten, C. A.; Monksfield, P.; Bosman, A.; Skarzynski, P. H.; Green, K.; Runge, C.; Wigren, S.; Blechert, J. I.; Flynn, M. C.; Mylanus, E. A. M.; Hol, M. K. S.	2019
The effect of cross-over frequency on binaural hearing performance of adults using electric-acoustic stimulation	Incerti, P. V.; Ching, T. Y.; Cowan, R.	2019
Controlled comparative clinical trial of hearing benefit outcomes for users of the CochlearTM Nucleus 7 Sound Processor with mobile connectivity	Warren, C. D.; Nel, E.; Boyd, P. J.	2019
Optimizing maps for electric acoustic stimulation users	Yoon, Y. S.; Shin, Y. R.; Kim, J. M.; Coltisor, A.; Chun, Y. M.	2019
Quality of life and social determinants in individual hearing AIDS users	Ribeiro, Uasl; Souza, V. C.; Lemos, S. M. A.	2019
Hear-Communicate-Remember: Feasibility of delivering an integrated intervention for family caregivers of people with dementia and hearing impairment via telehealth	Meyer, C. J.; Koh, S. S.; Hill, A. J.; Conway, E. R.; Ryan, B. J.; McKinnon, E. R.; Pachana, N. A.	2019
Sensorineural hearing loss in patients with vestibular schwannoma correlates with the presence of utricular hydrops as diagnosed on heavily T2-weighted MRI	Eliezer, M.; Poillon, G.; Maquet, C.; Gillibert, A.; Horion, J.; Marie, J. P.; Guichard, J. P.; Magne, N.; Attye, A.	2019

Gender differences in improvement of older-person-specific quality of life after hearing-aid fitting	Joanovic, E.; Kisvetrova, H.; Nemcek, D.; Kurkova, P.; Svejdikova, B.; Zapletalova, J.; Yamada, Y.	2019
Everyday technology use among older deaf adults	Singleton, J. L.; Remillard, E. T.; Mitzner, T. L.; Rogers, W. A.	2019
Comparing Two Hearing Aid Fitting Algorithms for Bimodal Cochlear Implant Users	Vroegop, J. L.; Homans, N. C.; van der Schroeff, M. P.; Goedegebure, A.	2019
A "Goldilocks" Approach to Hearing Aid Self-Fitting: Ear-Canal Output and Speech Intelligibility Index	Mackersie, C.; Boothroyd, A.; Lithgow, A.	2019
Effects of Hearing Loss and Fast-Acting Compression on Amplitude Modulation Perception and Speech Intelligibility	Wiinberg, A.; Jepsen, M. L.; Epp, B.; Dau, T.	2019
Dynamic Current Focusing: A Novel Approach to Loudness Coding in Cochlear Implants	de Jong, M. A. M.; Briaire, J. J.; Frijns, J. H. M.	2019
Factors Associated With Successful Setup of a Self-Fitting Hearing Aid and the Need for Personalized Support	Convery, E.; Keidser, G.; Hickson, L.; Meyer, C.	2019
Comparing the International Classification of Functioning, Disability, and Health Core Sets for Hearing Loss and Otorhinolaryngology/Audiology Intake Documentation at Mayo Clinic	Alfakir, R.; van Leeuwen, L. M.; Pronk, M.; Kramer, S. E.; Zapala, D. A.	2019
Achieved Gain and Subjective Outcomes for a Wide-Bandwidth Contact Hearing Aid Fitted Using CAM2	Arbogast, T. L.; Moore, B. C. J.; Puria, S.; Dundas, D.; Brimacombe, J.; Edwards, B.; Carr Levy, S.	2019
Benefits of Cochlear Implantation for Single-Sided Deafness: Data From the House Clinic-University of Southern California-University of California, Los Angeles Clinical Trial	Galvin, J. J.; Fu, Q. J.; Wilkinson, E. P.; Mills, D.; Hagan, S. C.; Lupo, J. E.; Padilla, M.; Shannon, R. V.	2019
Bimodal Hearing or Bilateral Cochlear Implants? Ask the Patient	Gifford, R. H.; Dorman, M. F.	2019

A Cross-Sectional Questionnaire Study of Tinnitus Awareness and Impact in a Population of Adult Cochlear Implant Users	Gomersall, P. A.; Baguley, D. M.; Carlyon, R. P.	2019
Biomarkers of Systemic Inflammation and Risk of Incident Hearing Loss	Gupta, S.; Curhan, S. G.; Curhan, G. C.	2019
Hearing in Real-Life Environments (HERE): Structure and Reliability of a Questionnaire on Perceived Hearing for Older Adults	Heinrich, A.; Mikkola, T. M.; Polku, H.; Tormakangas, T.; Viljanen, A.	2019
Evaluation of a New Algorithm to Optimize Audibility in Cochlear Implant Recipients	Holden, L. K.; Firszt, J. B.; Reeder, R. M.; Dwyer, N. Y.; Stein, A. L.; Litvak, L. M.	2019
A Laboratory Evaluation of Contextual Factors Affecting Ratings of Speech in Noise: Implications for Ecological Momentary Assessment	Jenstad, L. M.; Gillen, L.; Singh, G.; DeLongis, A.; Pang, F.	2019
Development of the Cochlear Implant Quality of Life Item Bank	McRackan, T. R.; Hand, B. N.; Samy, R. N.; Gubbels, S. P.; Golub, J. S.; Wilkinson, E. P.; Mills, D.; Carey, J. P.; Vorasubin, N.; Brunk, V.; Carlson, M. L.; Driscoll, C. L.; Sladen, D. P.; Camposeo, E. L.; Holcomb, M. A.; Lambert, P. R.; Meyer, T. A.; Thomas, C.; Moberly, A. C.; Blevins, N. H.; Larky, J. B.; Herzano, R. P.; Hoffer, M. E.; Prentiss, S. M.; Brant, J.; Hunter, J. B.; Isaacson, B.; Kutz, J. W.; Gurgel, R. K.; Zeitler, D. M.; Buchman, C. A.;	2019

Do Personality Factors Assessed Before Cochlear Implantation Predict Hearing-Related Quality Of Life After Cochlear Implantation in Postlingually Deafened Adults?	Muigg, F.; Bliem, H. R.; Holzner, B.; Kuhn, H.; Zorowka, P. G.; Weichbold, V. W.	2019
Clinical Assessment of Functional Hearing Deficits: Speech-in-Noise Performance	Phatak, S. A.; Brungart, D. S.; Zion, D. J.; Grant, K. W.	2019
Time From Hearing Aid Candidacy to Hearing Aid Adoption: A Longitudinal Cohort Study	Simpson, A. N.; Matthews, L. J.; Cassarly, C.; Dubno, J. R.	2019
The Emotional Communication in Hearing Questionnaire (EMO-CHeQ): Development and Evaluation	Singh, G.; Liskovoi, L.; Launer, S.; Russo, F.	2019
Efficacy and Effectiveness of Advanced Hearing Aid Directional and Noise Reduction Technologies for Older Adults With Mild to Moderate Hearing Loss	Wu, Y. H.; Stangl, E.; Chipara, O.; Hasan, S. S.; DeVries, S.; Oleson, J.	2019
How Do You Deal With Uncertainty? Cochlear Implant Users Differ in the Dynamics of Lexical Processing of Noncanonical Inputs	McMurray, B.; Ellis, T. P.; Apfelbaum, K. S.	2019
Effect of Audibility and Suprathreshold Deficits on Speech Recognition for Listeners With Unilateral Hearing Loss	Bost, T. J. M.; Versfeld, N. J.; Goverts, S. T.	2019
Temporal Sensitivity Measured Shortly After Cochlear Implantation Predicts 6-Month Speech Recognition Outcome	Erb, J.; Ludwig, A. A.; Kunke, D.; Fuchs, M.; Obleser, J.	2019
Early Sentence Recognition in Adult Cochlear Implant Users	James, C. J.; Karoui, C.; Laborde, M. L.; Lepage, B.; Molinier, C.; Tartayre, M.; Escude, B.; Deguine, O.; Marx, M.; Fraysse, B.	2019
Perceptual Doping: An Audiovisual Facilitation Effect on Auditory Speech Processing, From Phonetic Feature Extraction to Sentence Identification in Noise	Moradi, S.; Lidestam, B.; Ning Ng, E. H.; Danielsson, H.; Ronnberg, J.	2019

The Effect of Hearing Aid Bandwidth and Configuration of Hearing Loss on Bimodal Speech Recognition in Cochlear Implant Users	Neuman, A. C.; Zeman, A.; Neukam, J.; Wang, B.; Svirsky, M. A.	2019
Evaluation of the Optimized Pitch and Language Strategy in Cochlear Implant Recipients	Vandali, A.; Dawson, P.; Au, A.; Yu, Y.; Brown, M.; Goorevich, M.; Cowan, R.	2019
A New Speech, Spatial, and Qualities of Hearing Scale Short-Form: Factor, Cluster, and Comparative Analyses	Moulin, A.; Vergne, J.; Gallego, S.; Micheyl, C.	2019
Effects of Reverberation on the Relation Between Compression Speed and Working Memory for Speech-in-Noise Perception	Reinhart, P.; Zahorik, P.; Souza, P.	2019
Age-Related Changes in Temporal Resolution Revisited: Electrophysiological and Behavioral Findings From Cochlear Implant Users	Mussoi, B. S. S.; Brown, C. J.	2019
Predicting Speech-in-Noise Deficits from the Audiogram	Shub, D. E.; Makashay, M. J.; Brungart, D. S.	2019
Genetic Inheritance of Late-Onset, Down-Sloping Hearing Loss and Its Implications for Auditory Rehabilitation	Song, M. H.; Jung, J.; Rim, J. H.; Choi, H. J.; Lee, H. J.; Noh, B.; Lee, J. S.; Gee, H. Y.; Choi, J. Y.	2019
Understanding Variability in Individual Response to Hearing Aid Signal Processing in Wearable Hearing Aids	Souza, P.; Arehart, K.; Schoof, T.; Anderson, M.; Strori, D.; Balmert, L.	2019
Perception of lexical stress cued by low-frequency pitch and insights into speech perception in noise for cochlear implant users and normal hearing adults	Dincer D'Alessandro, H.; Mancini, P.	2019
Cochlear implantation in incomplete partition type I	Eftekharian, A.; Eftekharian, K.; Mokari, N.; Fazel, M.	2019
Evaluation of wireless Bluetooth devices to improve recognition of speech and sentences when using a mobile phone in bone conduction device recipients	Kong, T. H.; Kwak, C.; Han, W.; Seo, Y. J.	2019
Expanding unilateral cochlear implantation criteria for adults with bilateral acquired severe sensorineural hearing loss	Huinck, W. J.; Mylanus, E. A. M.; Snik, A. F. M.	2019

Consequences of hearing aid acclimatization on ALLRs and its relationship with perceived benefit and speech perception abilities	Megha,; Maruthy, S.	2019
A new bone conduction hearing aid to predict hearing outcome with an active implanted device	Canale, A.; Boggio, V.; Albera, A.; Ravera, M.; Caranzano, F.; Lacilla, M.; Albera, R.	2019
The association between obstructive sleep apnea and hearing loss: a cross-sectional analysis	Kayabasi, S.; Hizli, O.; Yildirim, G.	2019
Benefits of a contralateral routing of signal device for unilateral Naida CI cochlear implant recipients	Mosnier, I.; Lahlou, G.; Flament, J.; Mathias, N.; Ferrary, E.; Sterkers, O.; Bernardeschi, D.; Nguyen, Y.	2019
The effect of aging on auditory function	Markelov, O.; Kaplun, D.; Buhl, M.; Warzybok, A.; Boboshko, M.; Kollmeier, B.	2019
Functional age-related changes within the human auditory system studied by audiometric examination	Profant, O.; Jilek, M.; Bures, Z.; Vencovsky, V.; Kucharova, D.; Svobodova, V.; Korynta, J.; Syka, J.	2019
Assessing cognitive abilities in high-performing cochlear implant users	Hillyer, J.; Elkins, E.; Hazlewood, C.; Watson, S. D.; Arenberg, J. G.; Parbery-Clark, A.	2019
No difference in behavioral and self-reported outcomes for simultaneous and sequential bilateral cochlear implantation: Evidence from a multicenter randomized controlled trial	Kraaijenga, V. J. C.; Ramakers, G. G. J.; Smulders, Y. E.; Van Zon, A.; Free, R. H.; Frijns, J. H. M.; Huinck, W. J.; Stokroos, R. J.; Grolman, W.	2019
Exploring differences in speech processing among older hearing-impaired listeners with or without hearing aid experience: Eye-tracking and FMRI measurements	Habicht, J.; Behler, O.; Kollmeier, B.; Neher, T.	2019

Hearing dysfunction in a large family affected by dominant optic atrophy (OPA8-related DOA): A human model of hidden auditory neuropathy	Santarelli, R.; La Morgia, C.; Valentino, M. L.; Barboni, P.; Monteleone, A.; Scimemi, P.; Carelli, V.	2019
Hearing loss and cognition among older adults in a Han Chinese cohort	Ren, F.; Luo, J.; Ma, W.; Xin, Q.; Xu, L.; Fan, Z.; Ai, Y.; Zhao, B.; Gao, F.; Wang, H.	2019
Comparison Between Simulated and Actual Unilateral Hearing in Sequentially Implanted Cochlear Implant Users, a Cohort Study	van Zon, A.; Smulders, Y. E.; Kraaijenga, V. J. C.; van Zanten, G. A.; Stokroos, R. J.; Stegeman, I.	2019
Coping with age-related hearing loss: patient-caregiver dyad effects on quality of life	Lazzarotto, S.; Martin, F.; Saint-Laurent, A.; Hamidou, Z.; Aghababian, V.; Auquier, P.; Baumstarck, K.	2019
Restoration of spatial hearing in adult cochlear implant users with single-sided deafness	Litovsky, R. Y.; Moua, K.; Godar, S.; Kan, A.; Misurelli, S. M.; Lee, D. J.	2019
Effects of directional sound processing and listener's motivation on EEG responses to continuous noisy speech: Do normal-hearing and aided hearing-impaired listeners differ?	Mirkovic, B.; Debener, S.; Schmidt, J.; Jaeger, M.; Neher, T.	2019
Improved interaural timing of acoustic nerve stimulation affects sound localization in single-sided deaf cochlear implant users	Seebacher, J.; Franke-Trieger, A.; Weichbold, V.; Zorowka, P.; Stephan, K.	2019
Neural envelope tracking as a measure of speech understanding in cochlear implant users	Verschueren, E.; Somers, B.; Francart, T.	2019

Frequency change detection and speech perception in cochlear implant users	Zhang, F.; Underwood, G.; McGuire, K.; Liang, C.; Moore, D. R.; Fu, Q. J.	2019
Brainstem encoding of frequency-modulated sweeps is relevant to Mandarin concurrent-vowels identification for normal-hearing and hearing-impaired listeners	Fu, Z.; Yang, H.; Chen, F.; Wu, X.; Chen, J.	2019
Impact of room acoustic parameters on speech and music perception among participants with cochlear implants	Eurich, B.; Klenzner, T.; Oehler, M.	2019
Effect of age and hearing loss on sentence perception in noise using temporal envelope and temporal fine structure cues	Jain, S.; Nataraja, N. P.	2019
Aided cortical auditory evoked measures with cochlear implantees: the challenge of stimulus artefacts	Choi, S. M. S.; Wong, E. C. M.; McPherson, B.	2019
Performance of older adults with hearing loss on the staggered spondaic word test-Spanish version (SSW-SV)	Canete, O. M.; Almasio, V.; Torrente, M. C.; Purdy, S. C.	2019
Maximum preimplantation monosyllabic score as predictor of cochlear implant outcome	Hoppe, U.; Hocke, T.; Hast, A.; Iro, H.	2019
Towards Decoding Selective Attention from Single-Trial EEG Data in Cochlear Implant Users	Nogueira, W.; Cosatti, G.; Schierholz, I.; Egger, M.; Mirkovic, B.; Buchner, A.	2019
Hearing rehabilitation through bone-conducted sound stimulation: Preliminary results	Bahmad, F.; Cardoso, C. C.; Caldas, F. F.; De Souza Chelminski Barreto, M. A.; Da Silva Hilgenberg, A. M.; Teixeira, M. S.; Serra, L. S. M.	2019
A preliminary investigation into hearing aid fitting based on automated real-ear measurements integrated in the fitting software: test-retest reliability, matching accuracy and perceptual outcomes	Denys, S.; Latzel, M.; Francart, T.; Wouters, J.	2019
Individualised active communication education (I-ACE): another clinical option for adults with hearing impairment with a focus on problem solving and self-management	Hickson, L.; Worrall, L.; Scarinci, N.; Laplante-Levesque, A.	2019

The World Health Organization's hearing-impairment grading system: an evaluation for unaided communication in age-related hearing loss	Humes, L. E.	2019
Content validity and readability of patient-reported questionnaire instruments of hearing disability	Manchaiah, V.; Granberg, S.; Grover, V.; Saunders, G. H.; Hall, D. A.	2019
Effect of signal processing strategy and stimulation type on speech and auditory perception in adult cochlear implant users	Reynolds, S. M.; Gifford, R. H.	2019
Reported benefits of peer support group involvement by adults with hearing loss	Southall, K.; Jennings, M. B.; Gagne, J. P.; Young, J.	2019
Development and validation of the FrBio, an international French adaptation of the AzBio sentence lists	Bergeron, F.; Berland, A.; Fitzpatric, E. M.; Vincent, C.; Giasson, A.; Kam, K. L.; Chafici, W.; Fanouillere, T.; Demers, D.	2019
Measurement of pitch perception as a function of cochlear implant electrode and its effect on speech perception with different frequency allocations	Grasmeder, M. L.; Verschuur, C. A.; van Besouw, R. M.; Wheatley, A. M. H.; Newman, T. A.	2019
Characteristics, behaviours and readiness of persons seeking hearing healthcare online	Ratanjee-Vanmali, H.; Swanepoel, D. W.; Laplante-Levesque, A.	2019
Longitudinal effect of deactivating stimulation sites based on low-rate thresholds on speech recognition in cochlear implant users	Zhou, N.	2019
Assessment of the psychometric properties of the AQoL-4D questionnaire in Kannada language for use with adults with hearing loss	Thammaiah, S.; Manchaiah, V.; Krishna, R.; Zekveld, A. A.; Kramer, S. E.	2019
Improving the efficiency of the digit triplet test using digit scoring with variable adaptive step sizes	Denys, S.; Hofmann, M.; van Wieringen, A.; Wouters, J.	2019

Open-class repair initiations in conversations involving middle-aged hearing aid users with mild to moderate loss	Laakso, M.; Salmenlinna, I.; Aaltonen, T.; Koskela, I.; Ruusuvuori, J.	2019
Perceptual organization of sequential stimuli in cochlear implant listeners: A temporal processing approach	Saki, N.; Nikakhlagh, S.; Mirmomeni, G.; Bayat, A.	2019
Therapeutic effect of intratympanic injection of dexamethasone plus hyaluronic acid on patients with meniere's disease	Rogha, M.; Abtahi, H.; Asadpour, L.; Ghazavi, H.; Pourmohammadi, R.; Maleki, M.; Ghadimi, K.	2019
Association of Demographic and Hearing-Related Factors with Cochlear Implant-Related Quality of Life	McRackan, T. R.; Hand, B. N.; Velozo, C. A.; Dubno, J. R.	2019
Association between Flat-Panel Computed Tomographic Imaging-Guided Place-Pitch Mapping and Speech and Pitch Perception in Cochlear Implant Users	Jiam, N. T.; Gilbert, M.; Cooke, D.; Jiradejvong, P.; Barrett, K.; Caldwell, M.; Limb, C. J.	2019
Clinical performance evaluation of a personal sound amplification product vs a basic hearing aid and a premium hearing aid	Cho, Y. S.; Park, S. Y.; Seol, H. Y.; Lim, J. H.; Cho, Y. S.; Hong, S. H.; Moon, I. J.	2019
Speech Perception with Spectrally Non-overlapping Maskers as Measure of Spectral Resolution in Cochlear Implant Users	O'Neill, E. R.; Kreft, H. A.; Oxenham, A. J.	2019
Pre-operative Brain Imaging Using Functional Near-Infrared Spectroscopy Helps Predict Cochlear Implant Outcome in Deaf Adults	Anderson, C. A.; Wiggins, I. M.; Kitterick, P. T.; Hartley, D. E. H.	2019
A Site-Selection Strategy Based on Polarity Sensitivity for Cochlear Implants: Effects on Spectro-Temporal Resolution and Speech Perception	Goehring, T.; Archer-Boyd, A.; Deeks, J. M.; Arenberg, J. G.; Carlyon, R. P.	2019
Older Adults With a Combination of Vision and Hearing Impairment Experience Higher Rates of Cognitive Impairment, Functional Dependence, and Worse Outcomes Across a Set of Quality Indicators	Davidson, J. G. S.; Guthrie, D. M.	2019

Translation, Adaptation and Cross-Cultural Validation of Hearing Handicap Inventory for Adult in Malay Language	Zam, Tzhbtz; Dzulkarnain, A. A. A.; Rahmat, S.; Jusoh, M.	2019
Neural tracking of the speech envelope in cochlear implant users	Somers, B.; Verschueren, E.; Francart, T.	2019
First report of quality of life in adults with neurofibromatosis 2 who are deafened or have significant hearing loss: results of a live-video randomized control trial	Funes, C. J.; Mace, R. A.; Macklin, E. A.; Plotkin, S. R.; Jordan, J. T.; Vranceanu, A. M.	2019
Speech in noise understanding in patients with vestibular schwannoma	Vaisbuch, Y.; Ali, N.; Qian, J. Z.; Gianakas, S. P.; Fitzgerald, M. B.	2019
Gain adjustment at tinnitus pitch to manage both tinnitus and speech perception in noise	Shetty, H. N.; Pottackal, J. M.	2019
Determinants of Self-Efficacy among Individuals who are Hard-of-Hearing	Cuevas, S.; Chen, R. K.; Vang, C.; Saladin, S. P.	2019
Interpersonal communication and psychological well-being among couples coping with sensory loss: The mediating role of perceived spouse support	Hofsoe, S. M.; Lehane, C. M.; Wittich, W.; Hilpert, P.; Dammeyer, J.	2019
Benefits and Shortcomings of Direct-to-Consumer Hearing Devices: Analysis of Large Secondary Data Generated From Amazon Customer Reviews	Manchaiah, V.; Amlani, A. M.; Bricker, C. M.; Whitfield, C. T.; Ratinaud, P.	2019
Longitudinal Relationships Between Decline in Speech-in-Noise Recognition Ability and Cognitive Functioning: The Longitudinal Aging Study Amsterdam	Pronk, M.; Lissenberg-Witte, B. I.; van der Aa, H. P. A.; Comijs, H. C.; Smits, C.; Lemke, U.; Zekveld, A. A.; Kramer, S. E.	2019
Identification of the Spectrotemporal Modulations That Support Speech Intelligibility in Hearing-Impaired and Normal-Hearing Listeners	Venezia, J. H.; Martin, A. G.; Hickok, G.; Richards, V. M.	2019
Eliciting Naturalistic Conversations: A Method for Assessing Communication Ability, Subjective Experience, and the Impacts of Noise and Hearing Impairment	Beechey, T.; Buchholz, J. M.; Keidser, G.	2019

The Circle of Care for Older Adults With Hearing Loss and Comorbidities: A Case Study of a Geriatric Audiology Clinic	Dupuis, K.; Reed, M.; Bachmann, F.; Lemke, U.; Pichora-Fuller, M. K.	2019
Predicting Speech Recognition Using the Speech Intelligibility Index and Other Variables for Cochlear Implant Users	Lee, S.; Mendel, L. L.; Bidelman, G. M.	2019
The Impact of Communication Impairments on the Social Relationships of Older Adults: Pathways to Psychological Well-Being	Palmer, A. D.; Carder, P. C.; White, D. L.; Saunders, G.; Woo, H.; Graville, D. J.; Newsom, J. T.	2019
Age Effects on Concurrent Speech Segregation by Onset Asynchrony	Stuckenberg, M. V.; Nayak, C. V.; Meyer, B. T.; Volker, C.; Hohmann, V.; Bendixen, A.	2019
Poorer Speech Reception Threshold in Noise Is Associated With Lower Brain Volume in Auditory and Cognitive Processing Regions	Rudner, M.; Seeto, M.; Keidser, G.; Johnson, B.; Ronnberg, J.	2019
Speech recognition as a function of the number of channels in perimodiolar electrode recipients	Berg, K. A.; Noble, J. H.; Dawant, B. M.; Dwyer, R. T.; Labadie, R. F.; Gifford, R. H.	2019
Contralateral routing of signal yields significant speech in noise benefit for unilateral cochlear implant recipients	Dwyer, R. T.; Kessler, D.; Butera, I. M.; Gifford, R. H.	2019
Evaluation of the efficacy of a dual variable speed compressor over a single fixed speed compressor	Kuk, F.; Slugocki, C.; Korhonen, P.; Seper, E.; Hau, O.	2019
Applying the COM-B Model to Assess the Usability of Smartphone-Connected Listening Devices in Adults with Hearing Loss	Maidment, D. W.; Ali, Y. H. K.; Ferguson, M. A.	2019
An evaluation of hearing aid beamforming microphone arrays in a noisy laboratory setting	Picou, E. M.; Ricketts, T. A.	2019
The effects of extended input dynamic range on laboratory and field-trial evaluations in adult hearing aid users	Plyler, P. N.; Easterday, M.; Behrens, T.	2019
The effects of nonlinear frequency compression and digital noise reduction on word recognition and satisfaction ratings in noise in adult hearing aid users	Plyler, P. N.; Tardy, B.; Hedrick, M.	2019

Hearing screening in the community	Saunders, G. H.; Frederick, M. T.; Silverman, S. C.; Penman, T.; Gardner, A.; Chisolm, T. H.; Escabi, C. D.; Oree, P. H.; Westermann, L. C.; Sanchez, V. A.; Arnold, M. L.	2019
Tracking of noise tolerance to predict hearing aid satisfaction in loud noisy environments	Seper, E.; Kuk, F.; Korhonen, P.; Slugocki, C.	2019
Difference in Speech Recognition between a Default and Programmed Telecoil Program	Ledda, K. T.; Valente, M.; Oeding, K.; Kallogjeri, D.	2019
Monitoring Hearing in an Infectious Disease Clinic with mHealth Technologies	Brittz, M.; Heinze, B.; Mahomed-Asmail, F.; Swanepoel, D.; Stoltz, A.	2019
Psychometric comparison of the hearing in noise test and the American English matrix test	Harianawala, J.; Galster, J.; Hornsby, B.	2019
Influence of instantaneous compression on recognition of speech in noise with temporal dips	Rasetshwane, D. M.; Raybine, D. A.; Kopun, J. G.; Gorga, M. P.; Neely, S. T.	2019
Application of wireless contralateral routing of signal technology in unilateral cochlear implant users with bilateral profound hearing loss	Snapp, H. A.; Hoffer, M. E.; Spahr, A.; Rajguru, S.	2019
Speech recognition in noise in single-sided deaf cochlear implant recipients using digital remote wireless microphone technology	Wesarg, T.; Arndt, S.; Wiebe, K.; Schmid, F.; Huber, A.; Mulder, H. E.; Laszig, R.; Aschendorff, A.; Speck, I.	2019
A comparison of word-recognition performances on the Auditec and VA recorded versions of Northwestern University Auditory Test no. 6 by young listeners with normal hearing and by older listeners with sensorineural hearing loss using a randomized presentation-level paradigm	Wilson, R. H.	2019

Benefits of compression amplification in telephone bluetooth-assistive listening devices for people with hearing loss	Luo, C. H.; Chang, H. Y.; Lo, T. S.; Tai, C. C.	2019
Visual Reliance during Speech Recognition in Cochlear Implant Users and Candidates	Moberly, A. C.; Vasil, K. J.; Ray, C.	2019
Understanding functional, behavioral, and psychosocial factors for screening and interventions in a large population of older adults	Lee, D. R.; Lo, J. C.; Ramalingam, N. D.; Gordon, N. P.	2019
Long-term performance of hearing aids patients based on pre-treatment hearing ability	Goel, A. R.; Bruce, H.; Alexiades, G.	2019
Difficulty Hearing Is Associated With Low Levels of Patient Activation	Chang, J. E.; Weinstein, B. E.; Chodosh, J.; Greene, J.; Blustein, J.	2019
Feasibility of an Intervention to Support Hearing and Vision in Dementia: The SENSE-Cog Field Trial	Hooper, E.; Simkin, Z.; Abrams, H.; Camacho, E.; Charalambous, A. P.; Collin, F.; Constantinidou, F.; Dawes, P.; Elliott, R.; Falkingham, S.; Frison, E.; Hann, M.; Helmer, C.; Himmelsbach, I.; Hussain, H.; Marie, S.; Montecelo, S.; Reeves, D.; Regan, J.; Thodi, C.; Wolski, L.; Leroi, I.	2019
Long-term Average Speech Spectra of Postlingual Cochlear Implant Users	Yuksel, M.; Gunduz, B.	2019
Long-Term Outcome of Hearing Rehabilitation With An Active Middle Ear Implant	Brkic, F. F.; Riss, D.; Auinger, A.; Zoerner, B.; Arnoldner, C.; Baumgartner, W. D.; Gstoettner, W.; Vyskocil, E.	2019
Arachnoid cysts of the internal auditory canal: An underappreciated entity?	Ungar, O. J.; Franck, M.; Nadol, J. B.; Santos, F.	2019

The pattern of hearing outcome following surgery of the semicircular canals	Wolfovitz, A.; Grobman, A. B.; Babcock, T. A.; Angeli, S. I.	2019
Multifactorial positive influence of cochlear implantation on patients with single-sided deafness	Haussler, S. M.; Kopke, V.; Knopke, S.; Grabel, S.; Olze, H.	2019
The Size of Internal Auditory Canal Diverticula Is Unrelated to Degree of Hearing Loss	Muelleman, T. J.; Pippin, K.; Shew, M.; Villwock, M.; Lin, J.; Quesnel, A. M.; Ledbetter, L.; Staecker, H.	2019
Adverse effect of self-reported hearing disability in elderly Italians: Results from the InveCe.Ab study	Vaccaro, R.; Zaccaria, D.; Colombo, M.; Abbondanza, S.; Guaita, A.	2019
Musical training improves the ability to understand speech-in-noise in older adults	Zendel, B. R.; West, G. L.; Belleville, S.; Peretz, I.	2019
Hearing-impaired listeners show increased audiovisual benefit when listening to speech in noise	Puschmann, S.; Daeglau, M.; Stropahl, M.; Mirkovic, B.; Rosemann, S.; Thiel, C. M.; Debener, S.	2019
Hearing loss and verbal memory assessment among older adults	Wong, C. G.; Rapport, L. J.; Billings, B. A.; Ramachandran, V.; Stach, B. A.	2019
Hearing Response Following Internal Auditory Canal Decompression in Neurofibromatosis Type 2	Bonne, N. X.; Risoud, M.; Hoa, M.; Lemesre, P. E.; Aboukais, R.; Rhun, E. L.; Dubrulle, F.; Baroncini, M.; Lejeune, J. P.; Vincent, C.	2019

A Retrospective Cohort Study of Longitudinal Audiologic Assessment in Single and Fractionated Stereotactic Radiosurgery for Vestibular Schwannoma	Khattab, M. H.; Sherry, A. D.; Whitaker, R.; Wharton, D. M.; Weaver, K. D.; Chambless, L. B.; Cmelak, A. J.; Attia, A.	2019
The Audibility of Low Vision Devices with Speech Output Used by Older Adults with Dual Sensory Impairment	St-Amour, L.; Jarry, J.; Wittich, W.	2019
Electro-Natural Stimulation in Partial Deafness Treatment of Adult Cochlear Implant Users: Long-Term Hearing Preservation Results	Skarzynski, H.; Lorens, A.; Dziendziel, B.; Rajchel, J. J.; Matusiak, M.; Skarzynski, P. H.	2019
Evaluation of quality of life in patients after cochlear implantation surgery in 2014-2017	Czerniejewska-Wolska, H.; Kalos, M.; Gawlowska, M.; Sekula, A.; Mickiewicz, P.; Wiskirska-Woznica, B.; Karlik, M.	2019
Bevacizumab for Hearing Preservation in Neurofibromatosis Type 2: Emphasis on Patient-Reported Outcomes and Toxicities	Sverak, P.; Adams, M. E.; Haines, S. J.; Levine, S. C.; Nascene, D.; Sommer, K.; Dusenbery, K.; Huang, T. C.; Moertel, C.	2019
The Relationship of Cardiometabolic Risk and Auditory Processing among African Americans: The Jackson Heart Study	Smith, E.; Bishop, C. E.; Spankovich, C.; Su, D.; Valle, K.; Schweinfurth, J.	2019
Determining the Sample Size for Future Trials of Hearing Instruments for Unilaterally Deaf Adults: An Application of Network Meta-analysis	Gaunt, A. C.; Kitterick, P. T.	2019
Comprehensive Analysis of Factors Leading to Poor Performance in Prelingual Cochlear Implant Recipients	Panda, S.; Sikka, K.; Singh, V.; Agarwal, S.; Kumar, R.; Thakar, A.; Sharma, S. C.	2019

The Benefit of a Wireless Contralateral Routing of Signals (CROS) Microphone in Unilateral Cochlear Implant Recipients	Kurien, G.; Hwang, E.; Smilsky, K.; Smith, L.; Lin, V. Y. W.; Nedzelski, J.; Chen, J. M.	2019
Training of Speech Perception in Noise in Pre-Lingual Hearing Impaired Adults With Cochlear Implants Compared With Normal Hearing Adults	Bugannim, Y.; Roth, D. A. E.; Zechoval, D.; Kishon-Rabin, L.	2019
Association of Speech Processor Technology and Speech Recognition Outcomes in Adult Cochlear Implant Users	Dixon, P. R.; Shipp, D.; Smilsky, K.; Lin, V. Y.; Le, T.; Chen, J. M.	2019
Cochlear Implantation in Patients With Single-sided Deafness After the Translabyrinthine Resection of the Vestibular Schwannoma-Presented at the Annual Meeting of ADANO 2016 in Berlin	Klenzner, T.; Glaas, M.; Volpert, S.; Jansen, N.; Kristin, J.; Schipper, J.	2019
Individual Hearing Preservation Cochlear Implantation Using the Concept of Partial Insertion	Lenarz, T.; Timm, M. E.; Salcher, R.; Buchner, A.	2019
Efficacy and Safety of AM-111 in the Treatment of Acute Unilateral Sudden Deafness-A Double-blind, Randomized, Placebo-controlled Phase 3 Study	Staecker, H.; Jokovic, G.; Karpishchenko, S.; Kienle-Gogolok, A.; Krzyzaniak, A.; Lin, C. D.; Navratil, P.; Tzvetkov, V.; Wright, N.; Meyer, T.	2019
Long-term Benefit of Unilateral Cochlear Implantation on Quality of Life and Speech Perception in Bilaterally Deafened Patients	Haussler, S. M.; Knopke, S.; Wiltner, P.; Ketterer, M.; Grabel, S.; Olze, H.	2019
Audiologic Improvement Following MCF Approach for Spontaneous Cerebrospinal Fluid Leaks	Alwani, M.; Bandali, E.; Van Buren, L.; Yates, C. W.; Nelson, R. F.	2019
Prognostic Factors of Long-Term Hearing Preservation in Small and Medium-Sized Vestibular Schwannomas After Microsurgery	Huo, Z.; Chen, J.; Wang, Z.; Zhang, Z.; Wu, H.	2019
Speech Perception Outcomes for Adult Cochlear Implant Recipients Using a Lateral Wall or Perimodiolar Array	Moran, M.; Vandali, A.; Briggs, R. J. S.; Dettman, S.; Cowan, R. S. C.; Dowell, R. C.	2019

Datalogging Statistics and Speech Recognition During the First Year of Use in Adult Cochlear Implant Recipients	Schvartz-Leyzac, K. C.; Conrad, C. A.; Zwolan, T. A.	2019
Predicting cochlear dead regions in patients with hearing loss through a machine learning-based approach: A preliminary study	Chang, Y. S.; Park, H.; Hong, S. H.; Chung, W. H.; Cho, Y. S.; Joon Moon, I. I.	2019
A comparison between wireless CROS/BiCROS and soft-band BAHA for patients with unilateral hearing loss	Choi, J. E.; Ma, S. M.; Park, H.; Cho, Y. S.; Hong, S. H.; Moon, I. J.	2019
Speech-in-noise representation in the aging midbrain and cortex: Effects of hearing loss	Presacco, A.; Simon, J. Z.; Anderson, S.	2019
Effects of frontal-executive dysfunction on self-perceived hearing handicap in the elderly with mild cognitive impairment	Lee, S. J.; Kim, H.; Kim, L. S.; Kim, J. H.; Park, K. W.	2019
Sensitivity of EQ-5D-3L, HUI2, HUI3, and SF-6D to changes in speech reception and tinnitus associated with cochlear implantation	Summerfield, A. Q.; Barton, G. R.	2019
Immediate improvement of speech-in-noise perception through multisensory stimulation via an auditory to tactile sensory substitution	Ciesla, K.; Wolak, T.; Lorens, A.; Heimler, B.; Skarzynski, H.; Amedi, A.	2019
WHODAS 2.0-BO: normative data for the assessment of disability in older adults	Ferrer, M. L. P.; Perracini, M. R.; Rebustini, F.; Buchalla, C. M.	2019
Perceptions of deaf subjects about communication in Primary Health Care	Santos, A. S.; Portes, A. J. F.	2019
Effect of statins on hearing function and subjective tinnitus in hyperlipidemic patients	Yucel, H.; Yucel, A.; Arbag, H.; Cure, E.; Eryilmaz, M. A.; Ozer, A. B.	2019
The effect of age-related hearing loss and listening effort on resting state connectivity	Rosemann, S.; Thiel, C. M.	2019
Pilot Comparison of Adjustment Protocols of Personal Sound Amplification Products	Reed, N. S.; Oliver, A.; Srinivasan, N. K.; Lin, F. R.; Korczak, P. A.	2019

Hearing Impairment and Quality of Life in Adults with Asymmetric Hearing Loss: Benefits of Bimodal Stimulation	Sanhueza, I.; Manrique-Huarte, R.; Calavia, D.; Huarte, A.; Manrique, M.	2019
Trajectories of Limitations in Instrumental Activities of Daily Living in Frail Older Adults With Vision, Hearing, or Dual Sensory Loss	Mueller-Schotte, S.; Zuithoff, N. P. A.; van der Schouw, Y. T.; Schuurmans, M. J.; Bleijenberg, N.	2019
Audiometric Age-Related Hearing Loss and Cognition in the Hispanic Community Health Study	Golub, J. S.; Brickman, A. M.; Ciarleglio, A. J.; Schupf, N.; Luchsinger, J. A.	2019
Comparison of Frequency Transposition and Frequency Compression for People With Extensive Dead Regions in the Cochlea	Salorio-Corbetto, M.; Baer, T.; Moore, B. C. J.	2019
Computer-Based Connected-Text Training of Speech-in-Noise Perception for Cochlear Implant Users	Green, T.; Faulkner, A.; Rosen, S.	2019
The Important Role of Contextual Information in Speech Perception in Cochlear Implant Users and Its Consequences in Speech Tests	Dingemanse, J. G.; Goedegebure, A.	2019
Individual Variations in Effort: Assessing Pupillometry for the Hearing Impaired	Wagner, A. E.; Nagels, L.; Toffanin, P.; Opie, J. M.; Baskent, D.	2019
A Competing Voices Test for Hearing-Impaired Listeners Applied to Spatial Separation and Ideal Time-Frequency Masks	Bramslow, L.; Vatti, M.; Rossing, R.; Naithani, G.; Henrik Pontoppidan, N.	2019
Selecting Bolt-On Dimensions for the EQ-5D: Examining Their Contribution to Health-Related Quality of Life	Finch, A. P.; Brazier, J. E.; Mukuria, C.	2019
Is tinnitus a major concern in individuals with auditory neuropathy spectrum disorder? - Questionnaire based study	Prabhu, P.	2019
Prevalence of and Characteristics Associated With Self-reported Good Hearing in a Population With Elevated Audiometric Thresholds	Curti, S. A., Taylor, E. N., Su, D., & Spankovich, C.	2019
Intratympanic steroid therapy for refractory sudden sensory hearing loss: a 12-year experience with the Silverstein catheter	Vanwijck, F.; Rogister, F.; Pierre Barriat, S.; Camby, S.; Lefebvre, P.	2019

Benefit of hearing aid use in the elderly: the impact of age, cognition and hearing impairment	Tognola, G.; Mainardi, A.; Vincenti, V.; Cuda, D.	2019
Hearing outcomes of the active bone conduction system Bonebridge in conductive or mixed hearing loss	Carnevale, C.; Til-Perez, G.; Arancibia-Tagle, D. J.; Tomas-Barberan, M. D.; Sarria-Echegaray, P. L.	2019
Auditory and Cognitive Attributes of Hearing Aid Acclimatization in Individuals With Sensorineural Hearing Loss	Megha,; Maruthy, S.	2019
Prospective Evaluation of Cognitive Functions After Rehabilitation With Cochlear Implant or Hearing Aids: Preliminary Results of a Multicentric Study on Elderly Patients	Anzivino, R.; Conti, G.; Di Nardo, W.; Fetoni, A. R.; Picciotti, P. M.; Marra, C.; Guglielmi, V.; Fortunato, S.; Forli, F.; Paludetti, G.; Berrettini, S.	2019
The Effects of Amplification on Listening Self-Efficacy in Adults With Sensorineural Hearing Loss	Kawaguchi, L.; Wu, Y. H.; Miller, C.	2019
Relationship Between Domain-Specific Cognitive Function and Speech-in-Noise Performance in Older Adults: The Atherosclerosis Risk in Communities Hearing Pilot Study	Mamo, S. K.; Reed, N. S.; Sharrett, A. R.; Albert, M. S.; Coresh, J.; Mosley, T. H.; Knopman, D.; Lin, F. R.; Deal, J. A.	2019
Evidence-Based Recommendation for Bilateral Cochlear Implantation in Adults	Au, A.; Dowell, R. C.	2019
Speech Recognition of Cochlear Implant Users Inside a Noisy Helicopter Environment	Caldeira, J. M. A.; Goffi-Gomez, M. V. S.; Imamura, R.; Bento, R. F.	2019
Cochlear Implantation with the CI512 and CI532 Precurved Electrode Arrays: One-Year Speech Recognition and Intraoperative Thresholds of Electrically Evoked Compound Action Potentials	Videhult Pierre, P.; Eklof, M.; Smeds, H.; Asp, F.	2019
Outcomes after Application of Active Bone Conducting Implants	Koro, E.; Werner, M.	2019

Application of Ambulatory Phonation Monitoring (APM) in the measurement of daily speaking-time and voice intensity before and after cochlear implant in deaf adult patients	Mozzanica, F.; Schindler, A.; Iacona, E.; Ottaviani, F.	2019
Self-reported hearing difficulty and its association with general, cognitive, and psychosocial health in the state of Arizona, 2015	Marrone, N.; Ingram, M.; Bischoff, K.; Burgen, E.; Carvajal, S. C.; Bell, M. L.	2019
The effect of delayed auditory feedback (DAF) and frequency altered feedback (FAF) on speech production: cochlear implanted versus normal hearing individuals	Taitelbaum-Swead, R.; Avivi, M.; Gueta, B.; Fostick, L.	2019
Objective, audiological and quality of life measures with the CI532 slim modiolar electrode	Hey, M.; Wesarg, T.; Mewes, A.; Helbig, S.; Hornung, J.; Lenarz, T.; Briggs, R.; Marx, M.; Ramos, A.; Stover, T.; Escude, B.; James, C. J.; Aschendorff, A.	2019
Controlled comparative clinical trial of hearing benefit outcomes for users of the Cochlear TM Nucleus^R 7 Sound Processor with mobile connectivity	Warren, C. D.; Nel, E.; Boyd, P. J.	2019
Benefit of directional microphones for unilateral, bilateral and bimodal cochlear implant users	Ernst, A.; Anton, K.; Brendel, M.; Battmer, R. D.	2019
Measures of Listening Effort Are Multidimensional	Alhanbali, S.; Dawes, P.; Millman, R. E.; Munro, K. J.	2019
Clinical Effectiveness of an At-Home Auditory Training Program: A Randomized Controlled Trial	Humes, L. E.; Skinner, K. G.; Kinney, D. L.; Rogers, S. E.; Main, A. K.; Quigley, T. M.	2019
Effects of Reverberation on the Relation Between Compression Speed and Working Memory for Speech-in-Noise Perception	Reinhart, P.; Zahorik, P.; Souza, P.	2019

Understanding Variability in Individual Response to Hearing Aid Signal Processing in Wearable Hearing Aids	Souza, P.; Arehart, K.; Schoof, T.; Anderson, M.; Strori, D.; Balmert, L.	2019
Effects of Age and Hearing Loss on the Recognition of Emotions in Speech	Christensen, J. A.; Sis, J.; Kulkarni, A. M.; Chatterjee, M.	2019
Hearing Impairment and Perceived Clarity of Predictable Speech	Signoret, C.; Rudner, M.	2019
Cochlear implant outcomes in the elderly: a uni- and multivariate analyses of prognostic factors	Favaretto, N.; Marioni, G.; Brotto, D.; Sorrentino, F.; Gheller, F.; Castiglione, A.; Montino, S.; Giacomelli, L.; Trevisi, P.; Martini, A.; Bovo, R.	2019
The first results of a totally implanted active middle ear device	Peixoto, M. D. C.; Miranda, C.; Bento, M.; Oliveira, S.; Pratas, R.; Correia da Silva, V.	2019
Flat-based fitting: the evaluation and usefulness of a new strategy-based fitting approach for cochlear implants	Kurz, A.; Hagen, R.; van de Heyning, P.; Mertens, G.	2019
The impact of etiology and duration of deafness on speech perception outcomes in SSD patients	Kurz, A.; Grubenbecher, M.; Rak, K.; Hagen, R.; Kuhn, H.	2019
Evaluation of a system for enhancing mobile telephone communication for people with hearing loss	Moore, B. C. J.; Shaw, S.; Griffiths, S.; Stone, M. A.; Sherlock, Z.	2019
Adults who report difficulty hearing speech in noise: an exploration of experiences, impacts and coping strategies	Pang, J.; Beach, E. F.; Gilliver, M.; Yeend, I.	2019
The relationship between peripheral hearing loss and higher order listening function on cognition in older Australians	Nixon, G.; Sarant, J. Z.; Tomlin, D.; Dowell, R.	2019
ForwardFocus with cochlear implant recipients in spatially separated and fluctuating competing signals - introduction of a reference metric	Hey, M.; Hocke, T.; Bohnke, B.; Mauger, S. J.	2019

Effects of noise and reverberation on speech recognition with variants of a multichannel adaptive dynamic range compression scheme	Rallapalli, V. H.; Alexander, J. M.	2019
Quality of life in persons with hearing loss: a study of patients referred to an audiological service	Nordvik, O.; Heggdal, P. O. L.; Brannstrom, J. K.; Hjermstad, M. J.; Aarstad, A. K.; Aarstad, H. J.	2019
Audiologic, surgical and subjective outcomes of active transcutaneous bone conduction implant system (Bonebridge)	Oh, S. J.; Goh, E. K.; Choi, S. W.; Lee, S.; Lee, H. M.; Lee, I. W.; Kong, S. K.	2019
Are French Fries a Vegetable? Lexical Typicality Judgement Differences in Deaf and Hearing Learners	Crowe, K.; Marschark, M.	2019
Degree of Hearing Loss Affects Bilateral Hearing Aid Benefits in Ecologically Relevant Laboratory Conditions	Ricketts, T. A.; Picou, E. M.; Shehorn, J.; Dittberner, A. B.	2019
Systematic Evaluation of Self-Reported Hearing Ability in Six Dimensions Before and After a Hearing Aid Trial	de Ronde-Brons, I.; Soede, W.; Dreschler, W.	2019
Making Sense of Sentences: Top-Down Processing of Speech by Adult Cochlear Implant Users	Moberly, A. C.; Reed, J.	2019
Speech Perception in Noise With Formant Enhancement for Older Listeners	Guan, J.; Liu, C.	2019
The Production of Question Intonation by Young Adult Cochlear Implant Users: Does Age at Implantation Matter?	Lehnert-LeHouillier, H.; Spencer, L. J.; Machmer, E. L.; Burchell, K. L.	2019
Cognitive factors contribute to speech perception in cochlear-implant users and age-matched normal-hearing listeners under vocoded conditions	O'Neill, E. R.; Kreft, H. A.; Oxenham, A. J.	2019
Lexical bias in word recognition by cochlear implant listeners	Gianakas, S. P.; Winn, M. B.	2019
Audio-visual integration in cochlear implant listeners and the effect of age difference	Zhou, X.; Innes-Brown, H.; McKay, C. M.	2019
Individualized frequency importance functions for listeners with sensorineural hearing loss	Yoho, S. E.; Bosen, A. K.	2019
The optimal threshold for removing noise from speech is similar across normal and impaired hearing-a time-frequency masking study	Healy, E. W.; Vasko, J. L.; Wang, D.	2019
Recognition of emotional prosody by Mandarin-speaking adults with cochlear implants	Pak, C. L.; Katz, W. F.	2019

Contributions of lexical tone to Mandarin sentence recognition in hearing-impaired listeners under noisy conditions	Li, N.; Wang, S.; Wang, X.; Xu, L.	2019
AutoAdaptive: A Noise Level-Sensitive Beamformer for MED EL Cochlear Implant Patients	Dorman, M. F.; Natale, S. C.	2019
Auditory-frontal Channeling in alpha and beta Bands is Altered by Age-related Hearing Loss and Relates to Speech Perception in Noise	Price, C. N.; Alain, C.; Bidelman, G. M.	2019
Effect of compression release time of a hearing aid on sentence recognition and the quality judgment of speech	Shetty, H. N.; Raju, S.	2019
Selected Cognitive Factors Associated with Individual Variability in Clinical Measures of Speech Recognition in Noise Amplified by Fast-Acting Compression Among Hearing Aid Users	Yumba, W. K.	2019
Matched Cohort Comparison Indicates Superiority of Precurved Electrode Arrays	Holder, J. T.; Yawn, R. J.; Nassiri, A. M.; Dwyer, R. T.; Rivas, A.; Labadie, R. F.; Gifford, R. H.	2019
Evaluating the Long-Term Hearing Outcomes of Cochlear Implant Users With Single-Sided Deafness	Tavora-Vieira, D.; Rajan, G. P.; Van de Heyning, P.; Mertens, G.	2019
Validation of the Hearing Handicap Inventory for Adults Scale for Spanish-Speaking Patients	Carrillo, A.; Medina, M. D. M.; Polo, R.; Alonso, D.; Vaca, M.; Muriel, A.; Fernandez, B.; Rivera, T.; Cobeta, I.	2019
A Comparative Study of a Novel Adhesive Bone Conduction Device and Conventional Treatment Options for Conductive Hearing Loss	Skarzynski, P. H.; Ratuszniak, A.; Osinska, K.; Koziel, M.; Krol, B.; Cywka, K. B.; Skarzynski, H.	2019
Datalogging Statistics and Speech Recognition During the First Year of Use in Adult Cochlear Implant Recipients	Schvartz-Leyzac, K. C.; Conrad, C. A.; Zwolan, T. A.	2019
Hearing Preservation With the Use of Flex20 and Flex24 Electrodes in Patients With Partial Deafness	Skarzynski, P. H.; Skarzynski, H.; Dziendziel, B.; Rajchel, J. J.; Gos, E.; Lorens, A.	2019
Speech Perception Outcomes in Transcutaneous Versus Percutaneous Bone Conduction Stimulation in Individuals With Single-sided Deafness	Snapp, H. A.; Morgenstein, K. E.; Kuzbyt, B.	2019

Triphasic Pulses in Cochlear Implant Patients With Facial Nerve Stimulation	Braun, K.; Walker, K.; Surth, W.; Lowenheim, H.; Tropitzsch, A.	2019
The Role of Sex on Early Cochlear Implant Outcomes	Raymond, M. J.; Ballestas, S. A.; Wise, J. C.; Vivas, E. X.	2019
Speech Perception Outcomes for Adult Cochlear Implant Recipients Using a Lateral Wall or Perimodiolar Array	Moran, M.; Vandali, A.; Briggs, R. J. S.; Dettman, S.; Cowan, R. S. C.; Dowell, R. C.	2019
Auditory Performances in Older and Younger Adult Cochlear Implant Recipients: Use of the HEARRING Registry	Hofkens-Van den Brandt, A.; Mertens, G.; Gilles, A.; Fransen, E.; Lassaletta, L.; Gavilan, J.; Calvino, M.; Yanov, Y.; Kuzovkov, V.; Kliachko, D.; Zernotti, M.; Gregorio, Dmfd; Van Rompaey, V.; Van de Heyning, P.; Sugarova, S.	2019
Age-Dependent Psychological Factors Influencing the Outcome of Cochlear Implantation in Elderly Patients	Knopke, S.; Haussler, S.; Grabel, S.; Wetterauer, D.; Ketterer, M.; Fluger, A.; Szczepek, A. J.; Olze, H.	2019
Speech perception in noise: Impact of directional microphones in users of combined electric-acoustic stimulation	Weissgerber, T.; Stover, T.; Baumann, U.	2019
A psychoacoustic application for the adjustment of electrical hearing thresholds in cochlear implant patients	Plesch, J.; Ernst, B. P.; Strieth, S.; Rader, T.	2019
Electro-haptic enhancement of speech-in-noise performance in cochlear implant users	Fletcher, M. D.; Hadeedi, A.; Goehring, T.; Mills, S. R.	2019

Cochlear implants in single-sided deafness - clinical results of a Swiss multicentre study	Peter, N.; Kleinjung, T.; Probst, R.; Hemsley, C.; Veraguth, D.; Huber, A.; Caversaccio, M.; Kompis, M.; Mantokoudis, G.; Senn, P.; Wimmer, W.	2019
Does Asymmetric Hearing Loss Affect the Ability to Understand in Noisy Environments?	Barona, R.; Vizcaino, J. A.; Krstulovic, C.; Barona, L.; Comeche, C.; Montalt, J.; Ubeda, M.; Polo, C.	2019
High-Frequency Sensorineural Hearing Loss Alters Cue-Weighting Strategies for Discriminating Stop Consonants in Noise	Varnet, L.; Langlet, C.; Lorenzi, C.; Lazard, D. S.; Micheyl, C.	2019
Discrimination of Gain Increments in Speech	Caswell-Midwinter, B.; Whitmer, W. M.	2019
Age-Related Temporal Processing Deficits in Word Segments in Adult Cochlear-Implant Users	Xie, Z.; Gaskins, C. R.; Shader, M. J.; Gordon-Salant, S.; Anderson, S.; Goupell, M. J.	2019
On the Interaction of Head and Gaze Control With Acoustic Beam Width of a Simulated Beamformer in a Two-Talker Scenario	Hladek, L.; Porr, B.; Naylor, G.; Lunner, T.; Owen Brimijoin, W.	2019
Spatial Speech-in-Noise Performance in Bimodal and Single-Sided Deaf Cochlear Implant Users	Williges, B.; Wesarg, T.; Jung, L.; Geven, L. I.; Radeloff, A.; Jurgens, T.	2019
